# Chemoenzymatic Synthesis of Structurally Diverse Terpenoids from Farnesyl Pyrophosphates Modified at the Central Alkene Unit

**DOI:** 10.1021/jacs.5c14636

**Published:** 2025-12-22

**Authors:** Henry Struwe, Christopher Slotman, Laurent Höft, Gerald Dräger, Jörn Droste, Jörg Fohrer, Katharina Hausmann, Sascha Beutel, Dominik Kolling, Jesko Köhnke, Andreas Kirschning

**Affiliations:** † Institute of Organic Chemistry, Leibniz University Hannover, Schneiderberg 1B, 30167 Hannover, Germany; ‡ Department of Chemistry, Technical University Darmstadt, Alarich-Weiss-Straße 4, 64287 Darmstadt, Germany; § Institute of Technical Chemistry, Leibniz University Hannover, Callinstraße 5, 30167 Hannover, Germany; ∥ Institute of Food Chemistry, Leibniz University Hannover, Callinstraße 5, 30167 Hannover, Germany; ⊥ Uppsala Biomedical Center (BMC), Uppsala University, Husargatan 3, 752 37 Uppsala, Sweden

## Abstract

Sesquiterpene synthases (STSs) enable cationic cascade reactions with farnesyl pyrophosphate (FPP) resulting in an immense variety of oligocyclic sesquiterpenes. Their substrate promiscuity allows access to new sesquiterpene carbon skeletons. We explored the ability of eight STSs to process three distinct synthetic FPP derivatives modified at the central isoprenyl unit. These include the incorporation of a keto group at C7 (“keto”-FPP), the relocation of the olefinic double bond into the methyl group (“iso”-FPP), and the shift of the double bond toward the aliphatic terminus of the FPP backbone and loss of the methyl group at C7 (“nor-iso” FPP). We report the enzymatic production of 18 new terpenoids, including a large variety of new oxaterpenoids. One of these is known as a late stage intermediate in the total synthesis of the sex pheromone periplanone B, which is secreted by females of the American cockroach *Periplaneta americana* to attract mates. Thus, a formal chemoenzymatic synthesis of this pheromone is disclosed.

## Graphical abstract

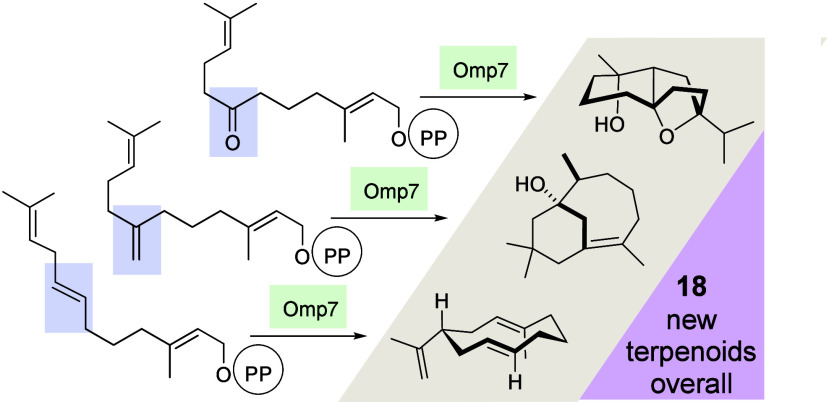

## Introduction

In recent years, terpene synthases (TSs) have sparked attention not only in the field of enzymology^1^ but also in chemoenzymatic approaches to expand what may be called the “terpenome”.^2,3^ Terpenoids are among the most structurally diverse families of natural products and elicit diverse biological effects, such as being antimicrobial or cytotoxic, and some of them are detected by olfaction.^4^ With over 10^5^ reported terpenoid entities, they are also the largest class of secondary metabolites.^1^ This immense structural diversity is achieved by highly complex cationic reaction cascades that are catalyzed by TSs, such as sesquiterpene synthases (STSs), which use the common linear isoprenoid precursor farnesyl pyrophosphate (FPP, **1**). The cationic species is formed via enzymatic cleavage of the pyrophosphate moiety, and during the ensuing reaction, on average, half of the carbon atoms experience a change in their hybridization, binding properties, and stereochemistry.^1,5^

Vibrant research on TSs has recently revealed that a significant number of STSs and diterpene synthases (DTSs) exhibit marked substrate promiscuity and could utilize unnatural linear isoprene precursors.^2,3^ A remarkable example was reported by Allemann and co-workers, who developed a chemoenzymatic approach toward the antimalaria drug artemisinine by employing a chemically modified FPP derivative and an amorphadiene synthase.^6^ In addition to chemical synthesis, enzymatic and chemoenzymatic approaches toward modified FPP derivatives, followed by TS-catalyzed cyclizations, have been pursued successfully.^7–11^

We^12–19^ and other groups^20–24^ have relied predominantly on the chemical synthesis of noncanonical FPP derivatives.

Some STSs, especially the presilphiperfolan-8-β-ol synthase (BcBOT2) from *Botrytis cinerea*, have shown enormous versatility and flexibility in dealing with non-natural substrates.^12,19,25^ In our hands, another STS proved to be almost as versatile. This concerns the whole family of Δ^6^-protoilludene synthases among which Omp7 from *Omphalotus olearius* turned out to be best suited.^13,16,18^ Naturally, Omp7 converts FPP (**1**) into the tricyclic sesquiterpene Δ^6^-protoilludene (**2**) (Scheme 1),^26^ but its substrate scope also included FPP ethers **3**, **8**, and **9** as well as “iso”-FPP **13**. Omp7 transformed FPP analogues **3**, **8**, and **9** into several oxygenated sesquiterpenes **4**–**7** and **10**–**12**, and the majority were di- and tricyclic. The enzyme also accepted **13** to yield the cyclodeca-1,6-diene **14**.^18^

**Scheme 1:**
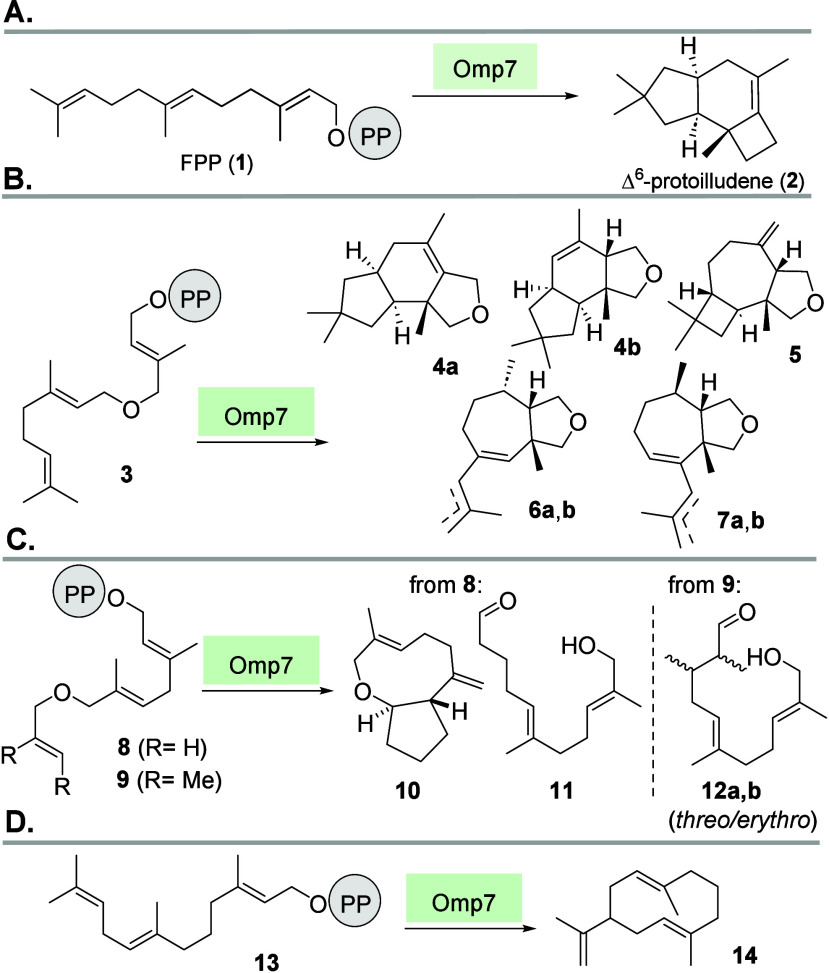
Omp7-Promoted Transformations of FPP **1** and FPP Derivatives 3, 8, 9 and 13^a^

a (A) Omp7-promoted transformation of FPP (**1**) to Δ^6^-protoilludene (**2**). (B) Omp7-promoted transformation of “FPP-ether” **3** to oxa-sesquiterpenoids **4**–**7**. (C) Omp7-promoted transformation of “FPP-ethers” **8** and **9** to oxygenated terpenoids **10**–**12**. (D) Omp7-promoted transformation of “*iso*”-FPP **13** to macrocyclic terpenoid **14**, named “germacrene F”.^13,16,18,26^

It has been documented that structural changes around the central olefinic double bond of FPP can provide new sesquiterpene scaffolds.^16,22,27^ This is particularly relevant for STSs that yield tricyclic products, requiring participation of the central olefinic double bond around C7 in the cation cascade. In the present work, we demonstrate that Omp7 is a highly promiscuous STS that enables a considerable expansion of the “terpenome”. Consequently, we therefore synthesized FPP derivatives **15**–**17**.^22,27^ To investigate the catalytic prowess of other members of the STS family of enzymes, we produced four additional enzymes: a caryolanol synthase (GCoA → (+)-caryolan-1-ol (**18**)),^28^ a viridoflorene/vetispiradiene synthase (Tps32 → vetispiradiene (**19**)),^29,30^ a β-cubebol synthase (Cop4 → β-cubebol (**20**)),^31^ and a patchoulol synthase (Pts → patchoulol (**21**)).^32^ In addition, we added three more STSs, namely, *Jungermania exsertifolia* C10-(*S*)-bicyclogermacrene synthase (JeSTS4 → *ent*-viridiflorol (**22**)),^33^ red macroalgae *Laurencia pacifica* prespatane synthase (LphTPS → prespatane (**23**)),^34^ and *Picea abies* longifolene synthase (PaTPS → longifolene (**24**))^35^ to the list of STSs. Noteworthily, these have not been studied in biotransformation with unnatural FPP derivatives so far. The syntheses of FPP derivatives **15**–**17** have been reported elsewhere,^11,12,27^ and the final steps in the preparation of the “keto”-FPP analogue **15** are covered in detail in the Supporting Information. Overall, our efforts yielded 18 new terpenoids, including a large variety of new oxaterpenoids and a late stage intermediate in the total synthesis of the American cockroach *Periplaneta americana* sex pheromone periplanone B.

## Results and Discussion

### Biotransformations

All eight STSs (Omp7, GCoA, Tps32, Cop4, Pts, JeSTS4, LphTPS, PaTPS (Figure 1B)) were heterologously produced in *E. coli* and purified to homogeneity for biochemical assays (see SI).^12,13^ Of these, Pts and the new STSs JeSTS4, LphTPS, and PaTPS showed significant solubility issues during enzyme purification, resulting in low protein yields.

**Figure 1:**
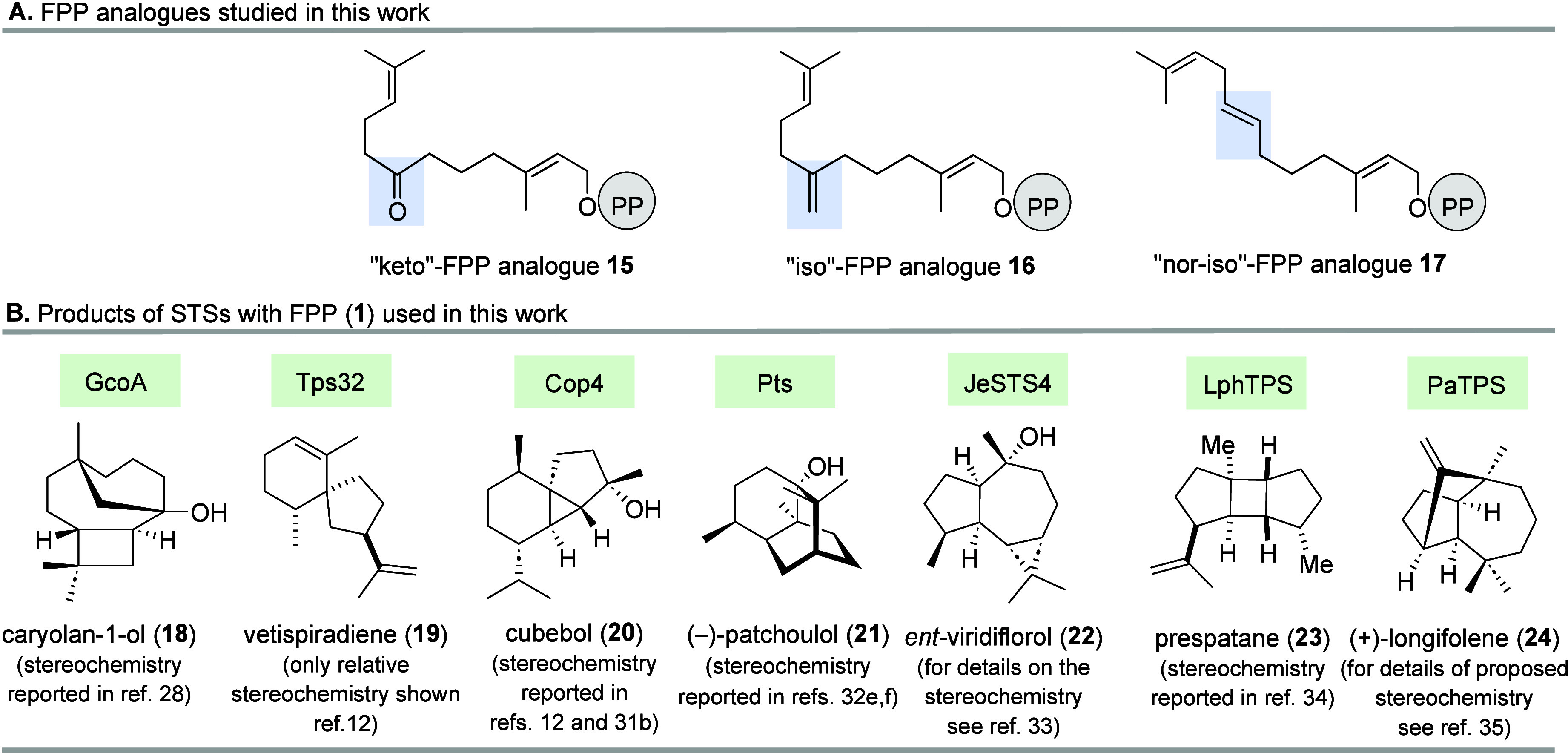
(A) Structures of FPP derivatives **15**–**17** used in this work. (B) Sesquiterpenes **18**–**24** formed from FPP (**1**) by sesquiterpene synthases (STS) used in this work (only major biotransformation products are shown).

*In vitro* enzyme assays to determine enzyme activity and substrate tolerance were performed on a small scale (*V* = 500 μL, *m*_STS_ = 0.05 mg, and *c*_Substrate_ = 150 μM) using the natural precursor FPP (**1**) (see SI). As suspected based on the difficulties in their purification, LphTPS and PaTPS showed comparably low turnovers in their respective biotransformations with the natural substrate FPP (**1**).

The preparative transformation of ketone **15** yielded sufficient quantities of new products with seven STSs investigated in this work (Scheme 2A). Only the conversion with Tps32 resulted in a complex mixture; therefore, the use of this STS was not pursued further. Omp7 yielded a bouquet of products, namely, cyclododecanone **25** and six more sesquiterpenoids that can be split into two subgroups. The first group consists of the tertiary alcohols **26a**,**b** and **27a**,**b**, whereby the hydroxyl group localized at the interface of the two rings originates from the keto functionality. The two diastereomeric terpenoids **26a** and **26b** differ in the localization of one of the two olefins and are *syn*-annulated. The third terpene alcohol **27b** corresponds constitutionally to the isomer **26b**, but here, the decahydronaphthalene backbone is *anti*-fused as in the fourth product of the series, namely, diol **27a**. The second subgroup is characterized by an additional ether bridge, which originates from the keto function, too. As such, the decahydronaphthalene backbone is also present in sesquiterpenoids **28a** and **28b**.

**Scheme 2:**
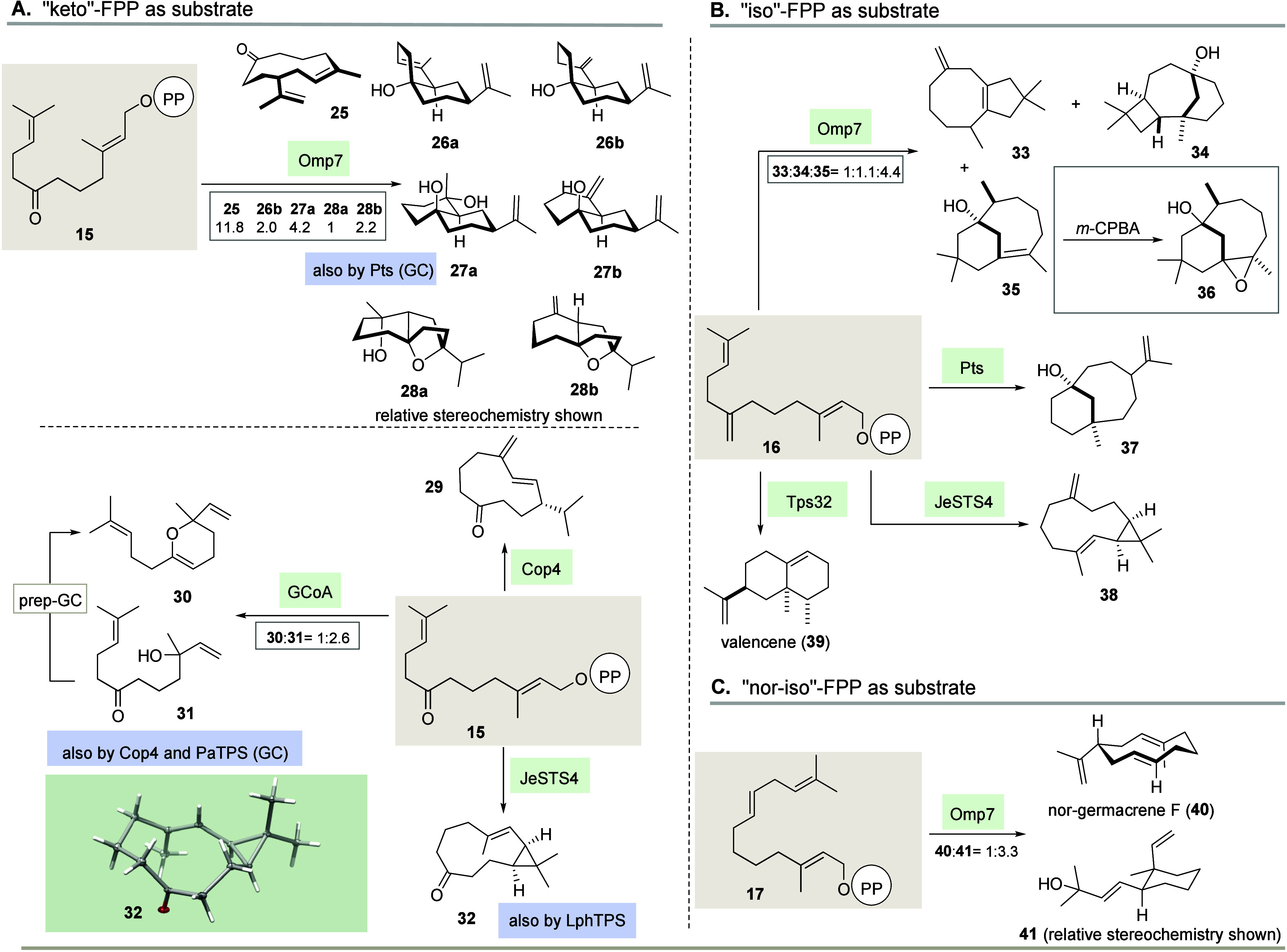
Formation of Products 25-41^a^

a (A) Products **25**–**32** isolated from biotransformations of STSs Omp7, Cop4, GCoA, and JeSTS4 with “keto”-FPP derivative **15**; (B) products **33**–**35** as well as **37**–**39** isolated from biotransformations of STSs Omp7, Pts, Tps32, and JeSTS4 with “iso”-FPP derivative **16**; (C) products **40** and **41** isolated from biotransformations of STS Omp7 with “nor-iso”-FPP derivative **17**. (The relative product ratios were calculated based on the GC chromatograms. In some cases (e.g., **26a** and **27b**), these could not be determined due to complete signal overlap. We sometimes observed variations in the relative product ratios depending on the chosen scale of biotransformation.)

Cop4 was found to generate cyclodecanone derivative **29**, while GCoA gave 4-dihydro-2*H*-pyran terpenoid **30** along hydroxyketone **31**. Interestingly, we also found this open-chain product in the reaction with PaTPS, which likely gave dihydropyran **30** only under the thermal conditions of GC analysis. The two STSs LphTPS and JeSTS4 are also able to convert FPP ketone **15**, whereby in both cases the terpenoid **32** was formed, which is characterized by a bicyclo[8.1.0]­undecan-4-one scaffold. Its constitution and stereochemistry were unequivocally assigned by X-ray analysis of **32**.^36a^ Omp7, Pts, JeSTS4, and Tps32 proved to be particularly suitable for the transformation of the FPP analogue “iso”-FPP **16** (Scheme 2B). Tree new terpenoids **33**–**35** were isolated from reactions with Omp7 (Figure 2). To facilitate structure elucidation of bridged sesquiterpenoid **35**, we epoxidized it chemically to give derivative **36** and collected and compared a second set of NMR data (see below). Biotransformation of **16** with Pts yielded the bridged bicyclic tertiary alcohol **37**, while JeSTS4 provided cyclopropyl terpenoid **38** as the main product. Tps32 transformed **16** into valencene (**39**) as the main product, which is a known aroma component of citrus fruit. In fact, valencene is a minor product in the *in vitro* transformation of FPP (**1**) by the sesquiterpene synthase Tps32, while with “iso”-FPP **16**, the product ratio of vetispiradiene (**19**) and valencene (**39**) is completely reversed (Figure 2). This raises interesting mechanistic questions, which are addressed below. “Nor-iso” FPP analogue **17** was a viable substrate for Omp7 only with nor-"germacrene F" (**40**) and cyclohexane derivative **41** as the main products (Scheme 2C).

**Figure 2:**
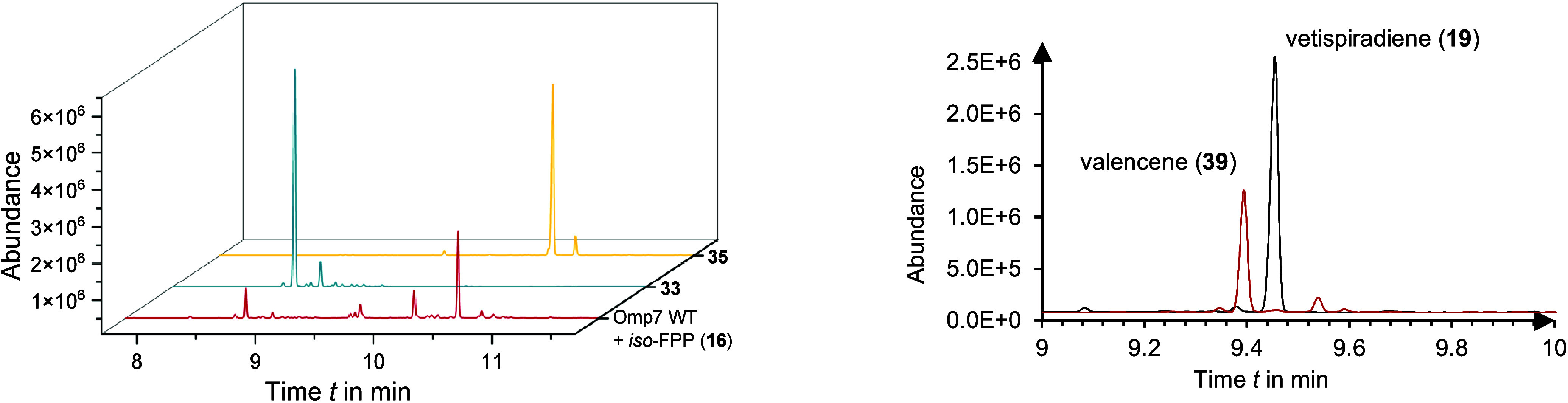
(Left) GC chromatograms of Omp7-promoted biotransformations with “*iso*”-FPP derivative **16** (red) and isolated sesquiterpenes **33** (blue) and **35** (orange). (Right) GC chromatograms collected from biotransformations (analytical scale) of Tps32 with FPP (**1**, black) and “iso”-FPP **16** (red).

In an effort to alter the product spectrum of our most versatile STS, Omp7, we sought to determine its crystal structure to enable precision protein engineering. Omp7 proved recalcitrant to crystallization, and we therefore resorted to an AlphaFold prediction of its structure.^36b^ Such models are notoriously unreliable for terpene synthases, and we were thus very careful in our analysis of the model, which suggested three mutations of hydrophobic residues in the active-site of the enzyme (F81W, Y172F, and Y227F).

Omp7 mutant F81W provided several new products when incubated with FPP (**1**). We were able to detect the formation of sterpurene (**42**),^37^ pentalenene (**43**,)^38,39^ and 7β-protoilludanol (**44**)^40^ initially by GC analysis and spectroscopically proved it by NMR after isolation (Scheme 3A–C).

**Scheme 3:**
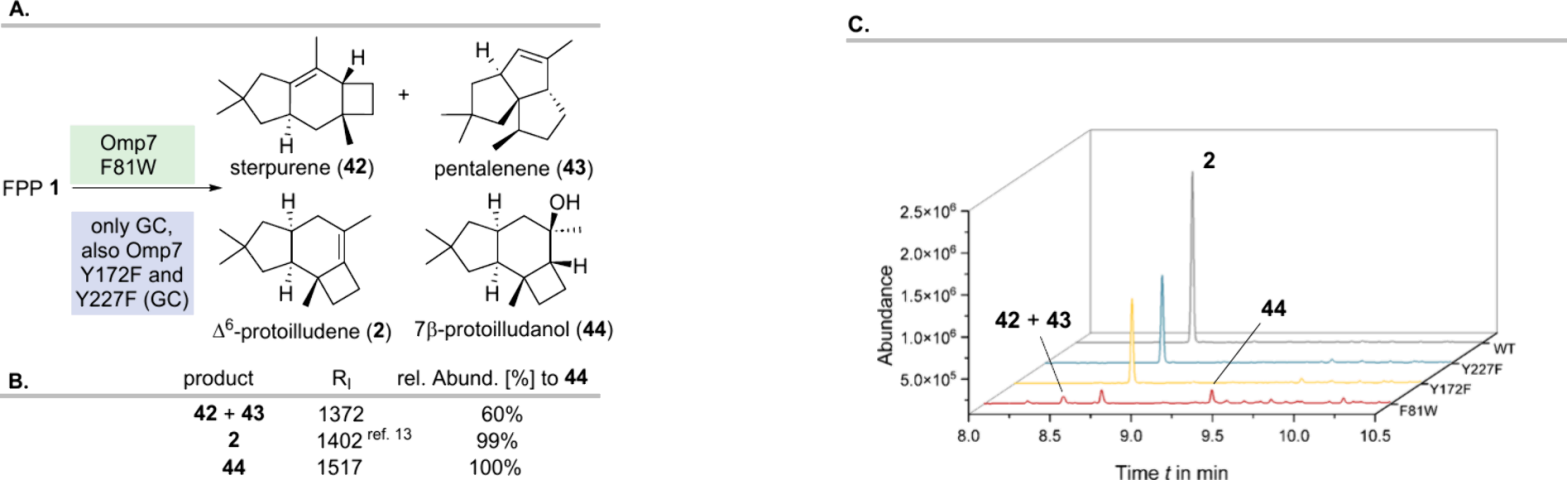
Formation of Δ6-protoilludene **2**, **42**, **43**, and **44** ^a^

a (A) Transformation of Omp7 mutants F81W, Y172F, and Y227F with **1** and formation of Δ^6^-protoilludene (**2**) and **42**–**44**. (B) Retention indices (*R*_I_) of sesquiterpenes using Omp7 F81W and FPP (**1**) with relative abundance to the main product **44**.^13^ (C) GC chromatograms of biotransformations with Omp7 variants (WT grey, F81W red, Y172F orange, Y227F blue) with FPP (**1**).

The choice of the GC column material was crucial in order to achieve the analytical separation of sesquiterpenes **42** and **43**. DB_5HT_ and ZB 1 columns were not suitable; however, an Optima WAX column allowed one to separate sterpurene (**42**) and pentalenene (**43**) for analytical GC measurements (for details see SI). The sterpurenes are a unique group of sesquiterpenes commonly produced by the fungus *Stereum purpureum*, while pentalenene (**43**) has been detected in *Streptomyces griseochromogenes*.^41^ The other two mutations, Y172F and Y227F, had no significant effect on the product spectrum compared to the wild type (wt). The selected Omp7 mutants all have the ability to convert substrates **15**–**17**. However, according to GC analysis, relative changes in the product ratios were only found for **15**, with no additional products found for those produced by wt Omp7. Substrates **16** and **17** generally showed similar product patterns compared to the wild type (see SI).

### Structure Elucidation

The structure elucidation of macrocyclic ketone **25** was straightforward with the help of the ^1^H–^13^C HSQC experiment, which allowed the assignment of the presence of one CH and one keto group as well as two alkenes. The isopropylidene side chain was identified by ^1^H–^1^H COSY and ^1^H–^13^C HMBC measurements. The ring alkene was determined to be (*Z*) as judged from the 1D ^1^H–^1^H NOE correlation between the methyl group (δ = 1.53 ppm) and the olefinic proton (δ = 5.07 ppm) (Figure 3). The NMR-driven structure determination of isomeric terpene alcohols **26a**,**b** revealed the presence of two olefinic double bonds. Here, the keto group was no longer present, suggesting that a second ring closure in which the keto group was involved must have taken place. ^1^H–^1^H-COSY and ^1^H–^13^C-HMBC analyzes for **26a** indicate the existence of two annulated six-membered rings, one of which bears a trisubstituted olefinic double bond. ^1^H–^1^H NOESY correlations and the multiplicity of one of the methylene groups at δ = 0.97 ppm revealed that both CH groups (δ = 1.87 ppm and δ = 1.71 ppm) and the tertiary alcohol at δ = 1.52 ppm are *syn*-oriented.

**Figure 3:**
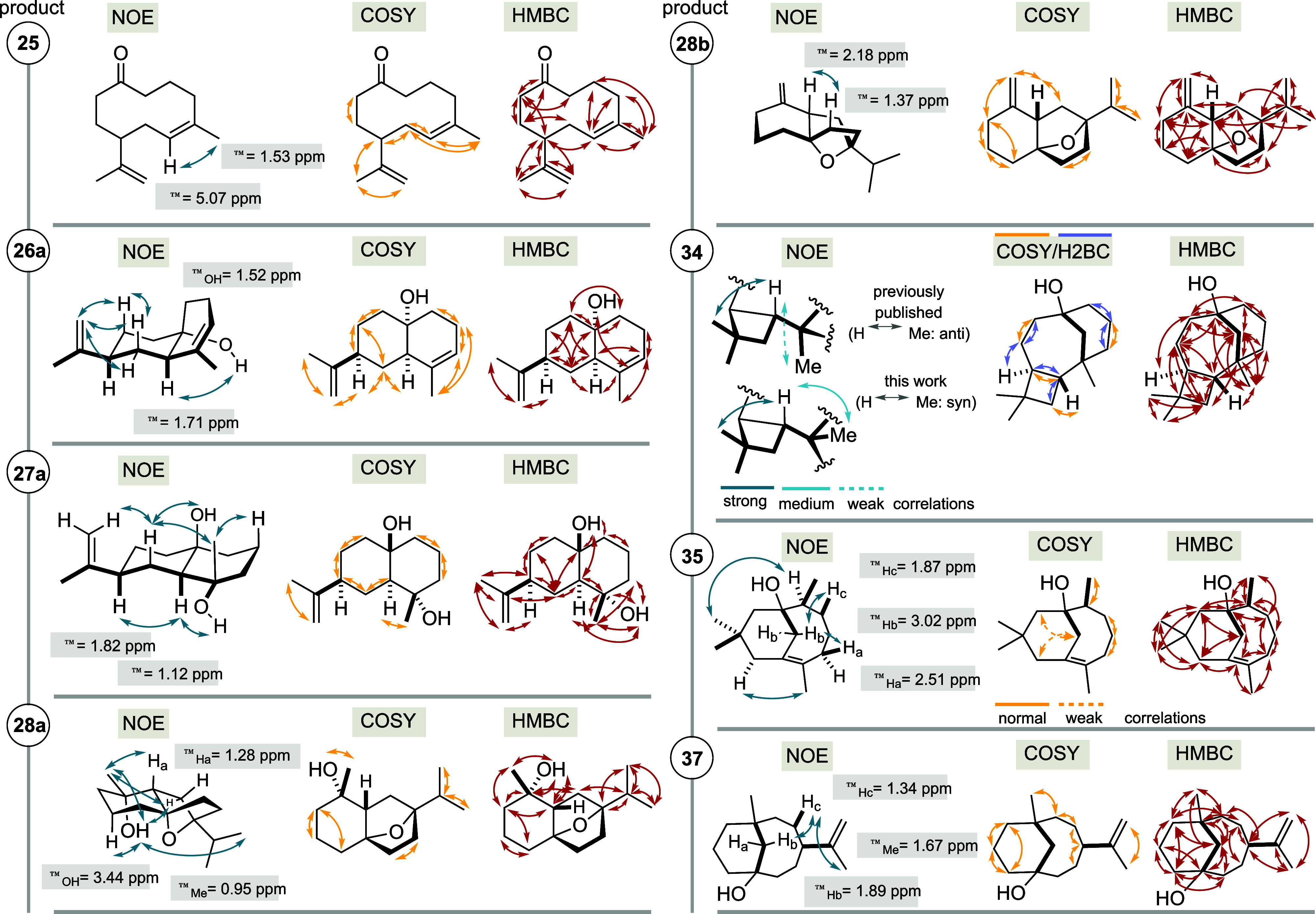
Key correlations in ^1^H–^1^H NOESY/1D-NOE (blue), ^1^H–^1^H COSY (orange), and ^1^H–^13^C HMBC (red) as analyzed during structure elucidation for selected products **25**, **26a**, **27a**, **28a**, **28b**, **34**, **35**, and **37** (details on the structure elucidation of these and other products are discussed in the text).

The structural elucidation of **27a** presented an additional challenge, as it is the only diol reported here, and it forms a network of hydrogen bonds with one water molecule. This leads to several ^1^H–^1^H-NOESY correlations between the alcoholic protons and different CH/CH_2_ groups. For elucidating the structure of **27a**, the relative stereochemistry could be determined by an ^1^H–^1^H NOESY NMR experiment. As we did not observe any correlations between the alcohol proton (δ = 1.19 ppm) and the vicinal CH group (δ = 1.73 ppm), we assume an *anti*-orientation. As the reported correlation^22^ between the isopropylidene group and the CH group (δ = 1.73 ppm) could not be found here, a 1,3-*syn*-orientation can be assumed so that **27a** resembles a diastereomer of the terpenoid reported by Dickschat and co-workers.^22^

Oxaterpenoid **28a** is characterized by the presence of three carbon atoms attached to the heteroatom oxygen, which is reflected in the ^13^C NMR spectrum by three signals at δ = 90.3, 85.2, and 70.0 ppm. ^1^H–^1^H-COSY and ^1^H–^13^C-HMBC analyses provided evidence for the presence of an ether bridge and a tertiary alcohol (δ = 70.0 ppm). ^1^H–^1^H-NOESY analysis revealed that the ether bridge and the alcohol are *syn*-oriented, while the vicinal CH group (δ = 1.28 ppm) is oriented on the same side as the methyl group (δ = 1.07 ppm).

The structural elucidation of **28b** is derived from that conducted for **28a**. The most notable difference is the *exo*-cyclic double bond, which reduces the number of stereocenters by one. The relative stereochemistry of the CH group (δ = 2.18 ppm) and the carbon bridge (δ = 1.37 ppm) could be deduced from a direct correlation in the ^1^H–^1^H NOESY spectrum.

The 1,3-diene moiety in **29** was unraveled by ^1^H–^1^H-COSY spectra that linked the four olefinic signals at δ = 4.85, 4.82, 5.25, and 5.90 ppm. Doublets in the ^1^H NMR spectrum for the methyl groups at δ = 0.80 and δ = 0.78 ppm indicate the presence of an isopropyl group. The proposed stereochemistry is based on mechanistic considerations with reference to the transformation of FPP promoted by STS Cop4. No correlation between δ = 5.25 and δ = 5.90 ppm was observed in the ^1^H–^1^H-NOESY spectrum. This is supported by a large ^3^ *J* coupling of 15.8 Hz as expected for olefinic protons in a *trans*-vicinal orientation.

^1^H and ^13^C NMR spectroscopic analysis of **30** revealed a characteristic proton at δ = 4.51 ppm attached to a carbon at δ = 94.3 ppm. This carbon atom has a neighboring carbon atom that resonances at δ = 152.7 ppm, which provides evidence for the proposed enol ether moiety. The analysis also unraveled another carbon atom attached to oxygen (δ = 75.9 ppm), which suggests the presence of a cyclic enol ether moiety. Obviously, major parts of the linear precursor **15** were not involved in the carbocationic cyclization cascade.

The constitution of **33** was found to be the bicyclic double bond isomer of literature known asteriscadiene (**45**) (Figure 4).^42^ In particular, the ^1^H and ^13^C NMR signals associated with the atoms of the cyclopentene subunit are almost identical for compounds **33** and **45**.

**Figure 4:**
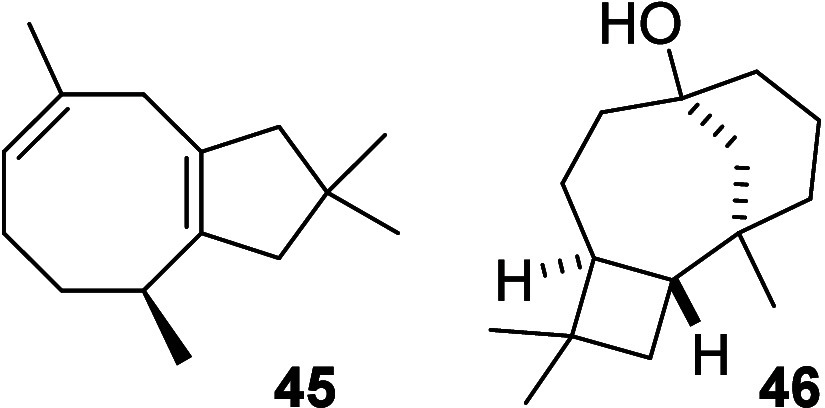
Structures of sesquiterpenes **45** and **46**.^35,42^ COSY correlations along the CH_3_–CH–CH_2_–CH_2_–CH_2_ element in **33** in combination with HMBC measurements on the geminal methyl groups and the exomethylene group provided further insights that led to our structural proposal.

We suggest that sesquiterpenoid **34** has the same constitution as a recently published sesquiterpene. However, in **34**, the methylene group is *syn*-orientated to the vicinal CH group as was supported from NOESY correlations of the vicinal Me groups compared to the diastereomer **46** that we reported before.^35^

Structure elucidation of **35** exerted some challenges due to the presence of four quaternary carbon atoms and a methylene moiety, which is surrounded by two of these quaternary centers. To gain a second set of NMR data, epoxide **36** was prepared as a single diastereomer. The ^1^H–^13^C-HMBC, ^1^H–^1^H-COSY, and ^1^H–^1^H-NOESY correlations for δ_Hb_ = 3.02 ppm and δ_Hb′_ = 1.60 ppm turned out to be diagnostic as these signals were also found in the corresponding NMR spectra of epoxide **36**. An ^1^H–^13^C ADEQUATE experiment of the epoxide confirmed that the methylene bridge is located between a tertiary alcohol and the oxirane. Structure elucidation of **37** first defined the NMR signals of the isopropylidene group and its neighboring methylene group (δ = 1.86 ppm). ^1^H–^1^H COSY and ^1^H–^13^C HMBC correlations indicate the presence of a cyclodecane core bridged by a methylene group (δ = 1.89 and 0.71 ppm). The ^13^C signal at δ = 72.0 ppm points to a tertiary alcohol. The suggested *anti*-orientation of the CH group (δ = 1.86 ppm) and the methylene bridge was based on the key ^1^H–^1^H NOESY correlation of one proton in the cyclodecane core at δ = 1.34 ppm with both the methylene bridge and the methyl group (δ = 1.67 ppm) of the isopropylidene group.

NMR analysis of product **38** revealed the presence of a cyclopropane ring that is part of the bicyclo[8.1.0]­undecane backbone. Especially, the ^1^H and ^13^C NMR chemical shifts δ of the cyclopropane protons and carbon atoms (δ = 0.50 ppm/δ = 29.6 ppm and δ = 1.31 ppm/δ = 26.4 ppm) and their proximity to the two geminal methyl groups as judged from the ^1^H–^13^C HMBC spectrum are diagnostic.

Cyclodeca-1,6-diene derivative **40** is the nor-derivative of the recently reported "germacrene F" (**14**).^16^ The ^1^H NMR spectrum reveals three alkenes with five overall olefinic protons. Two alkene moieties were found to be part of the macrocyclus while the third alkene unit is 1,1-disubstituted. ^1^H–^1^H-NOESY analysis provided information on the orientation of the CH group (δ = 2.08 ppm), and both olefinic protons in 1,3 position/γ-position (δ = 5.13 and 5.24 ppm) are orientated on the same side, while the allylic methyl group (δ = 1.51 ppm) and the olefinic proton in 1,4 distance (δ = 5.25 ppm) to the tertiary CH group (δ = 2.08 ppm) are located on the opposite side of the ring system (see Scheme 2C).

Structure elucidation of the cyclohexane derivate **41** relied on the ^1^H NMR signals of the monosubstituted alkene (δ = 6.10, 5.10, and 5.00 ppm) moiety along with those that relate to the disubstituted alkene of the side chain (δ = 5.59 and 5.50 ppm). These chains are located in vicinal positions at the cyclohexane ring, as was judged from the ^1^H–^1^H COSY and ^1^H–^13^C HMBC spectra. The relative stereochemistry was determined by ^1^H–^1^H NOESY correlations of the cyclohexane protons with either the methyl or the vinyl substituent. More detailed descriptions of the structural elucidations can be found in the Supporting Information.

### Mechanistic Considerations

The following section presents mechanistic considerations regarding the possible formation of the new terpenoids described here. Connections and similarities to the natural mechanisms involving FPP **1** are also discussed.

The formation of Δ^6^-protoilludene (**2**) by Omp7 is supposed to be initiated by (11 → 1)-cyclization, which is followed by a 1,2 hydride shift (Scheme 4). The resulting cationic intermediate **47** undergoes (2 → 9)-cyclization to cation **48**, and the cascade is terminated by (3 → 6)-cyclization, which provides the key intermediate protoilludane cation **49** followed by proton abstraction to yield Δ^6^-protoilludene (**2**) (Scheme 4, route a). This cationic sequence diverges from this known pathway at the stage of cation **49** when the mutant Omp7 F81W is employed. In fact, from there, three alternative routes can be postulated that can lead to the three sesquiterpenes **42**–**44**. 7β-Protoilludanol (**44**) is the result of a nucleophilic attack of water on cation **49** (route b). The protoilludane cation **49** has also been proven/proposed as a cationic intermediate in the formation of both sterpurene (**42**) and pentalenene (**43**) (Scheme 4; routes c and d). Tantillo et al. proposed two alternative mechanisms for the formation of pentalenene (**43**) from cation **49**.^43^

**Scheme 4:**
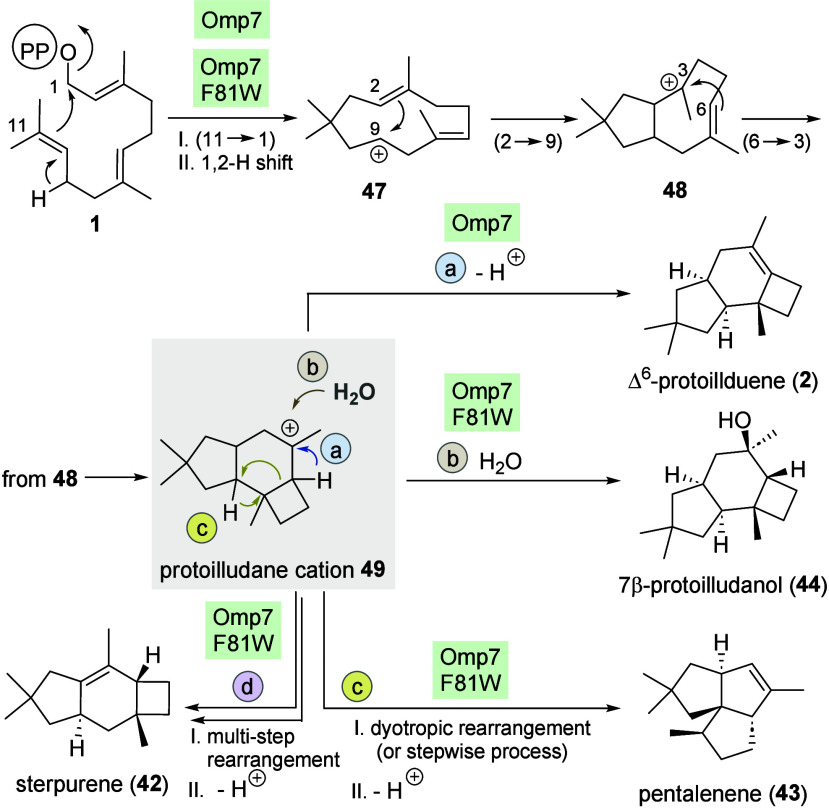
Mechanistic Considerations of the Formation of Omp7 and Mutant F81W with FPP (**1**)­^a^

a The formation of pentalenene (**43**) reveals the mechanistic link between terpenes **2** and **43**, as was identified in a related context before.^44^

The first route leads to a tricyclic system, which is reopened followed by a hydride shift and subsequent ring closure (not shown here), while the alternative and shorter route explains pentalenene formation directly from intermediate **49** via a dyotropic rearrangement.^43,44^ This type of rearrangement has rarely been studied in detail. Using quantum-chemical calculations on the enzyme-free reaction mechanism, the group of Tantillo was able to deduce that, in the course of the formation of pentalenene (**43**) by the sesquiterpene synthase PenA, such a dyotropic rearrangement, triggered by a cation acting through space, is very likely to occur.^43^ However, in later studies,^39b,45^ Tantillo and colleagues re-examined this case and concluded that a stepwise process is also feasible, so the exact mechanism remains a matter of debate to this day. Furthermore, it needs to be noted that Saeedi-Ghomi et al. investigated the formation of sterpurene (**42**) utilizing [1-^13^C] and [2-^13^C] acetate earlier and also proposed the protoilludane cation **49** as a mechanistic intermediate, followed by a multistep rearrangement.^46^

The keto group present in FPP derivative **15** provides additional opportunities for the cation cascade to proceed (Scheme 5A). The cascade commences with an initial (10 → 1)-cyclization to cation **51** after isomerization of keto-FPP derivative **15** to nerolidyl pyrophosphate derivative **50**. This initial step needs to be proposed in order to justify the formation of macrocyclic ketone **25** bearing a *Z*-configured olefinic double bond which results from proton abstraction (Scheme 5A, routes b and c) or a 1,2 hydride shift resulting in the tertiary cation **52** (Scheme 5A, route a). In the case of **52**, the keto group now serves as a nucleophile (O → 10), initiating a (2 → 7)-cyclization which results in the formation of the shared cation **53**. Subsequent nucleophilic attack of water results in the formation of oxaterpenoid **28a** (Scheme 5A, route a_1_), while proton abstraction leads to **28b** (Scheme 5A, route a_2_). Alternatively, the keto group can serve as an electrophile in a (2 → 7)-cyclization, commencing from ketone **25**. Depending on the orientation of the keto group, the ring closure yields two different diastereomeric cations **54** (Scheme 5A, route b) and **55** (Scheme 5A, route c), respectively, both of which contain a 1,1-disubstituted olefinic double bond and the tertiary alcohol. Cation **54** in which the alcohol is *syn* orientated to the vicinal CH group can undergo proton abstraction to form the isomeric terpene alcohols **26a** and **26b** (Scheme 5A, routes b_1_ and b_2_). For cation **55** in which the alcohol and the vicinal CH group are *anti* orientated, either nucleophilic attack of water yields terpene alcohol **27a** (Scheme 5A, route c_1_) or proton abstraction provides terpenoid **27b** (Scheme 5A, route c_2_).

**Scheme 5:**
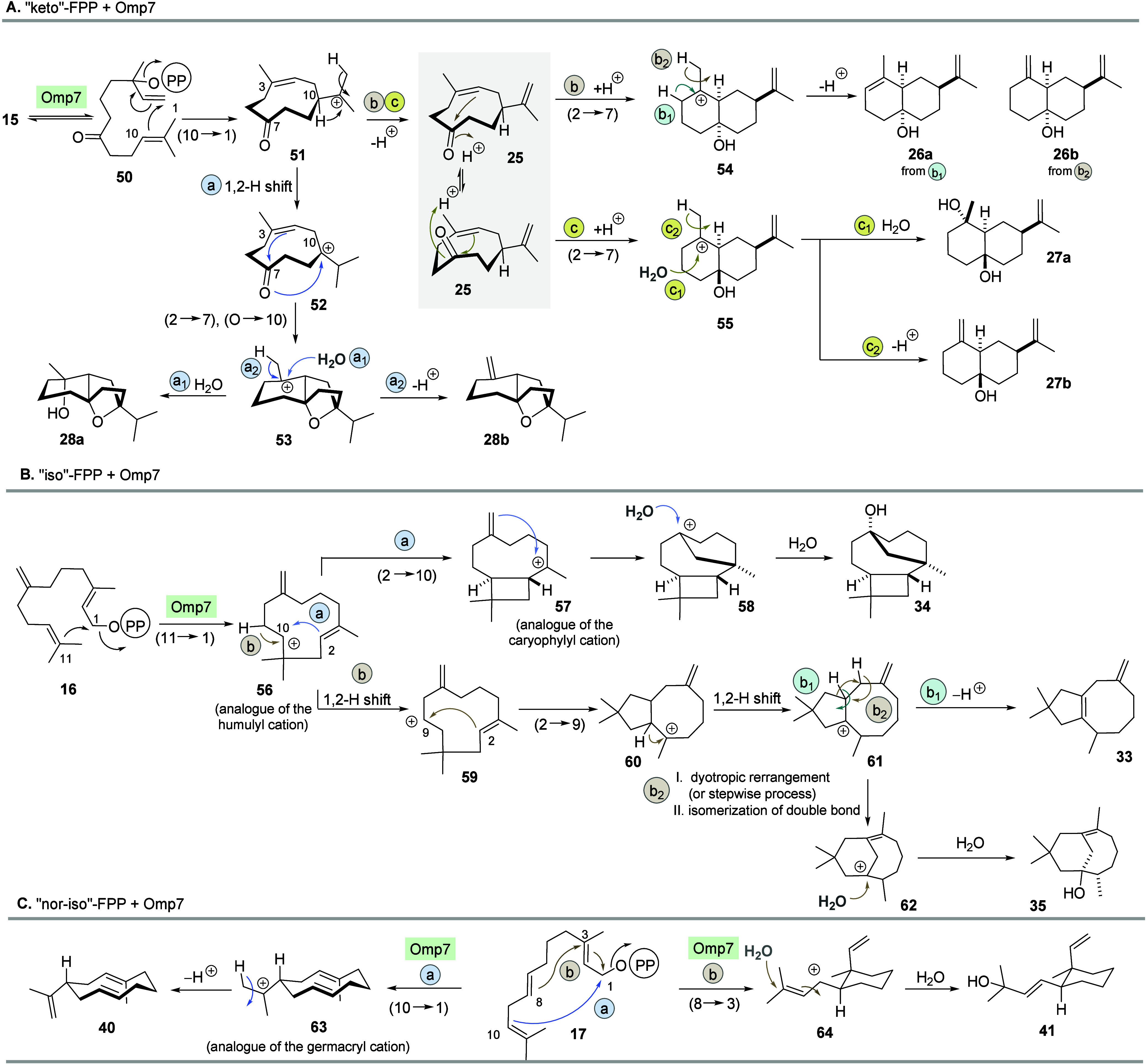
Mechanistic Considerations for the Transformation of (A) “keto”-FPP **15**, (B) “iso”-FPP **16**, and (C) “nor-iso”-FPP **17** by Omp7

In contrast to **15**, we propose an initial (11 → 1)-cyclization for “*iso*”-FPP **16** with Omp7 (Scheme 5B). Intermediate cation **56** that resembles a derivative of the humulyl cation undergoes a second (2 → 10)-cyclization to the corresponding caryophyllyl cation **57** (Scheme 5B, route a). From there, the 1,1-disubstituted alkene gets involved in a third cyclization which provides cation **58**, and the cationic cascade is terminated by trapping of water and the formation of sesquiterpenoid **34**. Cation **56** also serves as the starting point for an alternative mechanistic route which is initiated by a 1,2 hydride shift (Scheme 5B, route b). As a result, cation **59** is formed which undergoes a (2 → 9)-cyclization to **60**. A second 1,2 hydride-shift provides cation **61** from the separation of two pathways (Scheme 5B, routes b_1_ and b_2_). Deprotonation leads to diene **33**, while the second pathway, starting from intermediate **61**, which provides terpenoid **35**, is tricky to assess with regard to the possible mechanism. We postulate that a dyotropic rearrangement takes place first, followed by the isomerization of the 1,1-disubstituted olefinic double bond to the tetrasubstituted alkene, resulting in cation **62**. This is intercepted by water, forming alcohol **35**. Transformation of “nor-iso”-FPP analogue **17** by Omp7 is initiated by (10 → 1)-cyclization (Scheme 5C), and the resulting macrocyclic cation **63** gets deprotonated toward nor-germacrene F (**40**) (route a). The second product **41**, a cyclohexane bearing nor-sesquiterpene, may result from a rare initial (8 → 3) cyclization which provides cation **64** which is intercepted by water (route b).

Finally, the sesquiterpene synthases JeSTS4 and Pts also accept “iso”-FPP **16** to yield two other products, namely, sesquiterpenes **37** and **38** (Scheme 6A). After (10 → 1) cyclization, cation **65** is formed, and at this point, the mechanism splits into two alternative pathways depending on the STS employed. With Pts, deprotonation leads to germacrene A derivative **66** (Scheme 6A, route a). A second, proton-induced cyclization yields bicyclic cation **67** which is intercepted by water to yield alcohol **37**. Alternatively, with STS JeSTS4, intermediate **65** is deprotonated in the β-position with respect to the carbocation to establish the cyclopropane ring present in sesquiterpene **38** (Scheme 6A, route b). This pathway is also operative when JeSTS4 is exposed to the “keto”-FPP derivative **15**, as cyclopropane **32** is formed accordingly (Scheme 6B). Biotransformation of **15** with Cop4 results in the macrocyclic ketone **29**. After initial isomerization of the pyrophosphate moiety toward **50** and (10 → 1) cyclization yielding cation **69**, a 1,3-hydride shift and deprotonation via **70** lead to the formation of the 1,3-diene observed in **29** (Scheme 6C).

**Scheme 6:**
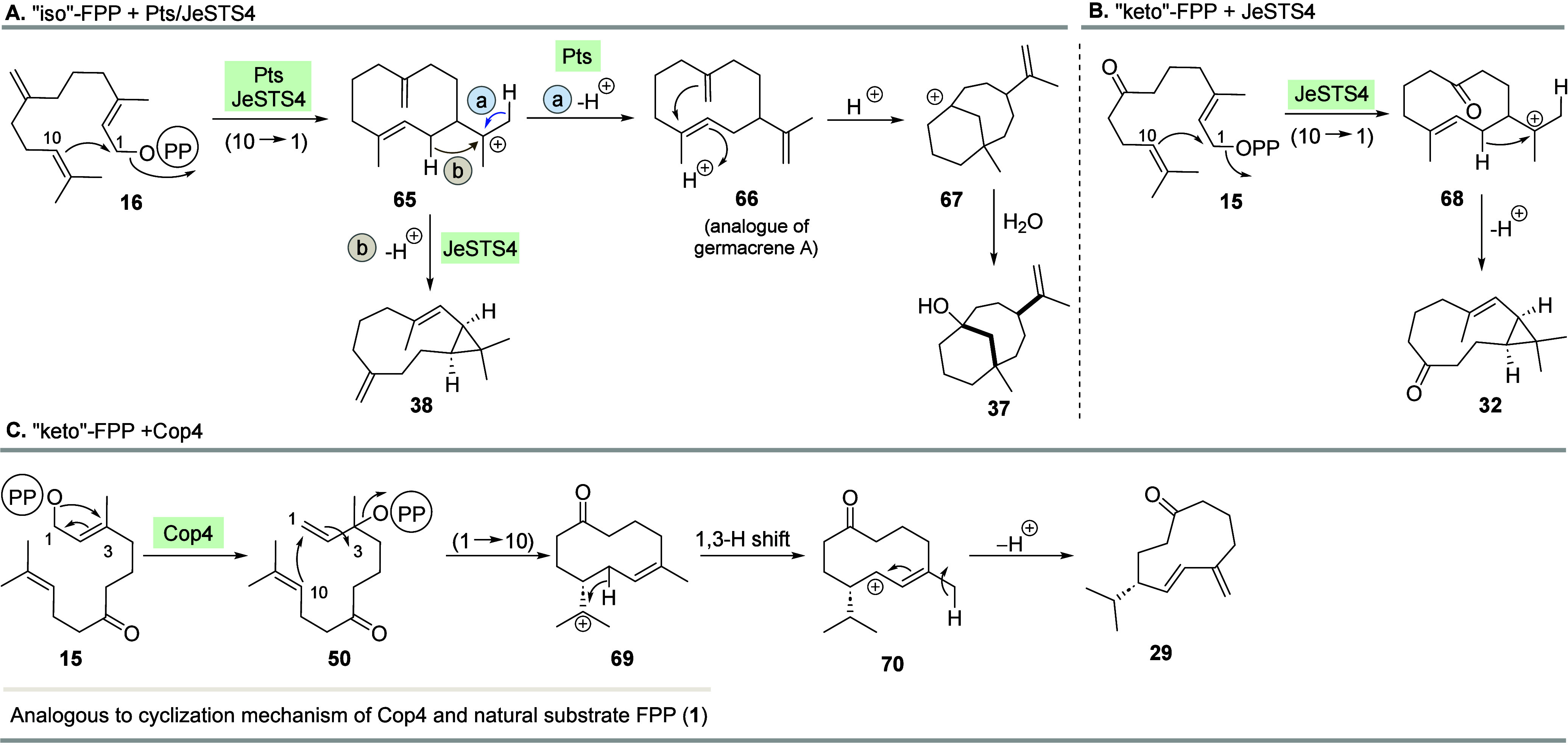
Mechanistic Considerations for the Transformation of (A) “iso”-FPP **16** by Pts and JeSTS4, (B) “keto”-FPP **15** by JeSTS4, and (C) “keto”-FPP **15** by Cop4

An interesting aspect of the results presented here concerns the fact that FPP (**1**) and “iso”-FPP **16** are converted by STS Tps32 to very structurally different products. In the first case, as already reported, vetispiradiene (**19**) is formed (Scheme 7).^29^ In the case of FPP derivative **16**, however, valencene (**39**) is the main product. A closer inspection of the possible mechanisms that could lead to these two sesquiterpenes suggests that, in both cases, (10 → 1) followed by (2 → 7) cyclization should lead to an intermediate that matches the eudesmane cation **72a**. However, the subsequent cascade then yields product **19** via cation **74** and sesquiterpene **39** via an alternative pathway and intermediate cation **75**.

**Scheme 7:**
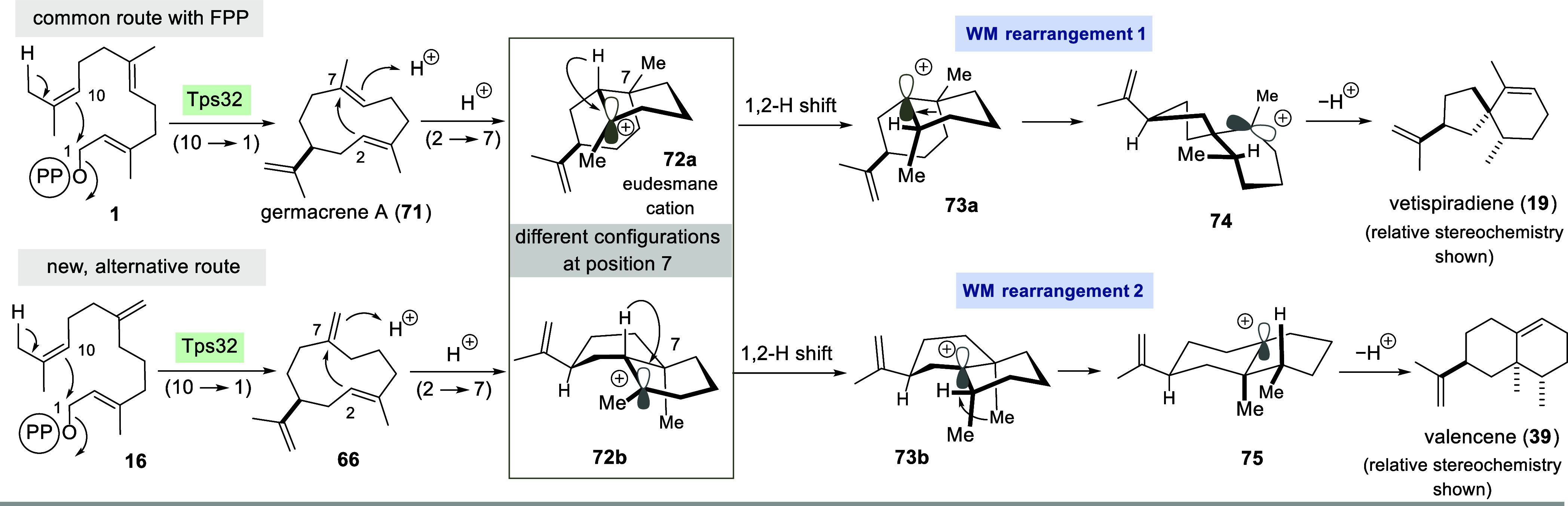
Mechanistic Considerations on the Ttransformation of Tps32 with FPP **1** and “iso”-FPP 16 to Vetispiradiene **19** and Valencene **39** ^a^

a Mechanistic considerations for the transformation of Tps32 with FPP (**1**) and “iso”-FPP **16** to vetispiradiene (**19**)^48^ and valencene (**39**), respectively. The routes presumably diverge at the second (2→7) cyclization step leading to the eudesmane cation **72a** (*syn*-annulated decahydronaphthalene) and its 7-diastereomer **72b** (*anti*-annulated decahydronaphthalene). These different scenarios induce two different Wagner-Meerwein rearrangements (1 with ring contraction and 2 with methyl shift).^48^

From a mechanistic point of view, this observation is most likely linked to the second (2 → 7) cyclization around the carbon atom at position 7. In fact, the structural differences between the substrates manifest themselves precisely at C7 and thus can lead to opposite facial attacks, resulting in the two diastereomeric cations **72a** and **72b** (*epi*-eudesmane cation). This in turn leads to two different periplanar scenarios with respect to the empty p orbital in diastereomeric cations **73a** and **73b** formed after the respective 1,2-hydride shifts. These differences in σ-bond orientation favor different WM rearrangements leading to the cations **74** and **75**, respectively.^47^

## Conclusions

In summary, we show that structural modifications around the central double bond of FPP (**1**) provide substrates that are accepted by several STSs, including promiscuous Omp7. In fact, among the STSs selected here, Omp7 stands out in terms of the diversity of new terpenoids formed.

The three new STSs JesTS4, LphTPS, and PaTPS are in principle also suitable for the expansion of the “terpenome” using unnatural substrates, but in our hands, the expression and isolation protocols employed gave only low yields of active protein. In addition, the biotransformations did not provide the same spectrum of structurally diversified new products as that of Omp7 and BcBOT2. Another notable observation is the fact that the keto group at C7 can serve as an electrophile, enabling C–C linkages that lead directly to tertiary terpene alcohols as shown in examples **26** and **27**. Furthermore, the keto group can also serve as a nucleophile in the cation cascade for which bridged ethers **28** are telling examples. Interestingly, these two products structurally closely resemble known terpenes, specifically the corvol ether sesquiterpenes A (**76**) and B (**77**), which are known to be products of a sesquiterpene synthase from the actinomycete *Kitasatospora seta* (Figure 5).^49^ Overall, these findings demonstrate that the chemoenzymatic approach presented here facilitates rapid penetration into the chemical space of the rather unexplored corners of the world of terpenes.

**Figure 5:**
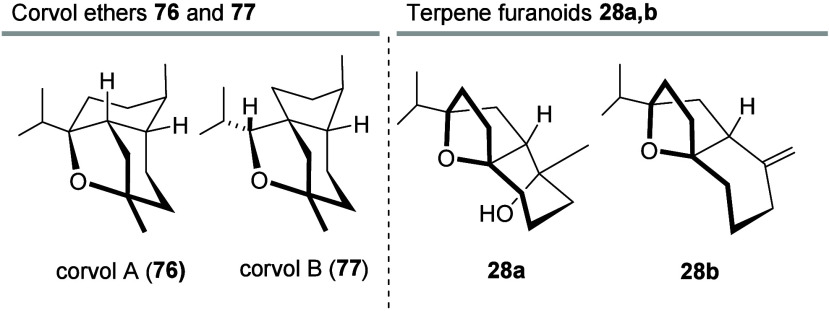
Structures of the corvol ethers **76** and **77** (left) and spatial 3D relationship to terpene furanones **28a**,**b** (right).^22,49^

Furthermore, site-specific mutagenesis of Omp7, in particular, F81W, led to the formation of three new products, all of which can be traced back to the protoilludane cation **49**. In conjunction with our proposed mechanism for **35**, this strengthens the hypothesis that another example of a dyotropic rearrangement may be found here.

Special attention should be paid to cyclodecanone derivative **29**. In the past, it served as the common late stage intermediate in several total syntheses^50^ directed toward the sex pheromone (−)-periplanone B (**78**).^51^ This oxygenated sesquiterpenoid and the reduced derivatives **79** and **80** are excreted by the female American cockroach *Periplaneta americana* (Scheme 8). In fact, our chemoenzymatic approach to this cyclodecanone demonstrates the potential of using terpene synthases in combination with synthetic chemistry to access advanced intermediates in natural product synthesis. It paves the way for more sophisticated chemosynthetic approaches in the field of terpene synthesis.

**Scheme 8:**
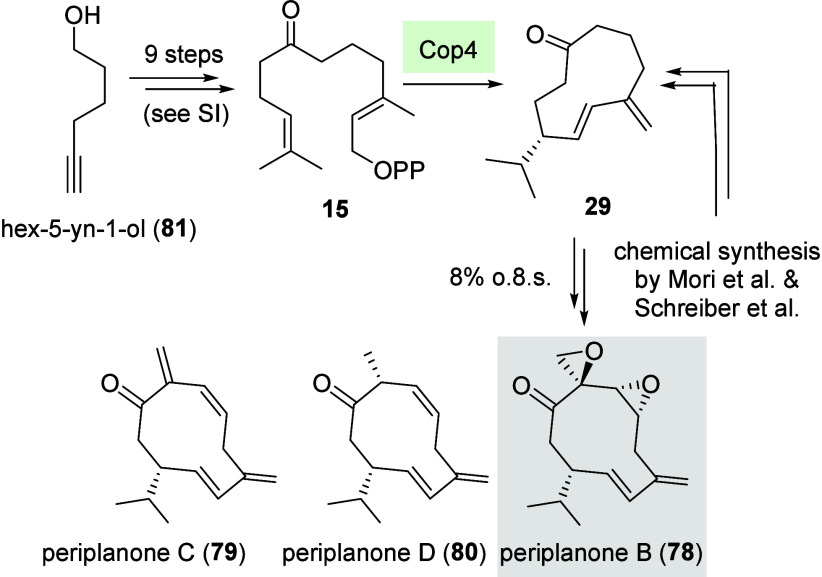
Formal Chemoenzymatic Synthesis of the Sex Pheromone Periplanone B (**78**)

## Supplementary Material



## Notes

Experimental procedures, characterizations, and analytical data of new compounds, X-ray diffraction data for **32**, and NMR spectra (PDF)
