# Supplementary material for: Chemoenzymatic Synthesis of Structurally Diverse Terpenoids from Farnesyl Pyrophosphates Modified at the Central Alkene Unit
Source: J Am Chem Soc. 2025 Dec 22;148(1):525–36. doi: 10.1021/jacs.5c14636 (PMC12814329; doi:10.1021/jacs.5c14636)
Supplement: Supplementary file 1 [file ja5c14636_si_001.pdf]

Chemoenzymatic Synthesis of Structurally Diverse Terpenoids from Farnesyl Pyrophosphates Modified at the Central Alkene Unit

*Henry Struwe[a], Christopher Slotman[a], Laurent Höft[a], Gerald Dräger[a], Jörn Droste[a], Jörg Fohrer[b], Katharina Hausmann[c], Sascha Beutel[c], Dominik Kolling[d], Jesko Köhnke[d] and Andreas Kirschning[a,e]**

[a] Institute of Organic Chemistry, Leibniz University Hannover, Schneiderberg 1B, 30167 Hannover, Germany
[b] Department of Chemistry, Technical University Darmstadt, Alarich-Weiss-Straße 4, 64287 Darmstadt, Germany
[c] Institute of Technical Chemistry, Leibniz University Hannover, Callinstraße 5, 30167 Hannover, Germany
[d] Institute of Food Chemistry, Leibniz University Hannover, Callinstraße 5, 30167 Hannover, Germany
[e] Uppsala Biomedical Center (BMC), Uppsala University, Husargatan 3, 752 37 Uppsala, Sweden

Email: andreas.kirschning@oci.uni-hannover.de

Table of Contents

1. **Materials and methods**
1.1 General information
1.2 Synthesis
1.2.1 Synthetic Routes
1.2.2 Chemical Synthesis
1.2.3 Experimental Procedures of Biotransformation
1.3 Microbiological Methods and Biotransformation
1.3.1 Procedure A
1.3.2 Procedure B
1.3.3 Procedure for point mutations
1.4 GC-MS Data
1.4.1 Positive and Negative controls
1.4.2 Biotransformation of GCoA and Keto-FPP
1.4.3 Biotransformation of Cop4 and Keto-FPP
1.4.4 Biotransformation of Omp7 and Keto-FPP
1.4.5 Biotransformation of Omp7 and *iso*-FPP
1.4.6 Biotransformation of JeSTS4 and Keto-FPP
1.4.7 Biotransformation of JeSTS4 and *iso*-FPP
1.4.8 Biotransformation of Pts and *iso*-FPP
1.4.9 Biotransformation of Tps32 and *iso*-FPP
1.4.10 Biotransformation of Omp7 mutants and different substrates
1.4.11 Biotransformation of Omp7 F81W mutant and FPP
1.4.12 Biotransformation of Omp7 and Nor-*iso*-FPP
1.4.13 Retention indices of isolated products
1.5 Structure elucidations
1.5.1 Structure elucidation of **28b**
1.5.2 Structure elucidation of **25**
1.5.3 Structure elucidation of **28a**
1.5.4 Structure elucidation of **26b**

1.5.5 Structure elucidation of **26a**
1.5.6 Structure elucidation of **27b**
1.5.7 Structure elucidation of **27a**
1.5.8 Structure elucidation of **29**
1.5.9 Structure elucidation of **30**
1.5.10 Structure elucidation of **33**
1.5.11 Structure elucidation of **34**
1.5.12 Structure elucidation of **37**
1.5.13 Structure elucidation of **38**
1.5.14 Structure elucidation of **32**
1.5.15 Structure elucidation of **44**
1.5.16 Structure elucidation of **42** in the presence of **43**
1.5.17 Structure elucidation of **35**
1.5.18 Structure elucidation of **36**
1.5.19 Structure elucidation of **40**
1.5.20 Structure elucidation of **41**
1.6 Presentation of AlphaFold model of Omp7
1.7 X-Ray Data

2. Copies of NMR Spectra
3. References (supporting information)

1. Materials and methods

1.1 General information

The experimental procedures described below, in which water is neither a reactant nor used as a solvent, were carried out in pre-dried flasks under an inert gas atmosphere. To ensure homogeneity, the reaction mixtures were stirred with a magnetic stirrer. The temperatures indicated refer to the bath vessels used. Temperatures of −78 °C were achieved by an acetone-dry ice mixture, −41 °C can be obtained by MeCN dry ice mixtures and 0 °C by a mixture of water and ice. The room temperature does not refer to a specific value, but varies with the ambient temperature. For varying temperatures water baths, oil baths, NaCl-ice mixtures or a cryostat were used.

Unless stated otherwise, dry solvents were used for each reaction in which a pre-dried flask was used. Tetrahydrofuran, dichloromethane, *N,N*-dimethylformamide, acetonitrile, and diethyl ether were obtained as dry solvents from a “Braun” solvent purification system or as dry solvents from “Acros Organics” or “thermos scientific”. Tetrahydrofuran was also used when being freshly distilled. Deuterated solvents were obtained from Deutero, ZeoTope or Sigma Aldrich.

Column chromatography was performed using silica gel obtained from Macherey-Nagel (particle size 40-63 µm). During chromatography pressure was applied and the used solvents/mixtures are listed in the corresponding experimental procedure. To perform TLC analysis pre-coated TLC sheets ALUGRAM® Xtra SIL G/UV_{254} foil from Macherey-Nagel (layer: 0.20 mm silica gel 60 with fluorescent indicator UV_{254}) were used. As stain solutions reagents containing vaniline or $KMnO_4$ were used. In selected cases UV light (λ = 254 nm/ 366 nm) was employed to identify TLC spots. The R_f values given are subject to certain inaccuracies due to measurement limitations, but can be used for initial orientation. Volumes given as a sum refer to the amount of solvent used for the first solution and the additional solvent used for rinsing the glassware (e.g. solvent (2 mL + 2 mL)).

NMR data (^{1}H, ^{13}C, ^{31}P, DEPT135, 2D-NMR) were recorded on the following spectrometers: Bruker AVANCE I (ν_L(^{1}H)= 400 MHz) equipped with a DUL probe, Bruker AVANCE III HD (ν_L(^{1}H)=400 MHz) equipped with either a PRODIGY BBFO or BBO probe, Bruker AVANCE III HD (ν_L(^{1}H)=500 MHz) equipped with a TCI cryoprobe, and Bruker AVANCE NEO (ν_L(^{1}H)=600 MHz) equipped with DUL cryoprobe. All probes are equipped with z-Gradient coils. The deuterated solvents are given in the respective procedures ($CDCl_3$, C_6D_6, D_2O). The analysis was performed using the Bruker Topspin® software. The residual solvent signal of the deuterated solvents was used to calibrate the chemical shift scale of the NMR spectra. Chemical shifts δ are given in ppm, J coupling constants are given in Hz and were determined manually or with appropriate software functions. The abbreviations used for multiplicities are s (singlet), d (doublet), t (triplet), q (quartet), qi (quintet), and m (multiplet). When a particular signal cannot be unambiguously assigned to a particular position, e.g. because signals overlap, the indices in question are separated by "/" to indicate that there are several possibilities. Structural assignments were made with additional information from HSQC, COSY, HMBC and 1D-NOE/NOESY experiments

HR-ESI-MS (Tof) analysis was performed with Aliance 2695 HPLC (Waters) coupled to a LCT premier (Waters) with a lock spray dual ion ESI source. GC-EI-MS (quadrupole) was measured on a 6890 GC (Agilent) / 5973 MSD (Hewlett Packard) (column: Optima WAX) and a 7890B GC (Agilent) / 5977B MSD (Agilent) (column: Optima5HT). HR-CI-MS (Tof) was performed with a 6890 GC + (Hewlett Packard) coupled to GCT Premier (Waters) equipped with a CI ion source. The GC systems are additionally equipped with a FI detector. Protein HRMS measurements of our Omp7 variants were performed using HR UPLC-ESI-MS (QTof Premier, Waters). The multiple charged ESI mass spectra were deconvoluted using MaxEnt1 as part of MassLynx MS software (Waters).

Compound isolation on a preparative GC was performed with GC system HP6890 Series Plus coupled to a HP7683 Series Injector and a Gerstel PFC with cooling traps at −3 °C (Thermos Scientific A40). The stationary phase is a Zebron ZB1: 30 m, 0.53 mm, 3.00 µm. Temperature program for **27a**: Starting temp. 100 °C; 45 °C/min for 3.44 min to 255 °C; 1 °C/min for 10 min to 265 °C holding for 5 min, 40 °C/min for 1 min to 280 °C. Temperature program for **42 - 44**: Starting temp. 100 °C; 30 °C/min for 3.33 min to 200 °C; 3 °C/min for 13.3 min to 240 °C holding for 2 min, 40 °C/min for 1 min to 280 °C. Temperature program for **30**: Starting temp. 100 °C; 20 °C/min for 5.00 min to 200 °C holding for 1 min; 5 °C/min for 9.00 min to 245 °C holding for 3 min, 20 °C/min for 2 min to 285 °C. **41**: Starting temp. 100 °C; 40 °C/min for 2.50 min to 200 °; 1.5 °C/min for 8.00 min to 212 °C, 40 °C/min for 1.70 min to 280 °C.

Ion exchange of tetra-*n*-butylammonium to ammonium cations was performed using DOWEX®50WX8 resin as column material. For this purpose, the resin was rinsed with an aqueous HCl solution (3 M) until the eluent was acidic (pH paper). It was then rinsed with H_2O to a near neutral pH, with NH_3 (6%) to an alkaline pH, and finally with ion exchange buffer (980 mL H_2O, 20 mL *i*-PrOH, 2 g NH_4HCO_3) to a pH of 8 to 9. The residues collected from the reactions were then uploaded onto the column and eluted with ion exchange buffer. The product-containing fractions, analyzed by $KMnO_4$-TLC staining, were combined and the solvent was removed in vacuo. The residue was diluted in an aqueous NH_4HCO_3 solution (0.05 M) and freeze-dried. To remove the inorganic ammonium pyrophosphate salt, the product was dissolved in an aqueous NH_4HCO_3 solution (0.05 M, 2 mL) and mixed with MeCN/*i*-PrOH (1/1, 8 mL). After centrifugation (5000 rpm, 10 min, 4 °C), the liquid was collected and the procedure was repeated. After removal of the solvent in vacuo, the residue was dissolved in an aqueous NH_4HCO_3 solution (0.05 M) and freeze-dried. The product was stored at temperatures between −20 °C and −80 °C. The diphosphate salts were analyzed by ^{1}H-, ^{13}C- and by ^{31}P-NMR spectroscopy. HRMS measurements and determination of R_f values could sometimes not be performed on these salts.

Experiments with living microorganisms were performed in either S1 or S2 laboratories. Sterile work was performed using a Thermos Scientific laminar flow cabinet (type 2020). Optical density (OD600) was measured at 600 nm using a photometer from FoodALYT. Cell lysis was performed using a SONOPULS ultrasonic homogenizer from Bandelin (Procedure A) and CF1 cell disruptor by Constant Systems Ltd. (Procedure B). IMAC was performed with column material from Macherey-Nagel GmbH & Co. KG containing Ni ions, such as Ni-NTA or Ni-

IDA (Procedure A) or using Protino® Ni-NTA Agarose from Macherey-Nagel GmbH & Co. KG on either ÄKTA Go™ or ÄKTA pure™ (Procedure B). Buffer exchange was performed using a column from GE Healthcare AB (V= 8.3 mL) (Procedure A) or Sephadex G-25 resin by Cytiva (Procedure B). A Merck KGaA filter unit with an exclusion limit of 30000 Da was used to constrict protein solutions (Procedure A only). The extinction coefficient for protein concentration measurement was determined using ProtParam (ExPASy). Concentration measurements of enzyme mutants were performed with wild type extinction coefficient. UV/Vis spectroscopy was performed at 280 nm using a DeNovix spectrophotometer (type: DS-11+). GC-MS analyses for in vitro biotransformation were performed using an Agilent 5977B GC/MSD with 7890B GC system and an Optima 5HT - 0.25 µm, carrier gas: He, column volume: 30 m x 250 µm x 0.25 µm, injection volume 1 µL. Retention indices (RI) were determined in comparison to *n*-alkanes (C7 to C30). Differences in measurement dates of a sample and *n*-alkanes are within a short time period but necessarily performed on the same day.

Composition of buffer solutions und culture media

In order to set pH value for the buffer solutions, aqueous NaOH and HCl solutions were used.

Lysis buffer:	Tris·HCl (40 mM), NaCl (100 mM), pH = 8
ÄKTA lysis buffer:	Tris·HCl (40 mM), NaCl (100 mM), imidazole (20 mM), pH = 8
Ni-NTA buffer (x M):	Tris·HCl (40 mM), NaCl (100 mM), Imidazol (x M)
HEPES buffer:	HEPES (50 mM), DTT (5 mM), pH =7.5
LB media:	0.50% (w/v) yeast extract (Duchefa Biochemie)
	1.00% (w/v) trypton (Duchefa Biochemie)
	0.50% (w/v) NaCl (Roth or VWR)
2-TY media:	1.00% (w/v) yeast extract (Duchefa Biochemie)
	1.60% (w/v) tryptone (Duchefa Biochemie)
	0.50% (w/v) NaCl (Roth or VWR)
SOB media:	0.50% (w/v) yeast extract (Duchefa Biochemie)
	2.00% (w/v) tryptone (Duchefa Biochemie)
	0.06% (w/v) sodium chloride (Roth or VWR)
	0.02% (w/v) potassium chloride (Roth)
SOC media:	93.8 mL SOB media
	1.25 mL magnesium chloride hexahydrate (2M) (Roth)
	5 mL D-(+)-glucose monohydrate (20%) (Roth)

Cloning of STSs (JeSTS4, PaTPS, LphTPS) was carried out in *E. coli TOP10* with pET28TEV plasmids, leading to a N-terminal polyhistidine tag for all enzymes through expression. Details

for the use of Cop4, GCoA, Omp7 and Tps32 can be seen in our previous published work.[S1, S2] Experimental details on Omp7 mutants are given below. Enzyme expression was performed in *E.coli* BL21 (DE3) for all Enzymes except for Omp7 Y22F mutant, which was expressed in *E.coli* Rosetta (DE3).

Information and characterization of Cop4

The gene for Cop4 used in this work was synthesized by GENEWIZ LLC as a construct with pUC57 and are optimized for protein expression in *E.coli*. The gene sequence can be used from a gene bank for the accession number A8NU13.[S3]

Information and characterization of GCoA

The gene for GCoA used in this work was synthesized by GENEWIZ LLC as a construct with pUC57 and are optimized for protein expression in *E.coli*. The gene sequence can be used from a gene bank for the accession number B1W019.[S4]

Information and characterization of Omp7

The gene for Omp7 used in this work was synthesized by GENEWIZ LLC as a construct with pUC57 and are optimized for protein expression in *E.coli*. The gene sequence can be used from a gene bank for the accession number MUStwsD_GLEAN_10000831.[S5]

Information and characterization of JeSTS4

The gene for JeSTS4 used in this work was synthesized by Twist Bioscience as a construct with pet28a and are optimized for protein expression in *E.coli*. The gene sequence can be used from a gene bank for the accession number UKS51568.1. [S6]

Information and characterization of PaTPS

The gene for PaTPS used in this work was synthesized by Twist Bioscience as a construct with pet28a and are optimized for protein expression in *E.coli*. The gene sequence can be used from a gene bank for the accession number Q675L0.1. [S7]

Information and characterization of LphTPS

The gene for LphTPS used in this work was synthesized by Twist Bioscience as a construct with pet28TEV and are optimized for protein expression in *E.coli*. The gene sequence can be used from a gene bank for the accession number ASV63464.1. [S8]

Information and characterization of Pts

The gene for Pts used in this work was synthesized by Twist Bioscience as a construct with pET16b and are optimized for protein expression in *E.coli*. The gene sequence can be used from a gene bank for the accession number Q49SP3. [S9]

Information and characterization of Tps32

The gene for GCoA used in this work was synthesized by GENEWIZ LLC as a construct with pUC57 and are optimized for protein expression in *E.coli*. The gene sequence can be used from a gene bank for the accession number AEP82773.[S10]

1.2 Synthesis

1.2.1 Synthetic Routes

Since we already published the synthesis of "*iso*"-FPP [S11] and "Nor-*iso*"-FPP [S12] their synthetic schemes are displayed here again. For details and experimental procedures, the reader is referred to the references.

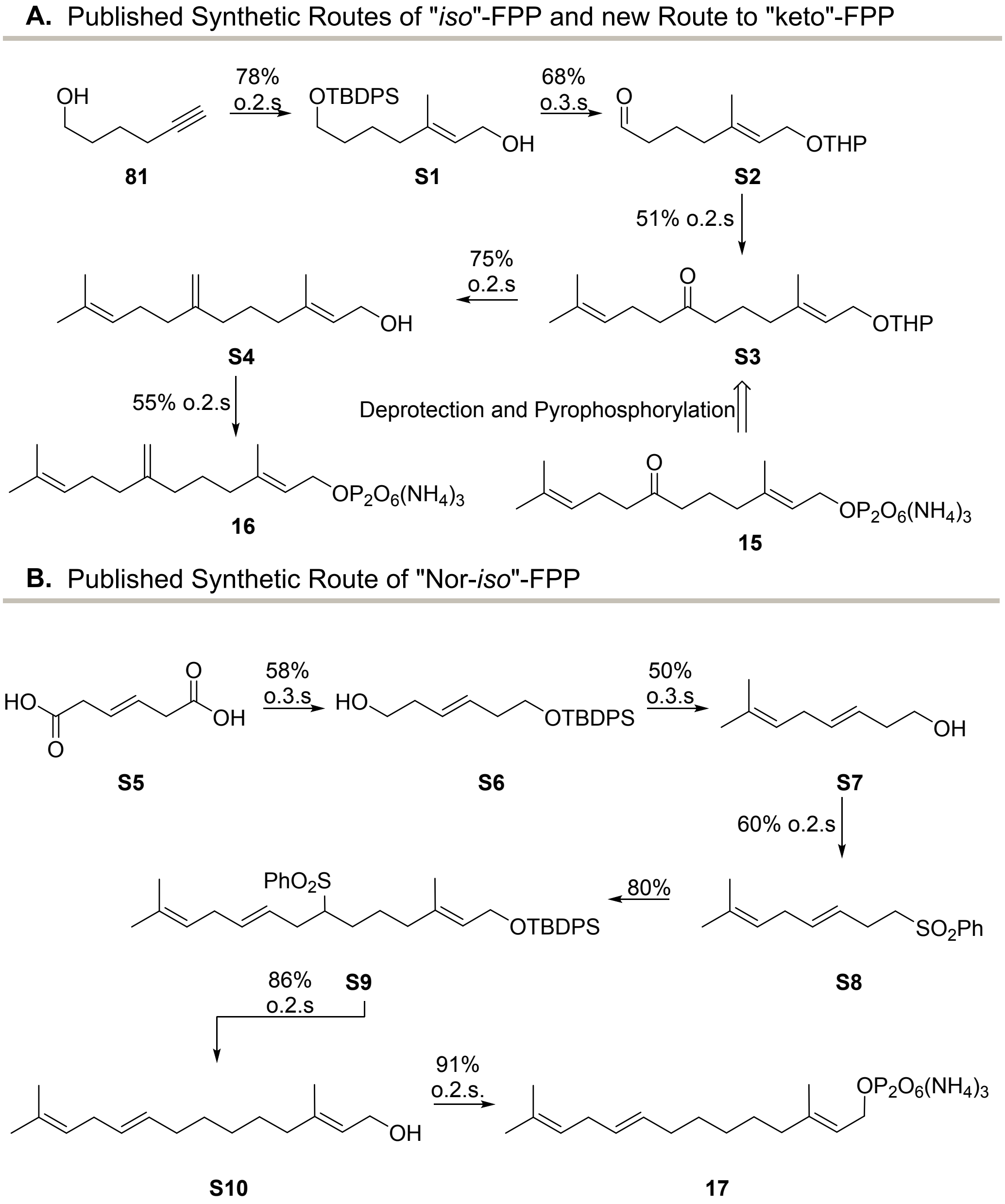

Scheme S1. A. Previous published synthetic route of "*iso*"-FPP, with shared intermediate **S4** as starting point of "keto"-FPP derivative **15**. [S11] **B**. Published synthetic route of "Nor-*iso*"-FPP. [S12]

1.2.2 Chemical Synthesis

Alcohol S12

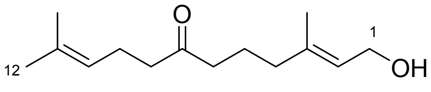

S12
$C_{14}H_{24}O_2$
224.34

THP ether **S4** (152 mg, 0.49 mmol, 1.00 eq.) was dissolved in dry EtOH (10 mL) and PPTS (50 mg, 0.20 mmol, 0.41 eq.) was added. The reaction mixture was warmed to 50 °C and stirred for 3 h, before adding water and EtOAc. The phases were separated and the aqueous phase was extracted with EtOAc (3x). The combined organic phases were washed with brine, dried over $MgSO_4 \cdot H_2O$, filtered and the solvent was removed *in vacuo*. The crude product was purified by column chromatography (PE:EtOAc, 3.33:1→1:1) and alcohol **S12** (101 mg, 0.45 mmol, 92%) was obtained as a colorless oil with a *t*-butyl ketone as an inseparable by-product from the previous two synthetic steps. The analytical data is in accordance to the literature, with respect to different deuterated solvents. [S13]

R_f = 0.30 (*n*-pentane:EtOAc, 3:1); ^{1}H NMR (500 MHz, $CDCl_3$): δ = 5.41 – 5.38 (m, 1H, H_2), 5.06 – 5.04 (m, 1H, H_{10}), 4.15 (d, J = 6.9 Hz, 2H, H_1), 2.41 (t, J = 7.5 Hz, 2H, H_8), 2.37 (t, J = 7.3 Hz, 2H, H_6), 2.26 – 2.22 (m, 2H, H_9), 2.01 (t, J = 7.5 Hz, 2H, H_4), 1.74 – 1.68 (m, 2H, H_5), 1.67 (s, 3H, $H_{12/14}$), 1.66 (s, 3H, H_{13}), 1.61 (s, 3H, $H_{12/14}$) ppm; ^{13}C NMR (126 MHz, $CDCl_3$): δ = 210.9 (C_7), 139.2 (C_3), 132.8 (C_{11}), 124.2 (C_2), 122.9 (C_{10}), 59.5 (C_1), 43.0 (C_8), 42.2 (C_6), 39.0 (C_4), 25.8 ($C_{12/14}$), 22.7 (C_9), 21.7 (C_5), 17.8 ($C_{12/14}$), 16.2 (C_{13}) ppm; HRMS [GCMS-CI]: *m/z* calcd for $C_{14}H_{22}O$ $[M+H_2O]^+$: 206.1671, found: 206.1673.

FPP derivative 15

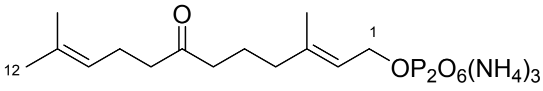

15
$C_{14}H_{35}N_3O_8P_2$
435.39

Alcohol **S12** (95 mg, 0.42 mmol, 1.00 eq.) was dissolved in Et_2O (16 mL) and cooled to 0 °C. PBr_3 (0.02 mL, 57 mg, 0.21 mmol) was added dropwise and after approx. 30 min, the same amount of PBr_3 was added, again. After additional 50 min, water was added and the phases were separated. The aqueous phase was extracted with *n*-pentane (3x) and the combined organic phases were washed with an aq. sat. $NaHCO_3$ solution, dried over $MgSO_4 \cdot H_2O$, filtered and the solvent was carefully removed *in vacuo*. Tris(tetra-*n*-butylammonium) hydrogen pyrophosphate (770 mg, 0.85 mmol, 2.03 eq.) was dissolved in MeCN (7 mL) and the crude bromide dissolved in MeCN (3 mL + 5 mL + 5 mL) was added to the solution. The reaction stirred at rt o/n before the solvent was removed *in vacuo*. The residue was loaded onto the ion exchange column as described in the General Information. FPP derivative **15** (181 mg, 0.42 mmol, 99%) was obtained as a fluffy solid. The analytical data is in accordance to the literature.[S13]

^{1}H NMR (400 MHz, D_2O): δ = 5.48 – 5.44 (m, 1H, H_2), 5.16 – 5.12 (m, 1H, H_{10}), 4.49 (dd, J = 6.6 Hz, 6.6 Hz, 2H, H_1), 2.58 (t, J = 7.1 Hz, 2H, H_8), 2.54 (t, J = 7.4 Hz, 2H, H_6), 2.29 – 2.23 (m, 2H, H_9), 2.06 (t, J = 7.3 Hz, 2H, H_4), 1.73 – 1.66 (m, 8H, H_5, $H_{12/14}$, H_{13}), 1.63 (s, 3H, $H_{12/14}$) ppm; ^{13}C NMR (101 MHz, D_2O): δ = 219.2 (C_7), 141.8 (C_3), 134.4 (C_{11}), 122.5 (C_{10}), 120.5 (d, J = 8.5 Hz, C_2), 62.5 (d, J = 5.2 Hz, C_1), 42.2 (C_8), 41.8 (C_6), 38.0 (C_4), 24.8 ($C_{12/14}$), 22.4 (C_9), 21.1 (C_5), 16.8 ($C_{12/14}$), 15.3 (C_{13}) ppm; ^{31}P NMR (162 MHz, D_2O): δ = −10.0 (d, J = 18.7 Hz), −6.7 (d, J = 19.2 Hz) ppm.

Epoxid **36**

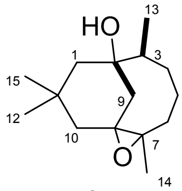

35
$C_{15}H_{26}O_2$
238.37

This reaction was not performed dry or under inert atmosphere. **35** (10 mg, 45 µmol, 1.00 eq.) was dissolved in CH_2Cl_2 (3 mL) and *m*-CPBA (13 mg, 75 µmol, 1.67 eq.) was added at room temperature. After approximately 20 min an aq. sat. $NaHCO_3$ solution was added and the phases were separated. The aqueous phase was extracted with *n*-pentane (3x) and the combined organic phases were dried over $MgSO_4 \cdot H_2O$, filtered and the solvent was removed *in vacuo*. The crude product was purified by column chromatography (*n*-pentane:EtOAc, 3:1) and epoxide **36** (8 mg, 34 µmol, 75%) was isolated a colourful oil.

R_f = 0.48 (PE:EtOAc, 8:1); ^{1}H NMR (600 MHz, C_6D_6): δ = 2.07 (dt, J = 12.4 Hz, 2.6 Hz, 1H, H_9), 1.89 – 1.85 (m, 1H, H_6), 1.69 – 1.64 (m, 2H, H_1, H_3), 1.63 – 1.52 (m, 4H, H_4, H_6, H_9, H_{10}), 1.49 – 1.44 (m, 1H, H_5), 1.36 (dt, J = 13.9 Hz, 1.9 Hz, 1H, H_{10}), 1.18 (s, 3H, H_{14}), 1.16 – 1.08 (m, 2H, H_4, H_5), 1.00 (d, J = 13.6 Hz, 1H, H_1), 0.91 (d, J = 7.1 Hz, 3H, H_{13}), 0.84 (s, 3H, H_{12}/H_{15}), 0.83 (s, 3H, H_{12}/H_{15}) ppm; ^{13}C NMR (151 MHz, C_6D_6): δ = 74.9 (C_2), 64.9 (C_7), 64.5 (C_8), 49.8 (C_1), 44.0 (C_{10}), 43.9 (C_9), 39.8 (C_3), 39.0 (C_6), 36.1 (C_{12}/C_{15}), 32.6 (C_4), 31.5 (C_{11}), 28.8 (C_{12}/C_{15}), 26.9 (C_5), 18.6 (C_{14}), 17.9 (C_{13}) ppm; HRMS [GC-MS, CI]: *m/z* calcd for $C_{14}H_{22}O$ $[M]^+$: 206.1671, found: 206.1671.

1.2.3 Experimental Procedures of Biotransformation

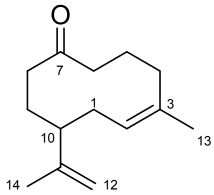

25
$C_{14}H_{22}O$
206.33

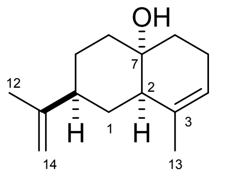

26a
$C_{14}H_{22}O$
206.33

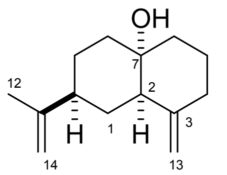

26b
$C_{14}H_{22}O$
206.33

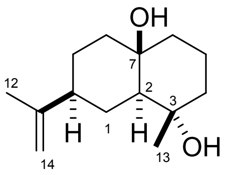

27a
$C_{14}H_{24}O_2$
224.34

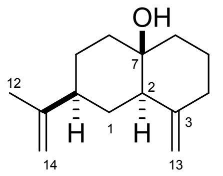

27b
$C_{14}H_{22}O$
206.33

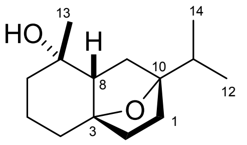

28a
$C_{14}H_{24}O_2$
224.34

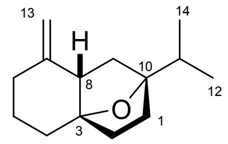

28b
$C_{14}H_{22}O$
206.33

FPP derivative **15** (461 mg, 1.06 mmol, 1.00 eq.) was dissolved in an aq. NH_4HCO_3 solution (0.05 M, 56.5 mL). The reaction was performed in fourteen 50 mL batches. The following procedure will be described for one 50 mL batch. Tween20® (10µL) and PPase (1 µL) were dissolved in HEPES buffer (44.3 mL). FPP derivative **15** (500 µL) and Omp7 enzyme solution (3.58 mg/mL, 700 µL) were added and the reaction was started by the addition of $MgCl_2$ (2 M, 250 µL). The reaction was stirred at 37 °C and 100 rpm and every 30 min more FPP derivative **15** (500 µL) was added to a total volume of 4 mL. After 2 h another batch of Omp7

enzyme solution (698 µL) was added. The reaction was then allowed to stir at 37 °C and 100 rpm o/n, before adding *n*-pentane. The phases were separated and the aqueous phase was extracted with *n*-pentane (3x). The combined organic phases were washed with brine and dried over $MgSO_4{\cdot}H_2O$, filtered and the solvent was carefully removed *in vacuo*. The crude product (about 80 mg) was purified by column chromatography (*n*-pentane:Et_2O, 10:1, 5:1, 3:1, 1:1) and the isolated compounds were prepared for NMR analysis by slow evaporation of the solvents residues and if necessary, also by co-evaporation with C_6D_6. **28b** (6 mg), **25** (30 mg), **28a** (7 mg, then co-evaporated), **26b** (3 g, then co-evaporated), **26a** (3 mg, then co-evaporated) were isolated from the first column chromatography. After a second run of mixed fractions (*n*-pentane:Et_2O, 10:1) **27b** (3 mg) were isolated. **27a** (before prep GC: 27 mg) was further purified by preparative GC (see General Information). All products appeared as colourless oils.

Analytical data of **28b**

R_f = 0.65 (PE:EtOAc, 8:1); ^{1}H NMR (600 MHz, C_6D_6): δ = 4.92 (m, 1H, H_{13}), 4.71 (m, 1H, H_{13}), 2.36 – 2.33 (m, 1H, H_6), 2.19 – 2.18 (m, 1H, H_8), 2.11 – 2.06 (m, 2H, H_4, H_{11}), 1.90 – 1.85 (m, 2H, H_6, H_9), 1.82 – 1.74 (m, 1H, H_5), 1.60 (dd, J = 11.9 Hz, 8.6 Hz, 1H, H_9), 1.53 – 1.41 (m, 4H, H_2, H_1, H_4, H_5), 1.39 – 1.30 (m, 2H, H_2, H_1), 0.98 (d, J = 5.9 Hz, 3H, $H_{12/14}$), 0.97 (d, J = 5.9 Hz, 3H, $H_{12/14}$) ppm; ^{13}C NMR (151 MHz, C_6D_6): δ = 151.2 (C_7), 108.3 (C_{13}), 89.2 (C_{10}), 84.4 (C_3), 48.4 (C_8), 38.2 (C_9), 38.0 (C_2), 35.6 (C_6), 33.4 (C_{11}), 32.6 (C_1), 31.7 (C_4), 23.8 (C_5), 18.4 ($C_{12/14}$), 18.3 ($C_{12/14}$) ppm; HRMS [GC-MS, CI]: *m/z* calcd for $C_{14}H_{22}O$ $[M]^+$: 206.1671, found: 206.1666.

Analytical data of **25**

R_f = 0.48 (PE:EtOAc, 8:1); ^{1}H NMR (600 MHz, C_6D_6): δ = 5.07 (t, J = 7.4 Hz, 1H, H_2), 4.90 (s, 1H, H_{12}), 4.80 (s, 1H, H_{12}), 2.30 – 1.62 (m, 13H, H_1, H_4, H_5, H_6, H_8, H_9, H_{10}), 1.60 (s, 3H, H_{14}), 1.54 – 1.53 (m, 3H, H_{13}) ppm; ^{13}C NMR (151 MHz, C_6D_6): δ = 148.0 (C_{11}), 134.7 (C_3), 125.4 (C_2), 110.9 (C_{12}), 45.4 (C_{10}), 42.5 (C_8), 34.7 (C_6), 29.1 (C_1), 28.1 (C_4), 26.8 (C_9), 22.9 (C_{13}), 22.8 (C_{14}), 19.7 (C_5) ppm; HRMS [GC-MS, CI]: *m/z* calcd for $C_{14}H_{22}O$ $[M]^+$: 206.1671, found: 206.1671.

Analytical data of **28a**

R_f = 0.35 (PE:EtOAc, 8:1); ^{1}H NMR (600 MHz, C_6D_6): δ = 3.44 (s, 1H, H_{OH}), 2.08 – 2.03 (m, 2H, H_9, H_{11}), 2.01 – 1.94 (m, 2H, H_4, H_5), 1.80 (d, J = 14.5 Hz, 1H, H_6), 1.40 – 1.17 (m, 8H, H_1, H_2, H_4, H_5, H_8, H_9), 1.08 – 1.03 (m, 1H, H_6), 1.07 (s, 3H, H_{13}), 0.95 (d, J = 7.0 Hz, 3H, $H_{12/14}$), 0.93 (d, J = 6.9 Hz, 3H, $H_{12/14}$) ppm; ^{13}C NMR (151 MHz, C_6D_6): δ = 90.3 (C_{10}), 85.2 (C_3), 70.0 (C_7), 51.0 (C_8), 38.8 (C_6), 37.3 (C_2), 33.8 (C_9), 33.2 (C_{11}), 32.8 (C_1), 30.3 (C_4), 27.8 (C_{13}), 18.4 ($C_{12/15}$), 18.2 ($C_{12/14}$), 18.1 (C_5) ppm; HRMS [GC-MS, CI]: *m/z* calcd for $C_{14}H_{24}O_2$ $[M]^+$: 224.1776, found: 224.1778.

Analytical data of **26b**

R_f = 0.23 (PE:EtOAc, 8:1); ^{1}H NMR (600 MHz, C_6D_6): δ = 4.76 (d, J = 9.9 Hz, 2H, H_{14}), 4.74 (s, 2H, H_{13}), 2.07 (dd, J = 12.2 Hz, 4.1 Hz, 1H, H_2), 1.94 – 1.92 (m, 2H, H_4), 1.85 (tt, J = 18.1 Hz, 3.5 Hz, 1H, H_{10}), 1.74 – 1.62 (m, 3H, H_5, H_6, H_8), 1.61 (s, 3H, H_{12}), 1.59 – 1.54 (m, 2H, H_1, H_9), 1.52 – 1.46 (m, 2H, H_1, H_8), 1.44 – 1.41 (m, 1H, H_5), 1.40 (s, 1H, H_{OH}), 1.23

(dd, $J = 12.5$ Hz, 3.5 Hz, 1H, H_9), 1.16 (d, $J = 12.4$ Hz, 1H, H_6) ppm; ^{13}C NMR (151 MHz, C_6D_6): $\delta = 150.3$ (C_3), 149.5 (C_{11}), 110.7 (C_{13}), 108.9 (C_{14}), 71.8 (C_7), 54.3 (C_2), 45.2 (C_{10}), 40.4 (C_8), 35.4 (C_1), 30.8 (C_6), 30.3 (C_4), 29.1 (C_9), 23.2 (C_5), 21.2 (C_{12}) ppm; HRMS [GC-MS, CI]: *m/z* calcd for $C_{14}H_{22}O$ $[M]^+$: 206.1671 , found: 206.1670.

Analytical data of **26a**

$R_f = 0.18$ (PE:EtOAc, 8:1); ^1H NMR (600 MHz, C_6D_6): $\delta = 5.24$ (s, 1H, H_4), 4.76 (m, 2H, H_{14}), 2.18 – 2.08 (m, 1H, H_5), 1.98 – 1.94 (m, 1H, H_1), 1.90 – 1.85 (m, 2H, H_5, H_{10}), 1.77 (dt, $J = 13.1$ Hz, 3.4 Hz, 1H, H_8), 1.75 – 1.70 (m, 2H, H_2, H_6), 1.66 (td, $J = 13.4$ Hz, 4.0 Hz, 1H, H_8), 1.61 (s, 3H, H_{12}), 1.59 – 1.57 (m, 1H, H_9), 1.56 (m, 3H, H_{13}), 1.52 (s, 1H, H_{OH}), 1.24 – 1.14 (m, 2H, H_6, H_9), 0.97 (dt, $J = 12.8$ Hz, 12.4 Hz, 1H, H_1) ppm; ^{13}C NMR (151 MHz, C_6D_6): $\delta = 149.7$ (C_{11}), 135.2 (C_3), 120.5 (C_4), 109.0 (C_{14}), 69.8 (C_7), 49.5 (C_2), 45.5 (C_{10}), 40.1 (C_8), 36.4 (C_1), 29.0 (C_9), 27.3 (C_6), 22.4 (C_{13}), 22.3 (C_5), 20.9 (C_{12}) ppm; HRMS [GC-MS, CI]: *m/z* calcd for $C_{14}H_{20}$ $[M-H_2O]^+$: 188.1565 , found: 188.1569.

Analytical data of **27b**

$R_f = 0.38$ (PE:EtOAc, 8:1); ^1H NMR (400 MHz, C_6D_6): $\delta = 4.89$ – 4.88 (m, 1H, H_{14}), 4.83 – 4.82 (m, 1H, H_{14}), 4.79 – 4.77 (m, 1H, H_{13}), 4.58 – 4.57 (m, 1H, H_{13}), 2.18 – 2.12 (m, 1H, H_4), 1.86 – 1.73 (m, 5H, H_2, H_4, H_8, H_9, H_{10}), 1.71 (m, 3H, H_{12}), 1.58 – 1.45 (m, 6H, H_1, H_5, H_6, H_8), 1.19 (s, 1H, H_{OH}), 1.19 – 1.10 (m, 2H, H_6, H_8) ppm; ^{13}C NMR (101 MHz, C_6D_6): $\delta = 150.4$ (C_{11}), 149.9 (C_3), 109.0 (C_{14}), 108.5 (C_{13}), 71.1 (C_7), 49.5 (C_2), 45.5 (C_{10}), 39.8 (C_6), 39.0 (C_8), 36.8 (C_4), 29.5 (C_1), 26.8 (C_9), 24.0 (C_5), 21.0 (C_{12}) ppm; HRMS [GC-MS, CI]: *m/z* calcd for $C_{14}H_{23}O$ $[M+H^+]^+$: 207.1749, found: 207.1756.

Analytical data of **27a**

$R_f = 0.13$ (PE:EtOAc, 5:1); ^1H NMR (600 MHz, C_6D_6): $\delta = 4.87$ – 4.83 (m, 2H, H_{14}), 1.96 – 1.93 (m, 1H, H_1), 1.85 – 1.80 (m, 1H, H_{10}), 1.72 (s, 3H, H_{12}), 1.71 – 1.63 (m, 2H, H_4, H_5), 1.51 – 1.45 (m, 2H, H_9), 1.40 (dt, $J = 12.4$ Hz, 12.3 Hz, 1H, H_1), 1.36 – 1.33 (m, 1H, H_5), 1.31 – 1.26 (m, 1H, H_4), 1.26 (s, 3H, H_{13}), 1.22 – 1.17 (m, 2H, H_6, H_8), 1.13 – 1.07 (m, 2H, H_2, H_8), 1.01 (td, $J = 20.1$ Hz, 4.2 Hz, 1H, H_6), 0.66 (s, 1H, H_{OH}), 0.16 (s, 1H, H_{OH}) ppm; ^{13}C NMR (151 MHz, C_6D_6): $\delta = 150.4$ (C_{11}), 108.8 (C_{14}), 71.8 (C_3), 70.7 (C_7), 53.2 (C_2), 46.0 (C_{10}), 43.5 (C_4), 41.6 (C_8), 40.3 (C_6), 27.0 (C_9), 26.4 (C_1), 23.5 (C_{13}), 21.2 (C_{12}), 20.0 (C_5) ppm; HRMS [GC-MS, CI]: *m/z* calcd for $C_{14}H_{22}O$ $[M-H_2O]^+$: 206.1671, found: 206.1664.

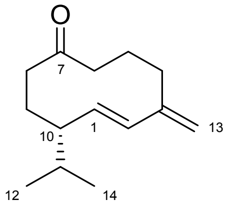

29

$C_{14}H_{22}O$

206.33

FPP derivative **15** (270 mg, 0.62 mmol, 1.00 eq.) was dissolved in an aq. NH_4HCO_3 solution (0.05 M, 33 mL). The reaction was performed in eight 50 mL batches. The following procedure

will be described for one 50 mL batch. Tween20® (10µL) and PPase (1 µL) were dissolved in HEPES buffer (41.5 mL). FPP derivative **15** (500 µL) and Cop4 enzyme solution (1.19 mg/mL, 2.10 mL) were added and the reaction was started by the addition of $MgCl_2$ (2 M, 250 µL). The reaction was stirred at 37 °C and 100 rpm and every 30 min more FPP derivative **15** (500 µL) was added to a total volume of 4 mL. After 2 h another batch of Cop4 enzyme solution (2.00 mL) was added. The reaction was then allowed to stir at 37 °C and 100 rpm o/n, before adding *n*-pentane. The phases were separated and the aqueous phase was extracted with *n*-pentane (3x). The combined organic phases were washed with brine, while a mix-phase was first frozen, then thawed to enhance phase separation. The aqueous phase was extracted with n-pentane (1x) and organic phases were dried over $MgSO_4{\cdot}H_2O$. The crude product was purified by column chromatography (*n*-pentane:Et_2O, 30:1 → 10:1) and the isolated compound was prepared for NMR analysis by slow evaporation of the solvents residues and also by co-evaporation with C_6D_6. **29** (23 mg pre co-evaporation) was obtained as a colourless oil. The analytical data is in accordance with the literature. [S14]

R_f = 0.80 (*n*-pentane:EtOAc, 8:1); ^{1}H NMR (600 MHz, C_6D_6): δ = 5.90 (d, J = 15.8 Hz, 1H, H_2), 5.25 (dd, J = 15.8 Hz, 10.5 Hz, 1H, H_1), 4.85 (s, 1H, H_{13}), 4.82 (m, 1H, H_{13}), 2.55 (dt, J = 12.3 Hz, 4.6 Hz, 1H, H_4), 2.32 – 2.24 (m, 2H, H_5, H_6), 2.13 – 2.06 (m, 1H, H_9), 2.00 (ddd, J = 13.0 Hz, 5.9 Hz, 2.1 Hz, 1H, H_4), 1.89 – 1.80 (m, 3H, H_6, H_8), 1.59 – 1.54 (m, 1H, H_9), 1.48 – 1.43 (m, 1H, H_{10}), 1.35 – 1.29 (m, 1H, H_{11}), 1.23 – 1.17 (m, 1H, H_5), 0.80 (d, J = 6.7 Hz, 3H, $H_{12/14}$), 0.78 (d, J = 6.7 Hz, 3H, $H_{12/14}$) ppm; ^{13}C NMR (126 MHz, C_6D_6): δ = 210.6 (C_7), 147.0 (C_3), 136.9 (C_1), 132.7 (C_2), 113.5 (C_{13}), 52.9 (C_{10}), 42.4 (C_8), 42.0 (C_6), 32.0 (C_{11}), 31.8 (C_9), 30.3 (C_4), 24.6 (C_5), 21.0 ($C_{12/14}$), 20.9 ($C_{12/14}$) ppm; HRMS [GC-MS, CI]: *m/z* calcd for $C_{14}H_{22}O$ $[M]^+$: 206.1671, found: 206.1677.

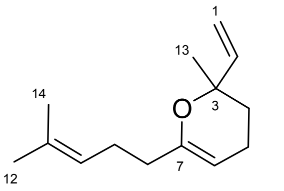

30
$C_{14}H_{22}O$
206.33

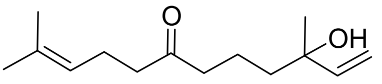

31
$C_{14}H_{24}O_2$
224.34

FPP derivative **15** (172 mg, 0.40 mmol, 1.00 eq.) was dissolved in an aq. NH_4HCO_3 solution (0.05 M, 21.1 mL). The reaction was performed in ten 25 mL batches. The following procedure will be described for one 25 mL batch. Tween20® (5 µL) and PPase (0.5 µL) were dissolved in HEPES buffer (22.3 mL). FPP derivative **15** (250 µL) and GCoA enzyme solution (4.28 mg/mL, 292 µL) were added, and the reaction was started by the addition of $MgCl_2$ (2 M, 125 µL). The reaction was stirred at 37 °C and 100 rpm and every 30 min more FPP derivative **15** (250 µL) was added to a total volume of 2 mL. After 2 h another batch of GCoA enzyme solution (292 µL) was added. The reaction was then allowed to stir at 37 °C and 100 rpm o/n, before adding *n*-pentane. The phases were separated, and the aqueous phase was extracted with *n*-pentane (3x). The combined organic phases were washed with brine and dried over $MgSO_4{\cdot}H_2O$. The crude product (approximately 120 mg) was purified multiple times by column chromatography (*n*-pentane:Et_2O, 4:1 → 2:1, then *n*-pentane 100% → *n*-pentane:MTBE 30:1 → 10:1). **31** (49 mg pre C_6D_6 coevaporation) was isolated as a colorless

oil. During column chromatography the non-polar product **30** decomposed resulting in the isolation of polar product **31**. At the same time, GC measurements of **31** also showed significant amounts of **30**. To isolate cyclic enol ether **30** preparative GC was used (see General Information), trying to take advantage of the heat induced reaction. **30** was isolated with small amounts of **31** also present during NMR measurements, which still allow full characterization of **30**. The analytical data of **30** is in accordance with the literature (assignment given). [S13]

Analytical data of **30**

R_f = 0.35 (*n*-pentane 100%); ^{1}H NMR (600 MHz, C_6D_6): δ = 5.75 (dd, *J* = 17.3 Hz, 10.8 Hz, 1H, H_2), 5.28 – 5.22 (m, 2H, H_1, H_{10}), 5.00 (d, *J* = 10.8 Hz, 1H, H_1), 4.51 (t, *J* = 3.3 Hz, 1H, H_6), 2.43 – 2.32 (m, 2H, H_9), 2.23 (t, *J* = 7.6 Hz, 2H, H_8), 1.92 – 1.80 (m, 2H, H_5), 1.65 (s, 3H, $H_{12/14}$), 1.56 (s, 3H, $H_{12/14}$), 1.52 – 1.42 (m, 2H, H_4), 1.26 (s, 3H, H_{13}) ppm; ^{13}C NMR (151 MHz, C_6D_6): δ = 152.7 (C_7), 142.7 (C_2), 131.4 (C_{11}), 124.8 (C_{10}), 112.5 (C_1), 94.3 (C_6), 75.9 (C_3), 35.3 (C_8), 32.1 (C_4), 26.4 (C_{13}), 26.4 (C_9), 25.9 ($C_{12/14}$), 18.7 (C_5), 17.8 ($C_{12/14}$) ppm; HRMS [GC-MS, CI]: *m/z* calcd for $C_{14}H_{22}O$ $[M]^+$: 206.1671, found: 206.1679.

Analytical data of **31**

R_f = 0.33 (*n*-pentane:EtOAc, 5:1); ^{1}H NMR (400 MHz, C_6D_6): δ = 5.75 (dd, *J* = 17.3 Hz, 10.7 Hz, 1H), 5.21 (dd, *J* = 17.3 Hz, 1.6 Hz, 1H), 5.12 – 5.07 (m, 1H), 4.95 (dd, *J* = 10.7 Hz, 1.6 Hz, 1H), 2.27 (dt, *J* = 7.3 Hz, 7.2 Hz, 2H), 2.06 (t, *J* = 7.3 Hz, 2H), 1.98 (t, *J* = 7.1 Hz, 2H), 1.62 (m, 3H), 1.61 – 1.55 (m, 2H), 1.53 (s, 3H), 1.35 – 1.30 (m, 2H), 1.11 (s, 3H) ppm; ^{13}C NMR (101 MHz, C_6D_6): δ = 208.8, 145.6, 132.2, 123.8, 111.5, 72.8, 42.8, 42.6, 41.9, 28.2, 25.8, 22.9, 18.5, 17.7 ppm; HRMS [GC-MS, CI]: *m/z* calcd for $C_{14}H_{24}O_2$ $[M]^+$: 224.1776, found: 224.1778.

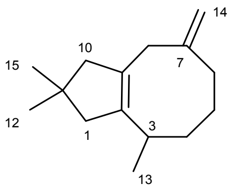

33
$C_{15}H_{24}$
204.36

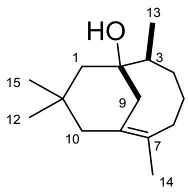

35
$C_{15}H_{26}O$
222.37

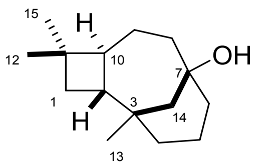

34
$C_{15}H_{26}O$
222.37

FPP derivative **16** (378 mg, 0.87 mmol, 1.00 eq.) was dissolved in an aq. NH_4HCO_3 solution (0.05 M, 46.5 mL). The reaction was performed in eleven 50 mL batches. The following procedure will be described for one 50 mL batch. Tween20® (10 µL) and PPase (1 µL) were dissolved in HEPES buffer (44.4 mL). FPP derivative **16** (500 µL) and Omp7 enzyme solution (3.68 mg/mL, 1.36 mL) were added, and the reaction was started by the addition of $MgCl_2$ (2 M, 250 µL). The reaction was stirred at 37 °C and 100 rpm and every 30 min more FPP derivative **16** (500 µL) was added to a total volume of 4 mL. After 2 h another batch of Omp7 enzyme solution (1.36 mL) was added. The reaction was then allowed to stir at 37 °C and 100 rpm o/n, before adding *n*-pentane. The phases were separated (ultrasonic), and the aqueous phase was extracted with *n*-pentane (3x). The combined organic phases were dried over $MgSO_4 \cdot H_2O$, filtered and the solvent was carefully removed *in vacuo.* The crude product was purified by column chromatography (*n*-pentane 100% → *n*-pentane:Et_2O, 8:1 → Et_2O 100%). **35** (20 mg pre C_6D_6 co-evaporation), **33** (9 mg pre C_6D_6 co-evaporation) and **34** (4 mg pre C_6D_6 co-evaporation) were obtained as colorless oils.

Analytical data of **33**

R_f = 0.92 (*n*-pentane 100%); ^{1}H NMR (600 MHz, C_6D_6): δ = 4.79 (m, 1H, H_{14}), 4.74 – 4.73 (m, 1H, H_{14}), 3.01 – 2.95 (m, 2H, H_3, H_8), 2.59 (d, J = 15.1 Hz, 1H, H_8), 2.31 – 2.27 (m, 1H, H_6), 2.18 – 2.14 (m, 3H, H_1, H_{10}), 2.13 – 2.08 (m, 1H, H_6), 2.02 – 1.99 (m, 1H, H_1), 1.55 – 1.50 (m, 2H, H_5), 1.46 – 1.40 (m, 1H, H_4), 1.30 – 1.24 (m, 1H, H_4), 1.11 (s, 3H, $H_{12/15}$), 1.06 (s, 3H, $H_{12/15}$), 0.95 (d, J = 6.9 Hz, 3H, H_{13}) ppm; ^{13}C NMR (126 MHz, C_6D_6): δ = 150.4 (C_7), 137.0 (C_2), 132.3 (C_9), 111.2 (C_{14}), 52.7 (C_{10}), 48.1 (C_1), 38.4 (C_8), 37.2 (C_4), 37.2 (C_6), 36.0 (C_{11}), 30.6 ($C_{12/15}$), 30.4 (C_3), 30.1 ($C_{12/15}$), 26.2 (C_5), 19.8 (C_{13}) ppm; HRMS [GC-MS, CI]: *m/z* calcd for $C_{15}H_{24}$ $[M]^+$: 204.1878, found: 204.1883.

Analytical data of **34**

R_f = 0.53 (PE:EtOAc, 8:1); ^{1}H NMR (600 MHz, C_6D_6): δ = 1.93 (dt, J = 14.8 Hz, 5.7 Hz, 1H, H_8), 1.73 – 1.67 (m, 2H, H_5, H_{10}), 1.66 – 1.55 (m, 4H, H_4, H_6, H_8, H_{14}), 1.54 – 1.45 (m, 2H, H_2, H_5), 1.43 (dd, J = 9.5 Hz, 7.4 Hz, 1H, H_1), 1.36 – 1.31 (m, 1H, H_9), 1.27 (dd, J = 11.1 Hz, 9.6 Hz, 1H, H_1), 1.26 – 1.19 (m, 3H, H_6, H_9, H_{14}), 0.99 (s, 3H, H_{15}), 0.96 – 0.90 (m, 1H, H_4), 0.95 (s, 3H, H_{12}), 0.73 (s, 3H, H_{13}) ppm; ^{13}C NMR (126 MHz, C_6D_6): δ = 73.5 (C_7), 54.1 (C_{14}), 50.4 (C_2), 48.9 (C_{10}), 42.4 (C_8), 41.8 (C_6), 35.9 (C_{11}), 35.6 (C_1), 34.0 (C_3), 31.0 (C_4), 30.5 (C_{15}), 30.3 (C_{13}), 24.4 (C_9), 22.2 (C_5), 20.3 (C_{12}) ppm; HRMS [GC-MS, CI]: *m/z* calcd for $C_{15}H_{26}O$ $[M]^+$: 222.1984, found: 222.1980.

Analytical data of **35**

R_f = 0.50 (PE:EtOAc, 5:1); ^{1}H NMR (500 MHz, C_6D_6): δ = 3.02 (d, J = 11.4 Hz, 1H, H_9), 2.51 (dd, J = 12.6 Hz, 12.6 Hz, 1H, H_6), 2.21 (d, J = 12.6 Hz, 1H, H_{10}), 1.92 – 1.83 (m, 2H, H_3, H_4), 1.73 – 1.68 (m, 2H, H_5, H_6), 1.66 (d, J = 13.4 Hz, 1H, H_1), 1.62 – 1.59 (m, 5H, H_9, H_{10}, H_{14}), 1.36 – 1.24 (m, 1H, H_5), 1.18 (d, J = 16.1 Hz, 1H, H_4), 1.14 (d, J = 13.8 Hz, 1H, H_1), 0.92 (s, 3H, H_{12}/H_{15}), 0.89 (d, J = 6.7 Hz, 3H, H_{13}), 0.86 (s, 3H, H_{12}/H_{15}) ppm; ^{13}C NMR (126 MHz, C_6D_6): δ = 129.7 (C_7/C_8), 129.4 (C_7/C_8), 77.2 (C2), 51.2 (C_1), 44.3 (C_9), 43.8 (C_{10}), 38.6 (C_3), 35.3 (C_6), 35.2 (C_{11}), 35.1 (C_{12}/C_{15}), 33.8 (C_4), 31.2 (C_5), 28.2 (C_{12}/C_{15}), 19.4 (C_{14}), 18.1 (C_{13}) ppm; HRMS [GC-MS, EI]: *m/z* calcd for $C_{15}H_{26}O$ $[M]^+$: 222.1984, found: 222.1989.

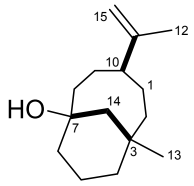

37
$C_{15}H_{26}O$
222.37

FPP derivative **16** (65 mg, 0.15 mmol, 1.00 eq.) was dissolved in an aq. NH_4HCO_3 solution (0.05 M, 8 mL). The reaction was performed in one 100 mL batch. Tween20® (20 µL) and PPase (2 µL) were dissolved in HEPES buffer (87 mL). FPP derivative **16** (1 mL) and Pts enzyme solution (2.1 mg/mL, 2.38 mL) were added, and the reaction was started by the addition of $MgCl_2$ (2 M, 500 µL). The reaction was stirred at 37 °C and 100 rpm and every 30 min more

FPP derivative **16** (1 mL) was added to a total volume of 8 mL. After 2 h another batch of Pts enzyme solution (2.38 mL) was added. The reaction was then allowed to stir at 37 °C and 100 rpm o/n, before adding *n*-pentane. The phases were separated (freeze and thaw) and the aqueous phase was extracted with *n*-pentane (3x). The combined organic phases were dried over $MgSO_4{\cdot}H_2O$, filtered and the solvent was carefully removed *in vacuo*. The crude product was purified by column chromatography (*n*-pentane:Et_2O, 10:1). After co-evaporation with C_6D_6 **37** (< 1 mg) was obtained as a colorless oil.

R_f = 0.36 (*n*-pentane:Et_2O, 2:1); ^{1}H NMR (600 MHz, C_6D_6): δ = 4.81 (m, 1H, H_{15}), 4.74 – 4.73 (m, 1H, H_{15}), 1.90 – 1.84 (m, 2H, H_{10}, H_{14}), 1.69 – 1.58 (m, 7H, H_1, H_8, H_9, H_{12}), 1.57 – 1.40 (m, 5H, H_2, H_5, H_6, H_8), 1.37 – 1.31 (m, 1H, H_1), 1.21 – 1.16 (m, 1H, H_6), 1.14 – 1.12 (m, 1H, H_4), 1.10 – 1.05 (m, 1H, H_2), 1.01 – 0.96 (m, 1H, H_4), 0.77 (s, 3H, H_{13}), 0.71 (dt, J = 13.6 Hz, 1.4 Hz, 1H, H_{14}) ppm; ^{13}C NMR (151 MHz, C_6D_6): δ = 152.4 (C_{11}), 108.7 (C_{15}), 72.0 (C_7), 51.8 (C_{10}), 44.0 (C_{14}), 41.1 (C_6), 38.9 (C_4), 37.4 (C_8), 34.9 (C_2), 34.1 (C_3), 32.2 (C_{13}), 28.9 (C_1), 28.2 (C_9), 20.9 (C_5), 2.07 (C_{12}) ppm; HRMS [GC-MS, CI]: *m/z* calcd for $C_{15}H_{26}O$ $[M]^+$: 222.1984, found: 222.1985.

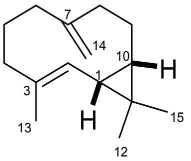

38

$C_{15}H_{26}O$

222.37

FPP derivative **16** (65 mg, 0.15 mmol, 1.00 eq.) was dissolved in an aq. NH_4HCO_3 solution (0.05 M, 8 mL). The reaction was performed in one 100 mL batch. Tween20® (20 µL) and PPase (2 µL) were dissolved in HEPES buffer (84 mL). FPP derivative **16** (1 mL) and JeSTS4 enzyme solution (1.35 mg/mL, 3.71 mL) were added, and the reaction was started by the addition of $MgCl_2$ (2 M, 500 µL). The reaction was stirred at 37 °C and 100 rpm and every 30 min more FPP derivative **16** (1 mL) was added to a total volume of 8 mL. After 2 h another batch of JeSTS4 enzyme solution (3.71 mL) was added. The reaction was then allowed to stir at 37 °C and 100 rpm o/n, before adding *n*-pentane. The phases were separated (freeze and thaw) and the aqueous phase was extracted with *n*-pentane (3x). The combined organic phases were dried over $MgSO_4{\cdot}H_2O$, filtered and the solvent was carefully removed *in vacuo*. The crude product was purified by column chromatography (*n*-pentane 100%). After co-evaporation with C_6D_6 **38** (1 mg) was obtained as a colorless oil.

R_f = 0.62 (*n*-pentane, 100%); ^{1}H NMR (600 MHz, C_6D_6): δ = 4.88 (d, J = 10.4 Hz, 1H, H_2), 4.82 (m, 1H, H_{14}), 4.67 (s, 1H, H_{14}), 2.14 – 2.10 (m, 2H, H_4, H_8), 2.06 – 1.96 (m, 2H, H_6, H_8), 1.83 (td, J = 18.5 Hz, 4.7 Hz, 1H, H_4), 1.71 – 1.64 (m, 1H, H_5), 1.60 (d, J = 1.2 Hz, 3H, H_{13}), 1.58 – 1.50 (m, 3H, H_5, H_6, H_9), 1.33 – 1.26 (m, 2H, H_1, H_9), 1.08 (s, 3H, $H_{12/15}$), 1.01 (s, 3H, $H_{12/15}$), 0.50 (td, J = 12.0 Hz, 9.0 Hz, 2.9 Hz, 1H, H_{10}) ppm; ^{13}C NMR (151 MHz, C_6D_6): δ = 147.9 (C_7), 133.3 (C_3), 122.7 (C_2), 108.2 (C_{14}), 42.0 (C_4), 39.1 (C_8), 31.0 (C_6), 29.6 (C_{10}), 29.1 ($C_{12/15}$), 26.4 (C_1), 22.9 (C_5), 22.1 (C_9), 18.8 (C_{11}), 17.1 (C_{13}), 15.7 ($C_{12/15}$) ppm; HRMS [GC-MS, CI]: *m/z* calcd for $C_{15}H_{25}$ $[M+H]^+$: 205.1956, found: 205.1964.

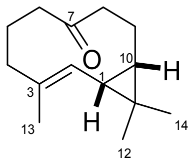

32
$C_{14}H_{22}O$
206.33

FPP derivative **15** (65 mg, 0.15 mmol, 1.00 eq.) was dissolved in an aq. NH_4HCO_3 solution (0.05 M, 8 mL). The reaction was performed in one 100 mL batch. Tween20® (20 µL) and PPase (2 µL) were dissolved in HEPES buffer (81 mL). FPP derivative **15** (1 mL) and JeSTS4 enzyme solution (1.01 mg/mL, 5 mL) were added, and the reaction was started by the addition of $MgCl_2$ (2 M, 500 µL). The reaction was stirred at 37 °C and 100 rpm and every 30 min more FPP derivative **15** (1 mL) was added to a total volume of 8 mL. After 2 h another batch of JeSTS4 enzyme solution (approx. 7 mL) was added. The reaction was then allowed to stir at 37 °C and 100 rpm o/n, before adding *n*-pentane. The phases were separated (freeze and thaw) and the aqueous phase was extracted with *n*-pentane (3x). The combined organic phases were dried over $MgSO_4 \cdot H_2O$, filtered and the solvent was carefully removed *in vacuo*. The crude product was purified by column chromatography (*n*-pentane:Et_2O 10:1). After co-evaporation with C_6D_6 **32** (<1 mg) was obtained as a colorless crystal.

R_f = 0.66 (*n*-pentane:Et_2O, 10:1); ^{1}H NMR (600 MHz, C_6D_6): δ = 4.67 (d, 10.7 Hz, 1H, H_2), 2.38 – 2.31 (m, 1H, H_5), 2.12 – 1.98 (m, 4H, H_4, H_6, H_8), 1.73 (d, J = 1.3 Hz, 3H, H_{13}), 1.66 (td, J = 12.5 Hz, 4.6 Hz, 1H, H_4), 1.59 – 1.51 (m, 2H, H_6, H_9), 1.36 – 1.25 (m, 3H, H_1, H_5, H_9), 1.01 (s, 3H, $H_{12/14}$), 0.93 (s, 3H, $H_{12/14}$), 0.50 (ddd, J = 11.9 Hz, 8.9 Hz, 3.1 Hz, 1H, H_{10}) ppm; ^{13}C NMR (151 MHz, C_6D_6): δ = 206.1 (C_7), 136.3 (C_3), 121.7 (C_2), 44.5 (C_8), 41.5 (C_4), 38.3 (C_6), 29.0 (C_{10}), 28.9 ($C_{12/14}$), 26.0 (C_1), 22.0 (C_9), 21.1 (C_5), 18.9 (C_{11}), 16.4 (C_{13}), 15.4 ($C_{12/14}$) ppm; HRMS [GC-MS, CI]: *m/z* calcd for $C_{14}H_{22}O$ $[M]^+$: 206.1671, found: 206.1673.

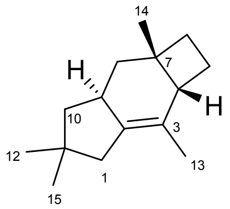

42
$C_{15}H_{24}$
204.36

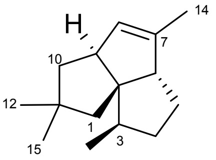

43
$C_{15}H_{24}$
204.36

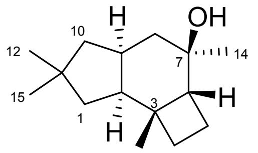

44
$C_{15}H_{26}O$
222.37

FPP (**1**) (530 mg, 1.22 mmol, 1.00 eq.) was dissolved in an aq. NH_4HCO_3 solution (0.05 M, 65 mL). The reaction was performed in eight 100 mL batches. Tween20® (20 µL) and PPase (1 µL) were dissolved in HEPES buffer (83 mL). The following procedure will be described for one 100 mL batch. FPP (**1**) (1 mL) and Omp7 F81W enzyme solution (1.26 mg/mL, 4 mL) were added, and the reaction was started by the addition of $MgCl_2$ (2 M, 500 µL). The reaction was stirred at 37 °C and 100 rpm and every 30 min more FPP (**1**) (1 mL) was added to a total volume of 8 mL. After 2 h another batch of Omp7 F81W enzyme solution (4 mL) was added. The reaction was then allowed to stir at 37 °C and 100 rpm o/n, before adding *n*-pentane. The phases were separated and the aqueous phase was extracted with *n*-pentane (3x). The combined organic phases were dried over $MgSO_4 \cdot H_2O$, filtered and the solvent was carefully removed *in vacuo*. The crude product was purified by prep-GC (see General Information). All products appeared as colourless oils in cooling traps.

Since the superposition of ^1H NMR doesn't allow the analysis of the exact chemical shifts, only the ^{13}C NMR data will be given exclusively for each compound while the ^1H NMR data will be given as the mixture. HRMS measurements were performed with the mixture of pentalenene and sterpurene without separation on GC. GC-MS using a WAX coloumn was also performed and is displayed below.

Analytical data of **43**

$R_f = 0.79$ (*n*-heptane 100%); ^1H NMR (600 MHz, C_6D_6): $\delta = 5.22$ (s, 1H), 2.71 – 2.62 (m, 2H), 2.54 – 2.52 (m, 1H), 2.42 – 2.35 (m, 1H), 2.16 – 2.10 (m, 2H), 2.03 – 1.98 (m, 1H), 1.85 – 1.80 (m, 1H), 1.79 – 1.74 (m, 2H), 1.72 – 1.62 (m, 3H), 1.60 – 1.58 (m, 6H), 1.57 – 1.47 (m, 3H), 1.39 – 1.34 (m, 2H), 1.31 – 1.28 (m, 2H), 1.22 (s, 3H), 1.12 – 1.08 (m, 4H), 1.08 (s, 3H), 1.04 (s, 3H), 1.03 (s, 3H) ppm; ^{13}C NMR (151 MHz, C_6D_6): $\delta = 140.7$ (C_7), 130.1 (C_8), 65.1 (C_2), 62.4 (C_6), 59.9 (C_9), 49.3 (C_1), 47.1 (C_{10}), 45.0 (C_3), 40.7 (C_{11}), 33.8 (C_4), 30.2 ($C_{12/15}$), 29.3 ($C_{12/15}$), 27.9 (C_5), 17.2 (C_{13}), 15.6 (C_{14}) ppm; HRMS [GC-MS, CI]: *m/z* calcd for $C_{15}H_{24}$ $[M]^+$: 204.1878, found: 204.1878.

The ^{13}C NMR data is in accordance with the literature.[S1] MS data of **43** was also compared to in house library [S1], confirming the NMR data.

Analytical data of **42**

$R_f = 0.79$ (*n*-heptane 100%); ^1H NMR (600 MHz, C_6D_6): $\delta = 5.22$ (s, 1H), 2.71 – 2.62 (m, 2H), 2.54 – 2.52 (m, 1H), 2.42 – 2.35 (m, 1H), 2.16 – 2.10 (m, 2H), 2.03 – 1.98 (m, 1H), 1.85 – 1.80 (m, 1H), 1.79 – 1.74 (m, 2H), 1.72 – 1.62 (m, 3H), 1.60 – 1.58 (m, 6H), 1.57 – 1.47 (m, 3H), 1.39 – 1.34 (m, 2H), 1.31 – 1.28 (m, 2H), 1.22 (s, 3H), 1.12 – 1.08 (m, 4H), 1.08 (s, 3H), 1.04 (s, 3H), 1.03 (s, 3H), 0.73 (dd, $J = 12.6$ Hz, 11.1 Hz, 1H) ppm; ^{13}C NMR (151 MHz, C_6D_6): $\delta = 137.1$ (C_2), 127.5 (C_3), 48.9 (C_{10}), 45.0 (C_4), 44.7 (C_1), 39.6 (C_8), 38.2 (C_7), 38.1 (C_9), 37.1 (C_{11}), 30.4 ($C_{12/15}$), 29.7 (C_{14}), 29.4 ($C_{12/15}$), 28.2 (C_6), 25.1 (C_5), 18.1 (C_{13}) ppm; HRMS [GC-MS, CI]: *m/z* calcd for $C_{15}H_{24}$ $[M]^+$: 204.1878, found: 204.1878.

The literature for sterpurene (**42**) is given for $CDCl_3$ instead of C_6D_6. [S15] Along with our own structure analysis (as displayed below), **42** can be determined as the second product beside pentalenene (**43**).

Analytical data of **44**

$R_f = 0.52$ (*n*-heptane:MTBE, 5:1); ^1H NMR (600 MHz, C_6D_6): $\delta = 2.47$ – 2.39 (m, 1H, H_9), 2.18 (dt, $J = 7.9$ Hz, 10.9 Hz, 1H, H_2), 1.88 – 1.80 (m, 3H, H_4, H_5, H_6), 1.71 (t, $J = 11.5$ Hz, 1H, H_1), 1.66 (ddd, $J = 11.9$ Hz, 7.5 Hz, 2.1 Hz, 1H, H_{10}), 1.56 – 1.52 (m, 2H, H_5, H_8), 1.43 – 1.40 (m, 2H, H_4, H_8), 1.37 (ddd, $J = 12.0$ Hz, 7.8 Hz, 2.1 Hz, 1H, H_1), 1.22 – 1.18 (m, 1H, H_{10}), 1.18 (s, 3H, H_{14}), 1.15 (s, 3H, $H_{12/15}$), 0.99 (s, 3H, $H_{12/15}$), 0.86 (s, 3H, H_{13}) ppm; ^{13}C NMR (151 MHz, C_6D_6): $\delta = 74.3$ (C_7), 50.9 (C_6), 50.4 (C_{10}), 41.6 (C_2), 40.6 (C_1), 40.3 (C_8), 38.2 (C_{11}), 36.9 (C_3), 34.4 (C_9), 32.2 (C_{14}), 29.5 ($C_{12/15}$), 27.9 (C_5), 27.6 ($C_{12/15}$), 27.1 (C_{13}), 16.9 (C_4) ppm; HRMS [GC-MS, CI]: *m/z* calcd for $C_{15}H_{26}O$ $[M]^+$: 222.1984, found: 222.1987.

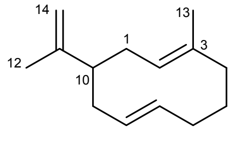

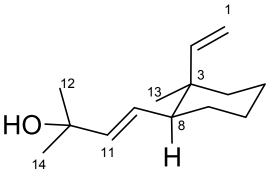

40
$C_{14}H_{22}$
190.33

41
$C_{14}H_{24}O$
208.35

FPP derivative **17** (653 mg, 1.57 mmol, 1.00 eq.) was dissolved in an aq. NH_4HCO_3 solution (0.05 M, 83 mL). The reaction was performed in eight 100 mL batch. The following procedure will be described for one 100 mL batch. Tween20® (20 µL) and PPase (1 µL) were dissolved in HEPES buffer (88 – 90 mL). FPP derivative **17** (1 mL) and Omp7 enzyme solution (2.98 mg/mL, 1.67 mL, in two batches the entire amount was added in the first place using 3.34 mL) were added, and the reaction was started by the addition of $MgCl_2$ (2 M, 500 µL). The reaction was stirred at 37 °C and 100 rpm and every 30 min more FPP derivative **17** (1 mL) was added to a total volume of 8 mL. After 2 h another batch of Omp7 enzyme solution (1.67 mL, one got 0.67 mL) was added. The reaction was then allowed to stir at 37 °C and 100 rpm o/n, before adding *n*-pentane. The phases were separated and the aqueous phase was extracted with *n*-pentane (3x). The combined organic phases were dried over $MgSO_4 \cdot H_2O$, filtered and the solvent was carefully removed *in vacuo*. The crude product was purified by column chromatography (*n*-pentane 100%, *n*-pentane:Et_2O 40:1 → 30:1 → 20:1 → 10:1 → 7.5:1). **40** (9 mg) was obtained as a colorless oil. Prep-GC of mixed fractions allowed isolation of **41**. GC-MS and NMR measurements indicate heat induced side reaction, likely elimination of water.

Analytical data of **40**

R_f = 0.62 (*n*-heptane 100%); ^{1}H NMR (500 MHz, C_6D_6): δ = 5.30 – 5.20 (m, 2H, H_7,H_8), 5.15 – 5.12 (m, 1H, H_2), 4.80 – 4.79 (m, 1H, H_{14}), 4.76 – 4.74 (m, 1H, H_{14}), 2.44 – 2.25 (m, 4H, H_1, H_4, H_6, H_9), 2.23 – 2.15 (m, 3H, H_1, H_4, H_6), 2.14 – 2.05 (m, 2H, H_9, H_{10}), 1.64 – 1.63 (m, 3H, H_{12}), 1.51 (m, 3H, H_{13}), 1.38 – 1.29 (m, 1H, H_5), 1.21 – 1.15 (m, 1H, H_5) ppm; ^{13}C NMR (126 MHz, C_6D_6): δ = 151.1 (C_{11}), 133.1 (C_8), 132.3 (C_3), 129.8 (C_2), 129.0 (C_7), 109.2 (C_{14}), 45.5 (C_{10}), 42.1 (C_4), 40.0 (C_9), 35.5 (C_6), 35.0 (C_1), 20.9 (C_{12}), 20.8 (C_5), 17.1 (C_{13}) ppm; HRMS [GC-MS, CI]: *m/z* calcd for $C_{14}H_{22}$ $[M]^+$: 190.1722, found: 190.1725.

Analtyical data of **41**

^{1}H NMR (600 MHz, C_6D_6): δ = 6.10 (dd, J = 17.6 Hz, 11.0 Hz, 1H, H_2), 5.59 (dd, J = 15.6 Hz, 8.6 Hz, 1H, H_9), 5.50 (d, J = 15.5 Hz, 1H, H_{10}), 5.10 (dd, J = 11.0 Hz, 1.7 Hz, 1H, H_1), 5.00 (dd, J = 17.6 Hz, 1.7 Hz, 1H, H_1), 1.73 (ddd, J = 11.8 Hz, 8.4 Hz, 3.5 Hz, 1H, H_8), 1.68 – 1.64 (m, 2H, H_4, H_6), 1.55 – 1.45 (m, 2H, H_5, H_7), 1.44 – 1.35 (m, 2H, H_5, H_7), 1.25 – 1.15 (m, 8H, H_4, H_6, H_{12}, H_{14}), 1.01 (s, 3H, H_{13}) ppm; ^{13}C NMR (151 MHz, C_6D_6): δ = 142.6 (C_2), 139.6 (C_{10}), 128.5 (C_9), 113.4 (C_1), 70.3 (C_{11}), 50.7 (C_8), 39.6 (C_4), 39.5 (C_3), 30.3 (C_{12}), 30.3 (C_{14}), 29.4 (C_7), 28.2 (C_{13}), 26.5 (C_6), 22.5 (C_5) ppm; HRMS [GC-MS, EI]: *m/z* calcd for $C_{14}H_{22}$ $[M-H_2O]^+$: 190.1722, found: 190.1729.

1.3 Microbiological Methods and Biotransformation

1.3.1 Procedure A

Heterologous protein expression and cell lysis *via* ultrasound

In order to cultivate the *E.coli* BL21 (DE3) cells, carrying the required plasmids, a seed culture (50 µL) was incubated with kanamycin (50 mg/mL, 3 µL) in LB-media (3 mL) for approximately 4.5 h at 37 °C and 200 rpm. Alternatively, a seed culture (5 µL) can be incubated with kanamycin (50 mg/mL, 5 µL) in LB-Media (5 mL) at 37 °C and 180/200 rpm o/n. From this pre-culture (1 mL) a main culture was created by incubation with kanamycin (50 mg/mL, 50 µL) in 2-TY media (50 mL) at 37 °C and 200 rpm until the culture reached an OD_{600} value of a 0.4 to 0.8. To initiate the protein expression IPTG (1 M, 25 µL or 50 µL) was added to the culture that was stirred at 16 °C and 180 rpm for approximately 22 h. After centrifugation, the cell pellets were stored at −20 °C or used immediately for cell lysis. Cells were resuspended in lysis buffer (20 mL) at 0 °C and lysed by ultrasonication (10 min, 45% amplitude, 4 s ultrasound to 6 s pause). The resulting solution was centrifuged (4 °C, 20 min, 10000 x*g*) to give the crude enzyme solution.

Immobilized metal-affinity chromatography

For conditioning the column was rinsed with water (10x the column volume) and lysis buffer (5x column volume). The lysate was loaded onto the column (2x) and eluted with Ni-NTA buffers (5 mL each) with increasing imidazole concentrations (25 mM, 50 mM, 100 mM, 250 mM, 500 mM). During this time the solutions were cooled at 0 °C. The fractions were analysed using a qualitative Brentford assay and those fractions containing protein were united and concentrated by centrifugation (4 °C, 4500 rpm).

Buffer exchange

To perform the buffer exchange the column was rinsed with water (10x column volume) and HEPES buffer (5x column Volume). The protein solutions were loaded onto the column and eluted with HEPES buffer (5 mL). After centrifugation (4 °C, 4500 rpm) the solutions can be used or stored as a mixture of water and glycerol (1/1) between −70 °C and −80 °C.

Concentration measurement

Concentrations were determined by measuring the absorption ($\lambda = 280$ nm) of the purified protein solutions, using the extinction coefficient for reduced cysteine side chains.

***In-vitro* biotransformation (analytical scale)**

Screening for new biotransformation products was performed in a reaction scale of 500 µL containing the corresponding enzyme (50 µg), the substrates (1.5 µL, 50 mM) and a $MgCl_2$ solution (1.25 µL, 2 M). In parallel also negative (without substrate or in the absence of enzymes) as well as positive control experiments were performed (using FPP (**1**)) under analogous conditions. Reactions using FPP derivatives were carried out in HEPES buffer (pH = 7.5) at 37 °C and 100 rpm for at least 60 min. Positive controls using FPP (**1**) were performed at 30 °C and 100 rpm. In order to extract the products *n*-hexane was added and the phases were separated by centrifugation (3000 rpm, 6 min, 4 °C). The hexane extract was used

for GC-MS analysis. Screening of Omp7 variants with FPP and its analogues was performed at 30 °C and 200 rpm.

1.3.2 Procedure B

Heterologous protein expression and cell lysis *via* ultrasound

In order to cultivate the *E.coli* BL21 (DE3) cells, carrying the required plasmids, a seed culture (10 µL) was incubated with kanamycin (50 mg/mL, 5 µL) in LB-media (10 mL) at 37 °C and 200 rpm o/n. From this pre-culture (10x 1 mL) a main culture was created by incubation with kanamycin (50 mg/mL, 50 µL) in 2-TY media (10x 1 L) at 37 °C and 200 rpm until the culture reached an OD_{600} value of a 0.4 to 0.8. To initiate the protein expression IPTG (1 M, 1 mL) was added to the culture that was stirred at 16 °C and 180 rpm for approximately 22 h. After centrifugation, the cell pellets were combined and either stored at −70 °C or used immediately for cell lysis. Cells were resuspended in lysis buffer (100 mL) at 0 °C and lysed by continuous flow cell disruption. The resulting solution was centrifuged (4 °C, 40 min, 20000 x*g*) to give the crude enzyme solution.

Immobilized metal-affinity chromatography

For immobilized metal-affinity purification an ÄKTA pure™ FPLC with HisTrap HP 5 mL column conditioned using ÄKTA lysis buffer (5 CV) containing imidazole (20 mM) at 0 °C. The enzyme was loaded onto the column and washed with ÄKTA lysis buffer (20 CV). The enzyme was eluted using ÄKTA lysis buffer containing a higher imidazole concentration (300 mM). The fractions were analyzed by UV-Vis spectrometry (280 nm), those fractions containing protein were united.

Buffer exchange

Buffer exchange was performed on an ÄKTA Go™ FPLC system using Superdex S200 16/600 conditioned with HEPES buffer (1.2 CV) at 0 °C. The protein solution (10 mL) was loaded onto the column and eluted with HEPES buffer (1.6 CV). The fractions were analyzed by UV-Vis spectrometry (280 nm), fractions containing protein were united and either directly used or cooled using liquid N_2 and stored at -70 °C.

Concentration measurement

Concentrations were determined by measuring the absorption (λ = 280 nm) of the purified protein solutions, using the extinction coefficient for reduced cysteine side chains.

***In-vitro* biotransformation (analytical scale)**

Screening for new biotransformation products was performed in a reaction scale of 500 µL containing the corresponding enzyme (50 µg for Pts and JeSTS4, 100 µg for LphTPS and PaTPS), the FPP-derivative (1.5 µL, 50 mM) and a $MgCl_2$ solution (1.25 µL, 2 M). In parallel also negative (without FPP derivative or in the absence of enzymes) as well as positive control experiments were performed (using FPP (**1**)) under analogous conditions. Reactions were carried out in HEPES buffer (pH = 7.5) for 30 min at 30 °C. The aqueous phase was extracted using *n*-hexane (100 µL) (3000 rpm, 8 min, 4 °C). The extract (50 µL) was used for GC-MS analysis.

1.3.3 Procedure for point mutations

E. coli stocks used for transformation and expression: *E.coli* Top10, *E.coli* BL21 (DE3), *E. coli* Rosetta (DE3).

Primer sequences used in this study were obtained from commercial supply (sigma-aldrich). Primer sequences are given in 5' → 3' direction.

F81W forward primer: CTGATGAACATGTTC**TGG**GTGTTCGATGAGTACAG

F81W reverse primer: CTGTACTCATCGAACAC**CCA**GAACATGTTCATCAG

Y172F forward primer: CGGTGGATGAG**TTT**CTGGAAGTGCGC

Y172F reverse primer: GCGCACTTCCAG**AAA**CTCATCCACCG

Y227F forward primer: GCCAGCTACAAC**TTT**GAACAAGCCCGC

Y227F reverse primer: GCGGGCTTGTTC**AAA**GTTGTAGCTGGC

Primer sequences for DNA sequencing: T7 primer

Components of PCR for Y227F mutant

Phusion® High-Fidelity DNA Polymerase, total volume of 250 µL, gradient PCR 8x25 µL

5x Phusion HF or GC buffer	50 µL
10 mM dNTPs	5 µL
10 µM Forward primer	12.5 µL
10 µM Reverse primer	12.5 µL
Phusion DNA polymerase	2.5 µL
DNA Template	0.3 µL DNA
Nuclease free H_2O	167.2 µL

Components of PCR for F81W and Y172F mutant

Q5® High-Fidelity 2X Master Mix, total volume of 250 µL, gradient PCR 8x25 µL

10 µM Forward primer	12.5 µL
10 µM Reverse Primer	12.5 µL
Template DNA	1 µL
Q5 High-Fidelity 2X Master Mix	125 µL
Nuclease free H_2O	99 µL

Temperature program for gradient PCR

Run volume 25 µL per Annealing Temperature → 8 x 25 µL

Initial denaturation	at 98 °C for 30 s
Denaturation	at 98 °C for 15 s
Annealing	at T profile (55, 56.5, 58.9, 62.5, 66.9, 71, 73.5, 75) °C for 30 s
Elongation	at 72 °C for 3:11 min
Final elongation	at 72 °C for 5 min
Infinite hold	at 4 °C
Number of cycles:	25

Plasmid isolation

Solution 1: Glucose 50 mM, Tris 25 mM, EDTA 10 mM, pH = 8.0

Solution 2: NaOH 200 mM, SDS 1%

Solution 3: Kaliumacetat 3 mol/L, pH = 5.5

DNA Sequencing

Omp7 F81W mutant (T7 primer, forward primer marked in red):

CTAGAATAATTTTGTTTAACTTTAAGAAGGAGATATACCATGGGCAGCAGCCATC
ATCATCATCATCACAGCAGCGGCCATATGCCAGAAACGTTCTACCTCCCGGATTG
CCTCGCCAACTGGAAATGGAAGCGCGCGCTGAATCCAAACTACCCGGAAGTGAA
AGCCGCGAGCAGCGAGTGGCTGCGTAGCTTCAAAGCCTTTCCGCCGAAAGCGCA
AGAAGCCTACGATCGCTGCGATTTCAATCTGCTGGCCAGTCTGGCCTATCCACTG
GCGGATAAGGATGGCCTCCGCACGGGTTGCGATCTGATGAACATGTTCTGGGTGT
TCGATGAGTACAGCGATGTGGCGCACGAAAGCGAGGTGCAAGTTCAAGCCGATA
TCATTATGGATGCCCTCCGCAACCCACACAAGCCACGCCCAGTTGGTGAATGGGT
TGGCGGCGAGGTTACCCGCCAGTTCTGGGAACTGGCGATTAAAACCGCCAGCCCA
CAGAGCCAAAAGCGCTTCATCGAGACGTTCGACACGTACACCAAGAGTGTTGTTC
AGCAAGCCGCGGATCGTACGCAGCATTACGTTCGTACGGTGGATGAGTATCTGGA
AGTGCGCCGCGATACCATCGGTGCCAAACCGAGCTTCGCGATTCTGGAACTGACG
ATGGACATCCCAGACGAAGTGATCCACCATCCAACGATCGAACGTCTGGCGATTC
TGGCCATCGACATGATTCTGCTGGGCAACGACACGGCCAGCTACAACTATGAACA
AGCCCGCGGCGACGACAACCACAACATGGTGACCATCGTGATGCATCAGTACAA
GACGGATATCCAAGGCGCGCTGAGCTGGATCGAAAAGTACCACAAGGAGCTCGA
GGAGGAGTTCATGCAGCTCTACAACAGTCTGCCAAAGTGGGGCGGTCAGATTGA
CGTGGATATCGCCCGCTATGTTGATGGTCTGGGCAATTGGGTGCGCGCCAGCGAT
CAGTGGGGTTTCGAGAGCGAACGCTACTTCGGCACCAAAGCCCCAGAAATCCAG
AAGACGCGCTGGGTGACGCTGATGCCAAAAAAACGTGCGGAGGGCGTGGG

Omp7 F81W mutant (T7 term primer, reverse primer marked in red):

TTGTTAGCAGCCGGATCTCAGTGGTGGTGGTGGTGGTGCTCGAGTGCGGCCGCAA
GCTTAGAGTTCGCTGATATCCACGATCTCTGGACCCACGCCCTCCGCACGTTTTTT
TGGCATCAGCGTCACCCAGCGCGTCTTCTGGATTTCTGGGGCTTTGGTGCCGAAG
TAGCGTTCGCTCTCGAAACCCCACTGATCGCTGGCGCGCACCCAATTGCCCAGAC
CATCAACATAGCGGGCGATATCCACGTCAATCTGACCGCCCCACTTTGGCAGACT
GTTGTAGAGCTGCATGAACTCCTCCTCGAGCTCCTTGTGGTACTTTTCGATCCAGC
TCAGCGCGCCTTGGATATCCGTCTTGTACTGATGCATCACGATGGTCACCATGTTG
TGGTTGTCGTCGCCGCGGGCTTGTTCATAGTTGTAGCTGGCCGTGTCGTTGCCCAG
CAGAATCATGTCGATGGCCAGAATCGCCAGACGTTCGATCGTTGGATGGTGGATC
ACTTCGTCTGGGATGTCCATCGTCAGTTCCAGAATCGCGAAGCTCGGTTTGGCAC
CGATGGTATCGCGGCGCACTTCCAGATACTCATCCACCGTACGAACGTAATGCTG
CGTACGATCCGCGGCTTGCTGAACAACACTCTTGGTGTACGTGTCGAACGTCTCG
ATGAAGCGCTTTTGGCTCTGTGGGCTGGCGGTTTTAATCGCCAGTTCCCAGAACT
GGCGGGTAACCTCGCCGCCAACCCATTCACCAACTGGGCGTGGCTTGTGTGGGTT
GCGGAGGGCATCCATAATGATATCGGCTTGAACTTGCACCTCGCTTTCGTGCGCC
ACATCGCTGTACTCATCGAACACCCAGAACATGTTCATCAGATCGCAACCCGTGC
GGAGGCCATCCTTATCCGCCAGTGGATAGGCCAGACTGGCCAGCAGATTGAAATC
GCAGCGATCGTAGGCTTCTTGCGCTTTCGGCGGAAAGGCTTTGAAGCTACGCAGC
CACTCGCTGCTCGCGGCTTTCACTTCCGGGTAGTTTGGATTCAGCGCGCGCTTC

Omp7 Y172F mutant (T7 primer, forward primer marked in orange):

AAGAAGGAGATATACCATGGGCAGCAGCCATCATCATCATCATCACAGCAGCGG
CCATATGCCAGAAACGTTCTACCTCCCGGATTGCCTCGCCAACTGGAAATGGAAG
CGCGCGCTGAATCCAAACTACCCGGAAGTGAAAGCCGCGAGCAGCGAGTGGCTG
CGTAGCTTCAAAGCCTTTCCGCCGAAAGCGCAAGAAGCCTACGATCGCTGCGATT
TCAATCTGCTGGCCAGTCTGGCCTATCCACTGGCGGATAAGGATGGCCTCCGCAC
GGGTTGCGATCTGATGAACATGTTCTTCGTGTTCGATGAGTACAGCGATGTGGCG
CACGAAAGCGAGGTGCAAGTTCAAGCCGATATCATTATGGATGCCCTCCGCAACC
CACACAAGCCACGCCCAGTTGGTGAATGGGTTGGCGGCGAGGTTACCCGCCAGTT
CTGGGAACTGGCGATTAAAACCGCCAGCCCACAGAGCCAAAAGCGCTTCATCGA
GACGTTCGACACGTACACCAAGAGTGTTGTTCAGCAAGCCGCGGATCGTACGCAG
CATTACGTTCGTACGGTGGATGAGTTTCTGGAAGTGCGCCGCGATACCATCGG

Omp7 Y172F mutant (T7 term primer, reverse primer marked in orange):

GCAGCCGGATCTCAGTGGTGGTGGTGGTGGTGCTCGAGTGCGGCCGCAAGCTTAG
AGTTCGCTGATATCCACGATCTCTGGACCCACGCCCTCCGCACGTTTTTTGGCAT
CAGCGTCACCCAGCGCGTCTTCTGGATTTCTGGGGCTTTGGTGCCGAAGTAGCGT
TCGCTCTCGAAACCCCACTGATCGCTGGCGCGCACCCAATTGCCCAGACCATCAA
CATAGCGGGCGATATCCACGTCAATCTGACCGCCCCACTTTGGCAGACTGTTGTA
GAGCTGCATGAACTCCTCCTCGAGCTCCTTGTGGTACTTTTCGATCCAGCTCAGCG
CGCCTTGGATATCCGTCTTGTACTGATGCATCACGATGGTCACCATGTTGTGGTTG
TCGTCGCCGCGGGCTTGTTCATAGTTGTAGCTGGCCGTGTCGTTGCCCAGCAGAA
TCATGTCGATGGCCAGAATCGCCAAACGTTCGATCGTTGGATGGTGGATCACTTC
GTCTGGGATGTCCATCGTCAGTTCCAGAATCGCGAAGCTCGGTTTGGCACCGATG
GTATCGCGGCGCACTTCCAGAAACTCATCCACCGTACGAACGTAATGCTGCGTAC
GATCCGCGGCTTGCTGAACAACACTCTTGGTGTACGTGTCGAACGTCTCGATGAA

GCGCTTTTGGATCTGTGGGCTGGCGGTTATAATCGCCACTTCCCAGAACTGGCGG
GTAACCTCGCCGCCAACCCATTCACCAACTG

Omp7 Y227F mutant (T7 primer, forward primer marked in blue):

NNNNTTCCCTCTAGAATAATTTTGTTTAACTTTAAGAAGGAGATATACCATGGGC
AGCAGCCATCATCATCATCATCACAGCAGCGGCCATATGCCAGAAACGTTCTACC
TCCCGGATTGCCTCGCCAACTGGAAATGGAAGCGCGCGCTGAATCCAAACTACCC
GGAAGTGAAAGCCGCGAGCAGCGAGTGGCTGCGTAGCTTCAAAGCCTTTCCGCC
GAAAGCGCAAGAAGCCTACGATCGCTGCGATTTCAATCTGCTGGCCAGTCTGGCC
TATCCACTGGCGGATAAGGATGGCCTCCGCACGGGTTGCGATCTGATGAACATGT
TCTTCGTGTTCGATGAGTACAGCGATGTGGCGCACGAAAGCGAGGTGCAAGTTCA
AGCCGATATCATTATGGATGCCCTCCGCAACCCACACAAGCCACGCCCAGTTGGT
GAATGGGTTGGCGGCGAGGTTACCCGCCAGTTCTGGGAACTGGCGATTAAAACC
GCCAGCCCACAGAGCCAAAAGCGCTTCATCGAGACGTTCGACACGTACACCAAG
AGTGTTGTTCAGCAAGCCGCGGATCGTACGCAGCATTACGTTCGTACGGTGGATG
AGTATCTGGAAGTGCGCCGCGATACCATCGGTGCCAAACCGAGCTTCGCGATTCT
GGAACTGACGATGGACATCCCAGACGAAGTGATCCACCATCCAACGATCGAACG
TCTGGCGATTCTGGCCATCGACATGATTCTGCTGGGCAACGACACGGCCAGCTAC
AACTTTGAACAAGCCCGCGGCGACGACAACCACAACATGGTGACCATCGTGATG
CATCAGTACAAGACGGATATCCAAGGCGCGCTGAGCTGGATCGAAAAGTACCAC
AAGGAGCTCGAGGAGGAGTTCATGCAGCTCTACAACAGTCTGCCAAAGTGGGGC
GGTCAGATTGACGTGGATATCGCCCGCTATGTTGATGGTCTGGGCAATTGGGTGC
GCGCCAGCGATCAGTGGGGTTTCGAGAGCGAACGCTACTTCGGCACCAAGCCCC
AGAAATCCAGAAGACGCGCTGGGTGACGCTGATGCCAAAAAACGTGCGGAGGGC
GTGGGTCCAGAGATCGTGGATTTCAGCGAACTCTAAGCTGGCGGCCNACTCGAGC
ACCCCACCACACCACTGAGATCCGGCTGCTAACAAGNCCCGAAAG

Omp7 Y227F mutant (T7term primer, reverse primer marked in blue):

NNNCTTCTTTCGGGCTTTGTTAGCAGCCGGATCTCAGTGGTGGTGGTGGTGGTGCT
CGAGTGCGGCCGCAAGCTTAGAGTTCGCTGATATCCACGATCTCTGGACCCACGC
CCTCCGCACGTTTTTTGGCATCAGCGTCACCCAGCGCGTCTTCTGGATTTCTGGG
GCTTTGGTGCCGAAGTAGCGTTCGCTCTCGAAACCCCACTGATCGCTGGCGCGCA
CCCAATTGCCCAGACCATCAACATAGCGGGCGATATCCACGTCAATCTGACCGCC
CCACTTTGGCAGACTGTTGTAGAGCTGCATGAACTCCTCCTCGAGCTCCTTGTGGT
ACTTTTCGATCCAGCTCAGCGCGCCTTGGATATCCGTCTTGTACTGATGCATCACG
ATGGTCACCATGTTGTGGTTGTCGTCGCCGCGGGCTTGTTCAAAGTTGTAGCTGGC
CGTGTCGTTGCCCAGCAGAATCATGTCGATGGCCAGAATCGCCAGACGTTCGATC
GTTGGATGGTGGATCACTTCGTCTGGGATGTCCATCGTCAGTTCCAGAATCGCGA
AGCTCGGTTTGGCACCGATGGTATCGCGGCGCACTTCCAGATACTCATCCACCGT
ACGAACGTAATGCTGCGTACGATCCGCGGCTTGCTGAACAACACTCTTGGTGTAC
GTGTCGAACGTCTCGATGAAGCGCTTTTGGCTCTGTGGGCTGGCGGTTTTAATCGC
CAGTTCCCAGAACTGGCGGGTAACCTCGCCGCCAACCCATTCACCAACTGGGCGT
GGCTTGTGTGGGTTGCGGAGGGCATCCATAATGATATCGGCTTGAACTTGCACCT
CGCTTTCGTGCGCCACATCGCTGTACTCATCGAACACGAAGAACATGTTCATCAG
ATCGCAACCCGTGCGGAGGCCATCCTTATCCGCCAGTGGATAGGCCAGACTGGCC
AGCAGATTGAAATCGCAGCGATCGTAGGCTTCTTGCGCTTTCGGCGGAAAGGCTT
TGAAGCTACGCAGCCACTCGCTGCTCGCGGCTTTCACTTCCGGGTAGTTTGGATTC

AGCGCGCGCTTCCATTTCCAGTTGGCGAGGCAATCCGGGAGGTAGAACGTTTCTG
GCATATGGCCGCTGCTGTGATGATGATGATGATGGCTGCTGCCCATGGAAT

SDS Pages

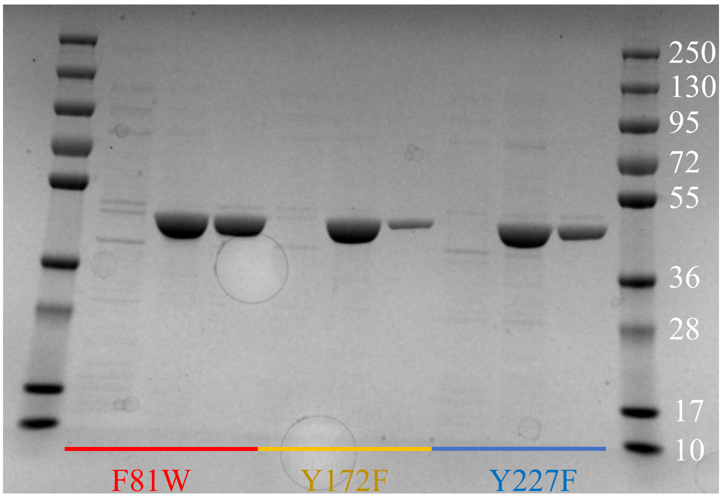

Figure S1. SDS Page for purification of Omp7 mutants. M = protein marker (Page Ruler™ Plus Prestained Protein Ladder), FT = Flow through of IMAC, W = 25 mM imidazole fraction, E = collected enzyme fraction (50 mM + 100 mM Imidazole).

Additionally, we performed HRMS measurements of our Omp7 variants. Protein masses were calculated without the methionine originated from the start codon. The plasmid carried no thrombin site anymore as analysed by sequencing.

Omp7 WT calculated mass: 41266 Da, found: 41265 Da

Omp7 F81W calculated mass: 41306 Da, found: 41308 Da

Omp7 Y172F calculated mass: 41250 Da, found: 41250 Da

Omp7 Y227F calculated mass: 41250 Da, found: 41248 Da

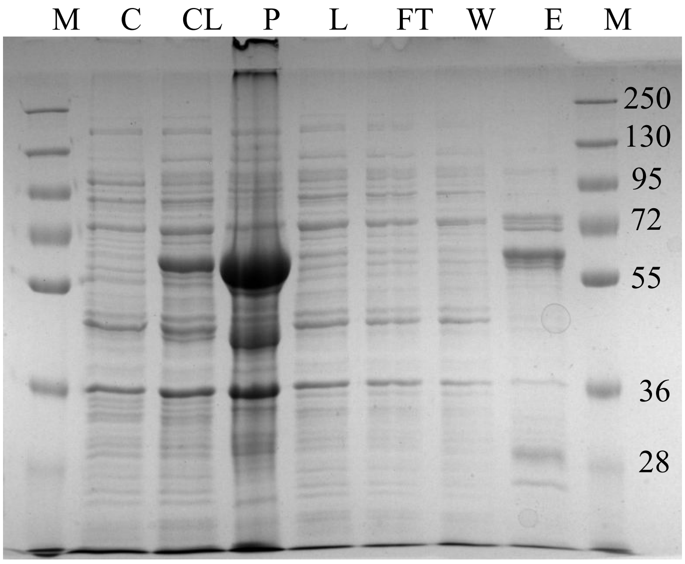

Figure S2. SDS Page for purification of PaTPS WT. M = protein marker (Page Ruler™ Plus Prestained Protein Ladder), C = uninduced control, CL = induced control, P = insoluble

fraction, L = soluble fraction, FT = Flow through of IMAC, W = 25 mM imidazole fraction, E = collected enzyme fraction (500 mM).

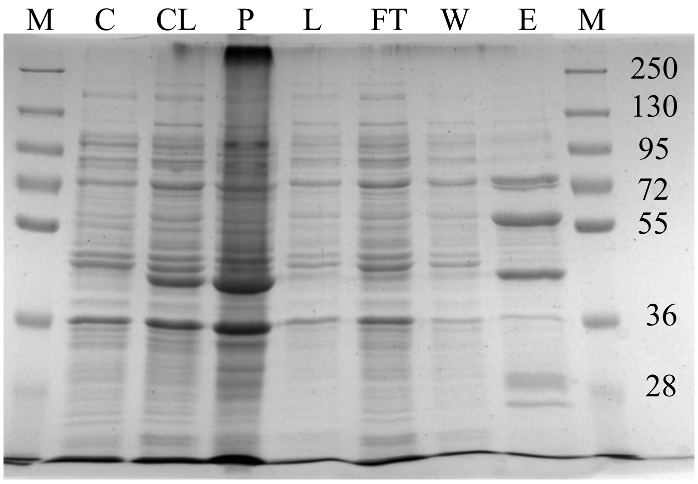

Figure S3. SDS Page for purification of JeSTS4 WT. M = protein marker (Page Ruler^{TM} Plus Prestained Protein Ladder), C = uninduced control, CL = induced control, P = insoluble fraction, L = soluble fraction, FT = Flow through of IMAC, W = 25 mM imidazole fraction, E = collected enzyme fraction (500 mM).

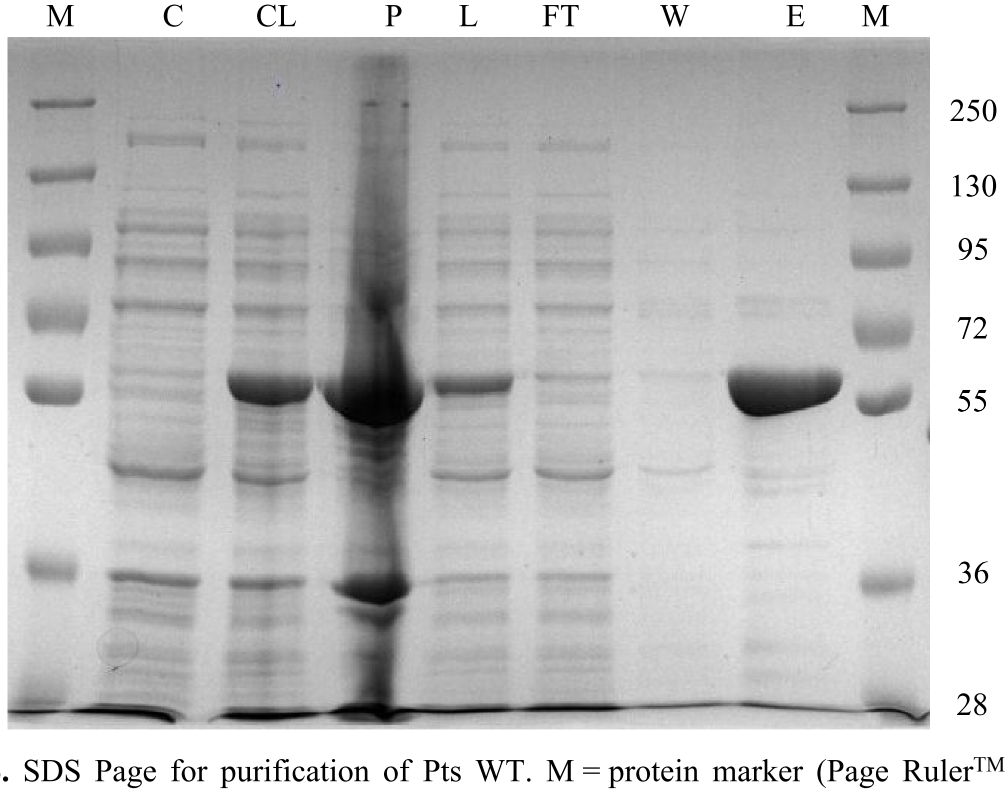

Figure S4. SDS Page for purification of Pts WT. M = protein marker (Page Ruler^{TM} Plus Prestained Protein Ladder), C = uninduced control, CL = induced control, P = insoluble fraction, L = soluble fraction, FT = Flow through of IMAC, W = 25 mM imidazole fraction, E = collected enzyme fraction (500 mM).

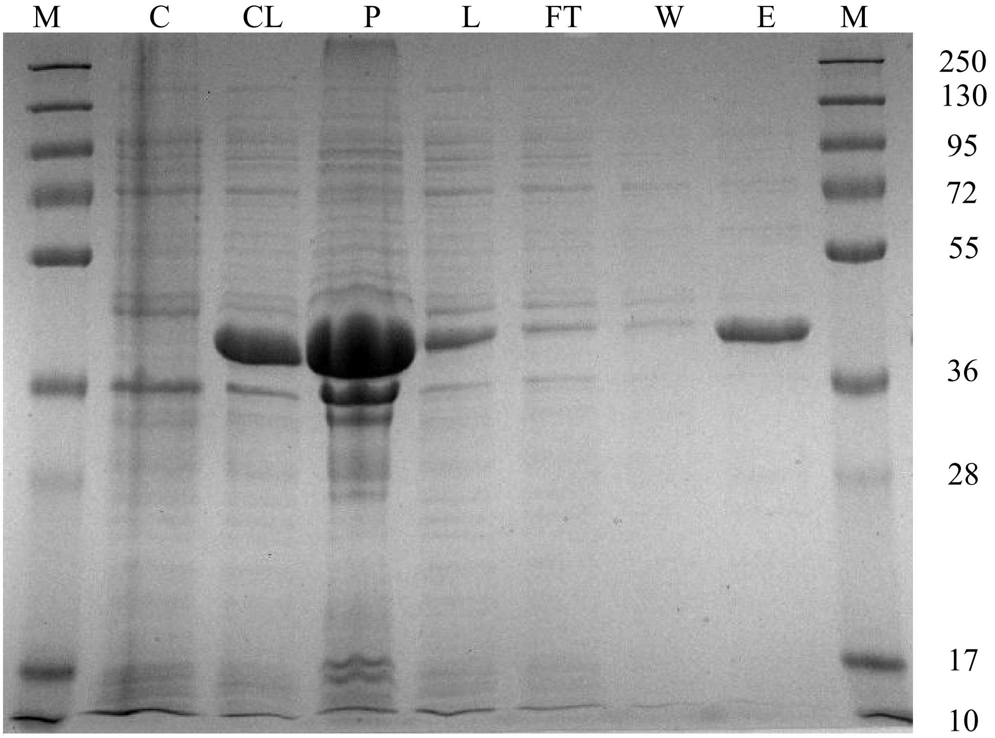

Figure S5. SDS Page for purification of LphTPS WT. M = protein marker (Page Ruler™ Plus Prestained Protein Ladder), C = uninduced control, CL = induced control, P = insoluble fraction, L = soluble fraction, FT = Flow through of IMAC, W = 25 mM imidazole fraction, E = collected enzyme fraction (500 mM).

1.4 GC-MS Data

1.4.1 Positive and Negative Controls

The different STSs were tested in an analytical scale with FPP (**1**) towards their general activity (positive control), without any substrate and the substrates without an enzyme (negative controls). The results are shown in **Schemes S2** – **S16** (results for Tps32 are shown in 1.4.9).

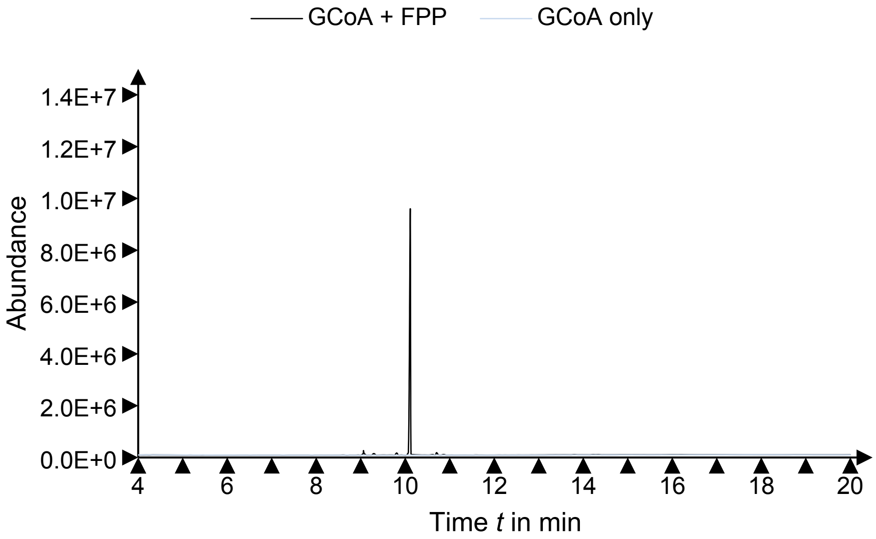

Scheme S2. GC chromatogram of analytical scale biotransformation of GCoA with FPP (**1**) (black) and without substrate (light blue).

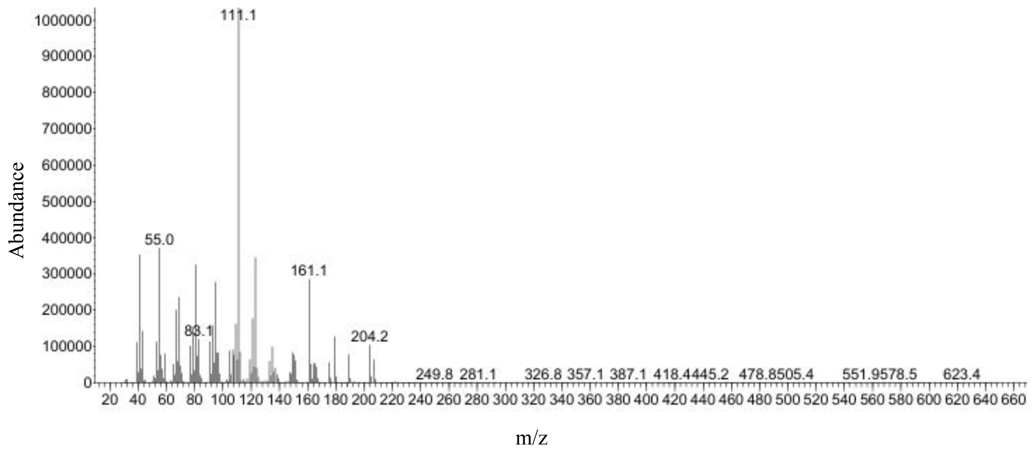

Scheme S3. MS data of analytical scale biotransformation of GCoA with FPP (**1**) (t_R = 10.020 to 10.061 min).

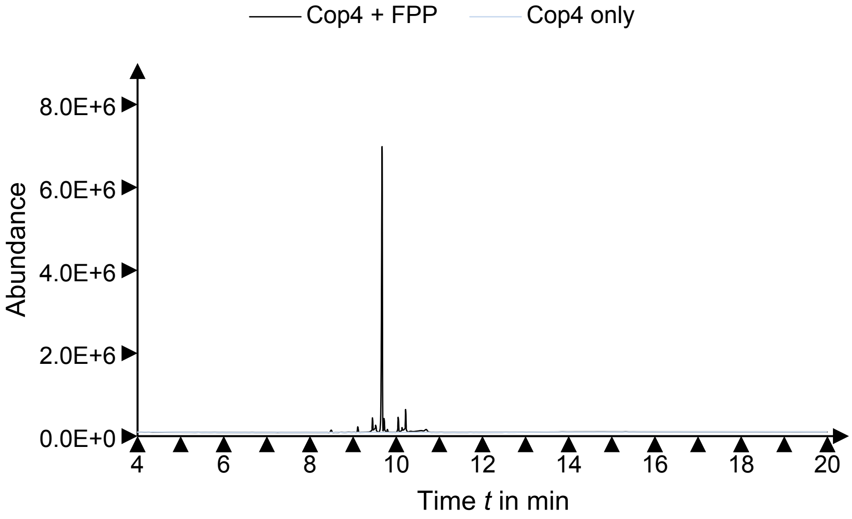

Scheme S4. GC chromatogram of analytical scale biotransformation of Cop4 with FPP (**1**) (black) and without substrate (light blue).

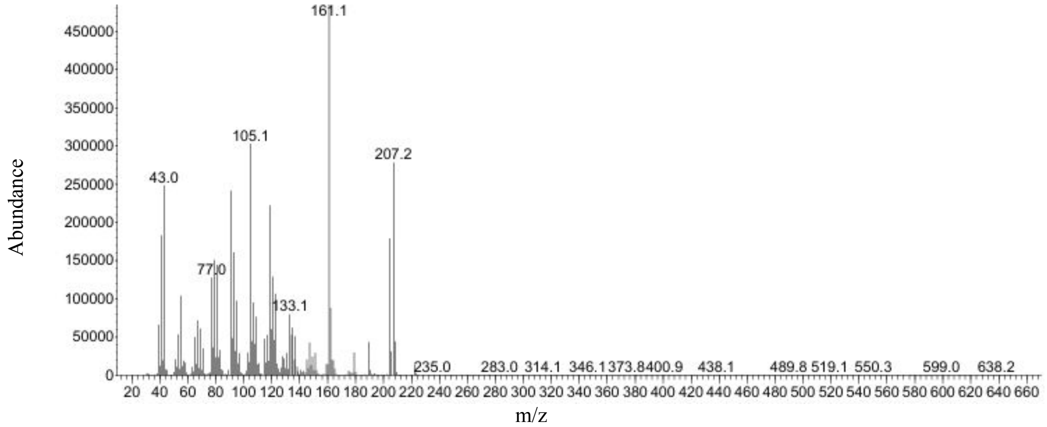

Scheme S5. MS data of analytical scale biotransformation of Cop4 with FPP (**1**) (t_R = 9.610 to 9.623 min).

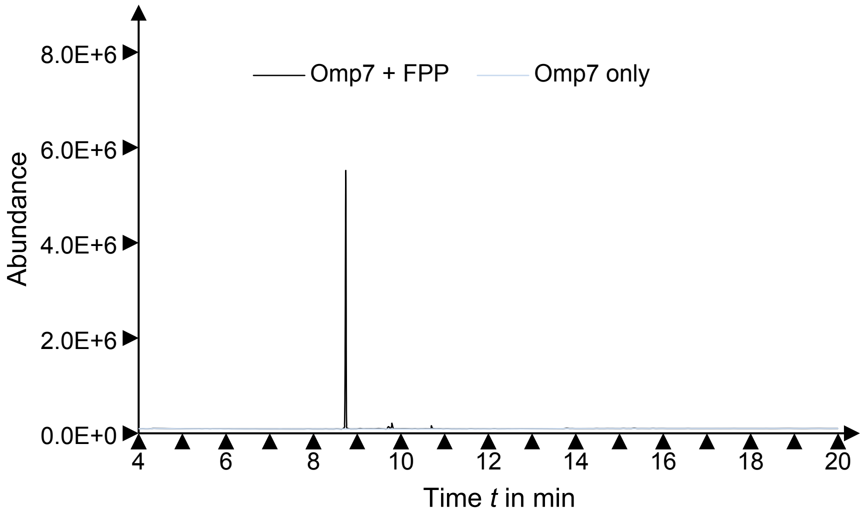

Scheme S6. GC chromatogram of analytical scale biotransformation of Omp7 with FPP (**1**) (black) and without substrate (light blue).

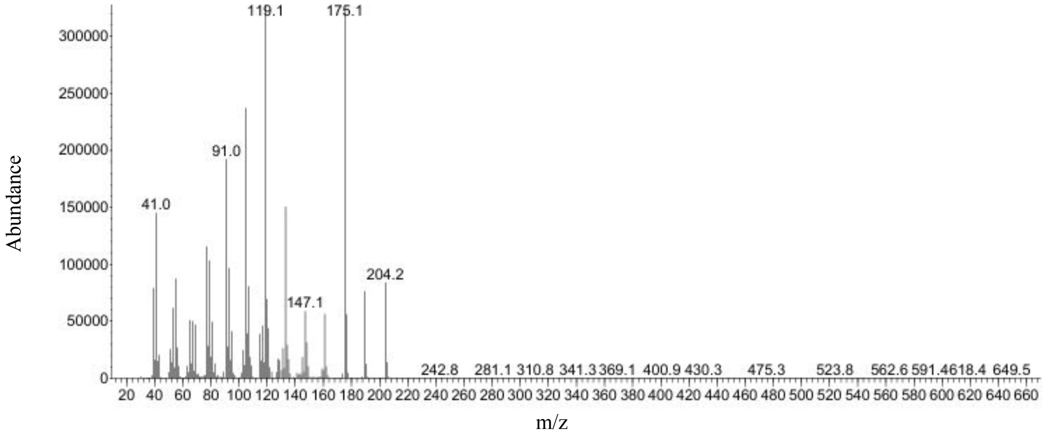

Scheme S7. MS data of analytical scale biotransformation of Omp7 with FPP (**1**) (t_R = 8.667 to 8.680 min).

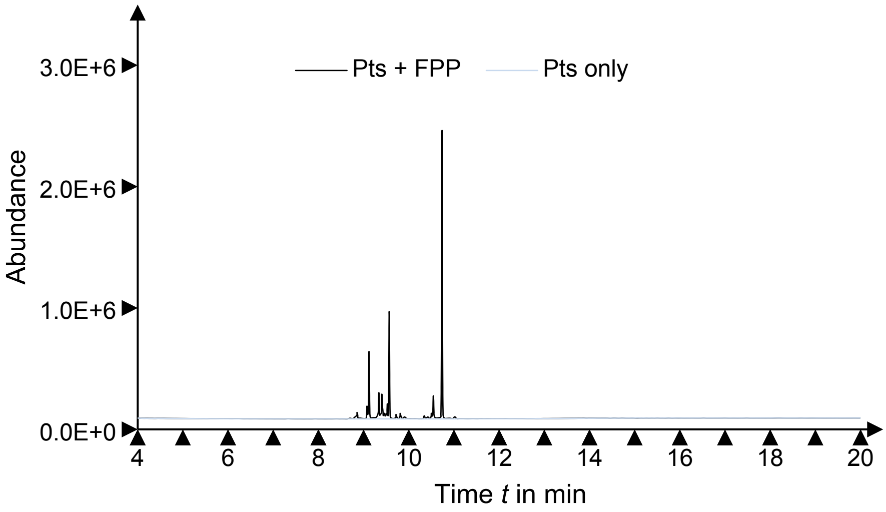

Scheme S8. GC chromatogram of analytical scale biotransformation of Pts with FPP (**1**) (black) and without substrate (light blue).

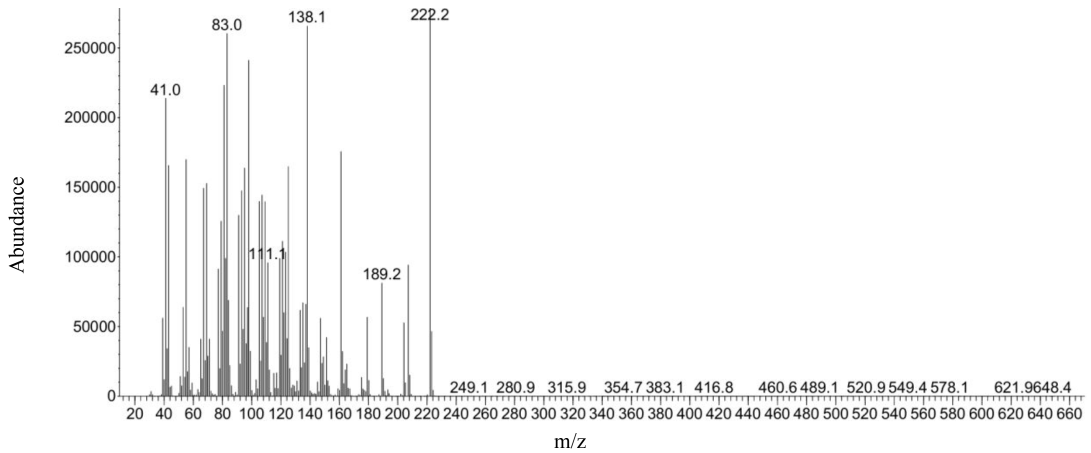

Scheme S9. MS data of analytical scale biotransformation of Pts with FPP (**1**) (t_R = 10.676 to 10.690 min).

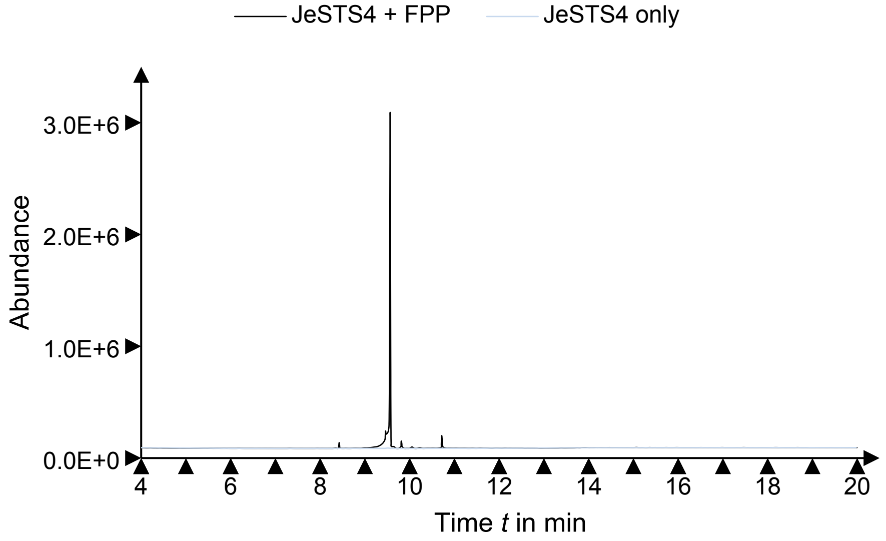

Scheme S10. GC chromatogram of analytical scale biotransformation of JeSTS4 with FPP (**1**) (black) and without substrate (light blue).

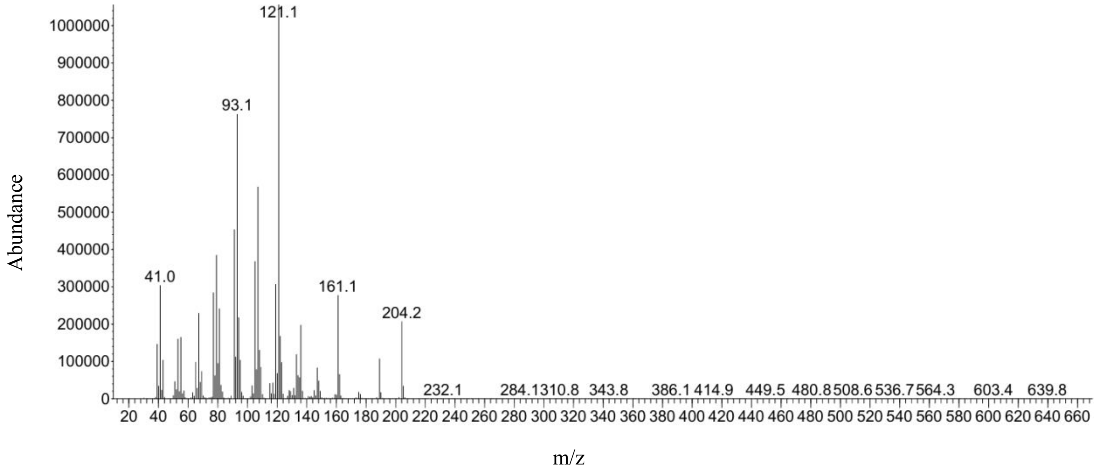

Scheme S11. MS data of analytical scale biotransformation of JeSTS with FPP (**1**) (t_R = 9.514 min).

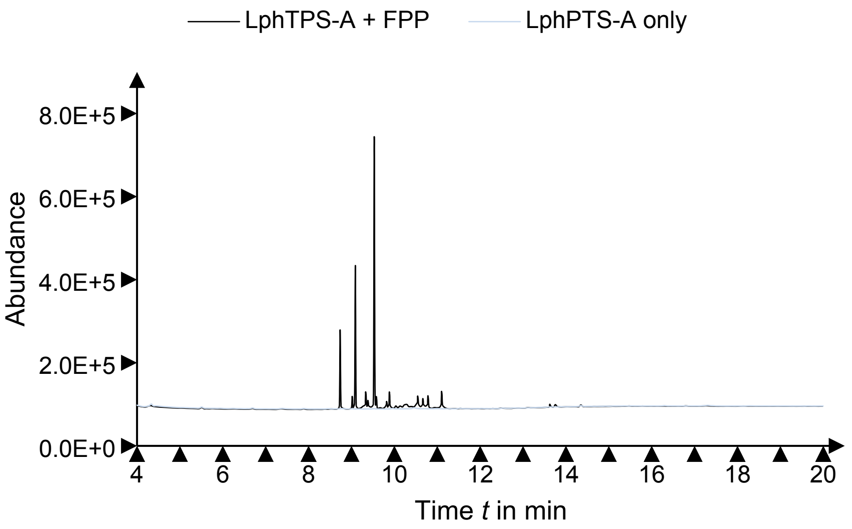

Scheme S12. GC chromatogram of analytical scale biotransformation of LphTPS with FPP (**1**) (black) and without substrate (light blue).

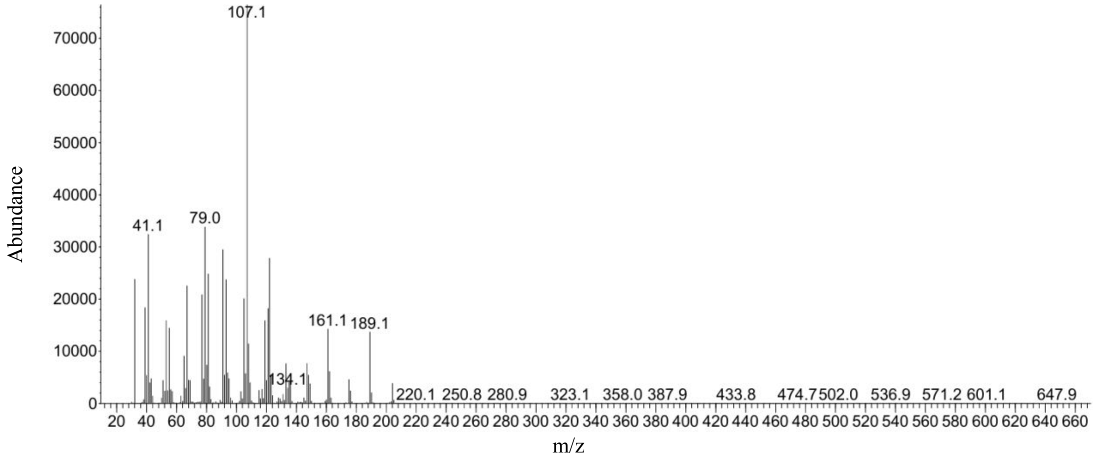

Scheme S13. MS data of analytical scale biotransformation of LphTPS with FPP (**1**) (t_R = 9.036 to 9.049 min).

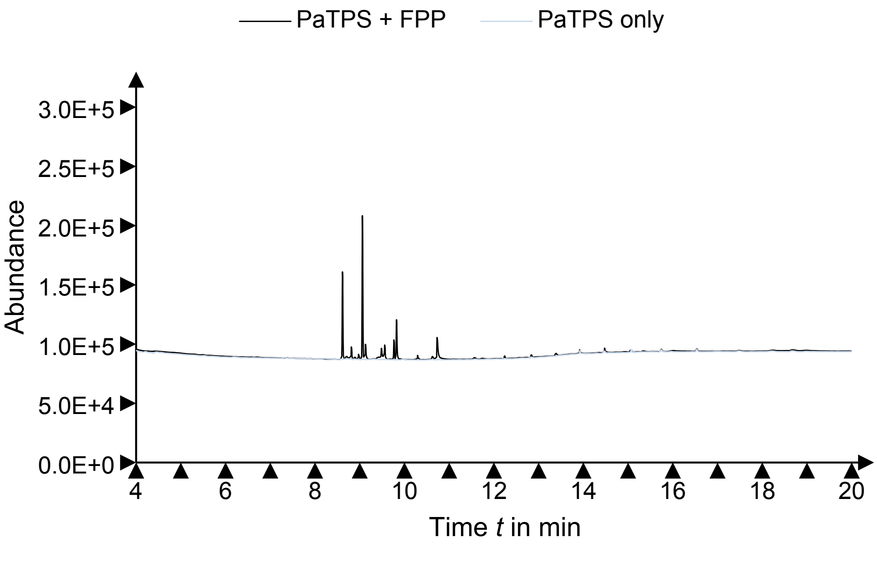

Scheme S14. GC chromatogram of analytical scale biotransformation of PaTPS with FPP (**1**) (black) and without substrate (light blue).

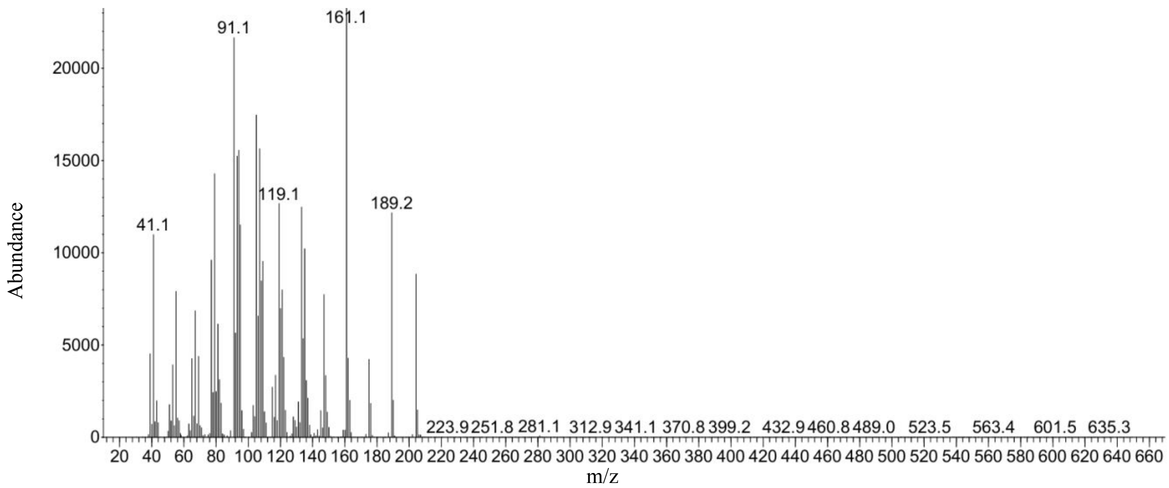

Scheme S15. MS data of analytical scale biotransformation of PaTPS with FPP (**1**) (t_R = 9.008 min).

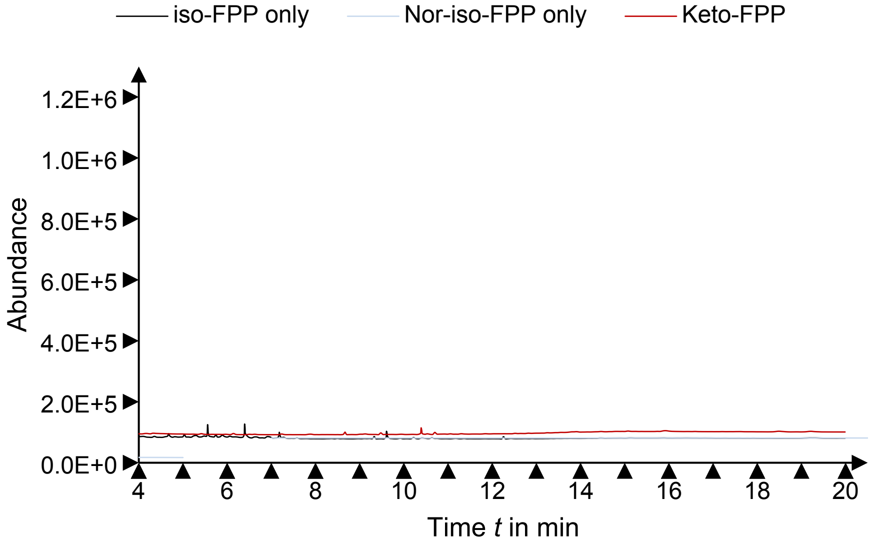

Scheme S16. GC chromatogram of analytical scale biotransformation of *iso*-FPP (**16**) (black) Nor-*iso*-FPP (**17**) (light blue) and keto-FPP (**15**) (red) without any enzyme.

1.4.2 Biotransformation of GCoA and Keto-FPP

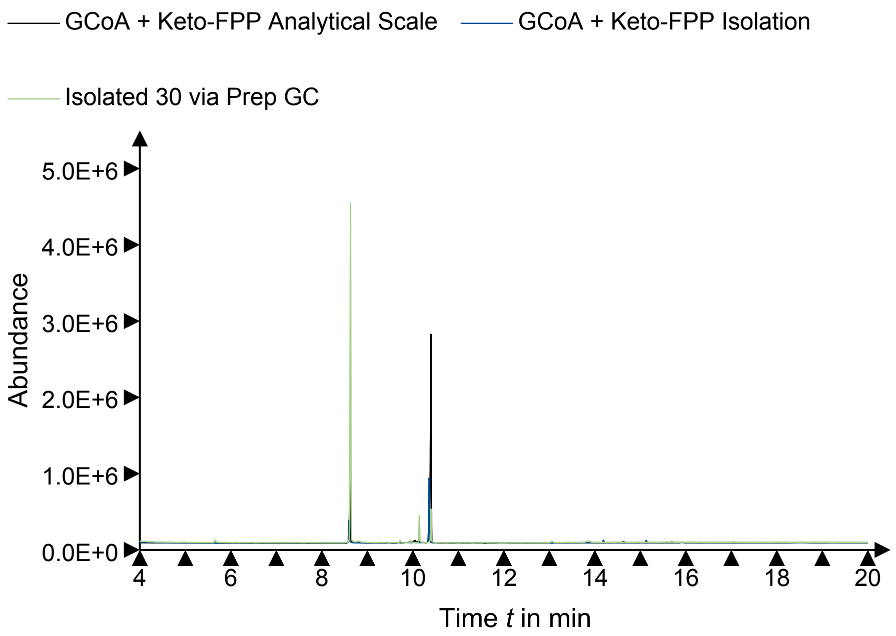

Scheme S17. GC chromatogram of analytical scale biotransformation of GCoA with Keto-FPP (**15**) in an analytical scale (black), for product isolation (blue) and isolated **30** from prep-GC (green).

Since the linear tertiary alcohol **31** forms **30** during GC, both products are seen. Preparative GC led to the isolation of **30**:**31** in a ration of appr. 4:1 (^{1}H-NMR).

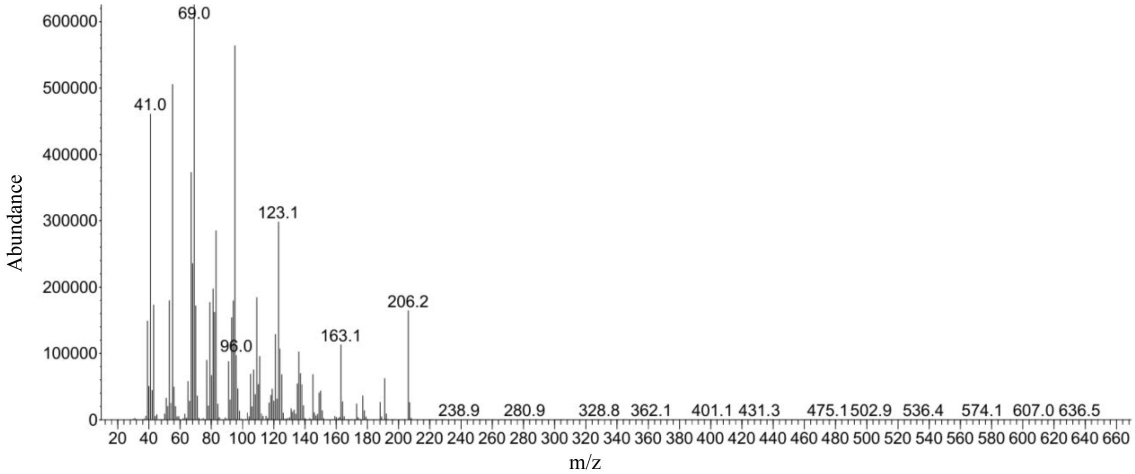

Scheme S18. MS data of **30** from biotransformation of GCoA with Keto-FPP (**15**) (t_R = 8.557 to 8.585 min).

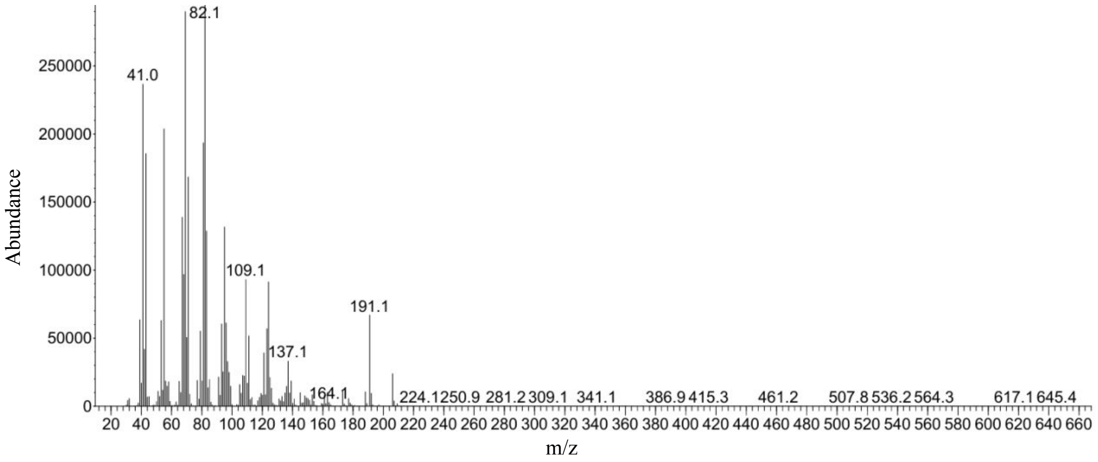

Scheme S19. MS data of **31** from biotransformation of GCoA with Keto-FPP (**15**) (t_R = 10.334 to 10.389 min).

1.4.3 Biotransformation of Cop4 and Keto-FPP

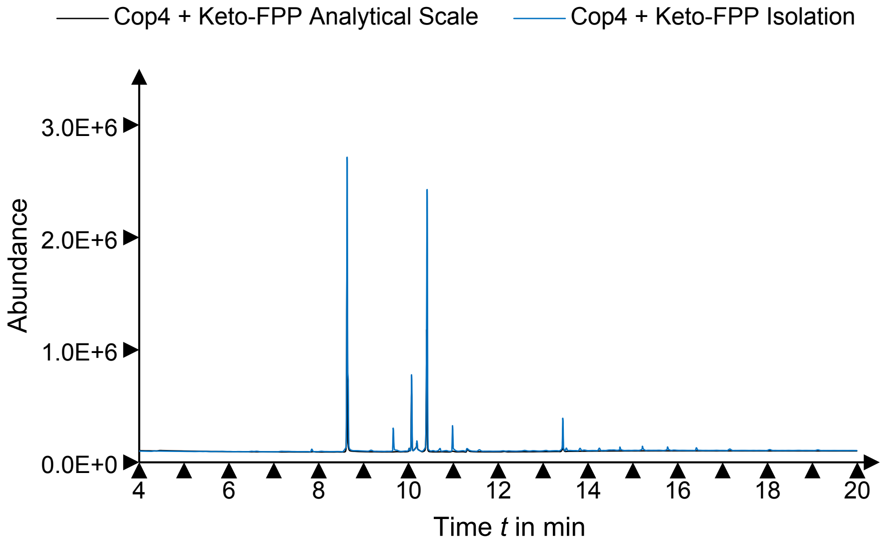

Scheme S20. GC chromatogram of analytical scale biotransformation of Cop4 with Keto-FPP (**15**) in an analytical scale (black) and for product isolation (blue).

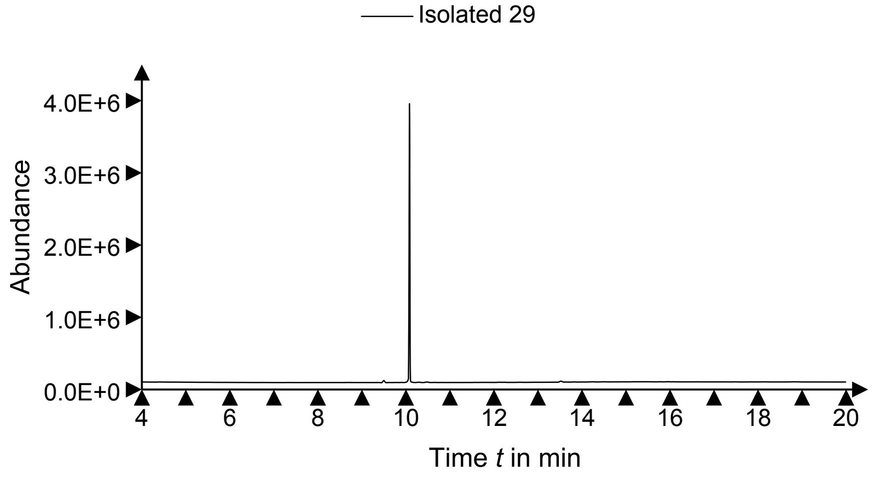

Scheme S21. GC chromatogram of isolated compound **29**.

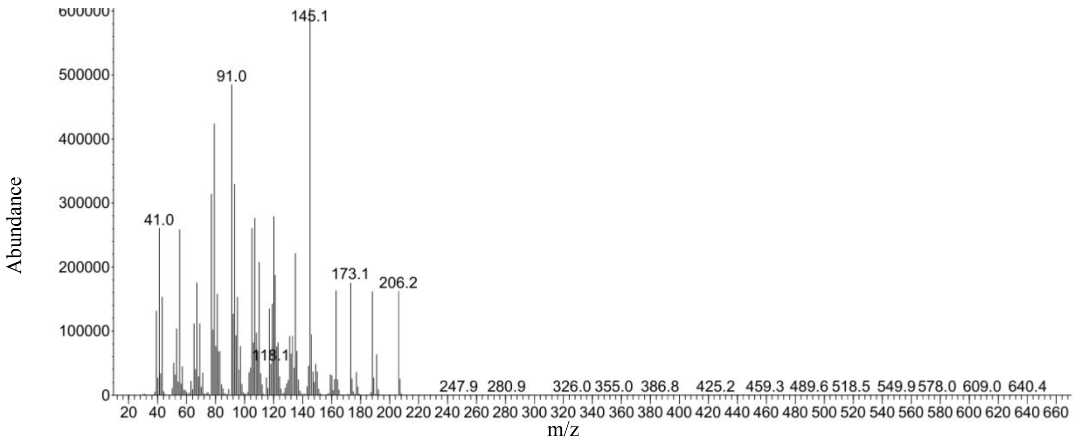

Scheme S22. MS data of **29** from biotransformation of Cop4 with Keto-FPP (**15**) (t_R = 10.020 to 10.034 min).

1.4.4 Biotransformation of Omp7 and Keto-FPP

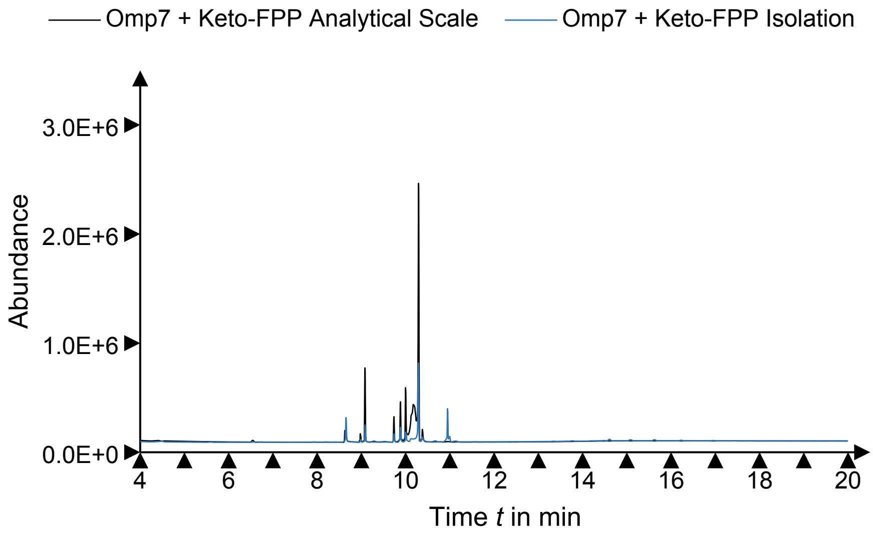

Scheme S23. GC chromatogram of analytical scale biotransformation of Omp7 with Keto-FPP (**15**) in an analytical scale (black) and for product isolation (blue).

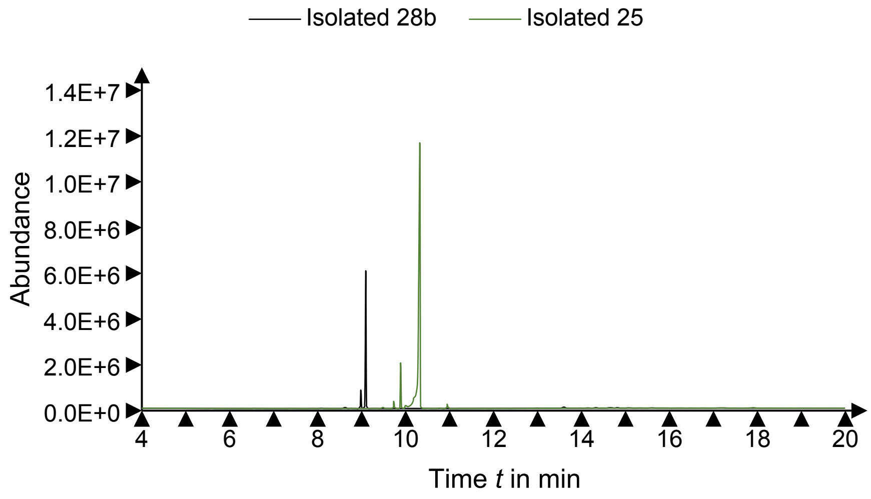

Scheme S24. GC chromatogram of isolated compounds **25** (green) and **28b** (black).

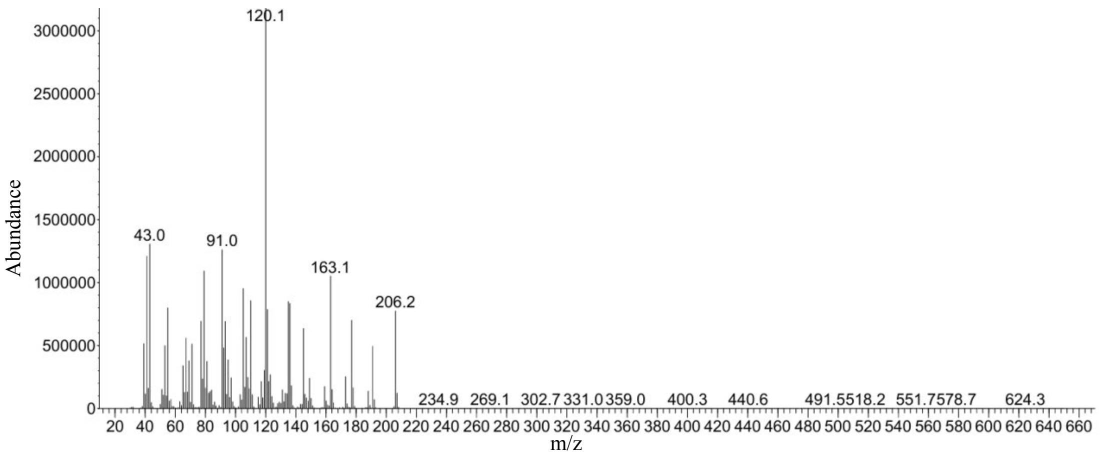

Scheme S25. MS data of **28b** from biotransformation of Omp7 with Keto-FPP (**15**) (t_R = 9.036 to 9.049 min).

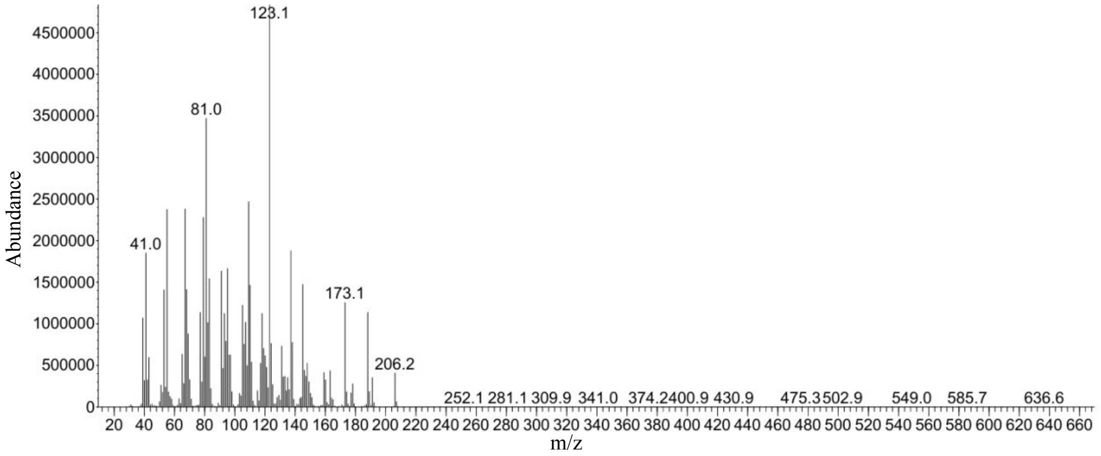

Scheme S26. MS data of **25** from biotransformation of Omp7 with Keto-FPP (**15**) (t_R = 10.252 to 10.280 min).

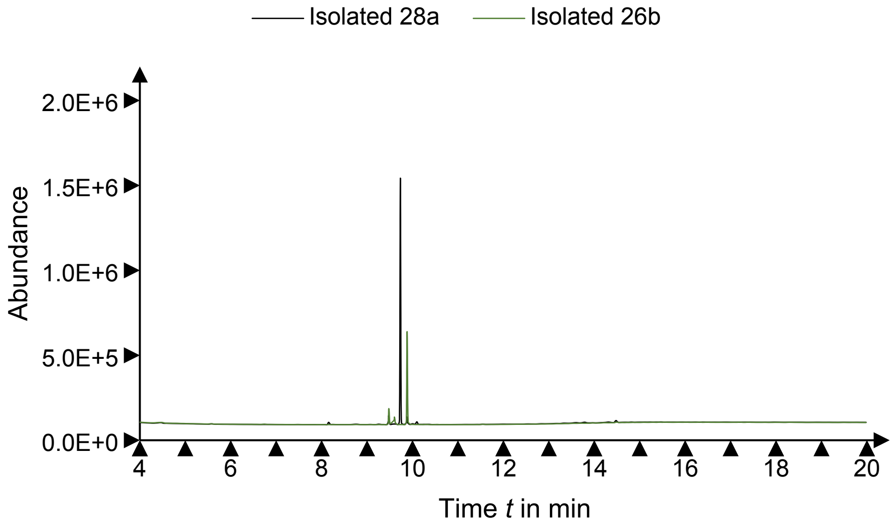

Scheme S27. GC chromatogram of isolated compounds **28a** (black) and **26b** (green).

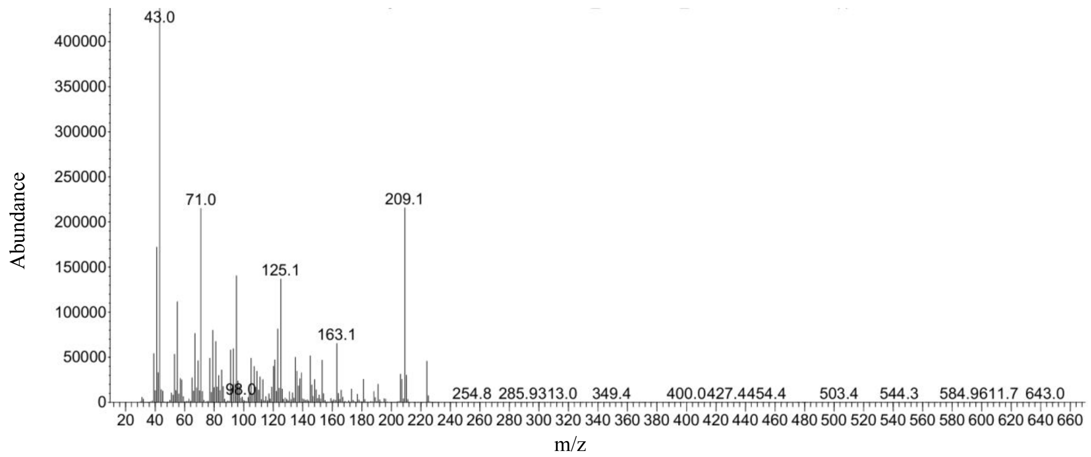

Scheme S28. MS data of **28a** from biotransformation of Omp7 with Keto-FPP (**15**) (t_R = 9.678 to 9.692 min).

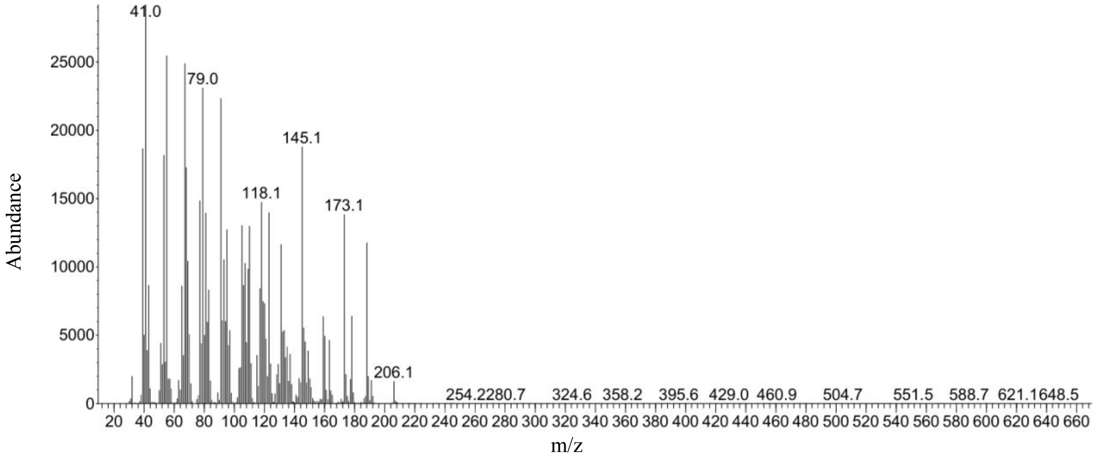

Scheme S29. MS data of **26b** from biotransformation of Omp7 with Keto-FPP (**15**) (t_R = 9.829 to 9.856 min).

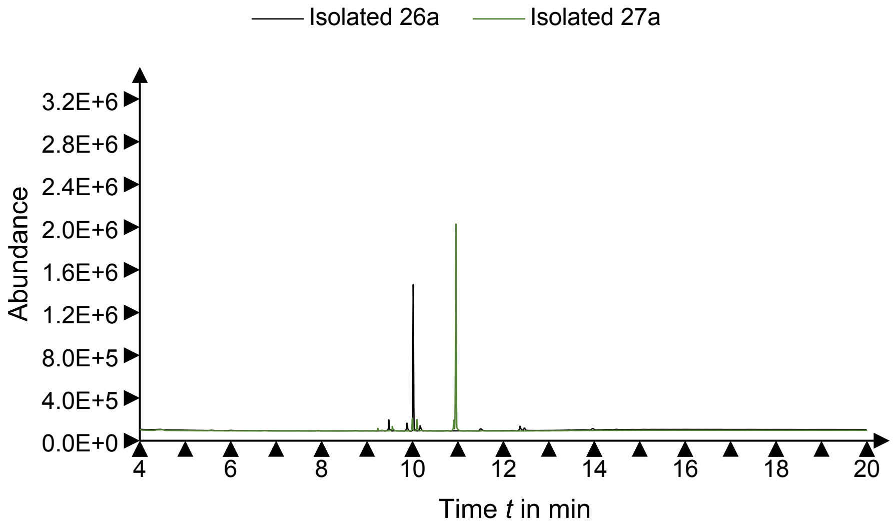

Scheme S30. GC chromatogram of isolated compounds **26a** (black) and **27a** (green).

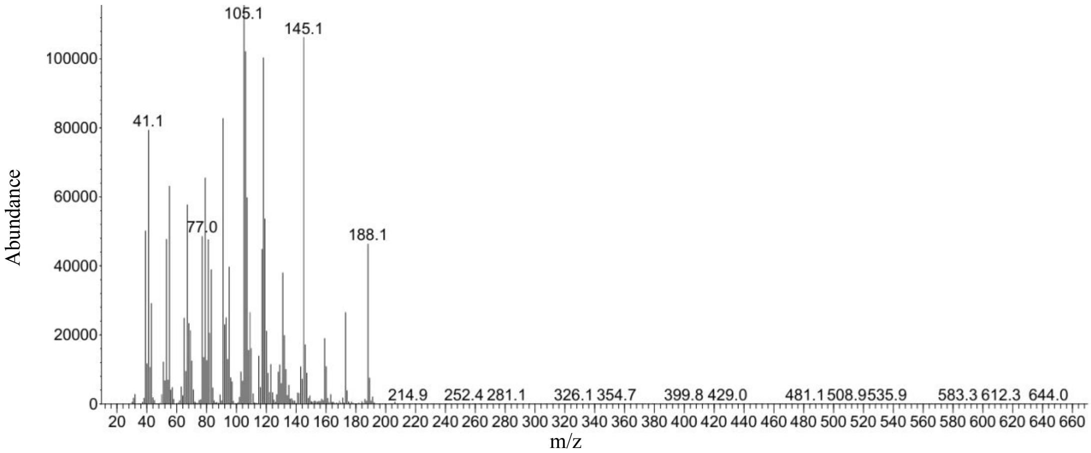

Scheme S31. MS data of **26a** from biotransformation of Omp7 with Keto-FPP (**15**) (t_R = 9.965 to 9.992 min).

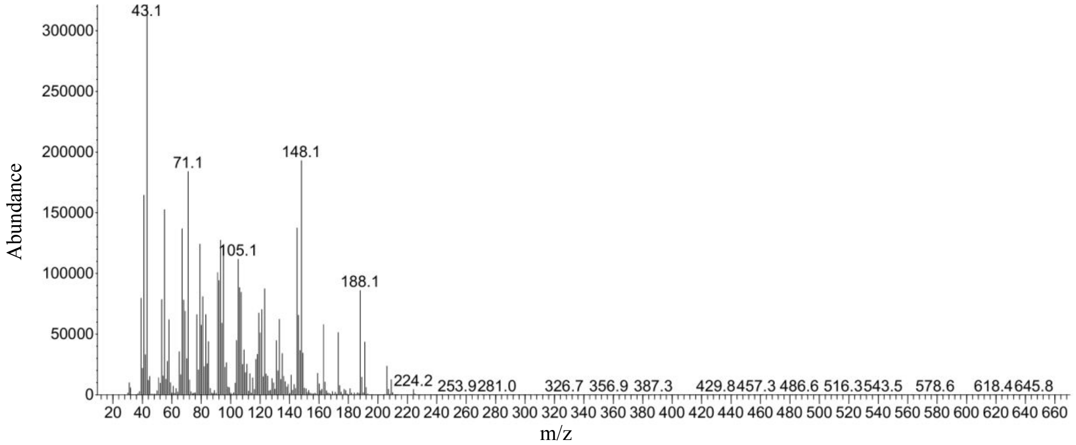

Scheme S32. MS data of **27a** from biotransformation of Omp7 with Keto-FPP (**15**) (t_R = 10.895 to 10.922 min).

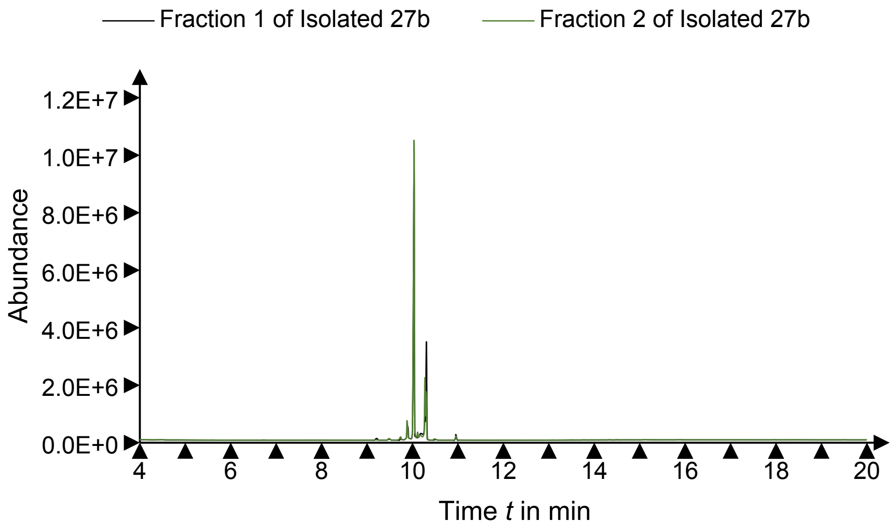

Scheme S33. GC chromatogram of isolated fractions of **27b**.

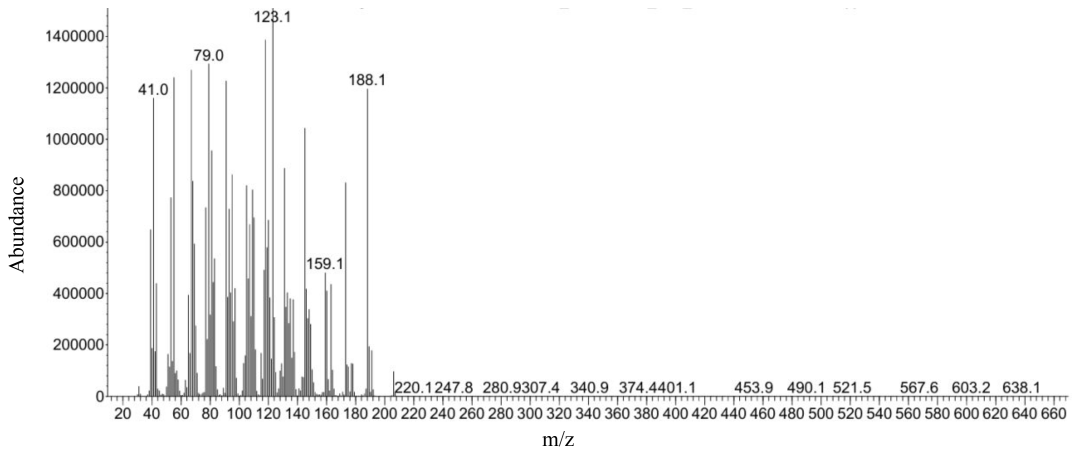

Scheme S34. MS data of **27b** from biotransformation of Omp7 with Keto-FPP (**15**) (t_R = 9.965 to 9.993 min).

1.4.5 Biotransformation of Omp7 and *iso*-FPP

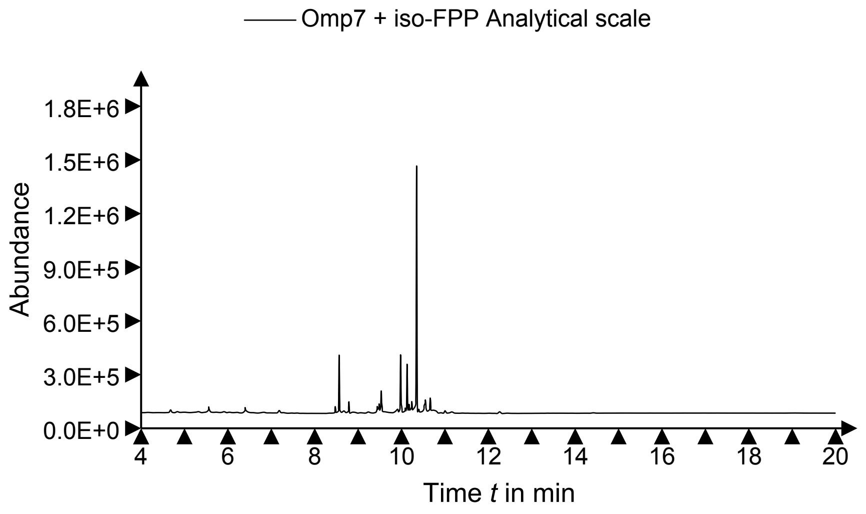

Scheme S35. GC chromatogram of analytical scale biotransformation of Omp7 with *iso*-FPP (**16**) in an analytical scale.

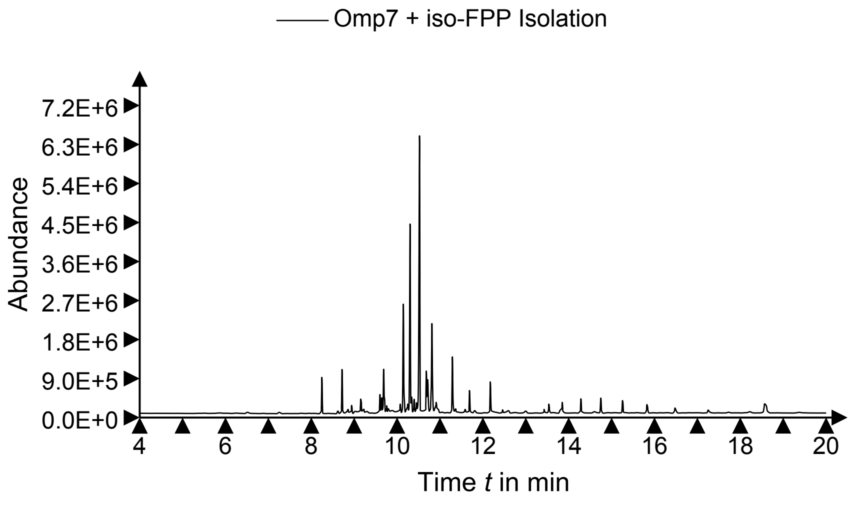

.

Scheme S36. GC chromatogram of biotransformation of Omp7 with *iso*-FPP (**16**) for product isolation.

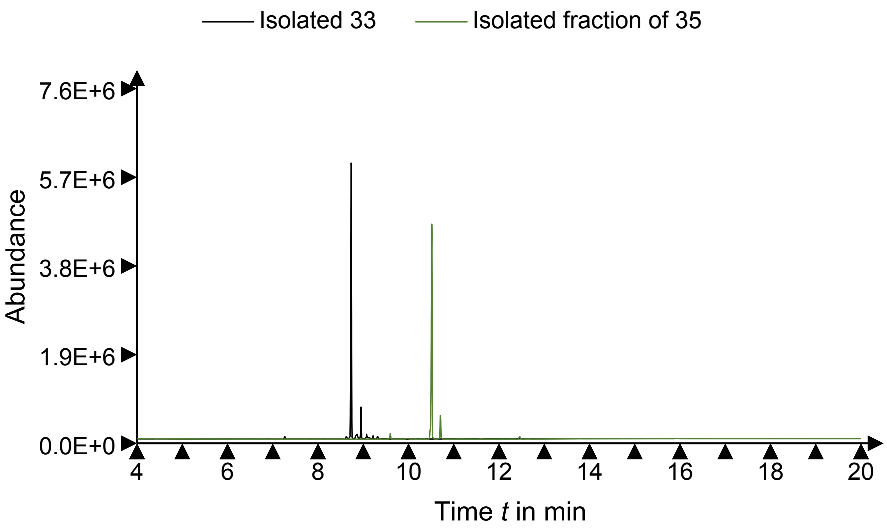

Scheme S37. GC chromatogram of isolated fractions of compounds **33** (black) and **35** (green).

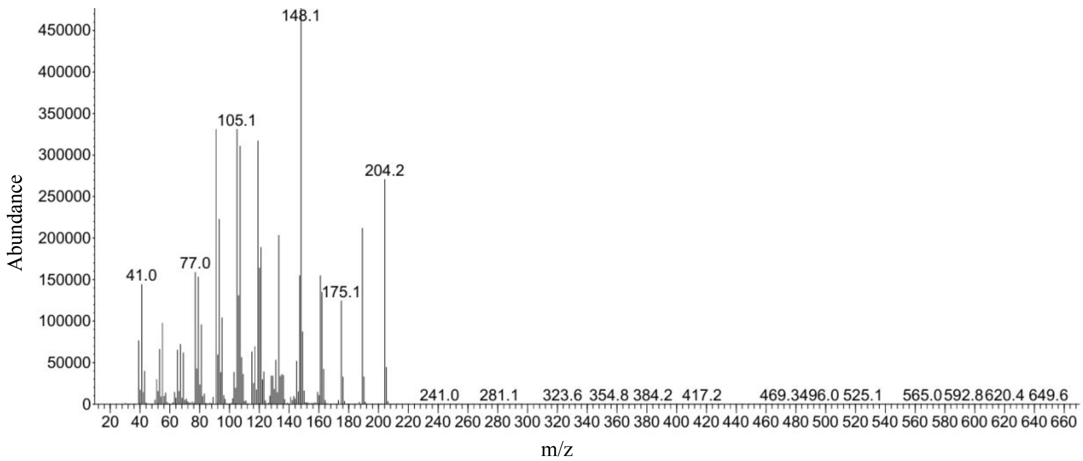

Scheme S38. MS data of **33** from biotransformation of Omp7 with *iso*-FPP (**16**) (t_R = 8.667 to 8.680 min).

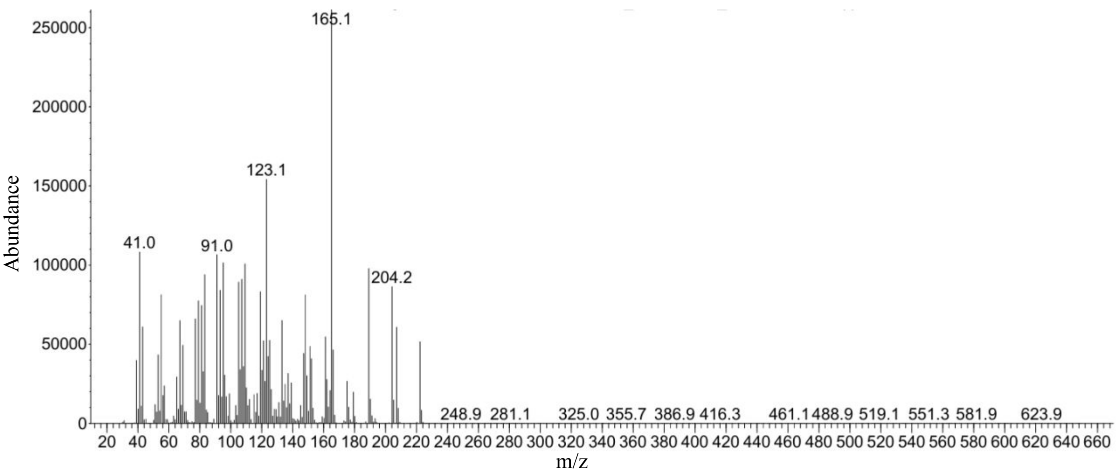

Scheme S39. MS data of **35** from biotransformation of Omp7 with *iso*-FPP (**16**) (t_R = 10.444 to 10.471 min).

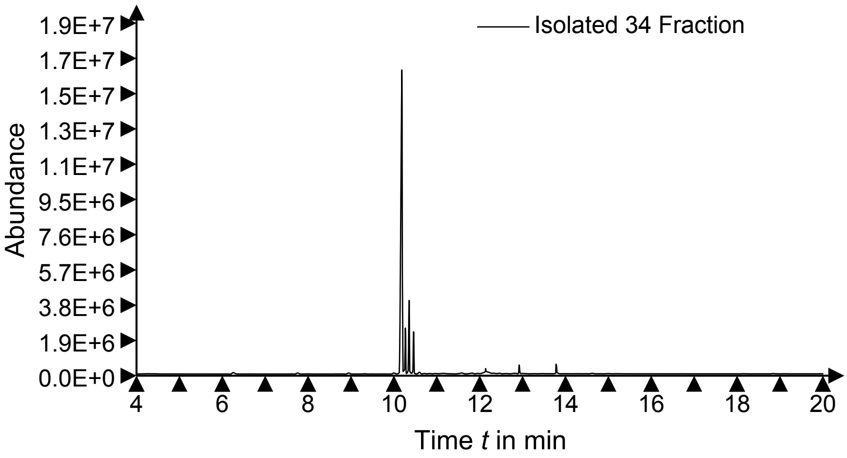

Scheme S40. GC chromatogram of isolated fractions of compound **34**.

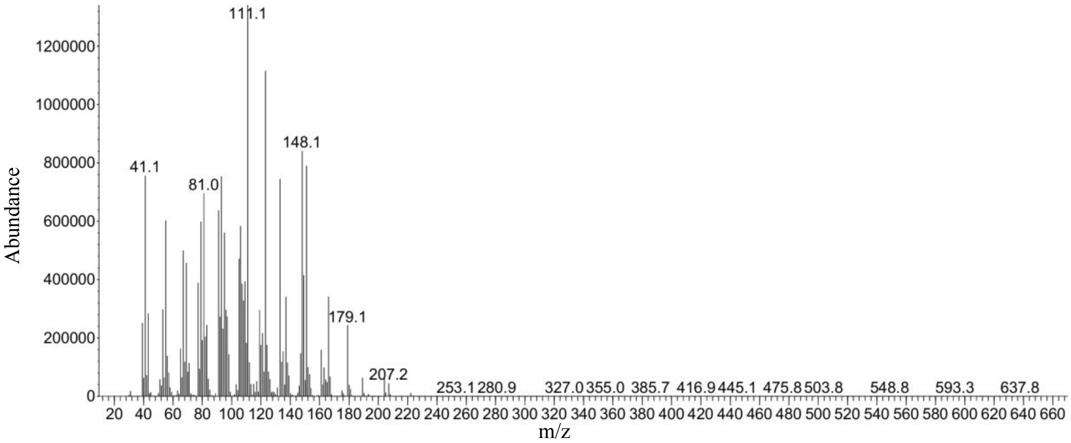

Scheme S41. MS data of **34** from biotransformation of Omp7 with *iso*-FPP (**16**) (t_R = 10.102 to 10.129 min).

1.4.6 Biotransformation of JeSTS4 and Keto-FPP

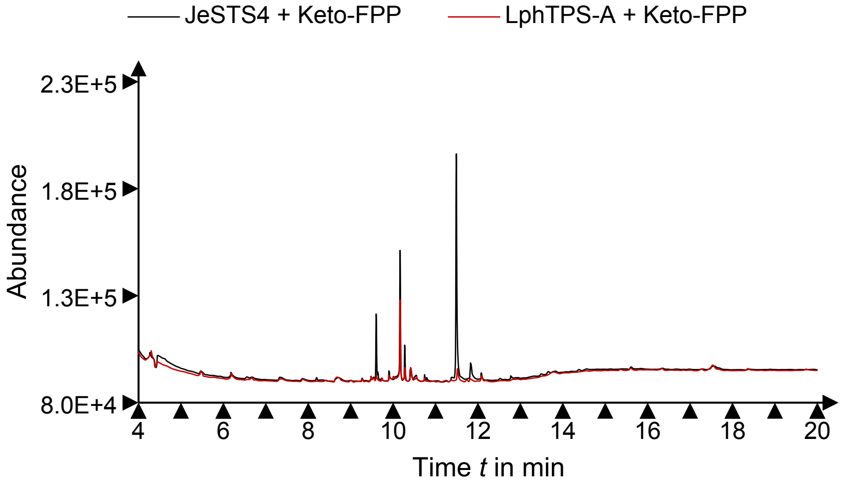

Scheme S42. GC chromatogram of Keto-FPP (**15**) and both JeSTS4 (black) and LphTPS (red) in an analytical scale.

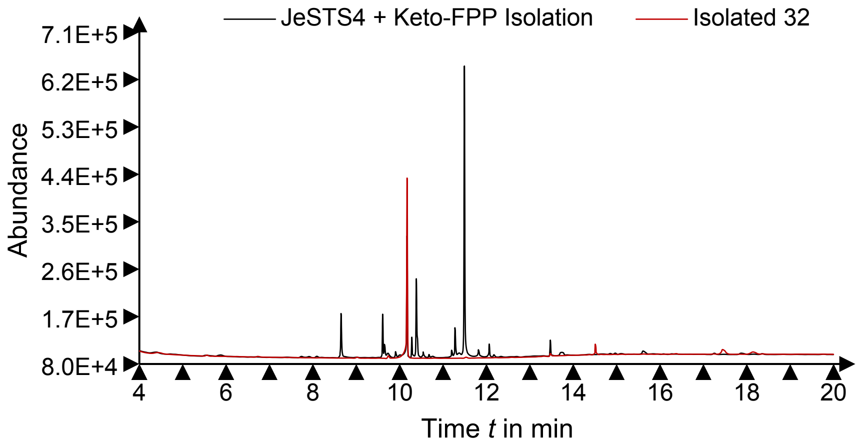

Scheme S43. GC chromatogram of JeSTS4 and Keto-FPP (**15**) for product isolation (black) and isolated **32** (red).

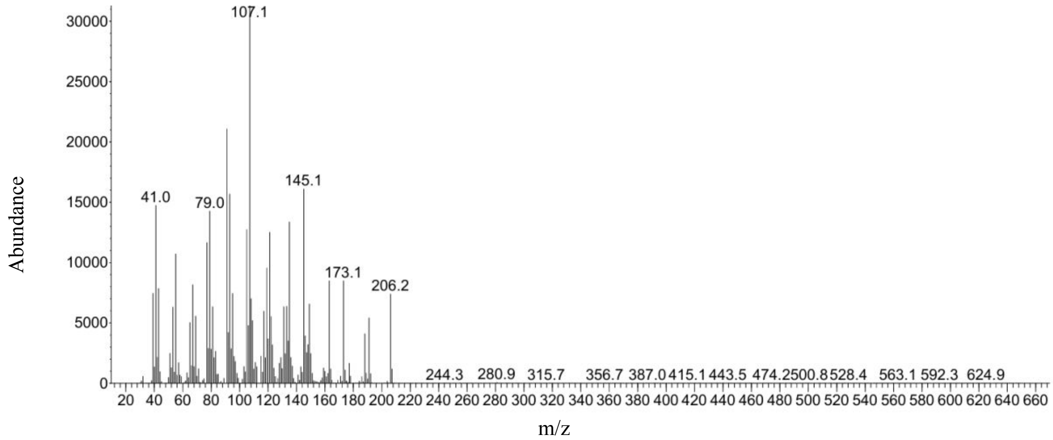

Scheme S44. MS data of **32** from biotransformation of JeSTS4 with Keto-FPP (**15**) (t_R = 10.143 min).

1.4.7 Biotransformation of JeSTS4 and *iso*-FPP

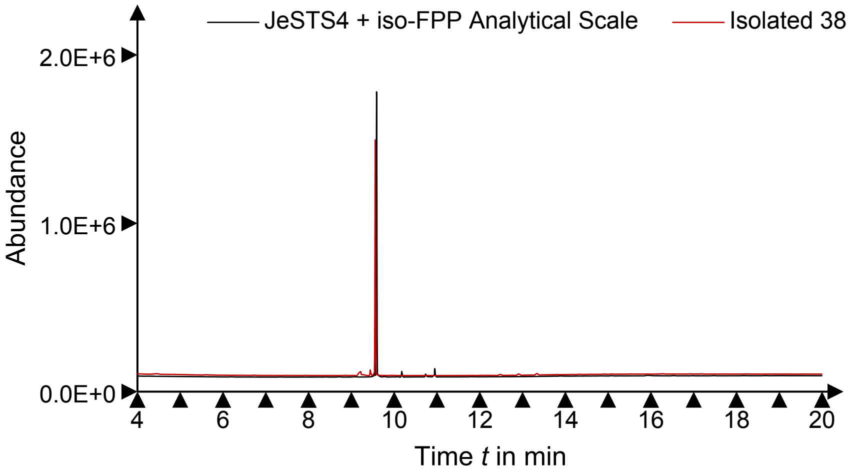

Scheme S45. GC chromatogram of *iso*-FPP (**16**) and JeSTS4 in an analytical scale (black) and the isolated product **38** (red).

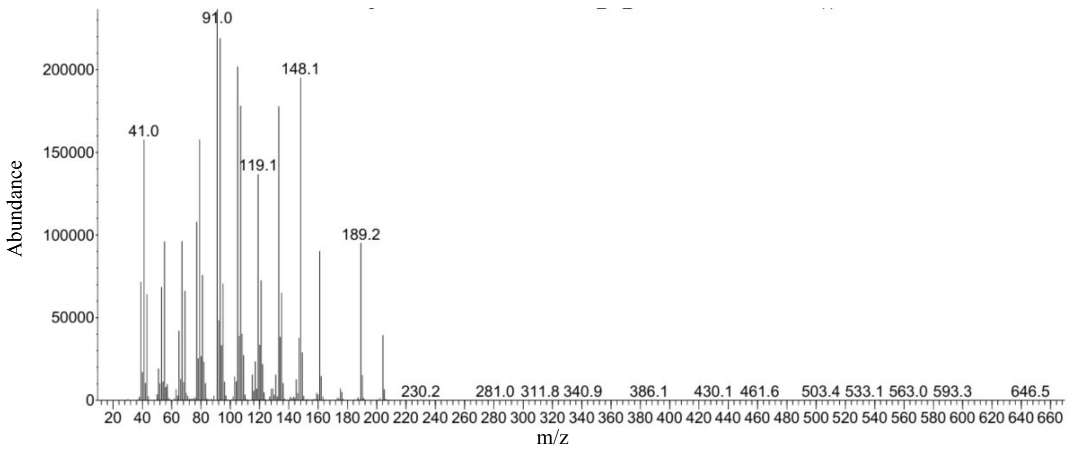

Scheme S46. MS data of **38** from biotransformation of JeSTS4 with *iso*-FPP (**16**) (t_R = 9.528 to 9.542 min).

1.4.8 Biotransformation of Pts and *iso*-FPP

Product formation with Pts and *iso*-FPP **16** showed significant differences between analytical scale and semi-preparative scale for product isolation, as only one product could be isolated. While the reaction showed a similar product distribution at the beginning of the reaction, only **37** was found after incubation overnight.

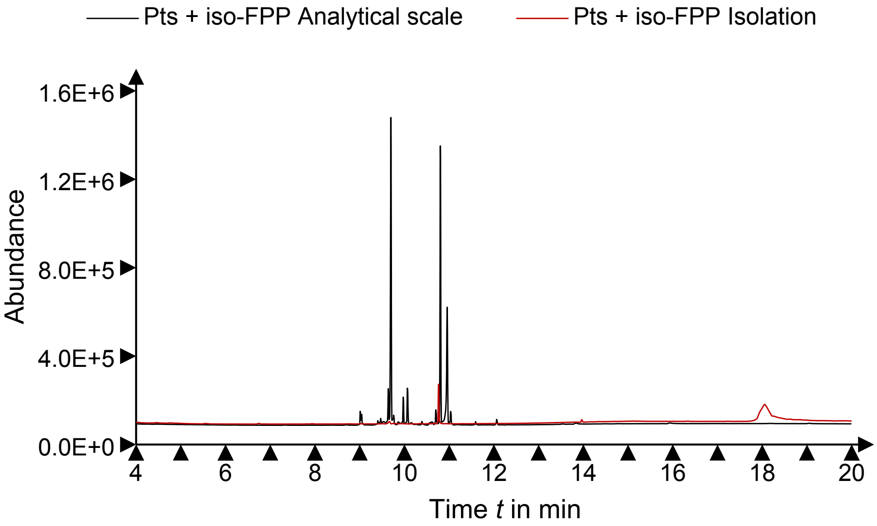

Scheme S47. GC chromatogram of *iso*-FPP **16** and Pts in an analytical scale (black) and for product isolation (red).

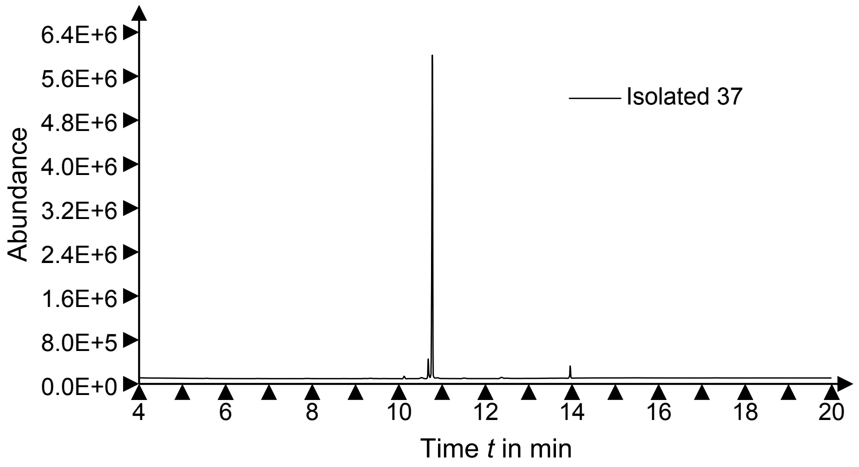

Scheme S48. GC chromatogram of isolated compound **37**

.

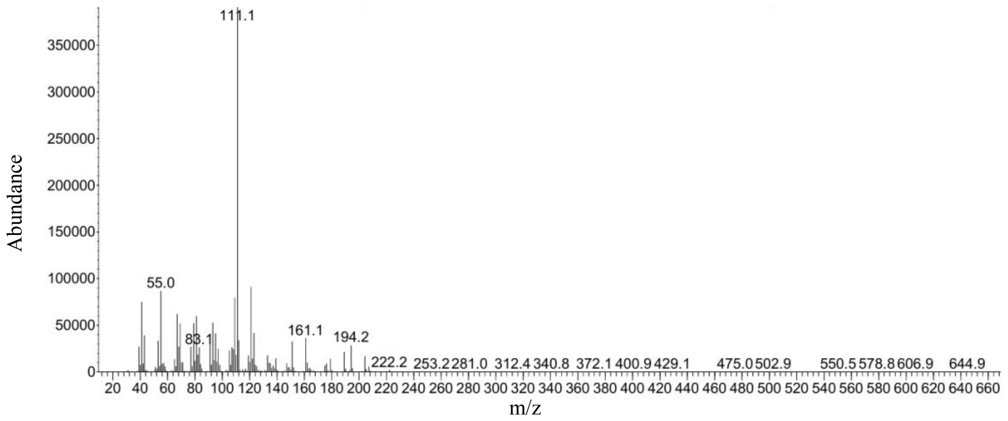

Scheme S49. MS data of **37** from biotransformation of Pts with *iso*-FPP (**16**) (t_R = 10.744 to 10.772 min).

1.4.9 Biotransformation of Tps32 and *iso*-FPP

Biotransformation of Tps32 and *iso*-FPP (**16**) leads to the formation of valencene (**39**) which can be observed as a small side product in the biotransformation with Tps32 and FPP (**1**).

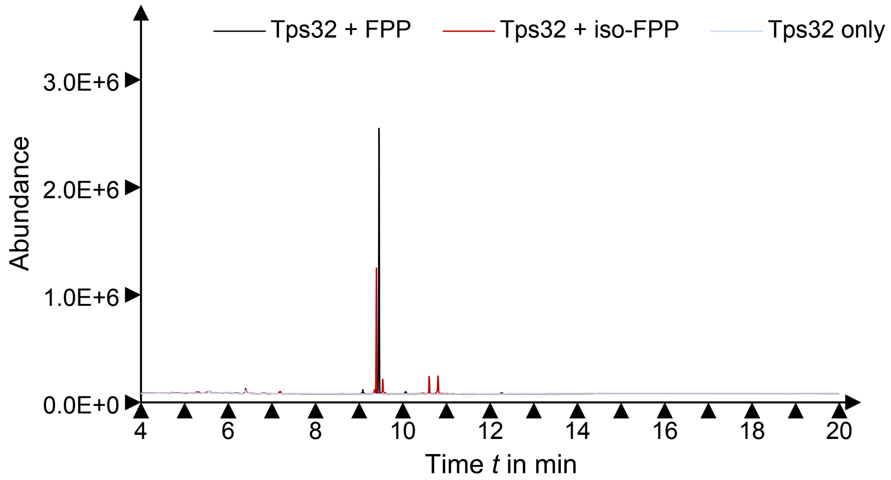

Scheme S50. GC chromatogram of biotransformation using Tps32 and either natural substrate FPP (**1**) (black) or *iso*-FPP (**16**) (red) or the negative control without substrate (grey).

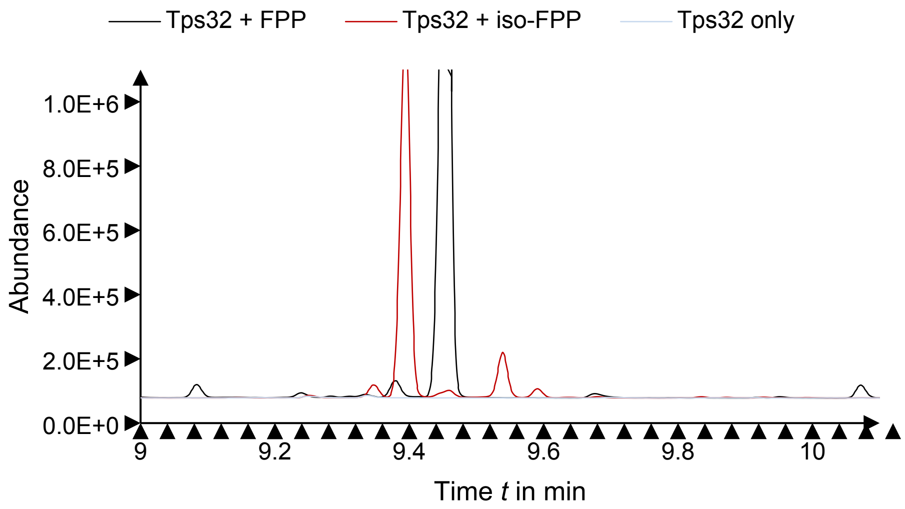

Scheme S51. Zoom-In of GC chromatogram of biotransformation using Tps32 and either natural substrate FPP (**1**) (black) or *iso*-FPP (**16**) (red) or the negative control without substrate (grey).

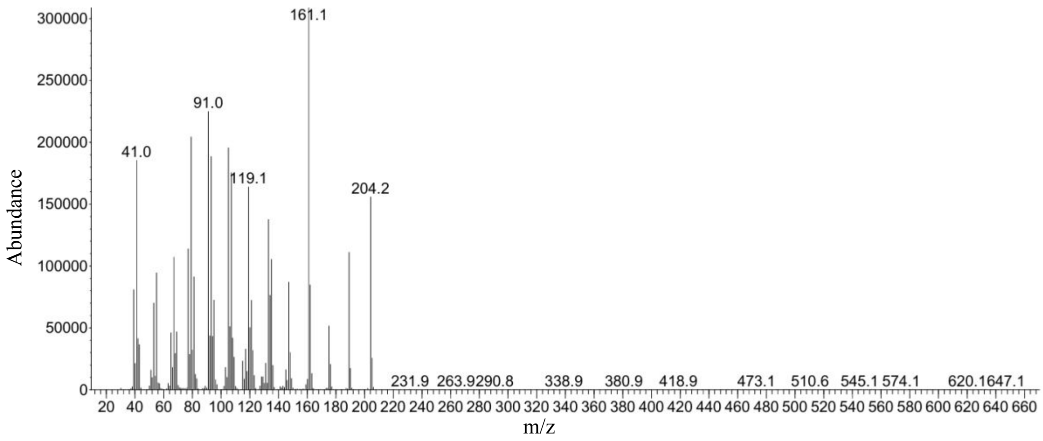

Scheme S52. MS data of **39** from biotransformation of Tps32 with *iso*-FPP (**16**) (t_R = 9.323 min).

1.4.10 Biotransformation of Omp7 mutants and different substrates

Omp7 WT and the mutants F81W, Y172F and Y227F were screened (30 °C, 200 rpm) with the natural substrate FPP (**1**), *iso*-FPP (**16**), keto-FPP (**15**) and nor-*iso*-FPP (**17**). The resulting chromatograms are shown below.

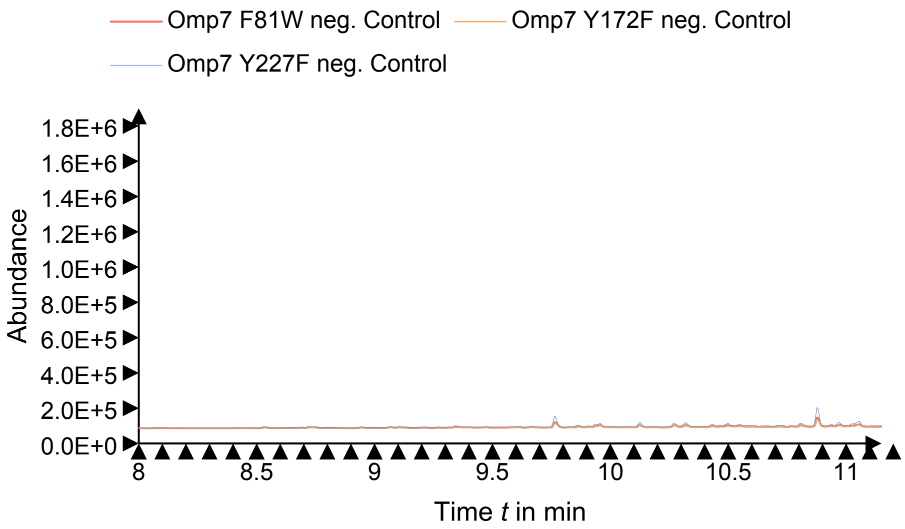

Scheme S53. Zoom-In of GC chromatograms of Omp7 variants (F81W in red, Y172F in yellow, Y227F blue) without substrates as negative control.

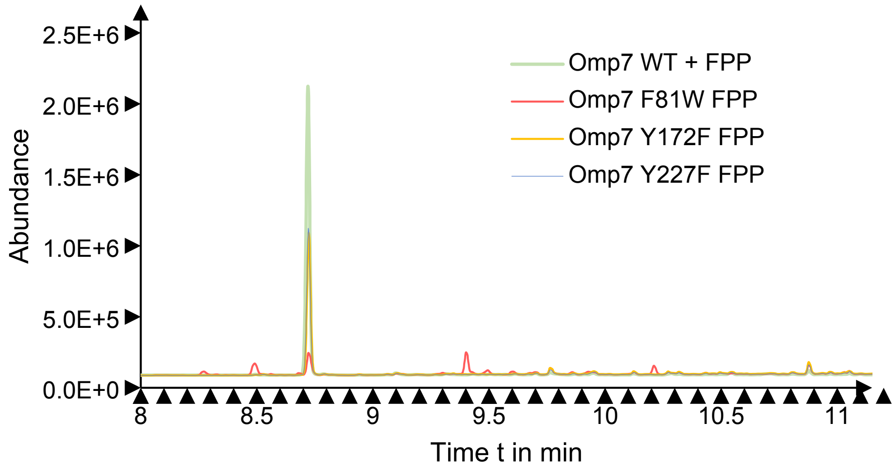

Scheme S54. Zoom-In of GC chromatograms of Omp7 variants (WT in green, F81W in red, Y172F yellow, Y227F blue) and FPP (**1**).

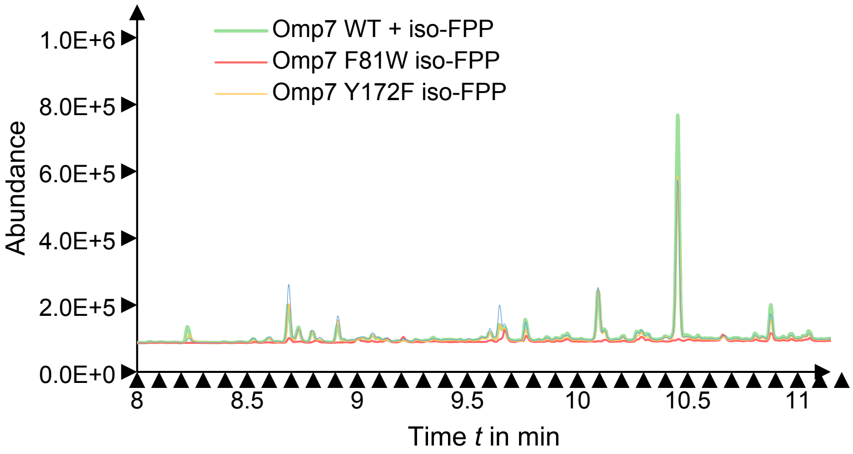

Scheme S55. Zoom-In of GC chromatograms of Omp7 variants (WT in green, F81W in red, Y172F yellow, Y227F blue) and *iso*-FPP (**16**).

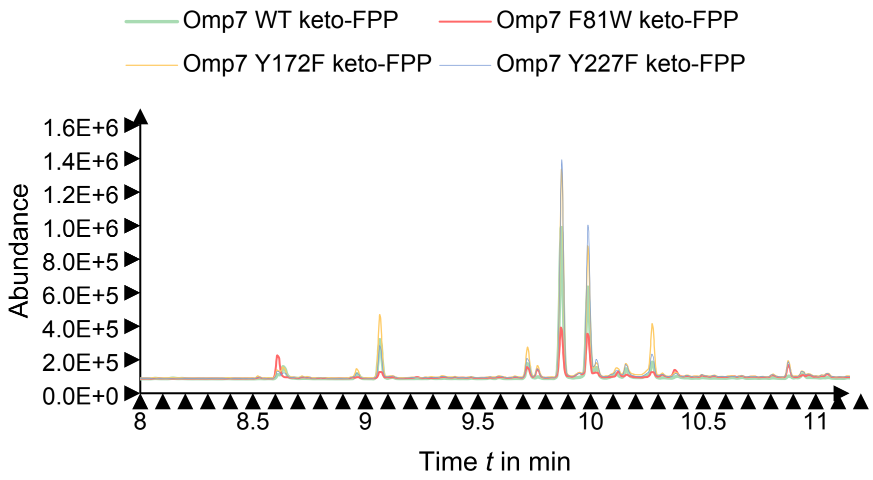

Scheme S56. Zoom-In of GC chromatograms of Omp7 variants WT in green, F81W in red, Y172F yellow, Y227F blue) and keto-FPP (**15**).

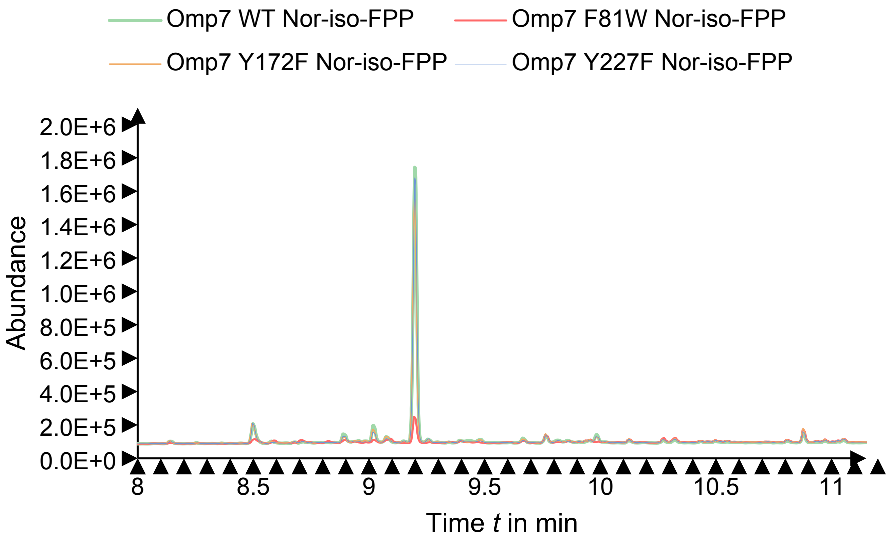

Scheme S57. Zoom-In of GC chromatograms of Omp7 variants (WT in green, F81W in red, Y172F yellow, Y227F blue) and Nor-*iso*-FPP (**17**).

1.4.11 Biotransformation of Omp7 F81W mutant and FPP

Since the reaction of F81W mutant and FPP (**1**) led to several new products, the isolation was performed using a preparative GC. The chromatograms of two isolated fractions are shown below.

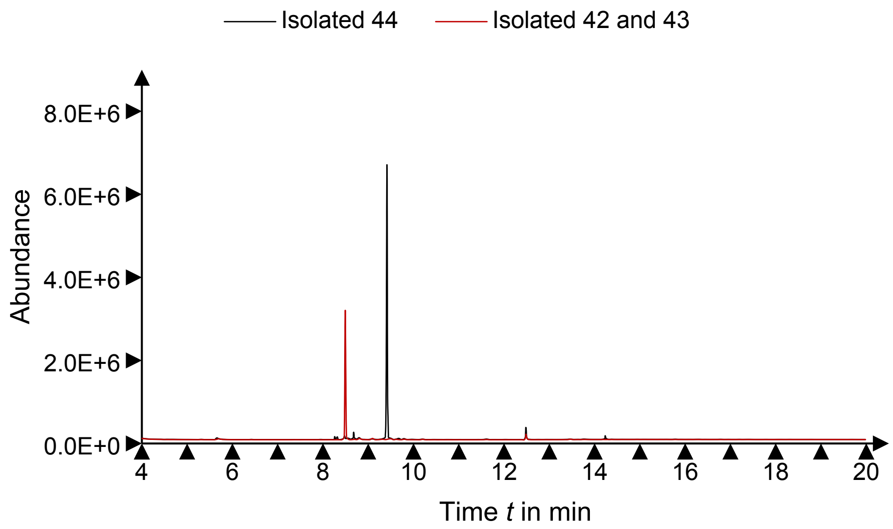

Scheme S58. GC chromatograms of isolated biotransformation products **42** – **44**.

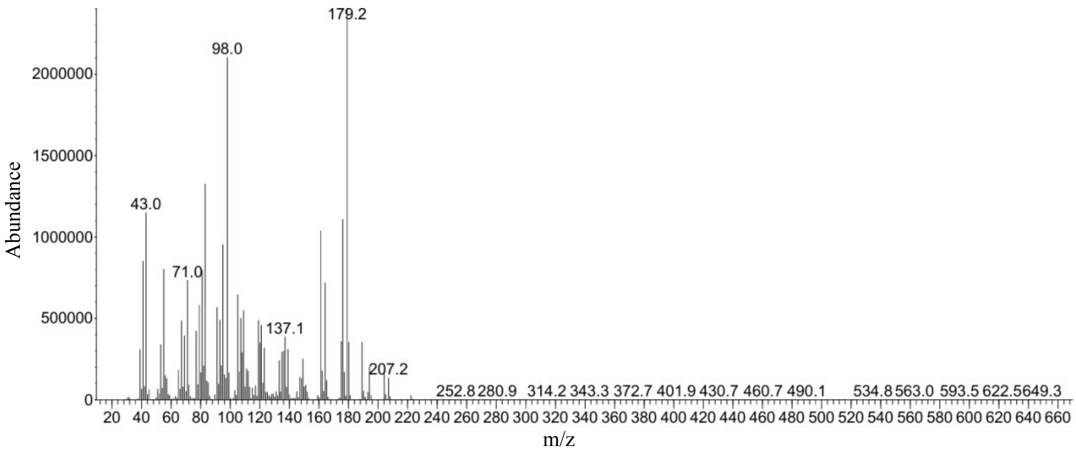

Scheme S59. MS data of **44** from biotransformation of Omp7 F81W with FPP (**1**) (t_R = 9.350 to 9.377 min).

While **42** and **43** appeared as one compound during previous GC measurements and during the preparative GC, the ^{13}C NMR revealed 30 carbon atoms. We therefore injected the sample to a different type of column.

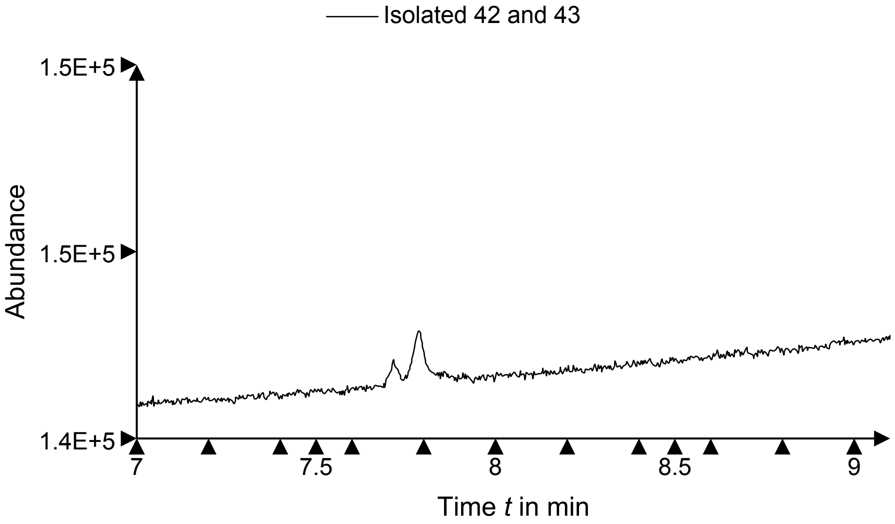

Scheme S60. GC chromatograms of isolated biotransformation products (prep-GC) **42** and **43** on Optima WAX column.

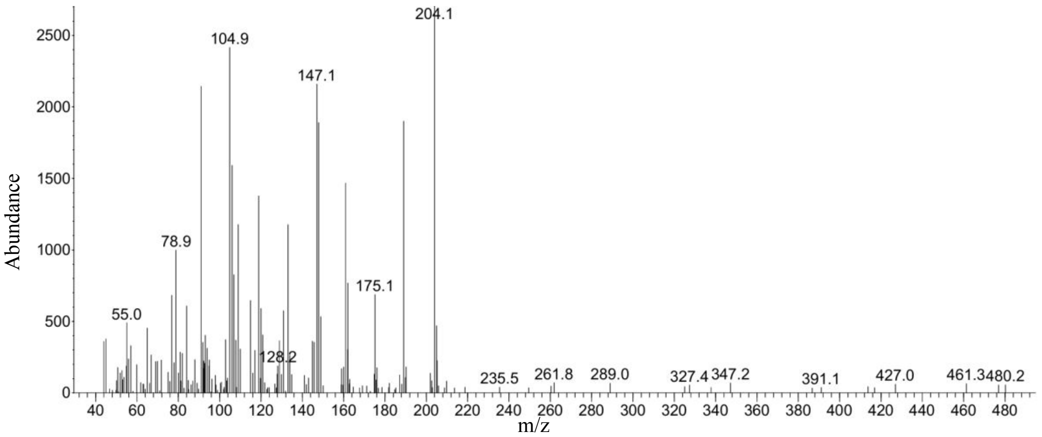

Scheme S61. MS data of **43** from biotransformation of Omp7 F81W with FPP (**1**) (t_R = 7.697 to 7.708 min).

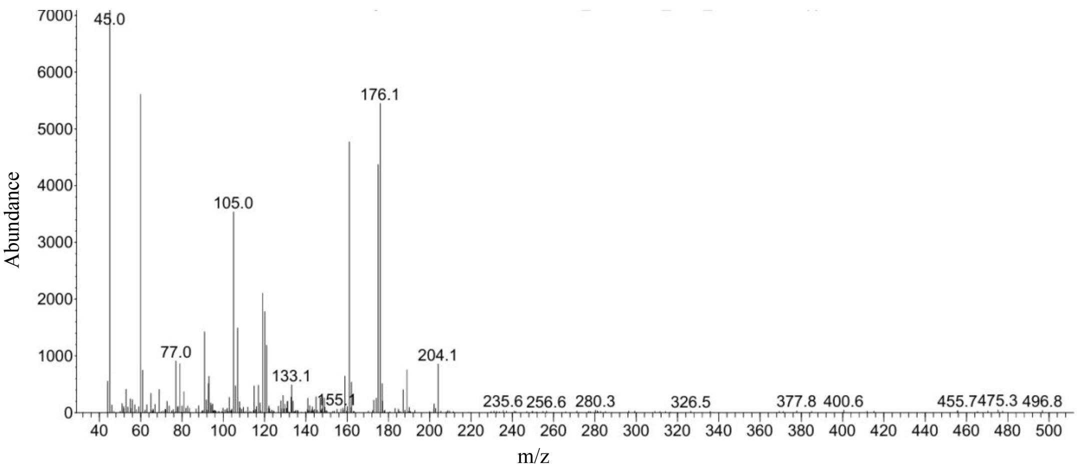

Scheme S62. MS data of **42** from biotransformation of Omp7 F81W with FPP (**1**) (t_R = 7.766 to 7.787 min).

1.4.12 Biotransformation of Omp7 and Nor-*iso*-FPP

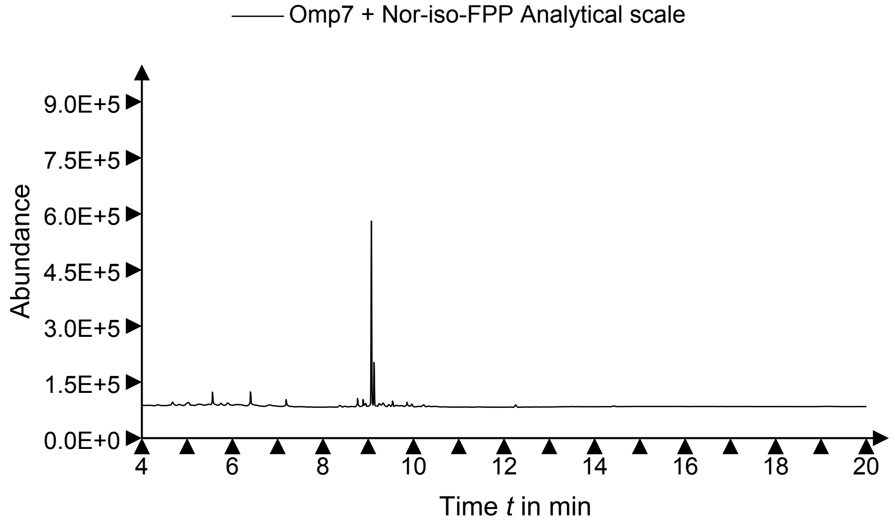

Scheme S63. GC chromatogram of analytical scale biotransformation of Omp7 with Nor-*iso*-FPP (**17**).

Note: When the biotransformation was repeated at larger scale we detected signals not observed in the initial screening. Two of these signals at 8.98 min and 9.16 min showed *m/z* values to be expected for biotransformation products resulting from "nor-*iso*"-FPP (**17**) as substrate. Therefore, isolation and structure elucidation focused on these compounds. After preparative-GC isolation of **41** a second signal with similar intensity was detected. We rationalize the formation of this new compound as a result of a heat induced reaction that occurred during GC-MS including .during preparative GC, because the NMR spectra provided signals for one major product.

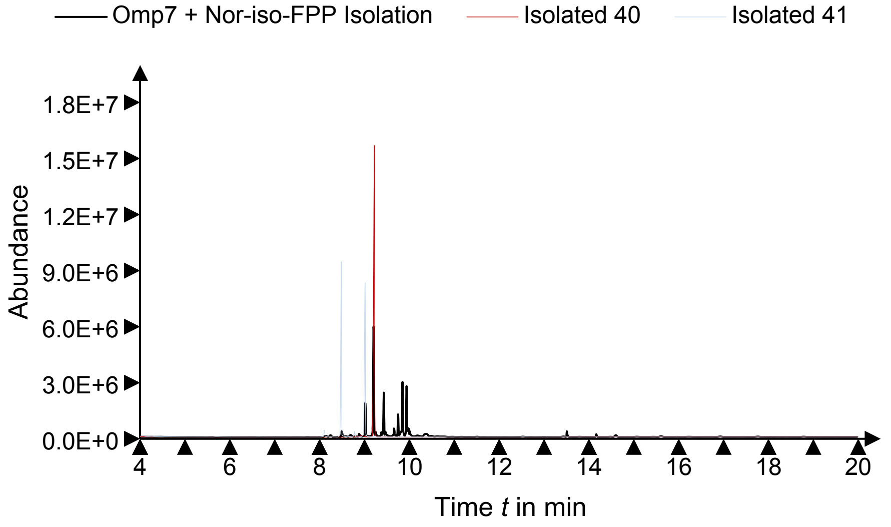

Scheme S64. GC chromatogram of biotransformation of Omp7 with Nor-*iso*-FPP (**17**) for product isolation (black) and the isolated products **40** (red) and **41** (light blue).

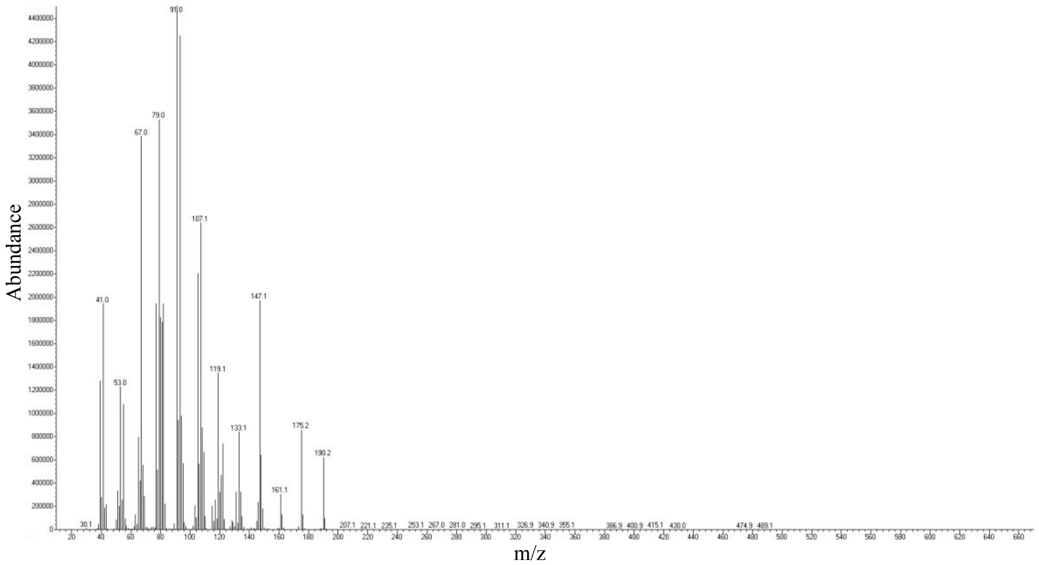

Scheme S65. MS data of **40** from biotransformation of Omp7 with Nor-*iso*-FPP (**17**) (t_R = 9.145 min to 9.172 min).

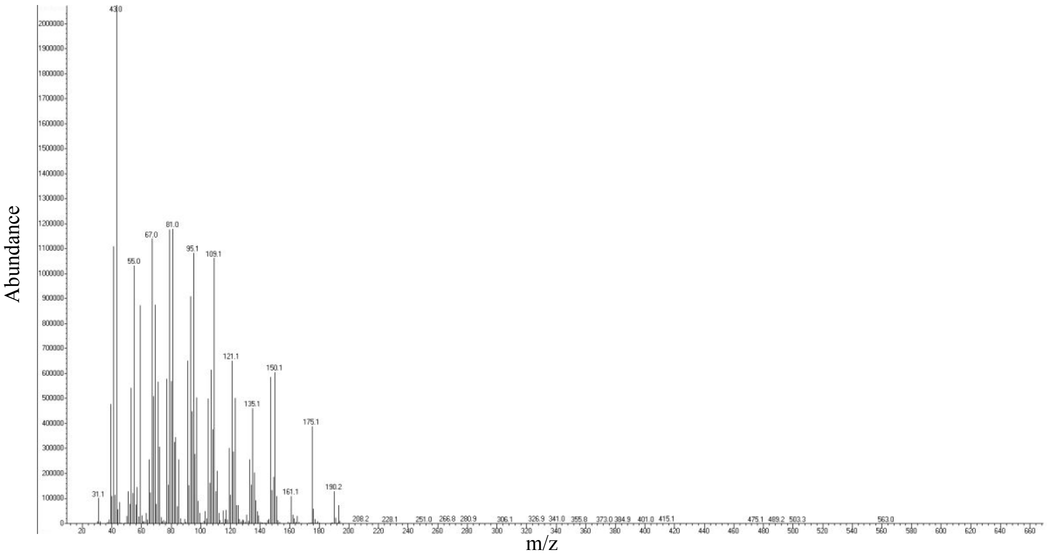

Scheme S66. MS data of **41** from biotransformation of Omp7 with Nor-*iso*-FPP (**17**) and isolated product measurement (t_R = 8.940 min to 8.967 min).

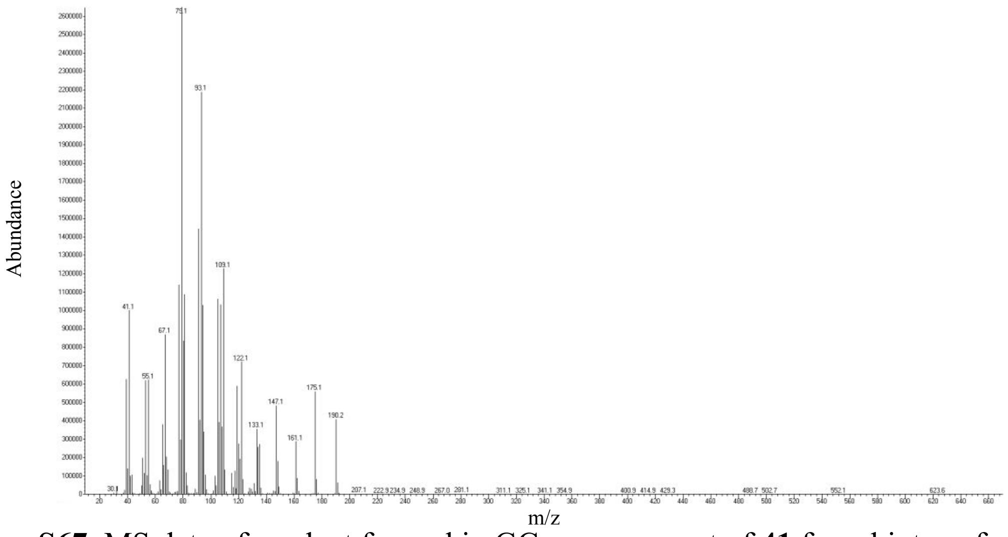

Scheme S67. MS data of product formed in GC measurement of **41** from biotransformation of Omp7 with Nor-*iso*-FPP (**17**) and isolated product measurement (t_R = 8.421 min to 8.448 min).

1.4.13 Retention indices of isolated products

Table S1: Retention index of isolated products.

Biotransformation product	Retention index
**33**	1408
**35**	1707
**34**	1649
**32**	1673
**38**	1531
**37**	1744
**28b**	1461
**25**	1669
**28a**	1566
**26b**	1591
**26a**	1615
**27a**	1784
**27b**	1618
**31**	1679
**30**	1386
**29**	1629
**44**	1517
**42** und **43**	1372
**40**	1483
**41**	1452

1.5 Structure elucidations

1.5.1 Structure elucidation of 28b

Copies of ^{1}H-NMR and ^{13}C spectra of all biotransformation products are found in section 2 (Copies of NMR spectra). To simplify structure elucidation only the centre of each HSQC signal will be used as the chemical shift, despite different multiplicities, since overlapping signals can be differentiated more easily.

Table S2. ^{1}H NMR signals and their corresponding ^{13}C NMR signals for compound **28b** as analysed with the support of ^{1}H-^{13}C HSQC and ^{13}C{^{1}H} DEPT135 experiments. The quaternary carbon atoms are listed at the bottom.

δ (^{1}H)/ppm	δ (^{13}C)/ppm	DEPT135/HSQC phase
4.92 + 4.71	108.3	CH_2
2.35 + 1.87	35.6	CH_2
2.18	48.4	CH/CH_3
2.09 + 1.49	31.7	CH_2
2.08	33.4	CH/CH_3
1.87 + 1.60	38.2	CH_2
1.80 + 1.51	23.8	CH_2
1.46 + 1.32	32.6	CH_2
1.43 + 1.37	38.0	CH_2
0.98	18.3	CH/CH_3
0.97	18.4	CH/CH_3
	151.2	C_q
	89.2	C_q
	84.4	C_q

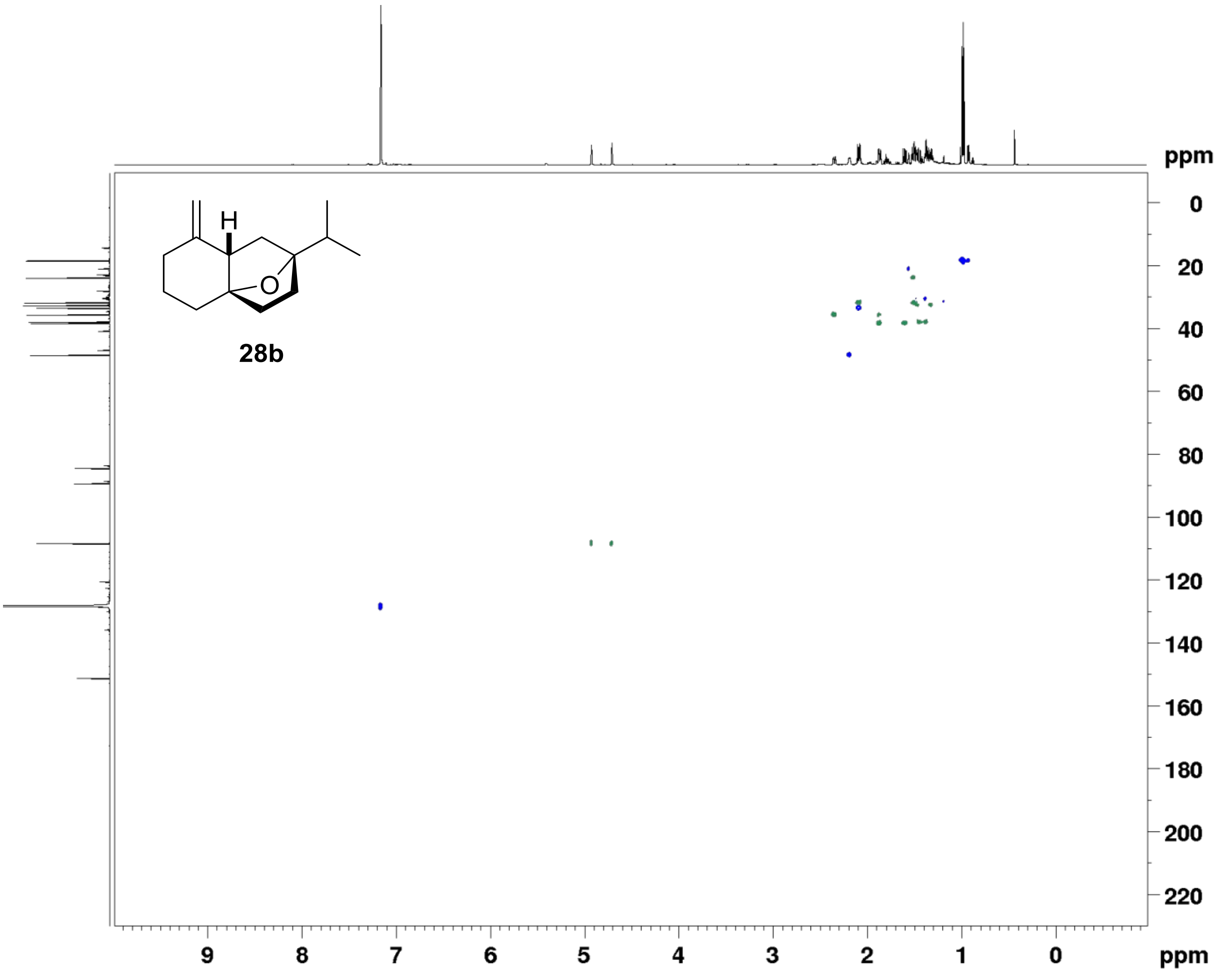

Figure S5. ^{1}H-^{13}C HSQC NMR spectrum of compound **28b** in C_6D_6 (pos. phase = blue (CH/CH_3), neg. phase = green (CH_2)).

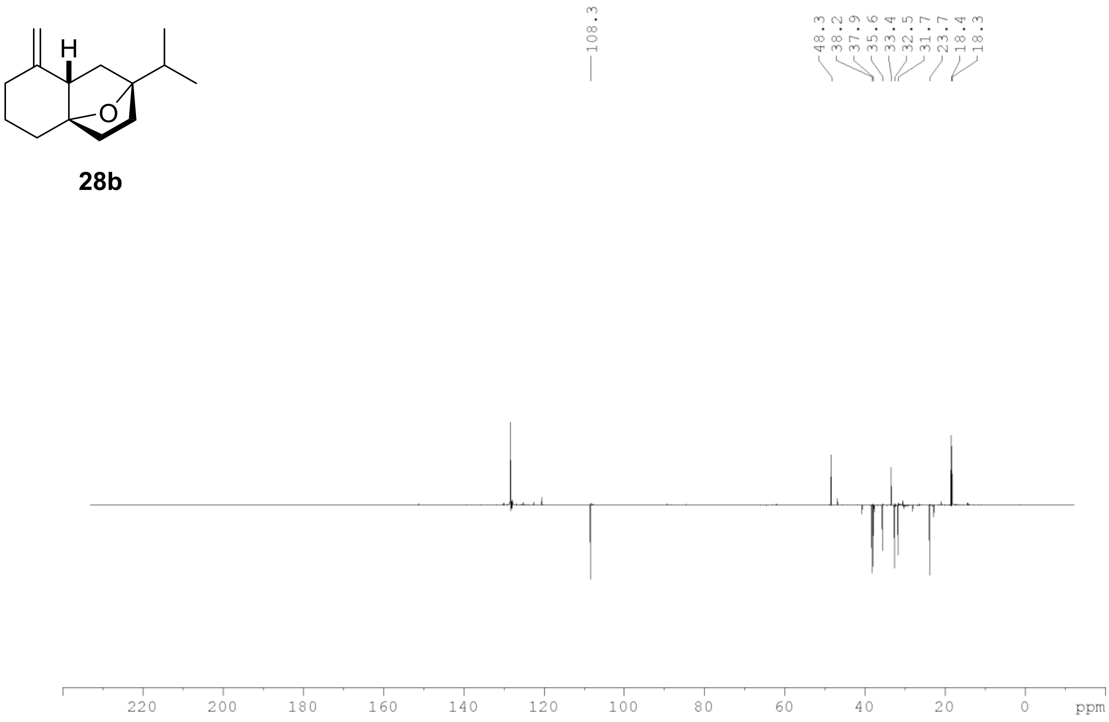

Figure S6. ^{13}C{^{1}H} DEPT135 NMR spectrum of compound **28b** in C_6D_6.

Table S3. ^{1}H NMR signals and the corresponding ^{1}H-^{1}H COSY correlations for compound **28b**. Signals with weak intensities are given in parentheses.

δ (^{1}H)/ppm	COSY correlations
4.92 + 4.71	2.18, 1.87, (2.35)
2.35 + 1.87	2.09, 1.80, 1.51
2.18	1.87, 1.60
2.09 + 1.49	2.35 ,1.80
2.08	0.98, 0.97
1.87 + 1.60	2.18
1.80 + 1.51	2.35, 2.09, 1.87
1.46 + 1.32	1.43 + 1.32
1.43 + 1.37	1.46 + 1.37
0.98	2.08
0.97	2.08

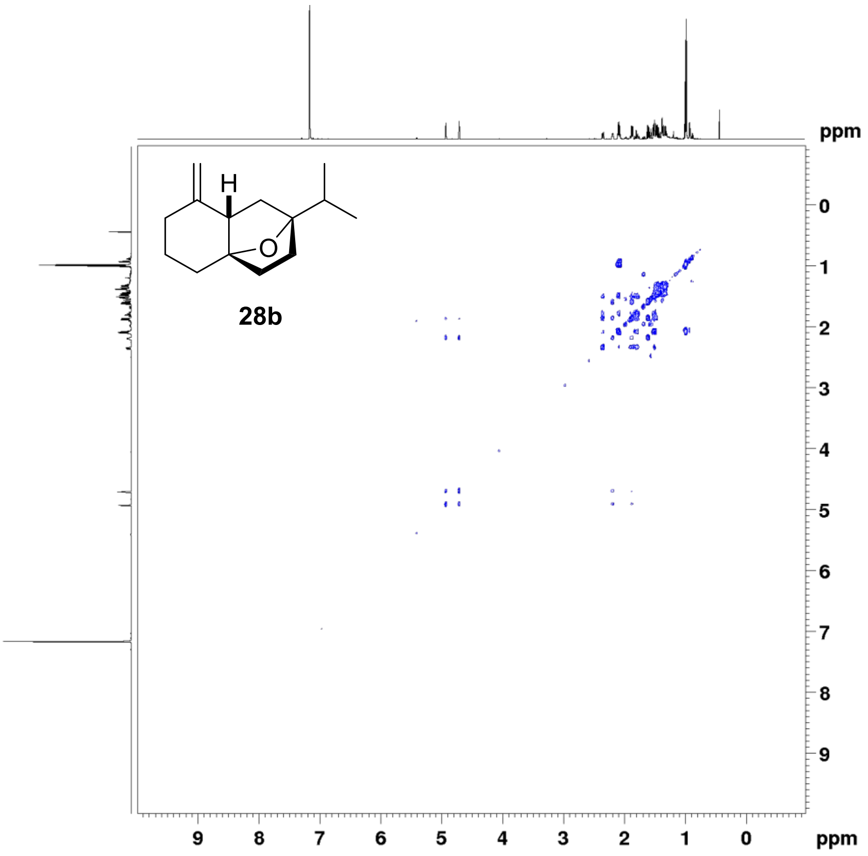

Figure S7. ^{1}H-^{1}H COSY NMR spectrum of compound **28b** in C_6D_6.

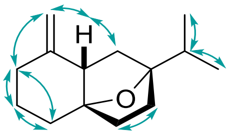

28b

Figure S8. Key ^{1}H-^{1}H COSY NMR correlations of **28b** as indicated by arrows.

Table S4. Selected correlations between ^{13}C NMR signals and neighbouring ^{1}H NMR signals as collected from the ^{1}H-^{13}C HMBC spectrum of compound **28b**. Note: Signals with weak intensities are given in parentheses.

δ (^{1}H)/ppm	δ (^{13}C)/ppm
4.92 + 4.71	151.2, 48.4, 35.6
2.35 + 1.87	151.2, 108.3, 48.4, 31.7, 23.8
2.18	151.2, 33.4, (108.3)
2.09 + 1.49	84.4, 48.4, 35.6
2.08	89.2, 38.2, 32.6, 18.4, 18.3
1.87 + 1.60	151.2, 84.4, 48.4, 32.6
1.80 + 1.51	151.2, 84.4, 35.6
1.46 + 1.32	84.4, 38.0
1.43 + 1.37	48.4, 32.6, (89.2, 84.4)
0.98	89.2, 33.4, 18.4
0.97	89.2, 33.4, 18.3

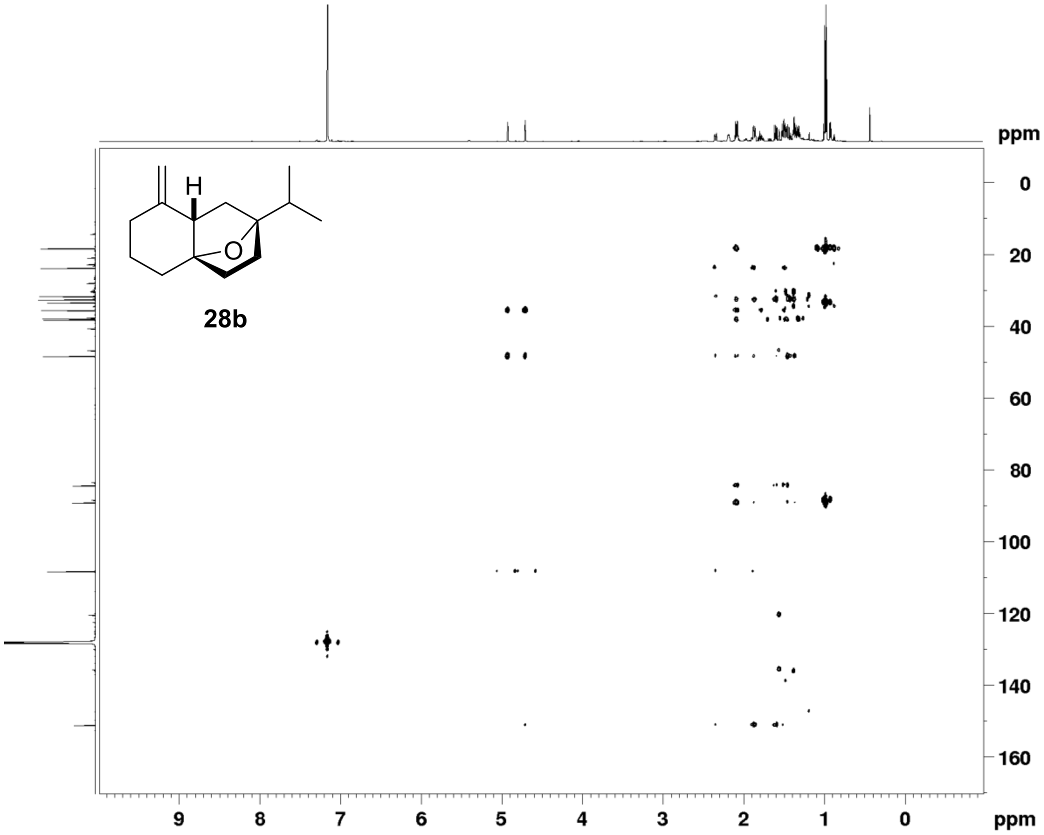

Figure S9. ^{1}H-^{13}C HMBC NMR spectrum of compound **28b** in C_6D_6.

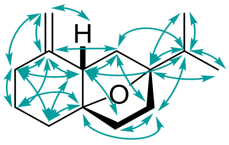

28b

Figure S10. Key ^{1}H-^{13}C HMBC NMR correlations of **28b** as indicated by arrows.

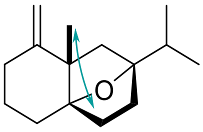

28b

Figure S11. Key ^{1}H-^{1}H NOESY NMR correlations of **28b** as indicated by arrows.

^{1}H-^{1}H NOESY NMR correlations between $H_{1.37}$ und $H_{2.18}$ indicates the *syn* orientation of the CH group and the two CH_2 groups.

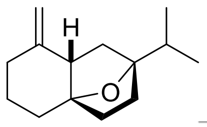

28b

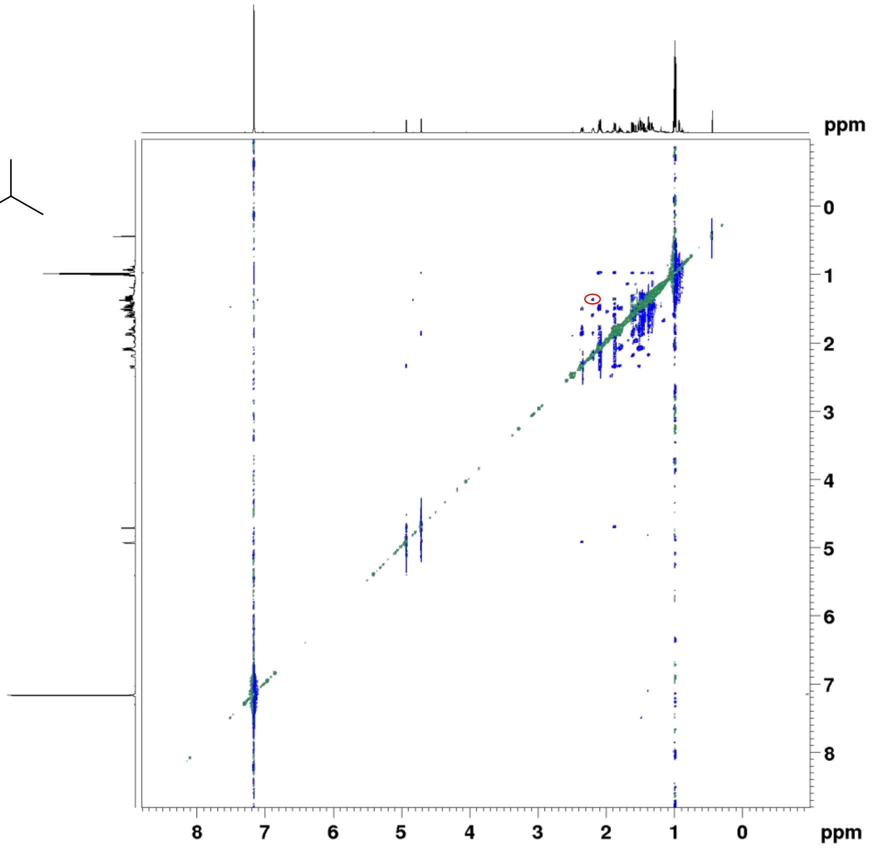

Figure S12. ^{1}H-^{1}H NOESY NMR spectrum of compound **28b** in C_6D_6. Key correlation for relative stereochemistry marked in red circle.

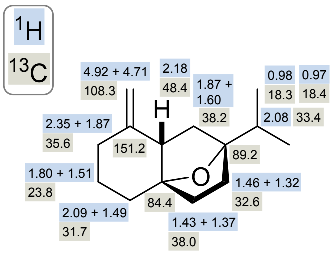

28b

Figure S13. Full assignment of chemical shifts for compound **28b**.

1.5.2 Structure elucidation of 25

^{1}H and ^{13}C NMR of **25** shows broad signals, likely due to high conformation flexibility. To simplify structure elucidation only the centre of each HSQC signal will be used as the chemical shift, despite different multiplicities, since overlapping signals can be differentiated more easily.

Table S5. ^{1}H NMR signals and their corresponding ^{13}C NMR signals for compound **25** as analysed with the support of ^{1}H-^{13}C HSQC and ^{13}C{^{1}H} DEPT135 experiments. The quaternary carbon atoms are listed at the bottom.

δ (^{1}H)/ppm	δ (^{13}C)/ppm	DEPT135/HSQC phase
5.07	125.4	CH/CH_3

δ (^{1}H)/ppm	δ (^{13}C)/ppm	DEPT135/HSQC phase
4.90 + 4.80	110.9	CH_2
2.25 + 1.96	28.1	CH_2
2.18 + 1.92	42.5	CH_2
2.15 + 2.04	29.1	CH_2
2.06 + 1.93	34.7	CH_2
1.94	45.4	CH/CH_3
1.79 + 1.65	19.7	CH_2
1.70	26.8	CH_2
1.60	22.8	CH/CH_3
1.53	22.9	CH/CH_3
	211.7	C_q
	148.0	C_q
	134.7	C_q

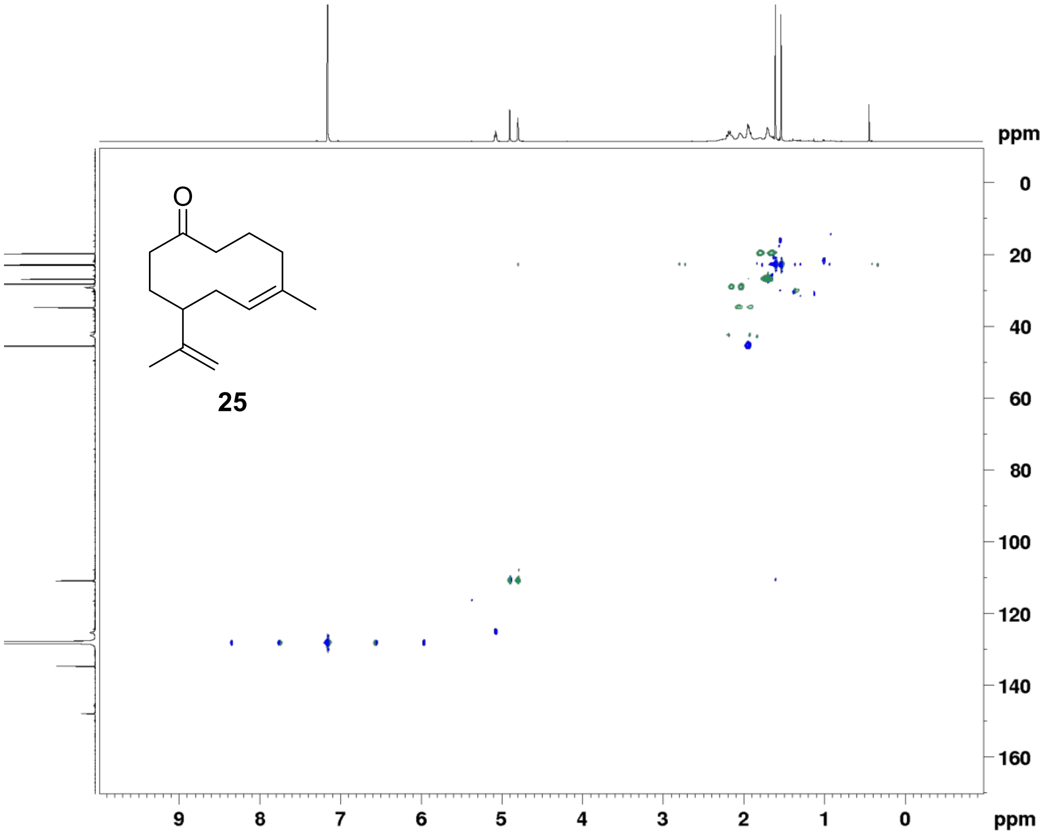

Figure S14. ^{1}H-^{13}C HSQC NMR spectrum of compound **25** in C_6D_6 (pos. phase = blue (CH/CH_3), neg. phase = green (CH_2)).

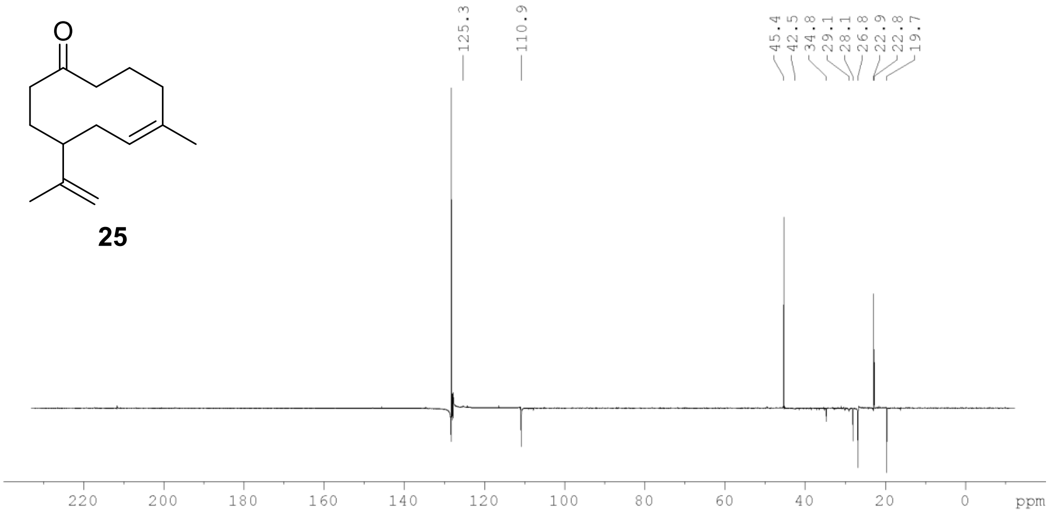

Figure S15. $^{13}C\{^1H\}$ DEPT135 NMR spectrum of compound **25** in C_6D_6.

Table S6: ^1H NMR signals and the corresponding ^1H-^1H COSY correlations for compound **25**. Signals with weak intensities are given in parentheses.

δ (^1H)/ppm	COSY correlations
5.07	2.15, 2.04, 1.53
4.90 + 4.80	1.60, (1.94)
2.25 + 1.96	(2.06)
2.18 + 1.92	1.70
2.15 + 2.04	5.07, 1.94, 1.53
2.06 + 1.93	
1.94	
1.79 + 1.65	
1.70	2.18 + 1.92
1.60	4.90 + 4.80, 1.94
1.53	5.07, 2.15 + 2.04

As the three CH_2 groups between the ketone and the trisubstituted double bond show no relevant COSY signals their assignment is based on HMBC correlations and chemical shifts in either allylic or alpha positions.

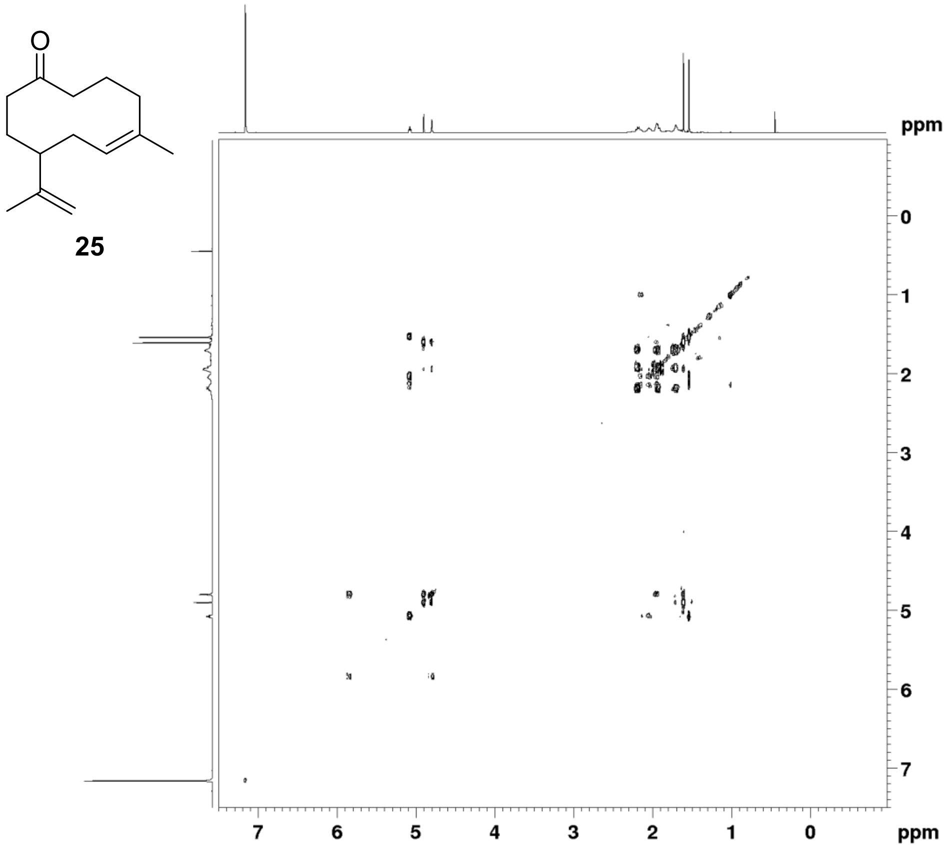

Figure S16. ¹H-¹H COSY NMR spectrum of compound **25** in C_6D_6.

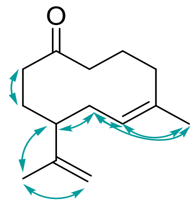

25

Figure S17. Key ¹H-¹H COSY NMR correlations of **25** as indicated by green arrows.

Table S7: Selected correlations between ¹³C NMR signals and neighbouring ¹H NMR signals as collected from the ¹H-¹³C HMBC spectrum of compound **25**. Note: Signals with weak intensities are given in parentheses.

δ (¹H)/ppm	δ (¹³C)/ppm
5.07	45.4, 28.1, 22.9, 19.7, (134.7)
4.90 + 4.80	148.0, 45.4, 22.8
2.25 + 1.96	
2.18 + 1.92	211.7, 45.4, 34.7, 26.8
2.15 + 2.04	45.4
2.06 + 1.93	211.7, 45.4, 26.8
1.94	148.0, 125.4, 42.5, 29.1, 22.8
1.79 + 1.65	

δ (^{1}H)/ppm	δ (^{13}C)/ppm
1.70	211.7, 45.4, 29.1
1.60	148.0, 110.9, 45.4
1.53	134.7, 125.4, 28.1, (19.7)

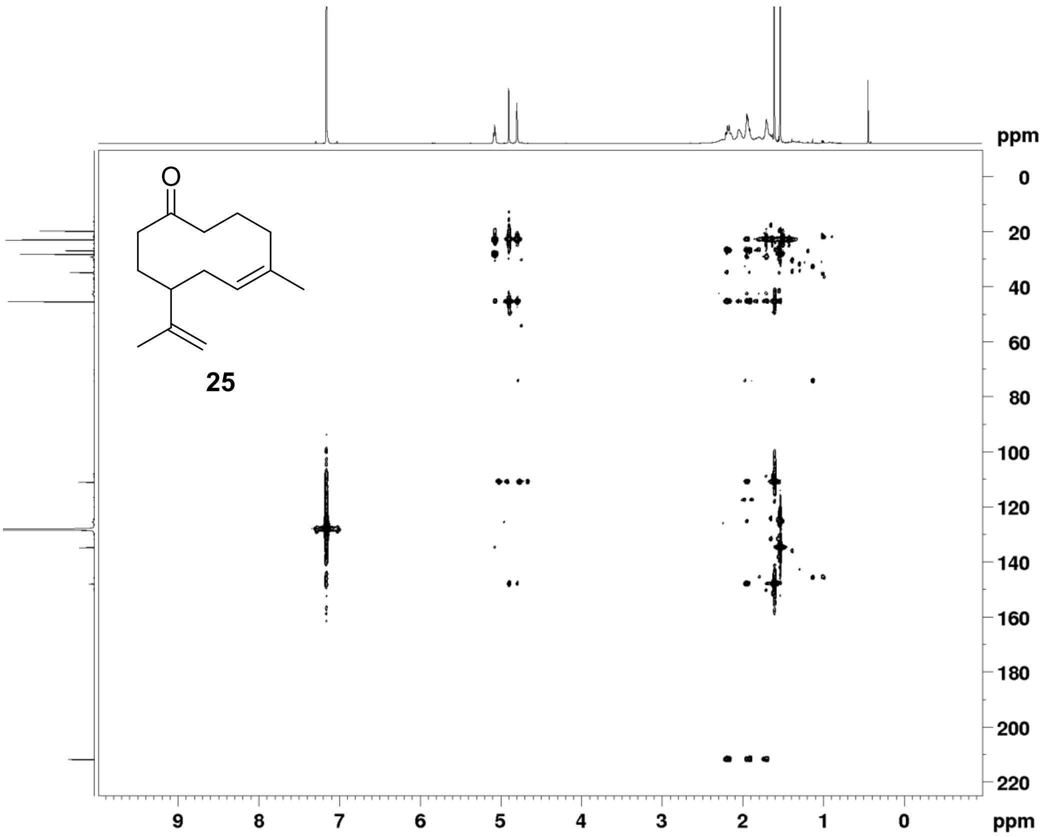

Figure S18. ^{1}H-^{13}C HMBC NMR spectrum of compound **25** in C_6D_6.

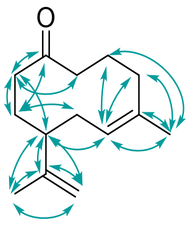

25

Figure S19. Key ^{1}H-^{13}C HMBC NMR correlations of **25** as indicated by green arrows.

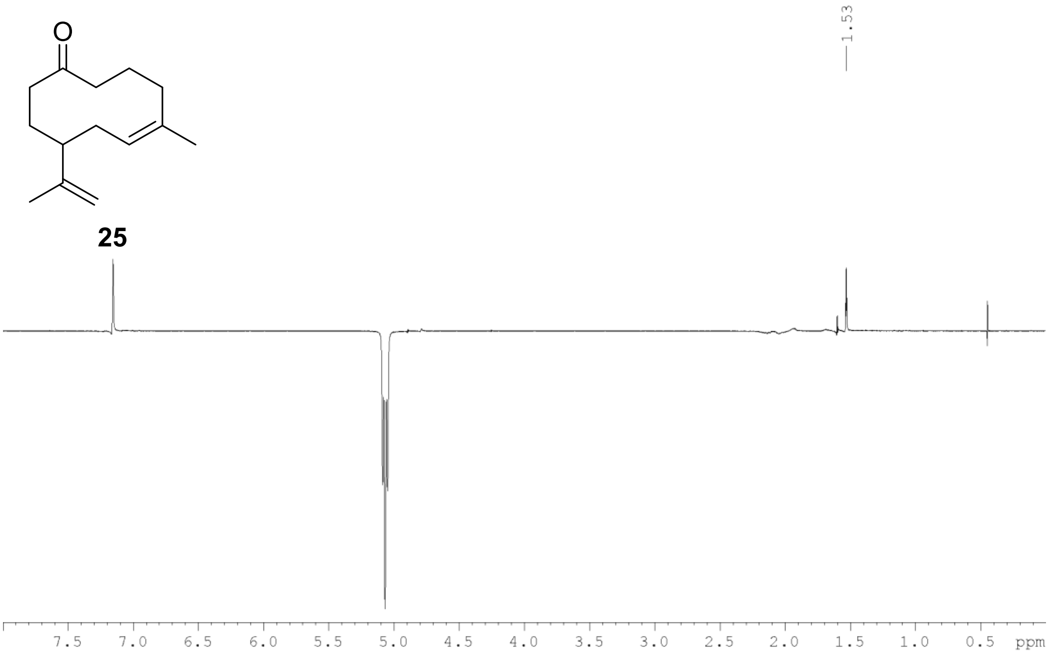

Figure S20. ^{1}H NOE NMR of signal at 5.07 ppm.

Since the olefinic signal at 5.07 ppm shows a strong 1D ^{1}H NOE correlation to the methyl group at 1.53 ppm, the double bond geometry is assigned as *cis*.

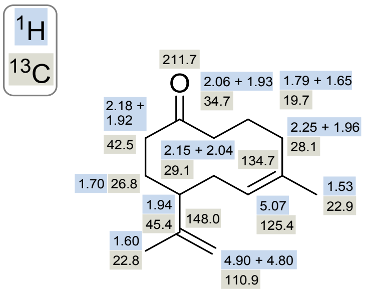

25

Figure S21. Full assignment of chemical shifts for compound **25**.

1.5.3 Structure elucidation of 28a

To simplify structure elucidation only the centre of each HSQC signal will be used as the chemical shift, despite different multiplicities, since overlapping signals can be differentiated more easily.

Table S8. ^{1}H NMR signals and their corresponding ^{13}C NMR signals for compound **28a** as analysed with the support of ^{1}H-^{13}C HSQC and $^{13}C\{^{1}H\}$ DEPT135 experiments. The quaternary carbon atoms are listed at the bottom.

δ (^{1}H)/ppm	δ (^{13}C)/ppm	DEPT135/HSQC phase
2.06 + 1.28	33.8	CH_2
2.05	33.2	CH/CH_3
1.99 + 1.37	18.1	CH_2

δ (^{1}H)/ppm	δ (^{13}C)/ppm	DEPT135/HSQC phase
1.95 + 1.36 – 1.28	30.3	CH_2
1.80 + 1.06	38.8	CH_2
1.39 + 1.22	32.8	CH_2
1.35 + 1.20	37.3	CH_2
1.28	51.0	CH/CH_3
1.07	27.8	CH/CH_3
0.95	18.4	CH/CH_3
0.93	18.2	CH/CH_3
	90.3	C_q
	85.2	C_q
3.44	70.0	C_{OH}

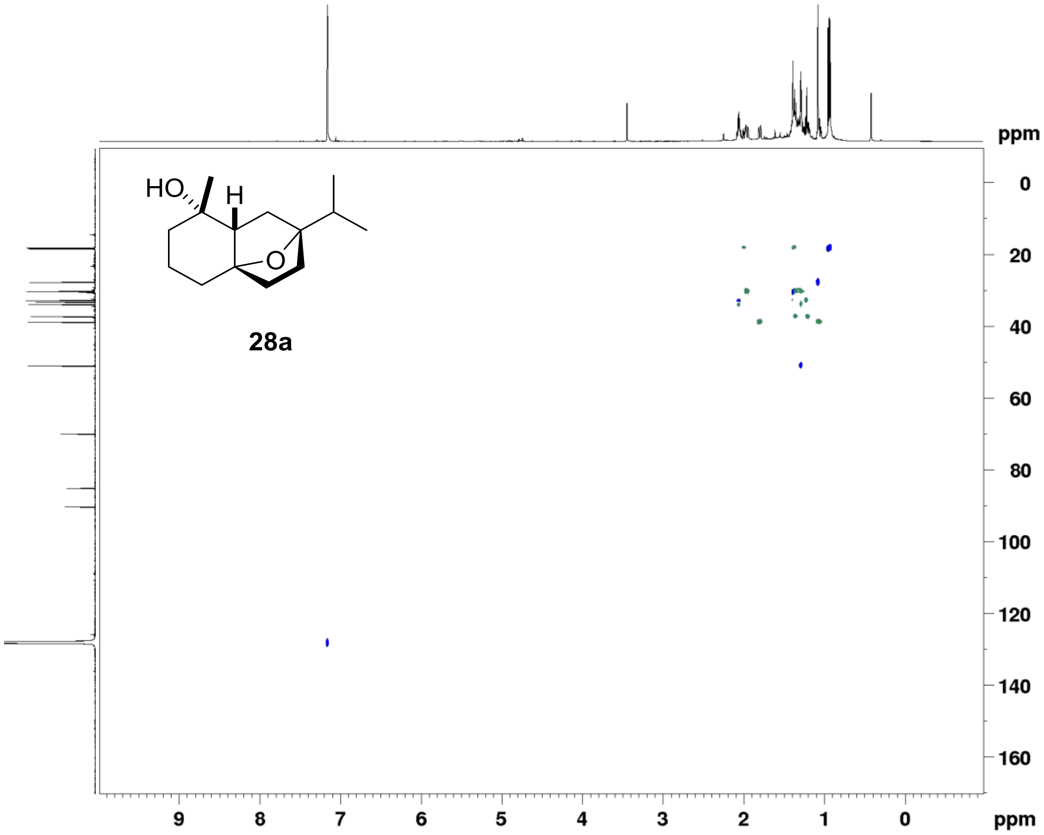

Figure S22. ^{1}H-^{13}C HSQC NMR spectrum of compound **28a** in C_6D_6 (pos. phase = blue (CH/CH_3), neg. phase = green (CH_2)).

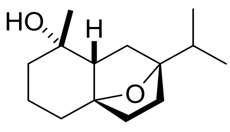

28a

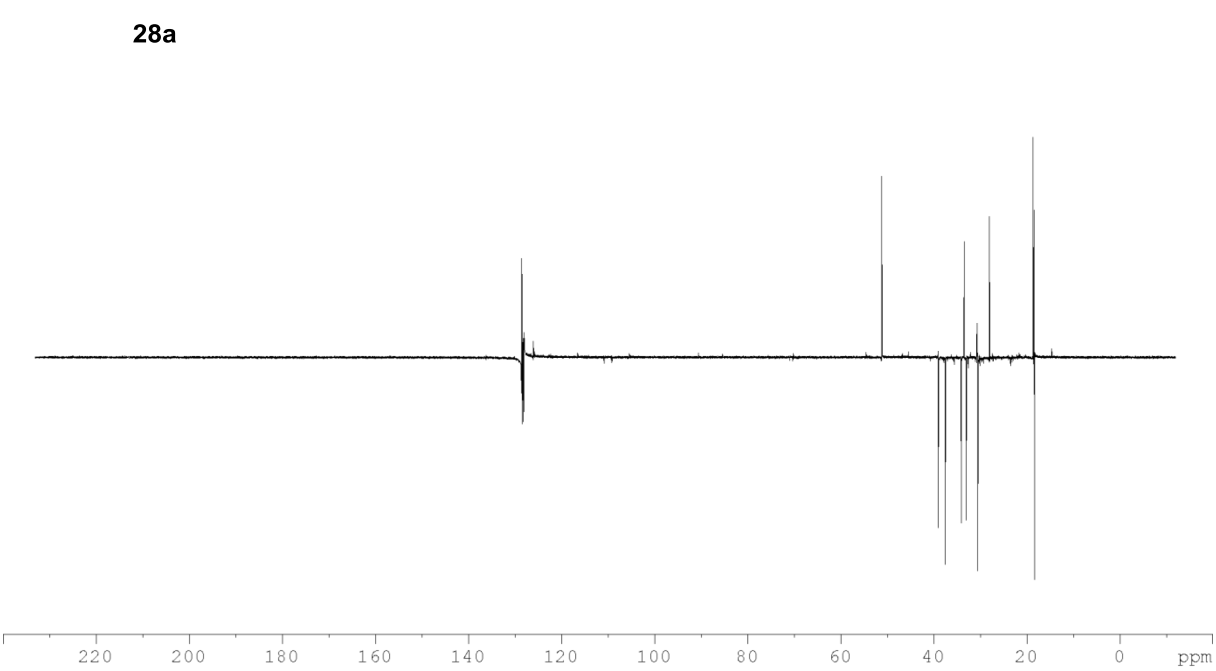

Figure S23. $^{13}C\{^{1}H\}$ DEPT135 NMR spectrum of compound **28a** in C_6D_6.

Table S9. ^{1}H NMR signals and the corresponding ^{1}H-^{1}H COSY correlations for compound **28a**. Signals with weak intensities are given in parentheses. Superposing signals which cannot be distinguished, are marked with "x".

δ (^{1}H)/ppm	COSY correlations
2.06 + 1.28 and 1.28	1.95, 1.3x
2.05	0.95, 0.93, (1.39)
1.99 + 1.37	1.80, 1.06
1.95 + 1.36 – 1.28	1.80, 1.3x, 1.2x,
1.80 + 1.06	1.9x, 1.3x
1.39 + 1.22	(2.05)
1.35 + 1.20	likely 1.39
1.07	3.44 (OH group)
0.95	2.05
0.93	2.05

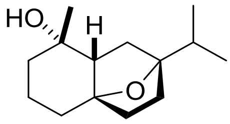

28a

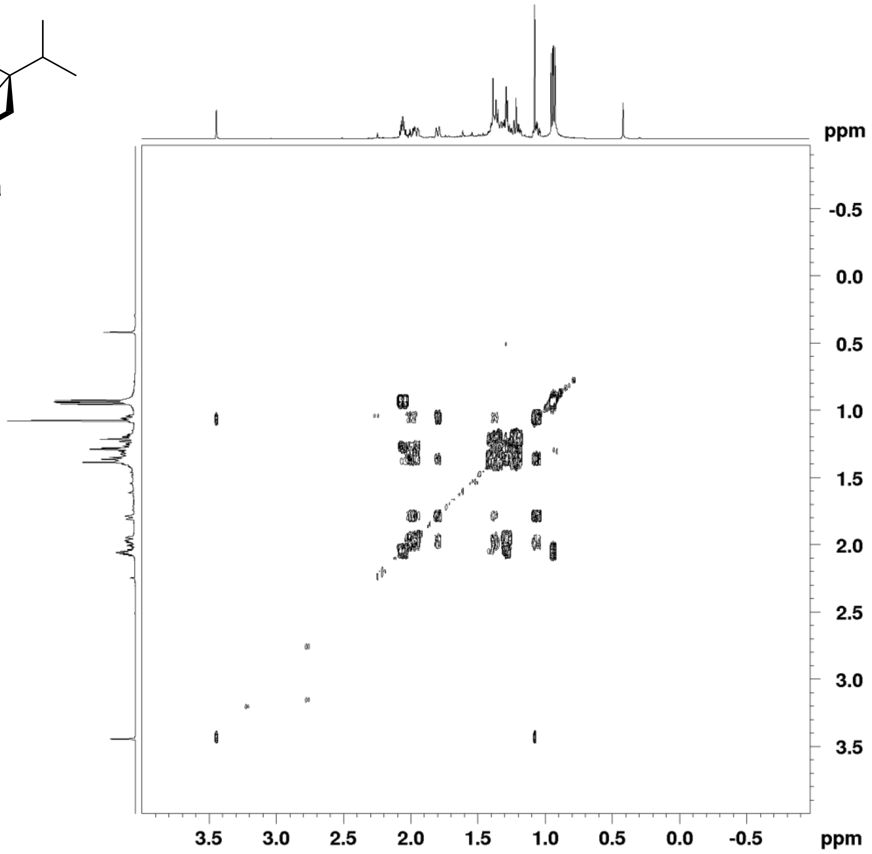

Figure S24. ^{1}H-^{1}H COSY NMR spectrum of compound **28a** in C_6D_6.

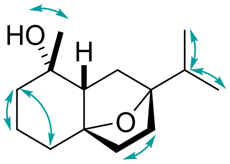

28a

Figure S25. Key ^{1}H-^{1}H COSY NMR correlations of **28a** as indicated by arrows.

Table S10. Selected correlations between ^{13}C NMR signals and neighbouring ^{1}H NMR signals as collected from the ^{1}H-^{13}C HMBC spectrum of compound **28a**. Note: Signals with weak intensities are given in parentheses.

δ (^{1}H)/ppm	δ (^{13}C)/ppm
3.44	70.0, 51.0. 27.8
2.06 + 1.28	70.0, 51.0, 33.2/32.8,
2.05	90.3, 33.8, 18.4, 18.2
1.9x	85.2, 51.0, 38.8, 33.2, 18.1
1.80 + 1.06	70.0, 51.0, 30.3, (18.1)
1.39 + 1.22	Hard to distinguish
1.35 + 1.20	Hard to distinguish
both 1.28	Hard to distinguish

δ (^{1}H)/ppm	δ (^{13}C)/ppm
1.07	70.0, 51.0, 38.8
0.95	90.3, 33.2, 18.2
0.93	90.3, 33.2, 18.4

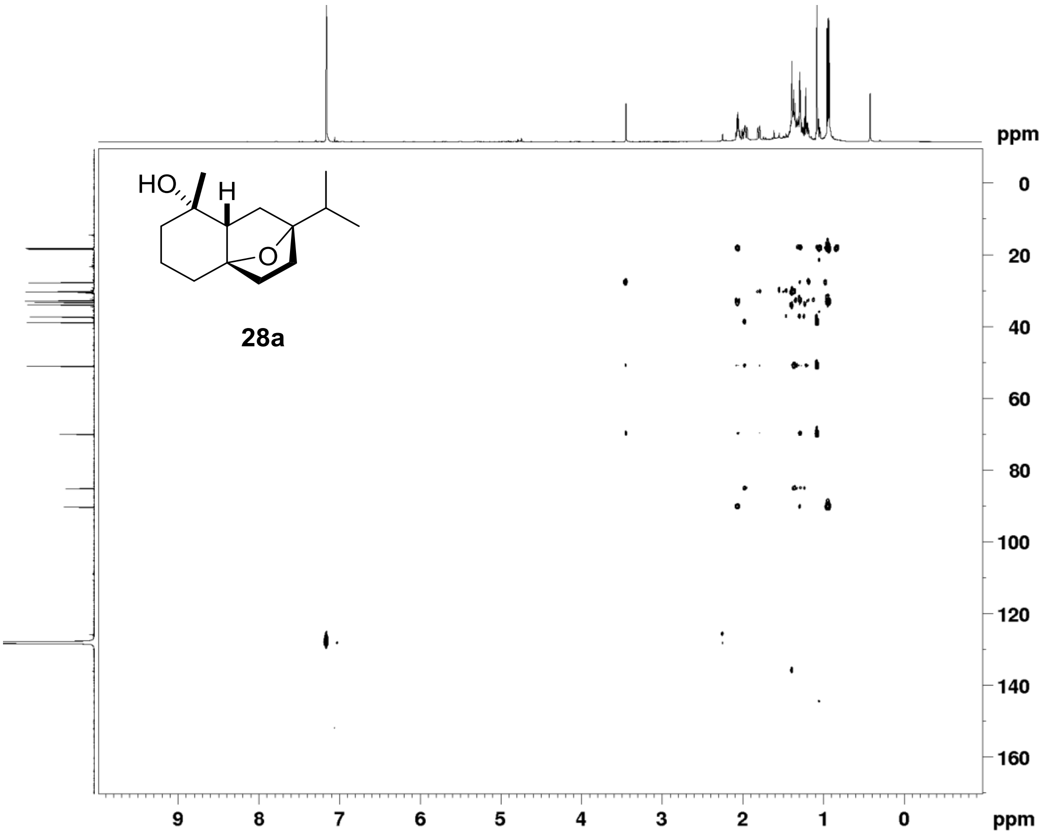

Figure S26. ^{1}H-^{13}C HMBC NMR spectrum of compound **28a** in C_6D_6.

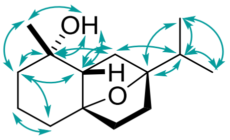

28a

Figure S27. Key ^{1}H-^{1}H COSY NMR correlations of **28a** as indicated by arrows.

^{1}H-^{13}C HMBC correlations are hard to distinguish for superposed signals. Especially information towards the two bridge CH_2 groups is rare. Elucidation also relies on knowledge of **28b**.

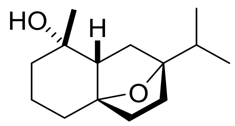

28a

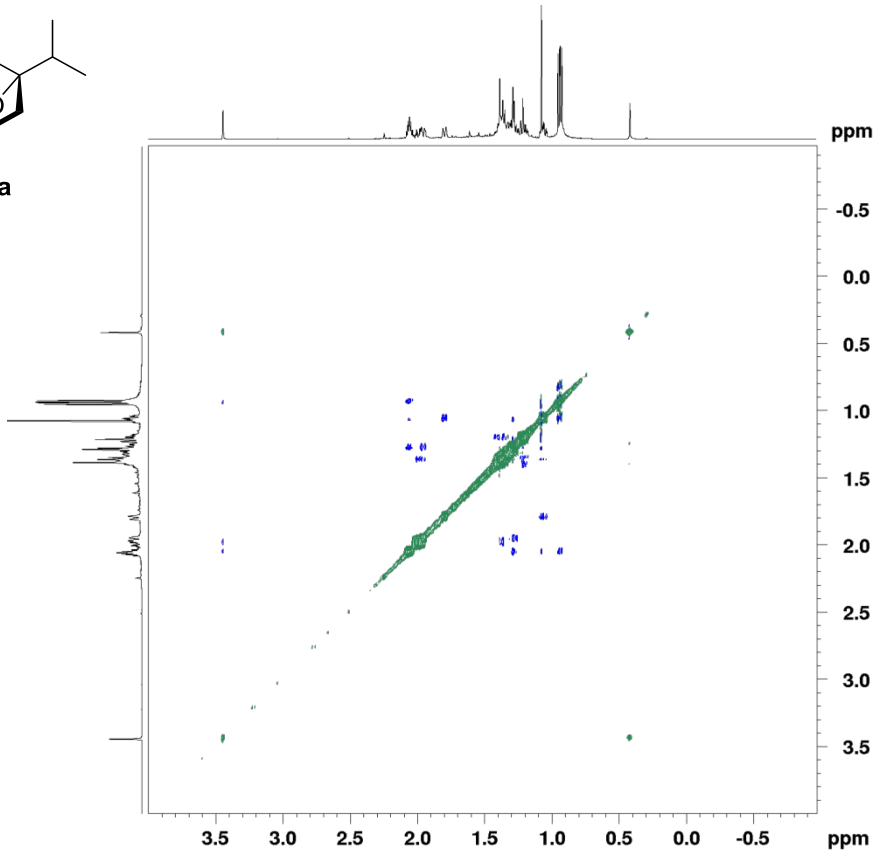

Figure S28. ^1H-^1H NOESY NMR spectrum of compound **28a** in C_6D_6.

^1H-^1H NOESY NMR spectra shows no correlation between alcohol proton (3.44 ppm) and CH group or proton of vicinal CH_2 (1.28 ppm), instead there is a correlation between the methyl group (1.07 ppm) and 1.28 ppm. This indicates that the methyl group (1.07 ppm) and the CH group (1.28 ppm) are *syn* orientated. The alcohol (3.44 ppm) shows correlations to one methyl group of the *iso*-propyl group (0.95 ppm), the geminal methyl group (1.07 ppm), and two different ring CH_2 protons (1.99 ppm and 2.06 ppm).

Assuming the first analysis of the *syn*-orientation for the methyl group (1.07 ppm) and the CH group (1.28 ppm), two diastereomers are possible in which only one can explain especially the signal between the alcohol proton (3.44 ppm) and the methyl group (0.95 ppm).

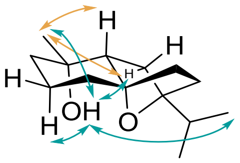

28a

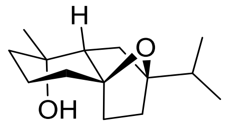

I

Figure S29. Different diastereomers of **28a** and **I**. Stereochemistry of **28a** fits ^1H-^1H NOESY NMR data best. Correlations above ring marked in orange, correlations below ring marked in green.

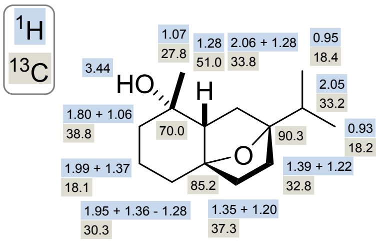

28a

Figure S30. Full assignment of chemical shifts for compound **28a**.

1.5.4 Structure elucidation of 26b

To simplify structure elucidation only the centre of each HSQC signal will be used as the chemical shift, despite different multiplicities, since overlapping signals can be differentiated more easily.

Table S11. ^{1}H NMR signals and their corresponding ^{13}C NMR signals for compound **26b** as analysed with the support of ^{1}H-^{13}C HSQC and ^{13}C{^{1}H} DEPT135 experiments. The quaternary carbon atoms are listed at the bottom.

δ (^{1}H)/ppm	δ (^{13}C)/ppm	DEPT135/HSQC phase
4.76	108.9	CH_2
4.74	110.7	CH_2
2.07	54.3	CH/CH_3
1.93	30.3	CH_2
1.85	45.2	CH/CH_3
1.72 + 1.48	40.4	CH_2
1.67 + 1.16	30.8	CH_2
1.63 + 1.43	23.2	CH_2
1.61	21.2	CH/CH_3
1.57 + 1.23	29.1	CH_2
1.54 + 1.49	35.4	CH_2
	150.3	C_q
	149.5	C_q
1.40	71.8	C_q

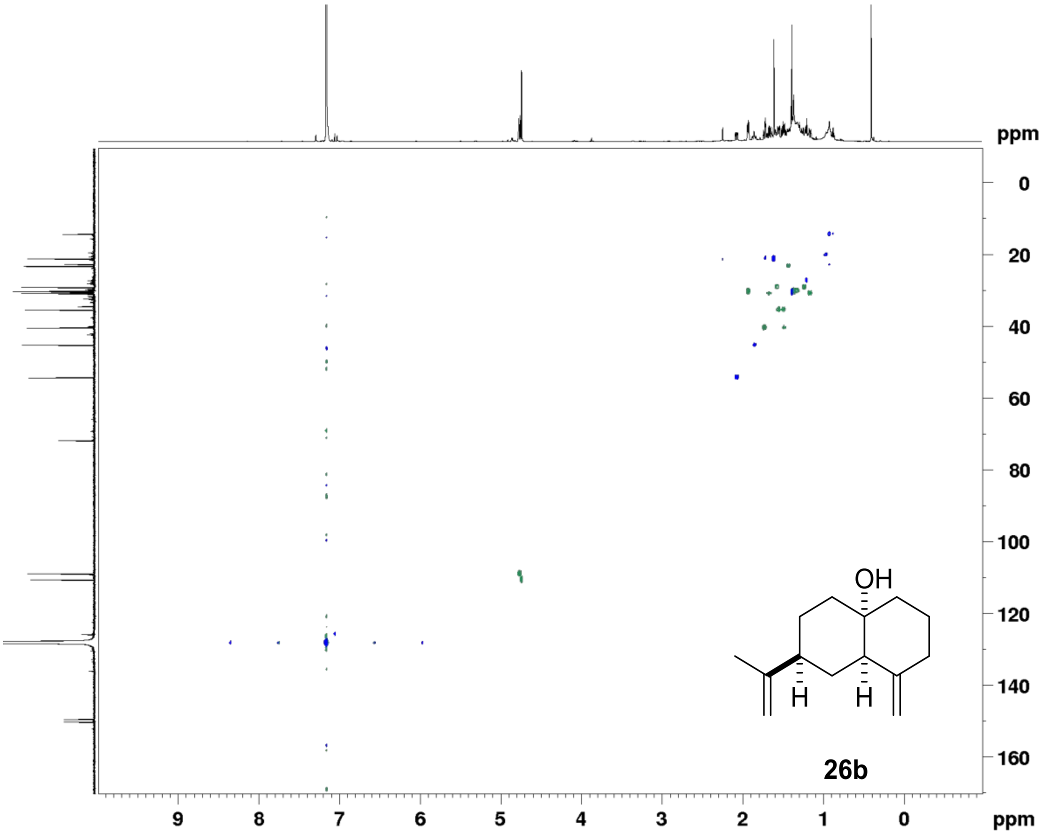

Figure S31. ^{1}H-^{13}C HSQC NMR spectrum of compound **26b** in C_6D_6 (pos. phase = blue (CH/CH_3), neg. phase = green (CH_2)).

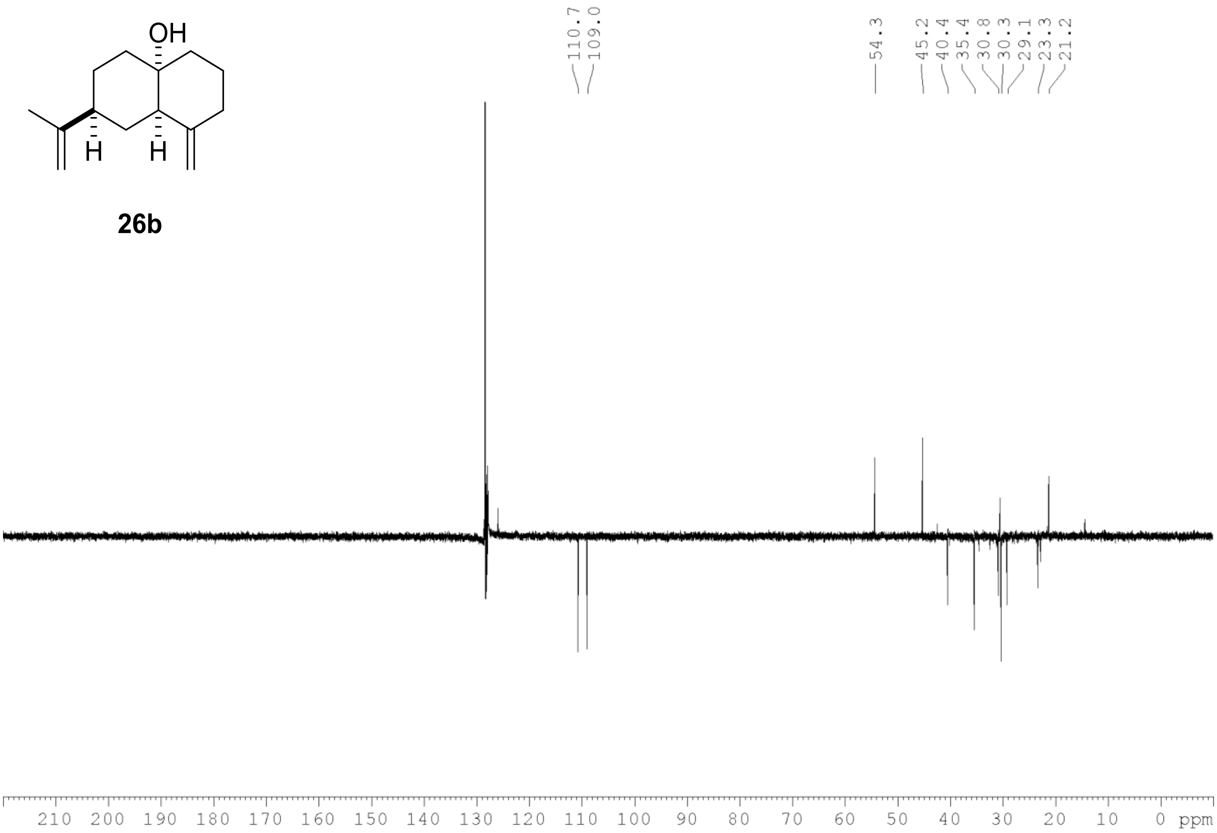

Figure S32. ^{13}C{^{1}H} DEPT135 NMR spectrum of compound **26b** in C_6D_6.

Table S12. ¹H NMR signals and the corresponding ¹H-¹H COSY correlations for compound **26b**. Signals with weak intensities are given in parentheses. Superposing signals which cannot be distinguished, are marked with "x".

δ (¹H)/ppm	COSY correlations
4.76	1.61
4.74	1.93
2.07	1.54 + 1.49, 1.16
1.93	4.74, 1.6x, 1.43, 1.16
1.85	4.76, 1.57 + 1.23, 1.54 + 1.49
1.72 + 1.48	1.57 + 1.23
1.67 + 1.16	1.93, 1.43, (2.07)
1.63 + 1.43	1.93
1.61	4.76
1.57 + 1.23	1.85, 1.72 + 1.48
1.54 + 1.49	2.07, 1.85

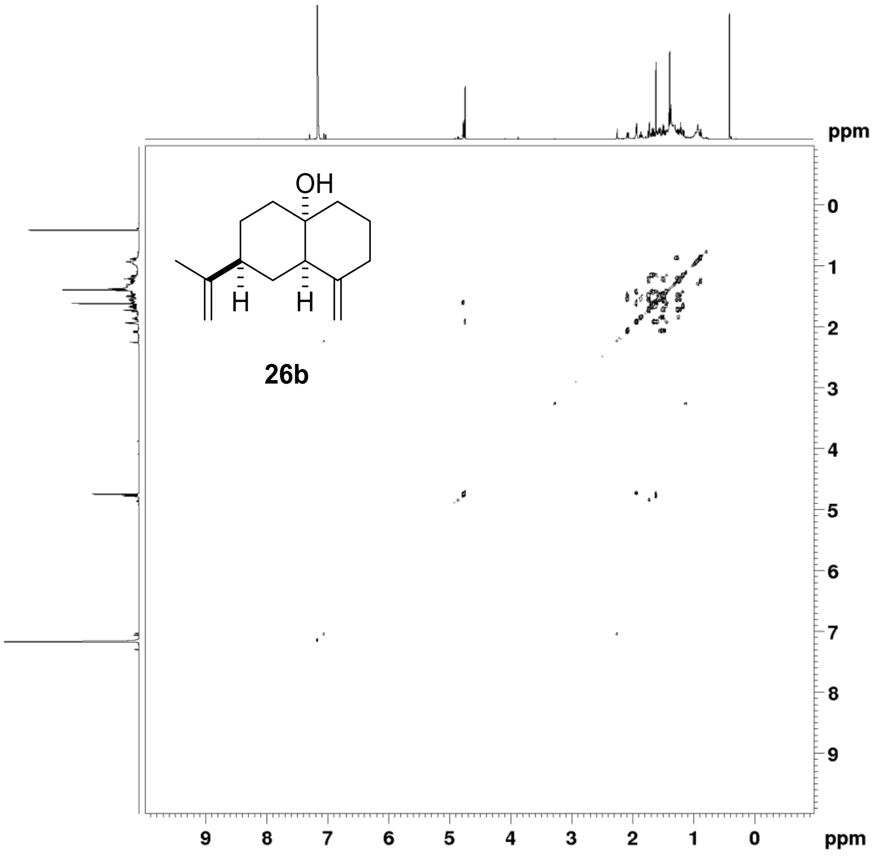

Figure S33. ¹H-¹H COSY NMR spectrum of compound **26b** in C_6D_6.

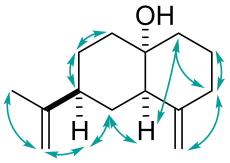

26b

Figure S34. Key ^{1}H-^{1}H COSY NMR correlations of **26b** as indicated by arrows.

Table S13. Selected correlations between ^{13}C NMR signals and neighbouring ^{1}H NMR signals as collected from the ^{1}H-^{13}C HMBC spectrum of compound **26b**. Note: Signals with weak intensities are given in parentheses.

δ (^{1}H)/ppm	δ (^{13}C)/ppm
4.76	150.3, 54.3, 30.3
4.74	149.5, 45.2, 21.2
2.07	150.3, 110.7, 71.8, 30.x, (35.4)
1.93	150.3, 110.7, 54.3, 30.8, 23.2
1.85	149.5
1.72 + 1.48	71.8, 54.3, 45.2
1.67 + 1.16	71.8, 54.3, 30.3, 23.2
1.63 + 1.43	Hard to distinguish
1.61	108.9, 149.5, 45.2
1.57 + 1.23	Hard to distinguish
1.54 + 1.49	Hard to distinguish
1.40	40.4, (71.8)

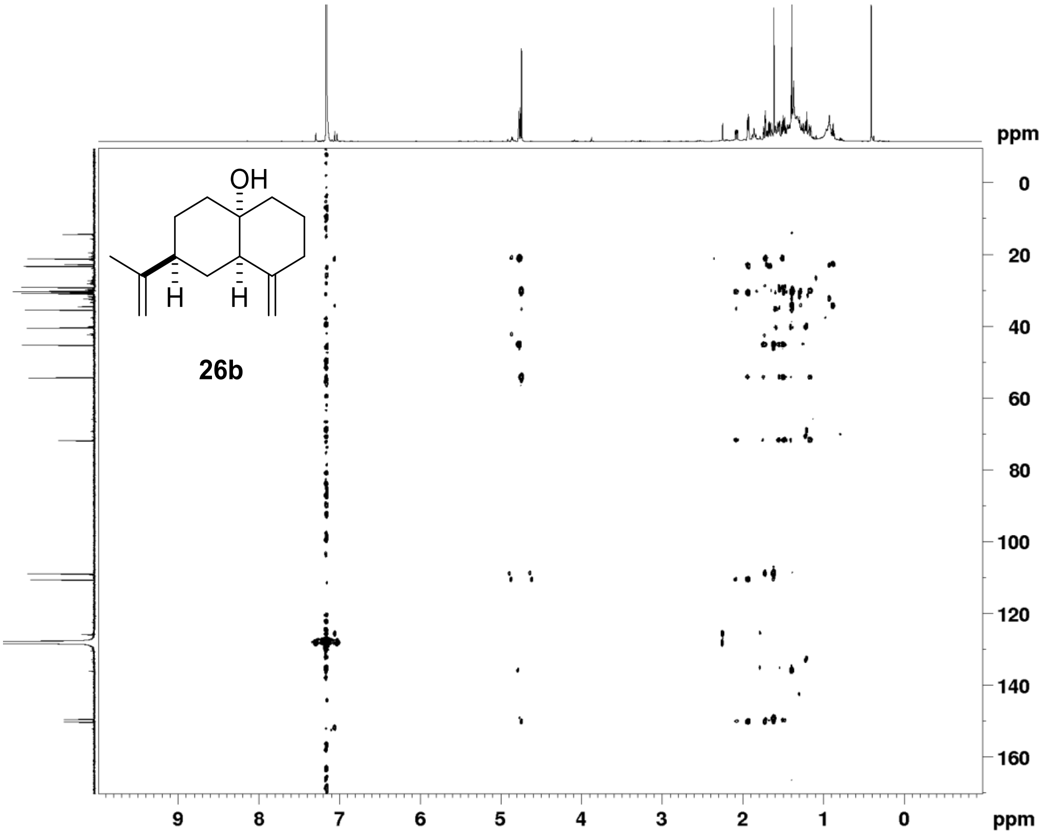

Figure S35. ^1H-^{13}C HMBC NMR spectrum of compound **26b** in C_6D_6.

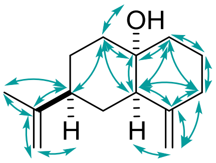

26b **Figure S36**. Key ^1H-^{13}C HMBC NMR correlations of **26b** as indicated by arrows.

The correlation between two CH groups (1.85 ppm and 2.07 ppm) shows a *syn*-orientation. A minor NOESY correlation between the CH group (2.07 ppm) and the alcohol proton (1.40 ppm) leads to a all *syn*-orientation for the three protons.

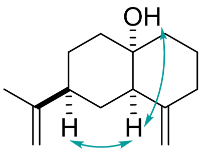

26b **Figure S37**. Key ^1H-^1H NOESY NMR correlations of **26b** as indicated by arrows.

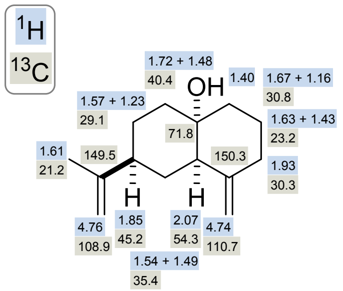

26b

Figure S38. Full assignment of chemical shifts for compound **26b**.

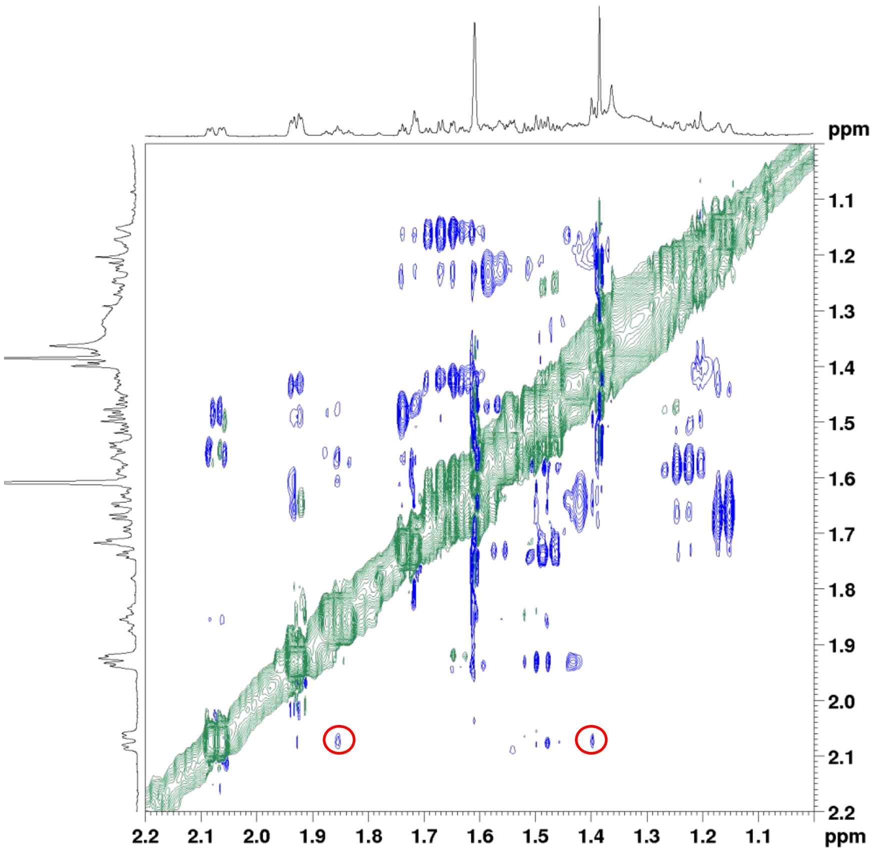

Figure S39. Zoom-in of ^{1}H-^{1}H NOESY NMR spectrum of compound **26b** in C_6D_6. Key correlations for 2.07 ppm marked with red circles.

1.5.5 Structure elucidation of 26a

To simplify structure elucidation only the centre of each HSQC signal will be used as the chemical shift, despite different multiplicities, since overlapping signals can be differentiated more easily.

Table S14. ^{1}H NMR signals and their corresponding ^{13}C NMR signals for compound **26a** as analysed with the support of ^{1}H-^{13}C HSQC. The quaternary carbon atoms are listed at the bottom.

δ (^{1}H)/ppm	δ (^{13}C)/ppm	HSQC phase
5.24	120.5	CH/CH_3
4.76	109.0	CH_2
2.14 + 1.88	22.3	CH_2
1.95 + 0.97	36.4	CH_2
1.87	45.5	CH/CH_3
1.77 + 1.66	40.1	CH_2
1.73 + 1.15	27.3	CH_2
1.71	49.5	CH/CH_3
1.61	20.9	CH/CH_3
1.57 + 1.21	29.0	CH_2
1.56	22.4	CH/CH_3
	149.7	C_q
	135.2	C_q
1.52	69.8	C_q

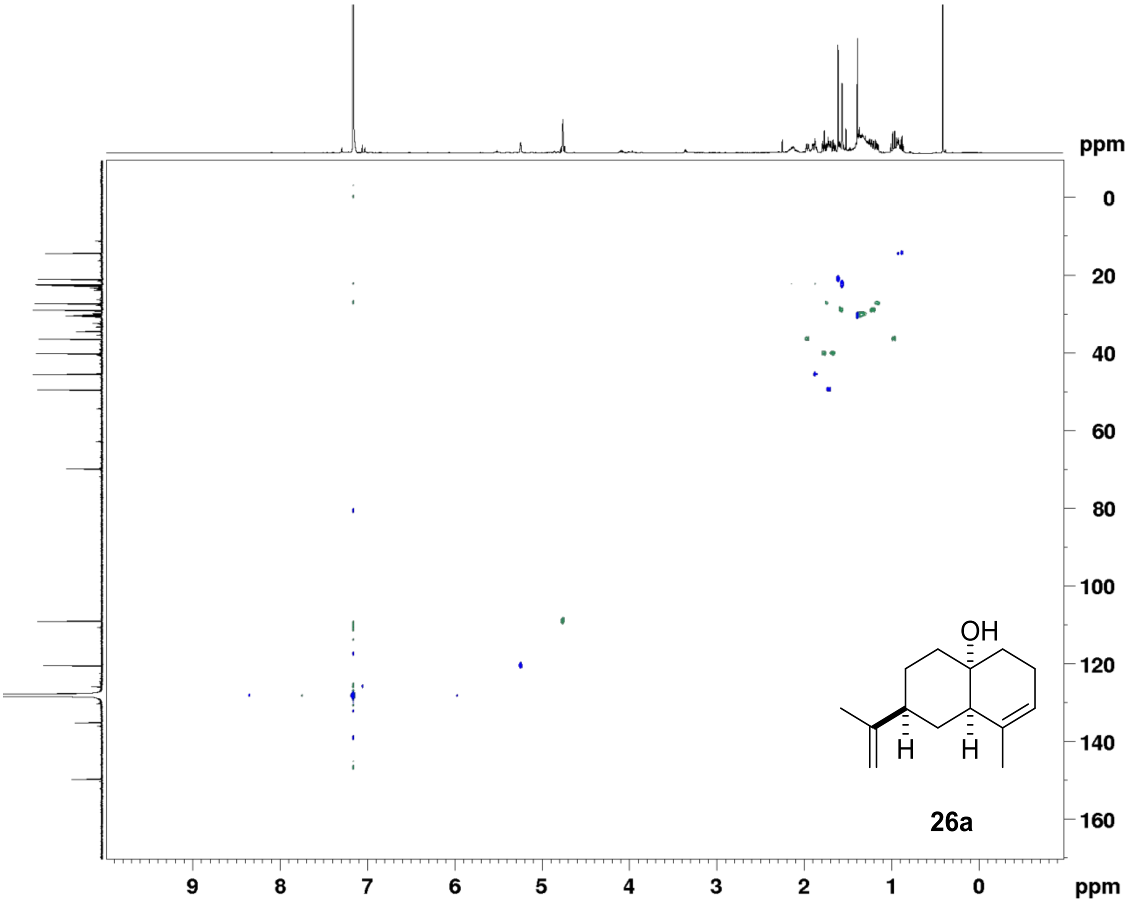

Figure S40. ^{1}H-^{13}C HSQC NMR spectrum of compound **26a** in C_6D_6 (pos. phase = blue (CH/CH_3), neg. phase = green (CH_2)).

Table S15. ^{1}H NMR signals and the corresponding ^{1}H-^{1}H COSY correlations for compound **26a**. Signals with weak intensities are given in parentheses. Superposing signals which cannot be distinguished, are marked with “x”.

δ (^{1}H)/ppm	COSY correlations
5.24	2.14, 1.8x, 1.56
4.76	1.61
2.14 + 1.88	5.24, 1.73, 1.56, 1.15
1.95 + 0.97	1.8x, 1.71, 1.57
1.87	4.76, 1.21, 0.97
1.77 + 1.66	1.57 + 1.21
1.73 + 1.15	2.14, 1.8x
1.71	1.95, 0.97
1.61	4.76
1.57 + 1.21	1.8x, 1.77, 1.66, (1.95)
1.56	5.24, 1.88, 2.14

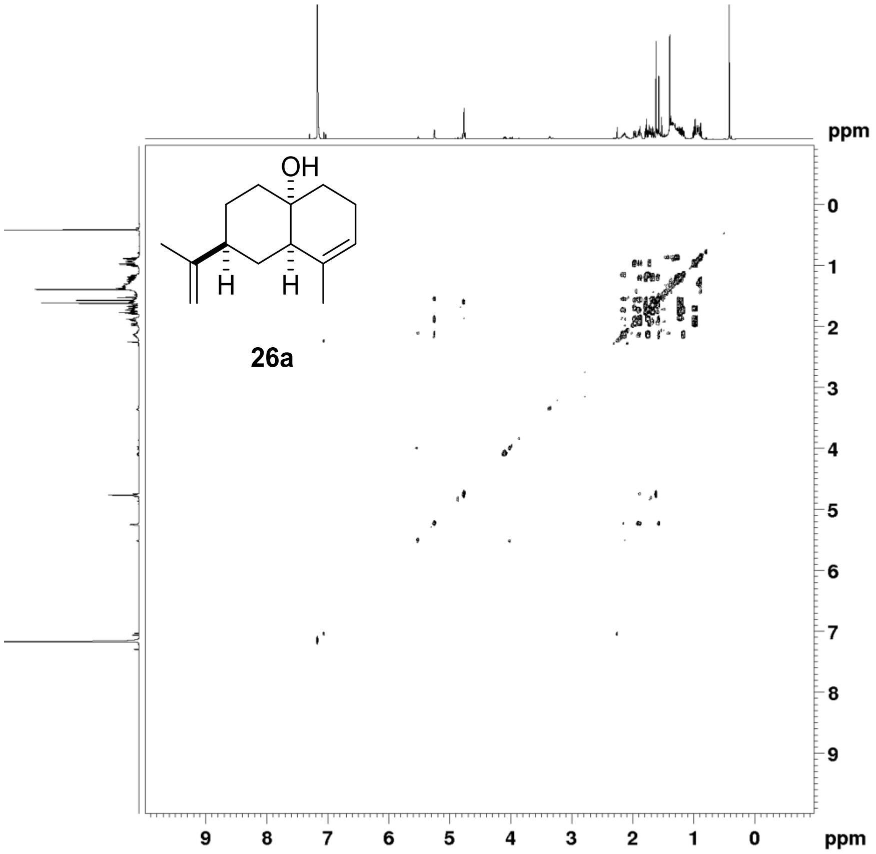

Figure S41. ^{1}H-^{1}H COSY NMR spectrum of compound **26a** in C_6D_6.

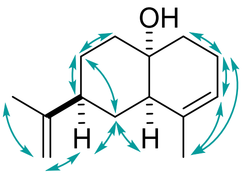

26a

Figure S42. Key ^{1}H-^{1}H COSY NMR correlations of **26a** as indicated by arrows.

Table S16. Selected correlations between ^{13}C NMR signals and neighbouring ^{1}H NMR signals as collected from the ^{1}H-^{13}C HMBC spectrum of compound **26a**. Note: Signals with weak intensities are given in parentheses.

δ (^{1}H)/ppm	δ (^{13}C)/ppm
5.24	49.5, 27.3, 22.4
4.76	45.5, 20.9
2.14 + 1.88	Hard to distinguish
1.95 + 0.97	69.8, 49.5, 45.5, 29.0
1.87	Hard to distinguish
1.77 + 1.66	69.8, 49.5, 45.5, 27.3
1.73 + 1.15	120.5, 69.8, 49.5, 40.1

δ (^{1}H)/ppm	δ (^{13}C)/ppm
1.71	135.2, 36.4, 22.x
1.61	149.7, 109.0, 45.5
1.57 + 1.21	69.8, 45.5, 40.1
1.56	135.2, 120.5, 49.5

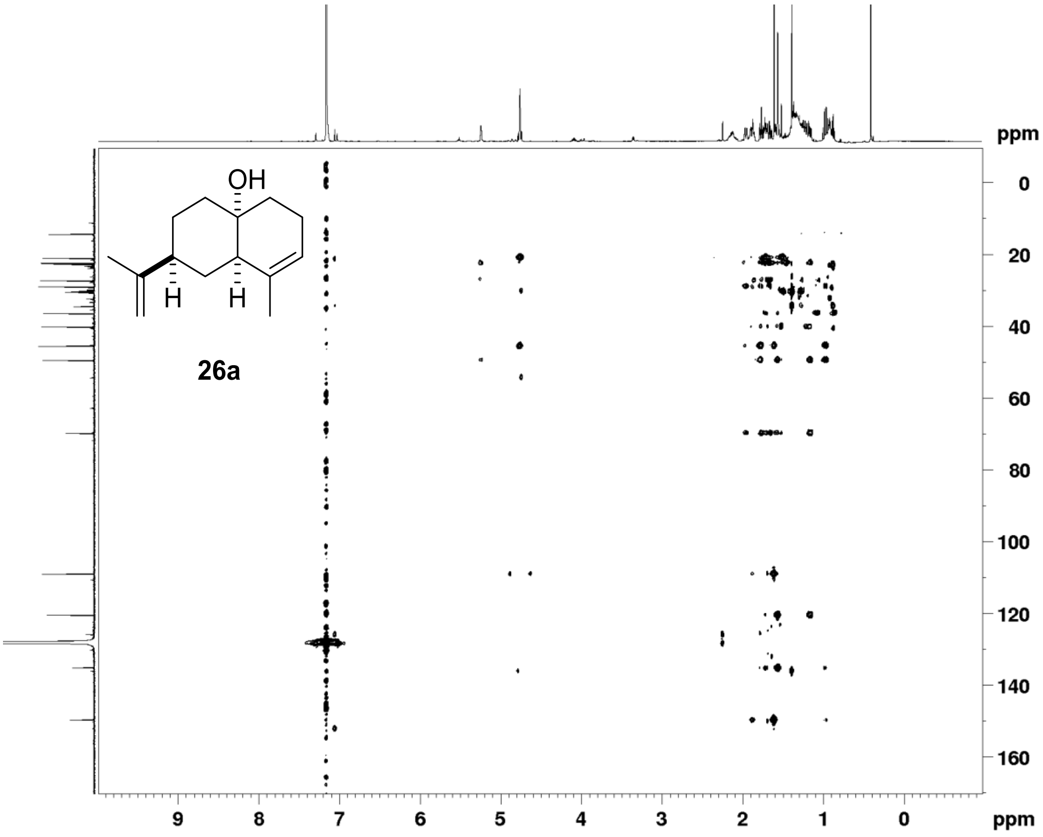

Figure S42. ^{1}H-^{13}C HMBC NMR spectrum of compound **26a** in C_6D_6.

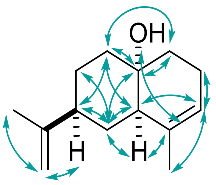

26a

Figure S43. Key ^{1}H-^{13}C HMBC NMR correlations of **26a** as indicated by arrows.

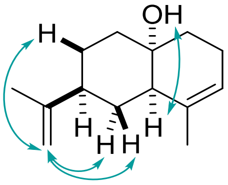

26a

Figure S44. Key ^{1}H-^{1}H NOESY NMR correlations of **26a** as indicated by arrows.

^{1}H-^{1}H NMR NOESY correlation between the alcohol proton (1.52 ppm) and CH group (1.71 ppm) leads to a *syn*-orientation. Area around 1.87 ppm and 1.71 ppm shows dispersive signals which do not allow for interpretation of spatial proximity. The protons at 0.97 ppm and 1.21 ppm show strong *J*-couplings, which indicate their axial position with axial vicinal protons.

^{1}H-^{1}H NMR NOESY signals of interest: 1.73 ppm to 0.97 ppm, 1.52 ppm to 2.14 ppm, 2.14 ppm to 1.15 ppm, 1.77 ppm to 1.15 ppm, 0.97 ppm to 1.21 ppm, 1.56 ppm to 1.95 ppm, 1.21 ppm to 1.73 ppm, 1.57 ppm to 1.87 ppm, 4.76 ppm to 0.97 ppm, 4.76 ppm to 1.95 ppm, 4.76 ppm to 1.21 ppm.

Compared to the literature of a similar compound, we are not able to observe the analogous correlation between 4.76 ppm and 1.71 ppm (this work, 4.87 ppm and 2.14 in literature). [S13] Instead, we can see several correlations of 4.76 ppm which would fit for the structure shown above.

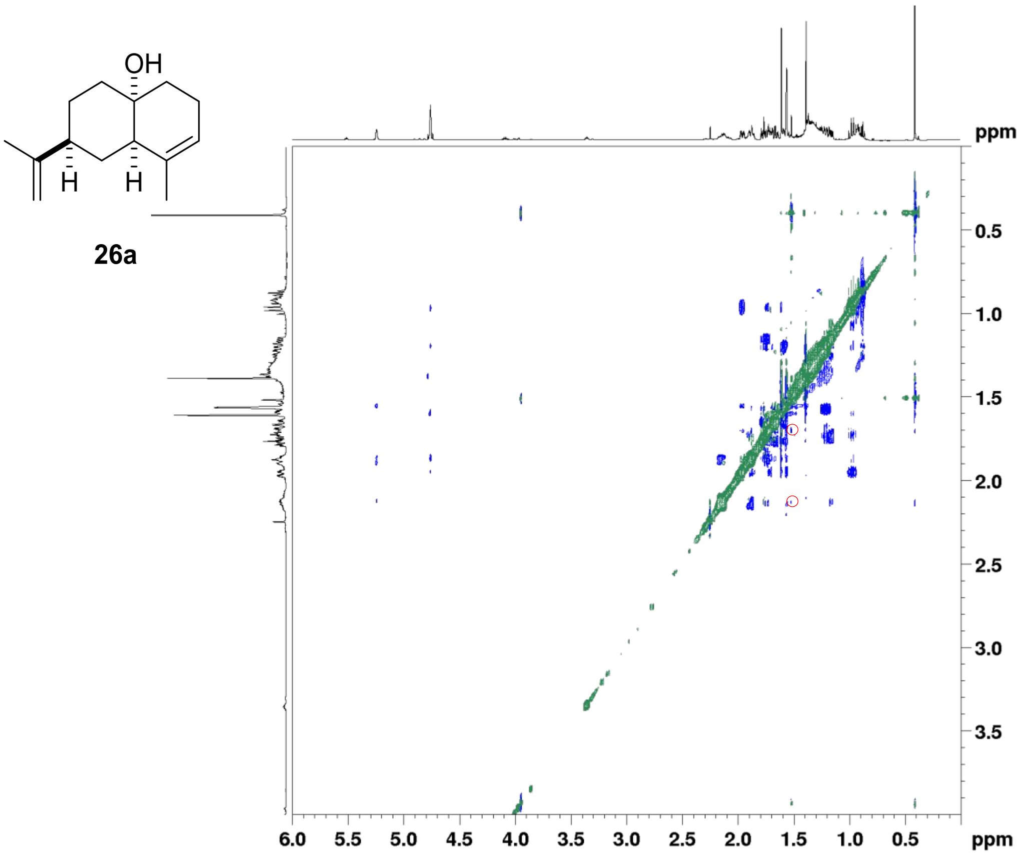

Figure S45. Zoom-in of ^{1}H-^{1}H NOESY NMR spectrum of compound **26a** in C_6D_6. Key correlations for 1.52 ppm marked with red circles.

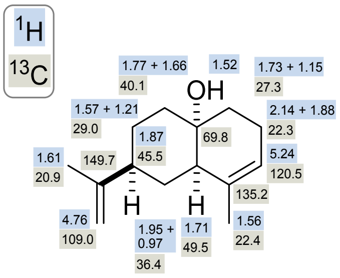

26a

Figure S46. Full assignment of chemical shifts for compound **26a**.

1.5.6 Structure elucidation of 27b

To simplify structure elucidation only the centre of each HSQC signal will be used as the chemical shift, despite different multiplicities, since overlapping signals can be differentiated more easily.

Due to ^{1}H NMR superposing singals, structure elucidation is heavily based on ^{1}H-^{13}C HMBC and ^{1}H-^{1}H NOESY correlations, as ^{1}H-^{1}H COSY NMR is hard to interpret due to large overlaying signals.

Table S17. ^{1}H NMR signals and their corresponding ^{13}C NMR signals for compound **27b** as analysed with the support of ^{1}H-^{13}C HSQC. The quaternary carbon atoms are listed at the bottom.

δ (^{1}H)/ppm	δ (^{13}C)/ppm	HSQC phase
4.88 + 4.82	109.0	CH_2
4.78 + 4.58	108.5	CH_2
2.15 + 1.79	36.8	CH_2
1.84 + 1.51	26.8	CH_2
1.83	45.5	CH/CH_3
1.77 + 1.16	39.0	CH_2
1.73	49.5	CH/CH_3
1.71	21.0	CH/CH_3
1.56 + 1.13	39.8	CH_2
1.55	29.5	CH_2
1.48	14.0	CH_2
	150.4	C_q
	149.9	C_q
	71.1	C_q

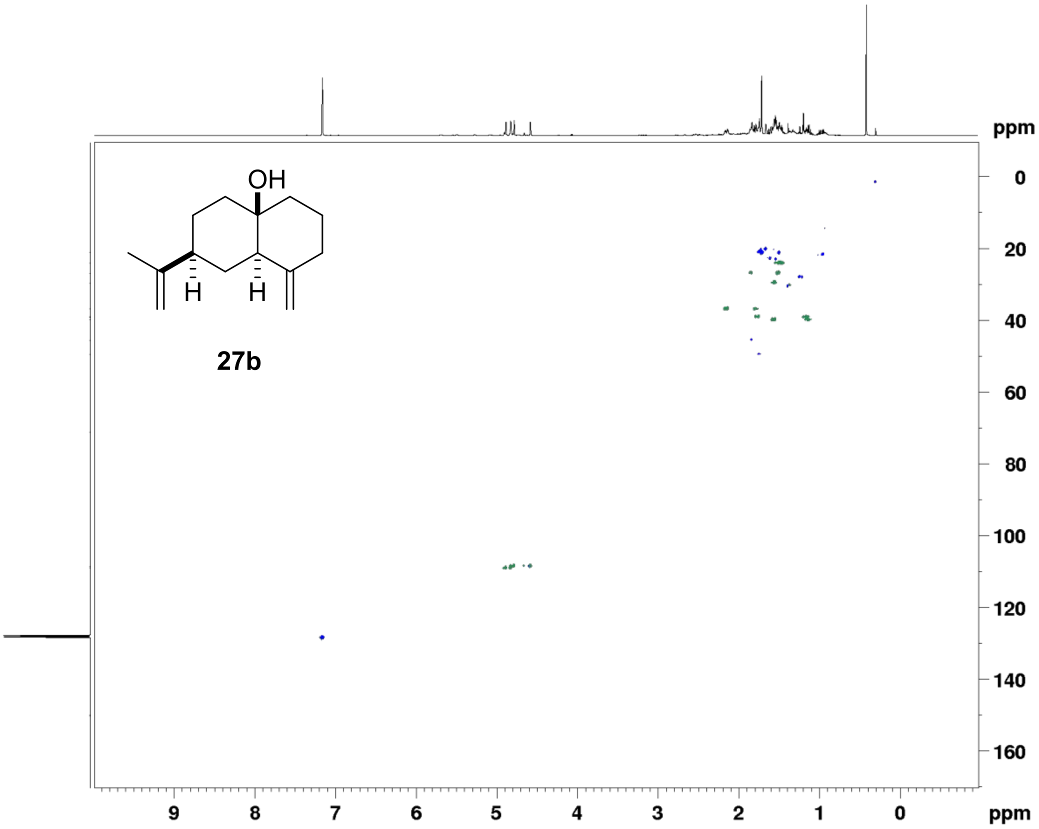

Figure S47. ^1H-^{13}C HSQC NMR spectrum of compound **27b** in C_6D_6 (pos. phase = blue (CH/CH_3), neg. phase = green (CH_2)).

Table S18. ^1H NMR signals and the corresponding ^1H-^1H COSY correlations for compound **27b**. Signals with weak intensities are given in parentheses. Superposing signals which cannot be distinguished, are marked with “x”.

δ (^1H)/ppm	COSY correlations
4.88 + 4.82	1.71
4.78 + 4.58	1.73
2.15 + 1.79	1.56, 1.48
1.84 + 1.51	Hard to distinguish
1.83	Hard to distinguish
1.77 + 1.16	1.84
1.73	Hard to distinguish
1.71	Hard to distinguish
1.56 + 1.13	1.48
1.55	Hard to distinguish
1.48	2.15

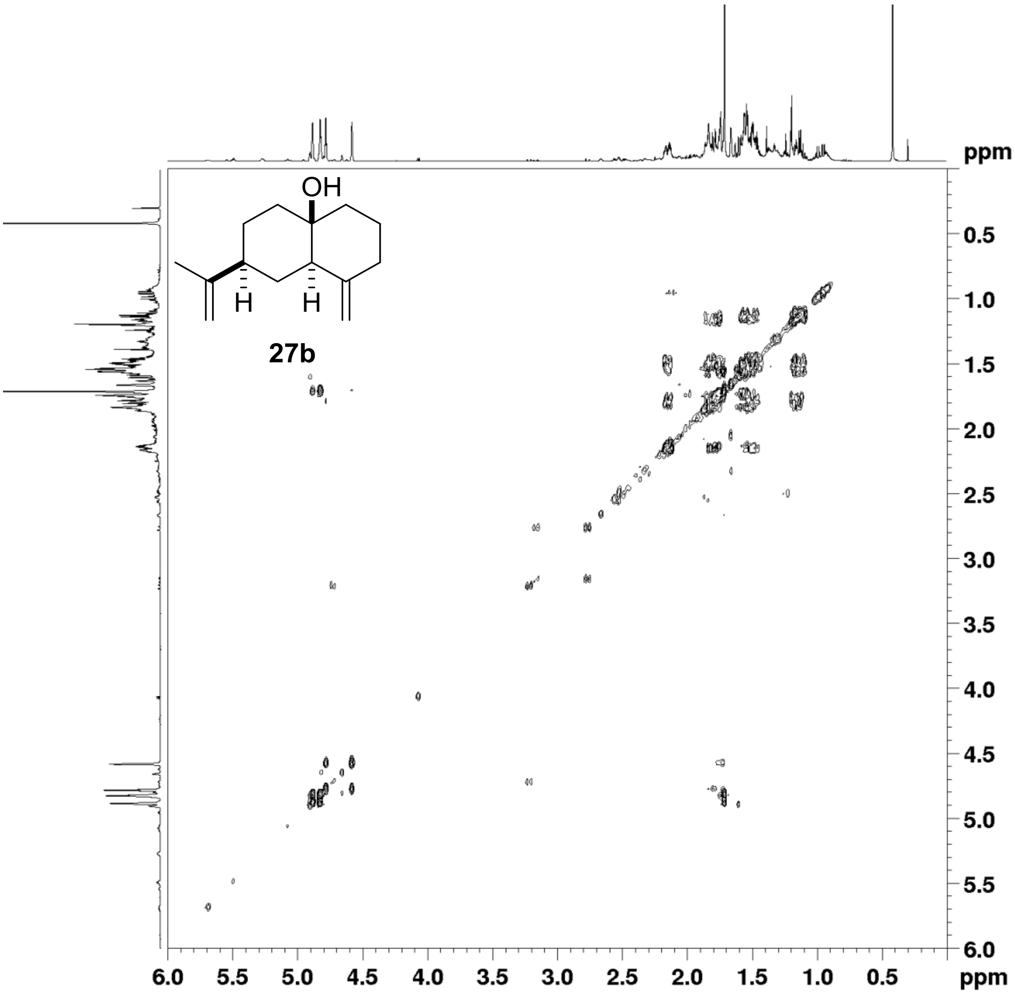

Figure S48. ^{1}H-^{1}H COSY NMR spectrum of compound **27b** in C_6D_6.

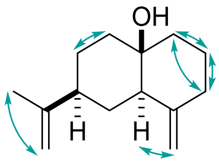

27b

Figure S49. Key ^{1}H-^{1}H COSY NMR correlations of **27b** as indicated by arrows.

Table S19. Selected correlations between ^{13}C NMR signals and neighbouring ^{1}H NMR signals as collected from the ^{1}H-^{13}C HMBC spectrum of compound **27b**. Note: Signals with weak intensities are given in parentheses.

δ (^{1}H)/ppm	δ (^{13}C)/ppm
4.88 + 4.82	45.5, 21.0
4.78 + 4.58	49.5, 36.8
2.15 + 1.79	149.9, 108.5, 24.0, (49.5, 39.8)
1.84 + 1.51	Hard to distinguish
1.83	Hard to distinguish

δ (^{1}H)/ppm	δ (^{13}C)/ppm
1.77 + 1.16	71.1, 49.5
1.73	29.5
1.71	150.4, 109.0, 45.5
1.56 + 1.13	36.8, 24.0
1.55	49.5, 26.8
1.48	-

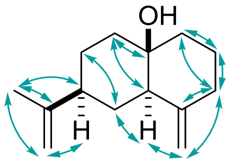

27b

Figure S50. Key ^{1}H-^{13}C HMBC NMR correlations of **27b** as indicated by arrows.

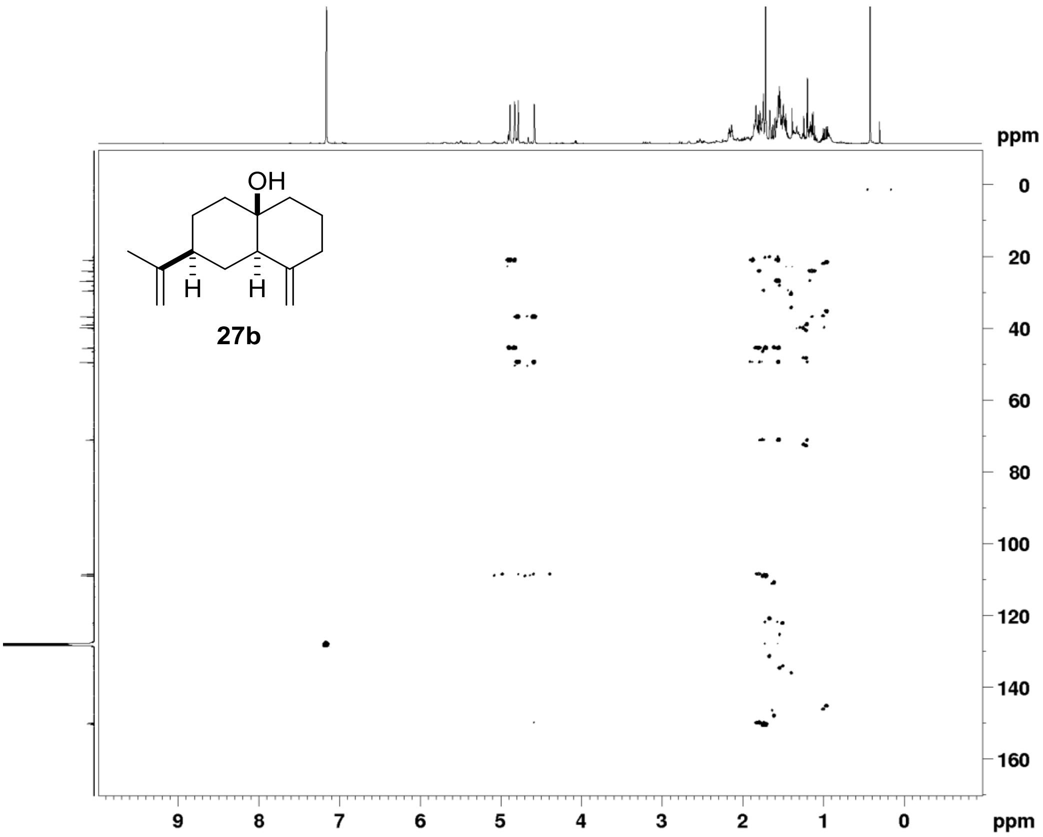

Figure S51. ^{1}H-^{13}C HMBC NMR spectrum of compound **27b** in C_6D_6.

^{1}H-^{1}H NOESY NMR shows no correlation between the CH group (1.73 ppm) and the alcohol proton (1.19 ppm), which indicates the *anti*-orientation. Comparison with a literature known compound, leads to the conclusion of *syn* orientated CH groups (1.73 ppm and 1.84 pm). [S13]

Additionally no correlation between 4.88/4.82 ppm and 1.73 ppm is observed (analogous signal reported in literature spectra of diastereomer).

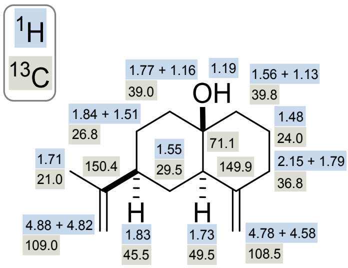

27b

Figure S52. Full assignment of chemical shifts for compound **27b**.

1.5.7 Structure elucidation of 27a

To simplify structure elucidation only the centre of each HSQC signal will be used as the chemical shift, despite different multiplicities, since overlapping signals can be differentiated more easily.

Table S20. ^{1}H NMR signals and their corresponding ^{13}C NMR signals for compound **27a** as analysed with the support of ^{1}H-^{13}C HSQC. The quaternary carbon atoms are listed at the bottom.

δ (^{1}H)/ppm	δ (^{13}C)/ppm	DEPT135/HSQC phase
4.87 + 4.83	108.8	CH_2
1.94 + 1.40	26.4	CH_2
1.82	46.0	CH/CH_3
1.72	21.2	CH/CH_3
1.67 + 1.28	43.5	CH_2
1.67 + 1.35	20.0	CH_2
1.46	27.0	CH_2
1.26	23.5	CH/CH_3
1.20 + 1.01	40.3	CH_2
1.19 + 1.09	41.6	CH_2
1.12	53.2	CH/CH_3
	150.4	C_q
0.66	71.8	C_{OH}
0.16	70.7	C_{OH}

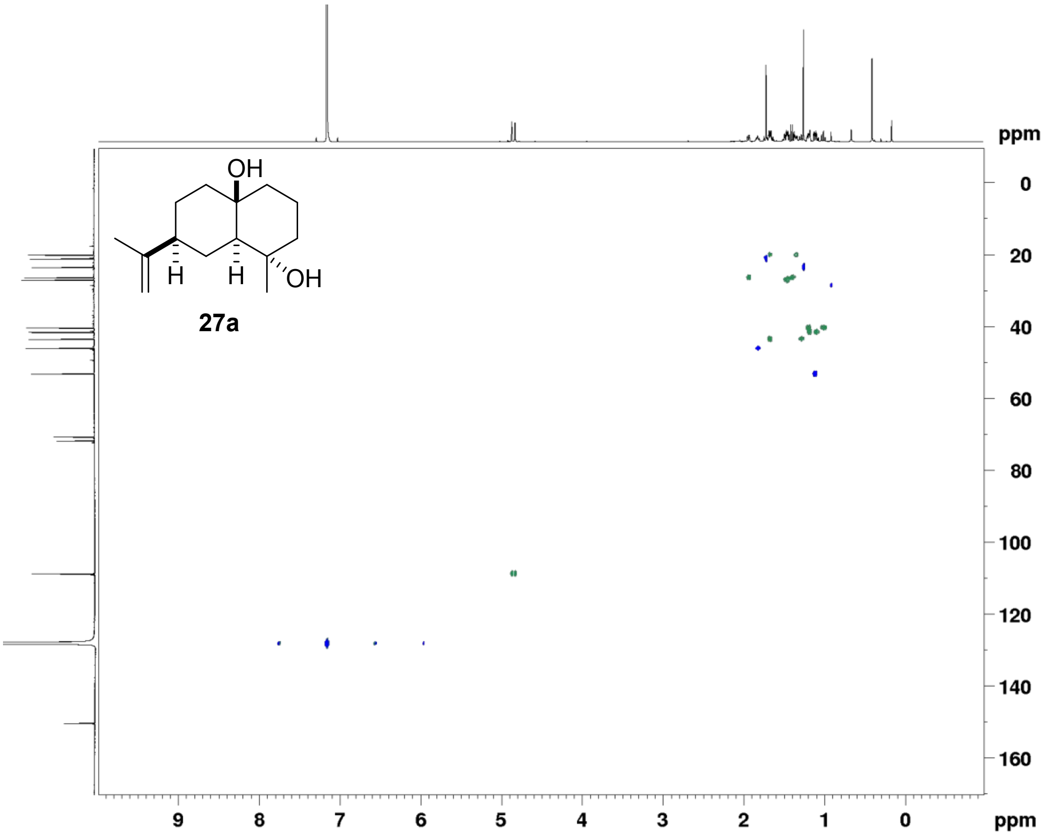

Figure S53. ^{1}H-^{13}C HSQC NMR spectrum of compound **27a** in C_6D_6 (pos. phase = blue (CH/CH_3), neg. phase = green (CH_2)).

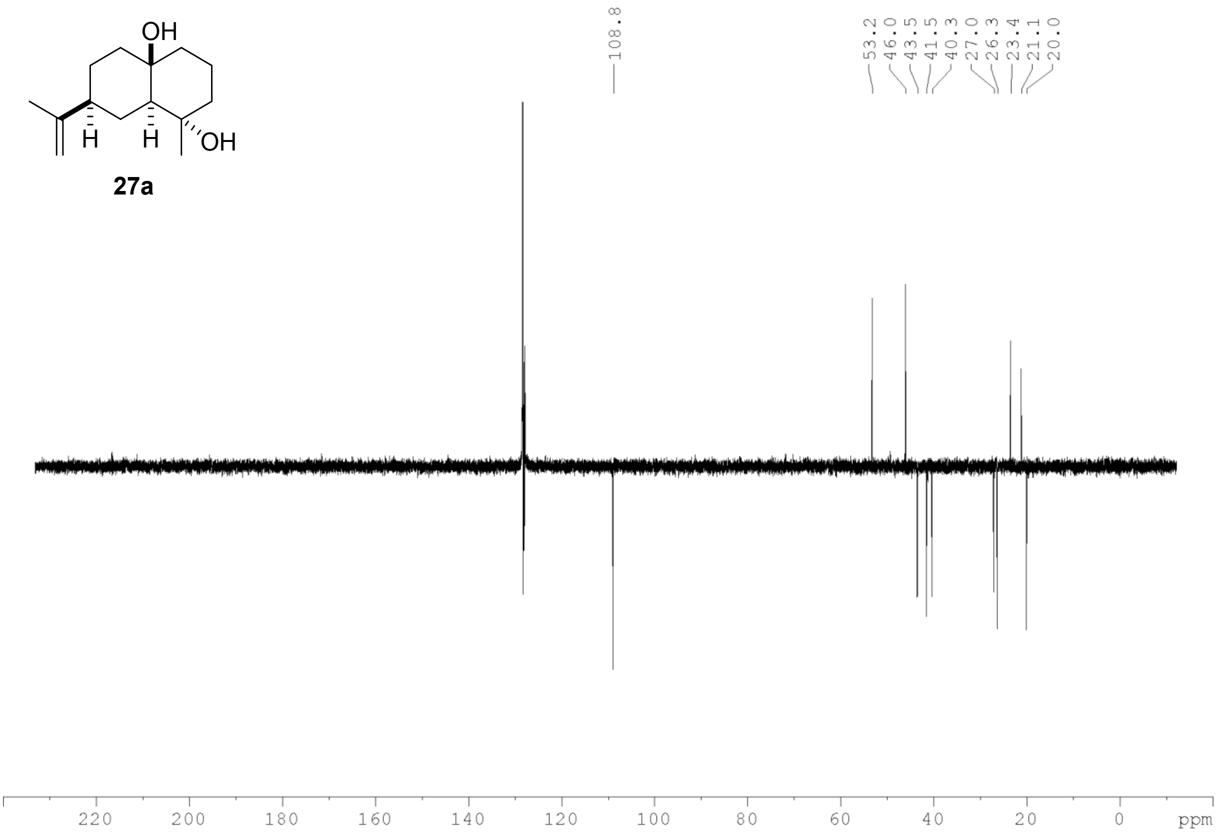

Figure S54. $^{13}C\{^{1}H\}$ DEPT135 NMR spectrum of compound **27a** in C_6D_6.

Table S21. ^{1}H NMR signals and the corresponding ^{1}H-^{1}H COSY correlations for compound **27a**. Signals with weak intensities are given in parentheses.

δ (^{1}H)/ppm	COSY correlations
4.87 + 4.83	1.72
1.94 + 1.40	1.82, 1.12
1.82	1.94, 1.46, 1.40, (1.09)
1.72	4.87 + 4.83
1.67 + 1.28	1.35
1.67 + 1.35	1.28, 1.20, 1.01
1.46	1.82, 1.19, 1.09
1.26	1.12
1.20 + 1.01	1.67, 1.35
1.19 + 1.09	1.46
1.12	1.94 + 1.40

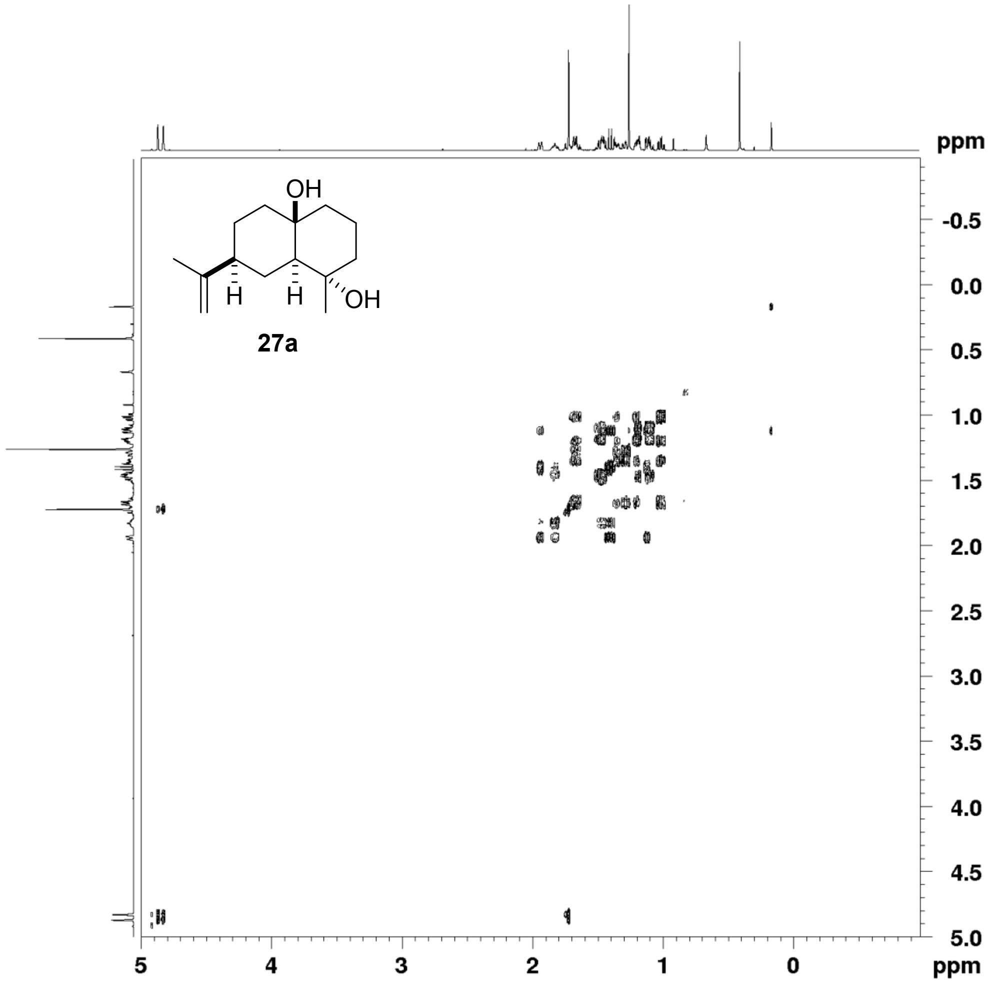

Figure S55. ^{1}H-^{1}H COSY NMR spectrum of compound **27a** in C_6D_6.

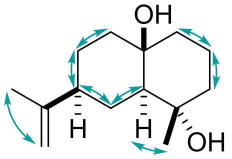

27a **Figure S56**. Key ^{1}H-^{1}H COSY NMR correlations of **27a** as indicated by arrows.

Table S22. Selected correlations between ^{13}C NMR signals and neighbouring ^{1}H NMR signals as collected from the ^{1}H-^{13}C HMBC spectrum of compound **27a**. Note: Signals with weak intensities are given in parentheses.

δ (^{1}H)/ppm	δ (^{13}C)/ppm
4.87 + 4.83	46.0, 21.2, (150.4)
1.94 + 1.40	150.4, 70.7, 53.2, 46.0, 27.0
1.82	26.4
1.72	150.4, 108.8, 46.0
1.67 (two groups)	71.8, 53.2, 40.3
1.46	46.0, 41.6, 26.4
1.26	71.8, 53.2, 43.5
1.20 + 1.01	20.0
1.19 + 1.09	27.0
1.12	71.8
0.66 (OH)	71.8, 53.2, 43.5, 23.5
0.16 (OH)	70.7, 53.2, 40.3

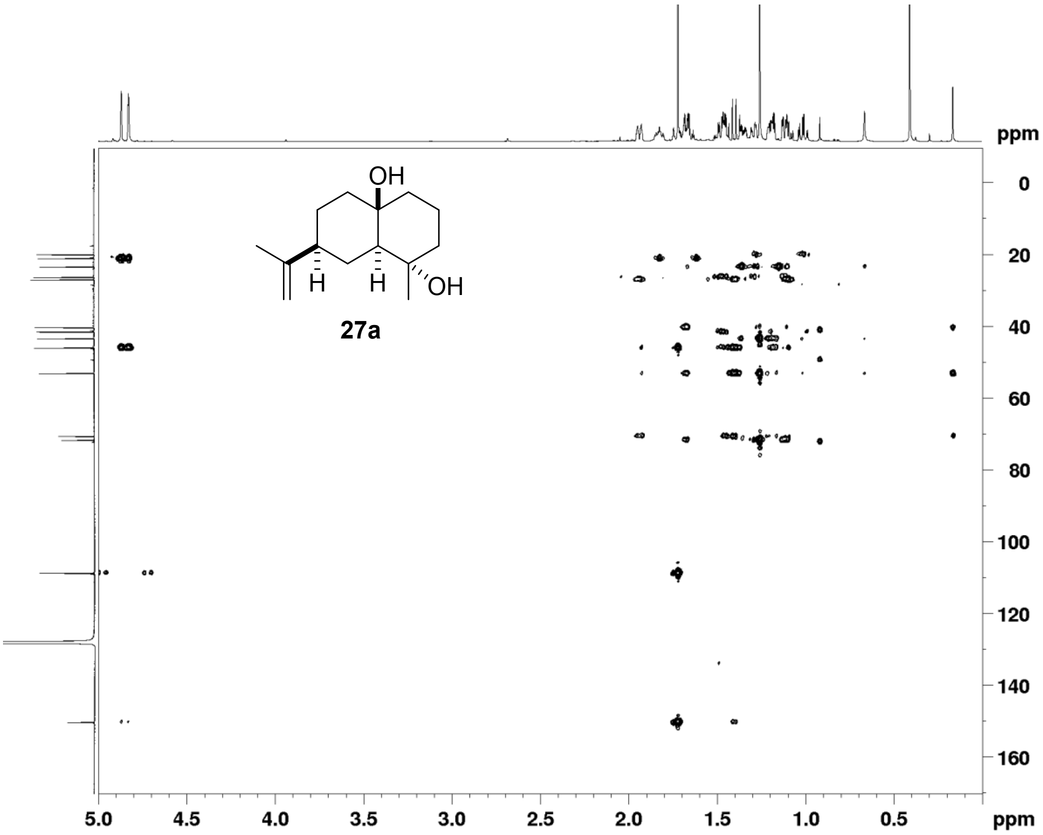

Figure S57. ^{1}H-^{13}C HMBC NMR spectrum of compound **27a** in C_6D_6.

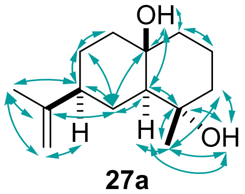

Figure S58. Key ^{1}H-^{13}C HMBC NMR correlations of **27a** as indicated by arrows.

Analysis of relative stereochemistry for compound **27a** is challenging, since the alcohol protons seem to form a proton network *via* water molecules, which leads to high number of ^{1}H-^{1}H NOESY correlation signals with both alcoholic protons.

The relative orientation between the two CH groups can be determined as *syn* by a correlation between 1.82 ppm and 1.12 ppm. This leads to four different possible pairs of diastereomeric enantiomers.

The proton at 1.40 ppm shows a strong J-coupling of 12 Hz (*pseudo*-quartet), which indicates an axial position. Since the proton at 1.40 ppm shows a ^{1}H-^{1}H NOESY correlation signal to the interchangeable proton network, there must be at least a spatial proximity to a hydroxy group. With this in mind, the pair of enantiomers in which both alcohols and the CH groups are all syn can be excluded.

If the strongest correlation signal between a backbone proton and one of the network protons arises from the spatial proximity, 0.16 ppm should be closer to 1.40 ppm and 0.16 ppm should be closer to 1.12 ppm. The shown relative stereochemistry would fit this observation. Additionally, the Me group (1.26 ppm) shows a stronger correlation signal to 1.40 ppm than to 1.94 ppm, which indicates that they are in a 1,3-diaxial relation. Also, 1.26 ppm shows a correlation to 1.67 ppm (geminal to 1.35 ppm), which strengthens the analysis towards this specific relative stereochemistry.

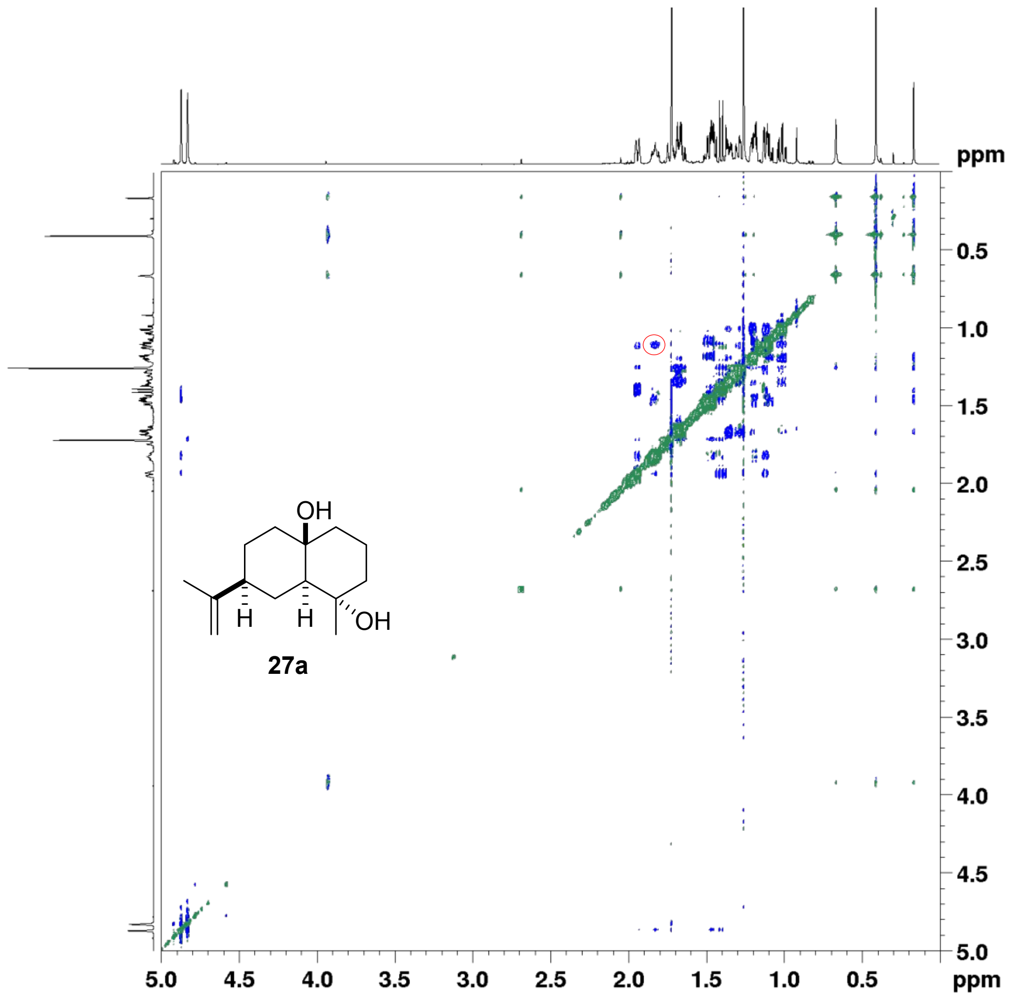

Figure S59. ^{1}H-^{1}H NOESY NMR spectrum of compound **27a** in C_6D_6. Key correlation for 1.82 ppm marked with red circles.

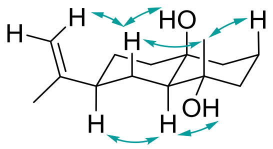

27a

Figure S60. Key ^{1}H-^{1}H NOESY correlations in compound **27a**.

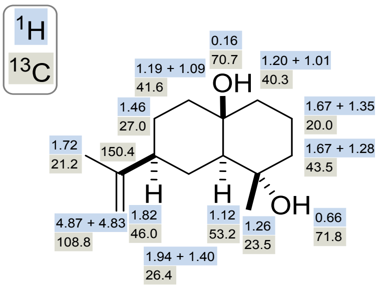

27a

Figure S61. Full assignment of chemical shifts for compound **27a**.

1.5.8 Structure elucidation of 29

To simplify structure elucidation only the centre of each HSQC signal will be used as the chemical shift, despite different multiplicities, since overlapping signals can be differentiated more easily.

Table S23. ^{1}H NMR signals and their corresponding ^{13}C NMR signals for compound **29** as analysed with the support of ^{1}H-^{13}C HSQC. The quaternary carbon atoms are listed at the bottom.

δ (^{1}H)/ppm	δ (^{13}C)/ppm	DEPT135/HSQC phase
5.90	132.7	CH/CH_3
5.25	136.9	CH/CH_3
4.85 + 4.82	113.5	CH_2
2.55 + 2.00	30.3	CH_2
2.28 + 1.20	24.6	CH_2
2.28 + 1.87	42.0	CH_2
2.10 + 1.57	31.8	CH_2
1.84	42.4	CH_2
1.45	52.9	CH/CH_3
1.32	32.0	CH/CH_3
0.80	21.0	CH/CH_3
0.78	20.9	CH/CH_3
	210.6	C_q
	147.0	C_q

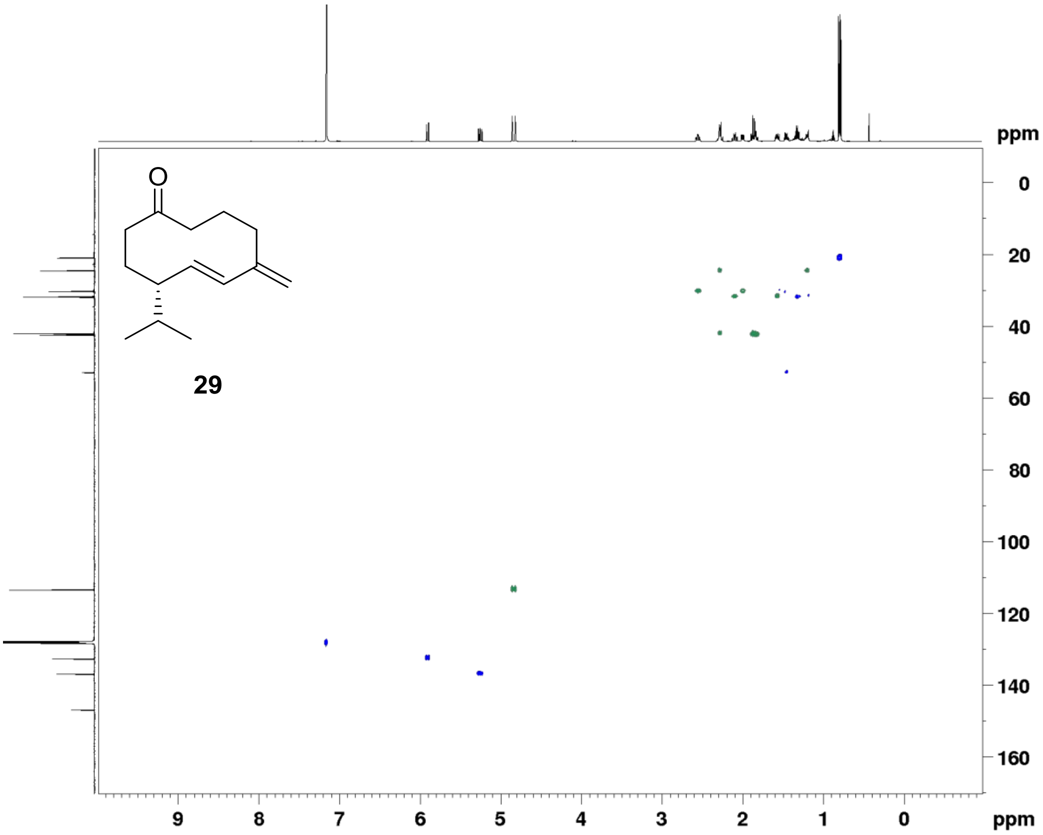

Figure S62. ^{1}H-^{13}C HSQC NMR spectrum of compound **29** in C_6D_6 (pos. phase = blue (CH/CH_3), neg. phase = green (CH_2)).

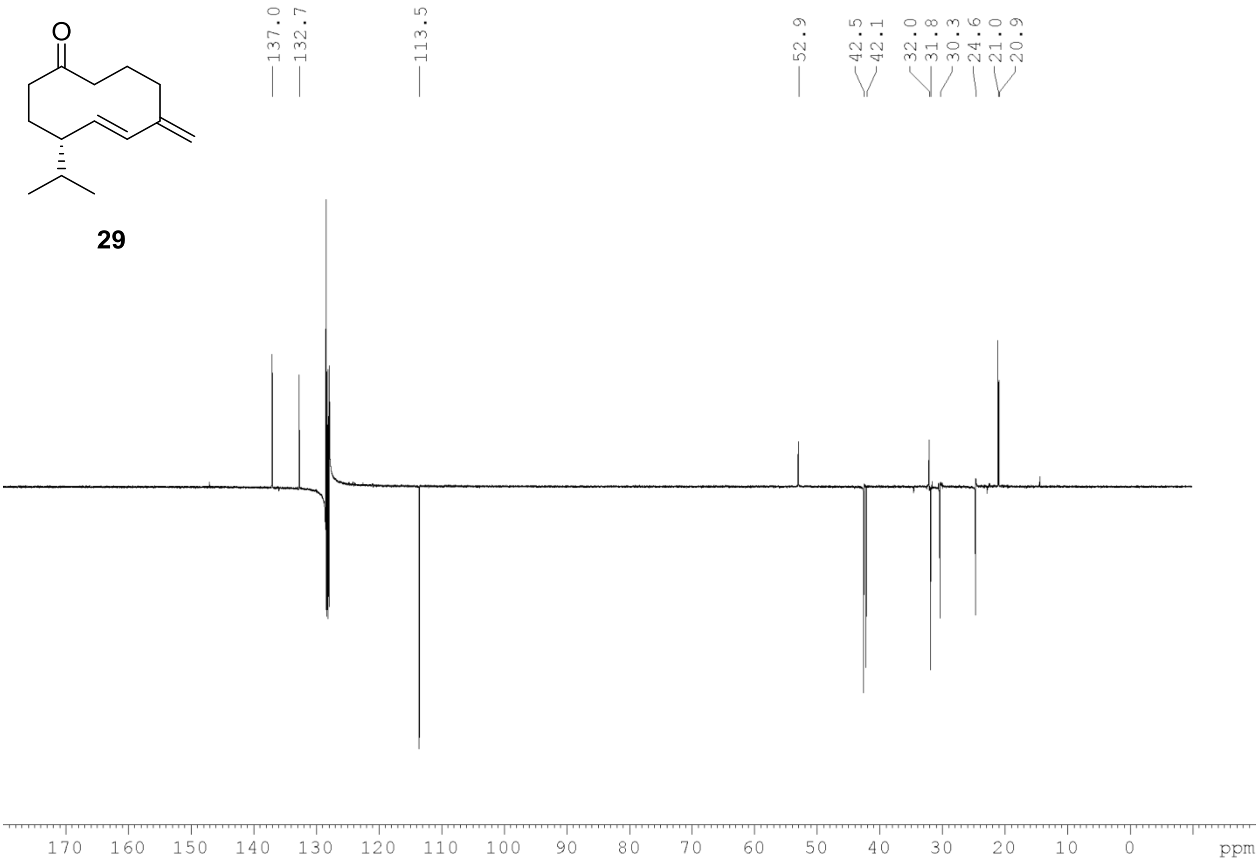

Figure S63. $^{13}C\{^{1}H\}$ DEPT135 NMR spectrum of compound **29** in C_6D_6.

Table S24. ^{1}H NMR signals and the corresponding ^{1}H-^{1}H COSY correlations for compound **29**. Signals with weak intensities are given in parentheses.

δ (^{1}H)/ppm	COSY correlations
5.90	5.25, 4.85 + 4.82, (2.55 + 2.00, 1.45)
5.25	5.90, 1.45, (4.85 + 4.82)
4.85 + 4.82	5.90, (2.55 + 2.00)
2.55 + 2.00	2.28 + 1.20
2.28 + 1.20	2.55, 2.28, 2.00, 1.87
2.28 + 1.87	
2.10 + 1.57	1.84, 1.45
1.84	2.10 + 1.57
1.45	5.25, 2.10 + 1.57, 1.32
1.32	1.45, 0.80, 0.78
0.80	1.32
0.78	1.32

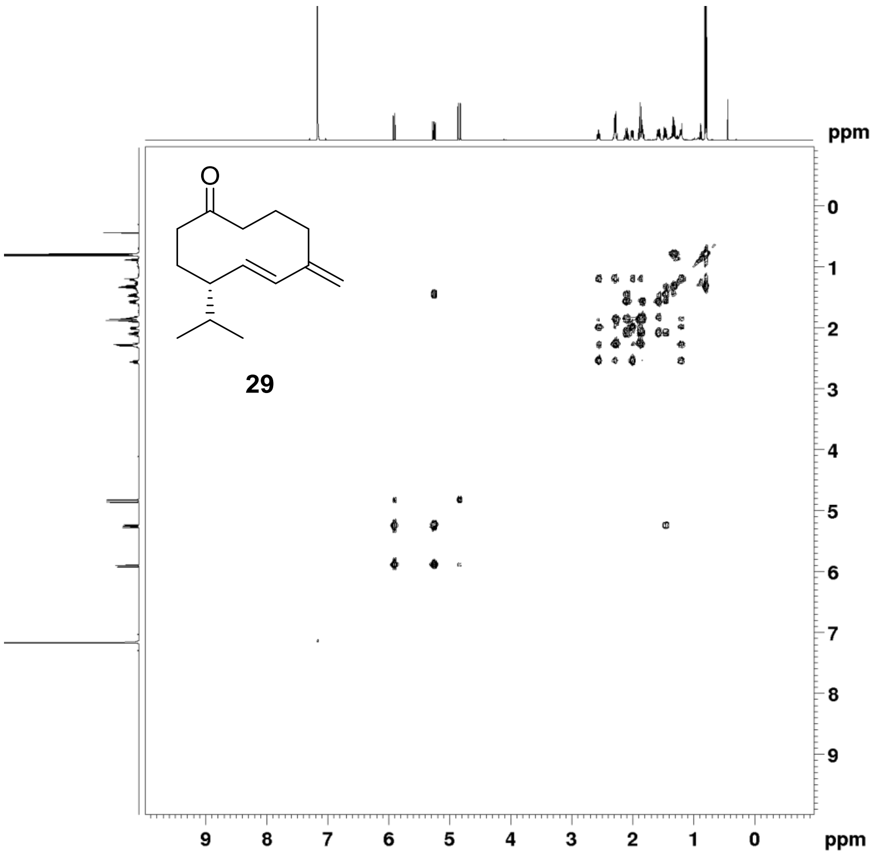

Figure S64. ^{1}H-^{1}H COSY NMR spectrum of compound **29** in C_6D_6.

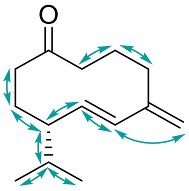

29

Figure S65. Key ^{1}H-^{1}H COSY NMR correlations of **29** as indicated by green arrows.

Table S25. Selected correlations between ^{13}C NMR signals and neighbouring ^{1}H NMR signals as collected from the ^{1}H-^{13}C HMBC spectrum of compound **29**. Note: Signals with weak intensities are given in parentheses.

δ (^{1}H)/ppm	δ (^{13}C)/ppm
5.90	147.0, 113.5, 52.9, 31.8 or 32.0, 30.3
5.25	147.0, 113.5, 52.9, 31.8 or 32.0
4.85 + 4.82	147.0, 132.7, 30.2, 24.6
2.55 + 2.00	147.0, 132.7, 113.5, 42.4, 24.6
2.28 + 1.20	210.6, 147.0, 30.3
2.28 + 1.87	30.3, 24.6
2.10 + 1.57	210.6, 136.9, 52.9, 42.4, 31.8 or 32.0
1.84	Hard to distinguish
1.45	136.9, 132.7, 31.9 or 32.0, 20.9/21.0,
1.32	136.9, 52.9, 31.9 or 32.0, 20.9/21.0
0.80	52.9, 31.9 or 32.0, 20.9
0.78	52.9, 31.9 or 32.0, 21.0

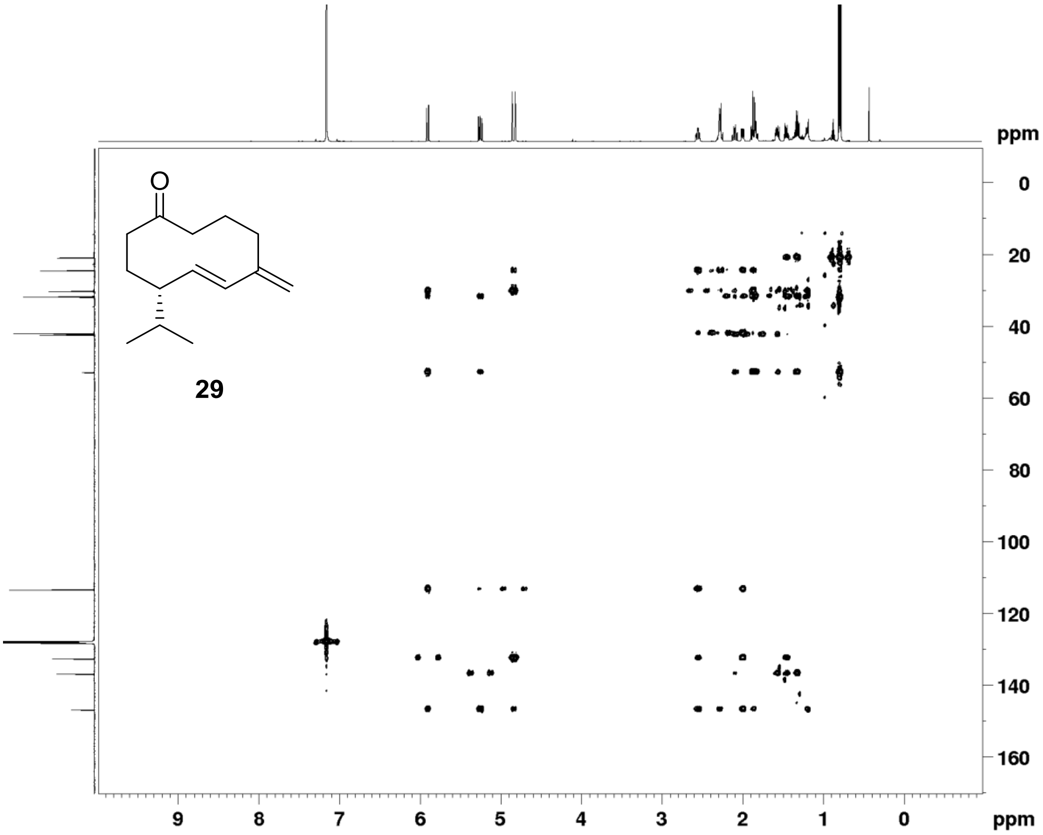

Figure S66. ^{1}H-^{13}C HMBC NMR spectrum of compound **29** in C_6D_6.

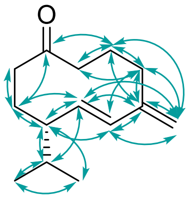

29

Figure S67. Key ^{1}H-^{13}C HMBC NMR correlations of **29** as indicated by arrows.

Since ^{1}H-^{1}H NOESY NMR shows no correlation between 5.90 ppm and 5.25 ppm, but only *J* coupling, the double geometry is assigned as *trans*. The NMR data fits to the reported data. [S14] The isopropyl group is stereochemical derived/proposed from Cop4's natural cyclisation mechanism.

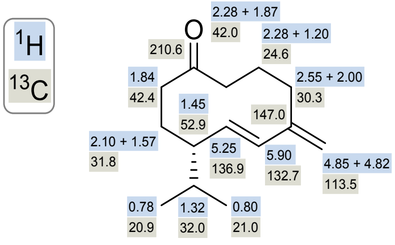

29

Figure S68. Full assignment of chemical shifts for compound **29**.

1.5.9 Structure elucidation of 30

To simplify structure elucidation only the centre of each HSQC signal will be used as the chemical shift, despite different multiplicities, since overlapping signals can be differentiated more easily.

Table S26. ^{1}H NMR signals and their corresponding ^{13}C NMR signals for compound **30** as analysed with the support of ^{1}H-^{13}C HSQC. The quaternary carbon atoms are listed at the bottom.

δ (^{1}H)/ppm	δ (^{13}C)/ppm	DEPT135/HSQC phase
5.75	142.7	CH/CH_3
5.27	124.8	CH/CH_3
5.24 + 5.00	112.5	CH_2
4.51	94.3	CH/CH_3
2.38	26.4	CH_2
2.23	35.3	CH_2
1.86	18.7	CH_2
1.66	25.9	CH/CH_3
1.56	17.8	CH/CH_3
1.48	32.1	CH_2
1.26	26.4	CH/CH_3
	152.7	
	131.4	
	75.9	

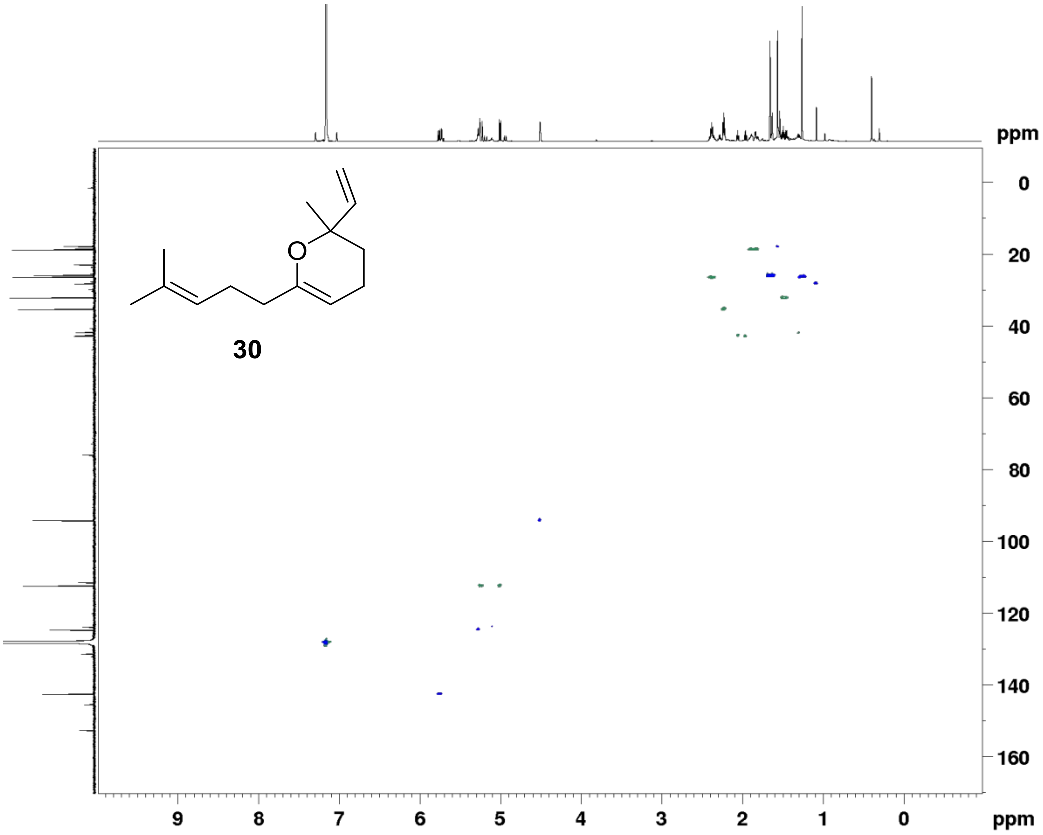

Figure S69. ^{1}H-^{13}C HSQC NMR spectrum of compound **30** in C_6D_6 (pos. phase = blue (CH/CH_3), neg. phase = green (CH_2)).

Table S27. ^{1}H NMR signals and the corresponding ^{1}H-^{1}H COSY correlations for compound **30**. Signals with weak intensities are given in parentheses.

δ (^{1}H)/ppm	COSY correlations
5.75	5.24 + 5.00
5.27	2.38, 1.65, 1.56
5.24 + 5.00	5.75
4.51	1.86
2.38	5.27, 2.23, 1.65
2.23	2.38
1.86	4.51, 1.48
1.66	5.27, 2.38
1.56	5.27, 1.65
1.48	1.86
1.26	

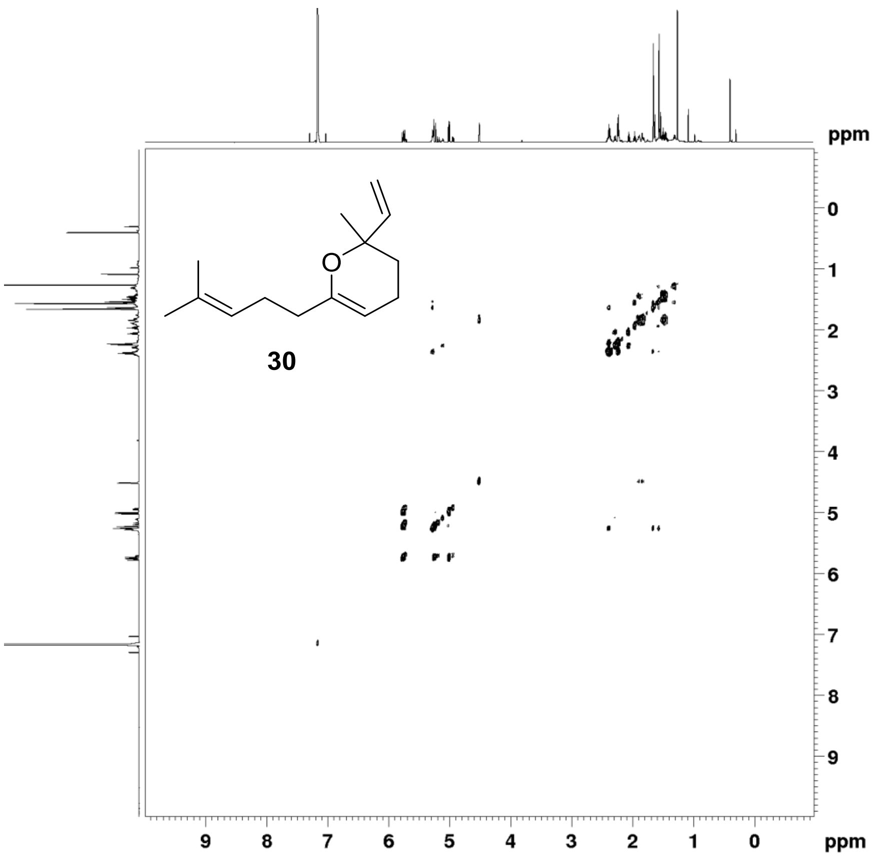

Figure S70. ^{1}H-^{1}H COSY NMR spectrum of compound **30** in C_6D_6.

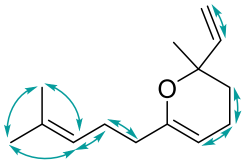

30

Figure S71. Key ^{1}H-^{1}H COSY NMR correlations of **30** as indicated by arrows.

Table S28. Selected correlations between ^{13}C NMR signals and neighbouring ^{1}H NMR signals as collected from the ^{1}H-^{13}C HMBC spectrum of compound **30**. Note: Signals with weak intensities are given in parentheses.

δ (^{1}H)/ppm	δ (^{13}C)/ppm
5.75	75.9
5.27	25.9, 17.8
5.24 + 5.00	142.7, 75.9
4.51	152.7, 32.1
2.38	152.7, 131.4, 124.8, 35.3
2.23	152.7, 124.8, 94.3, 26.4
1.86	152.7, 94.3, 32.1
1.66	131.4, 124.8, 17.8

δ (^{1}H)/ppm	δ (^{13}C)/ppm
1.56	131.4, 124.8, 25.9
1.48	142.7, 94.3, 75.9, 26.4, 18.7
1.26	142.7, 75.9, 32.1

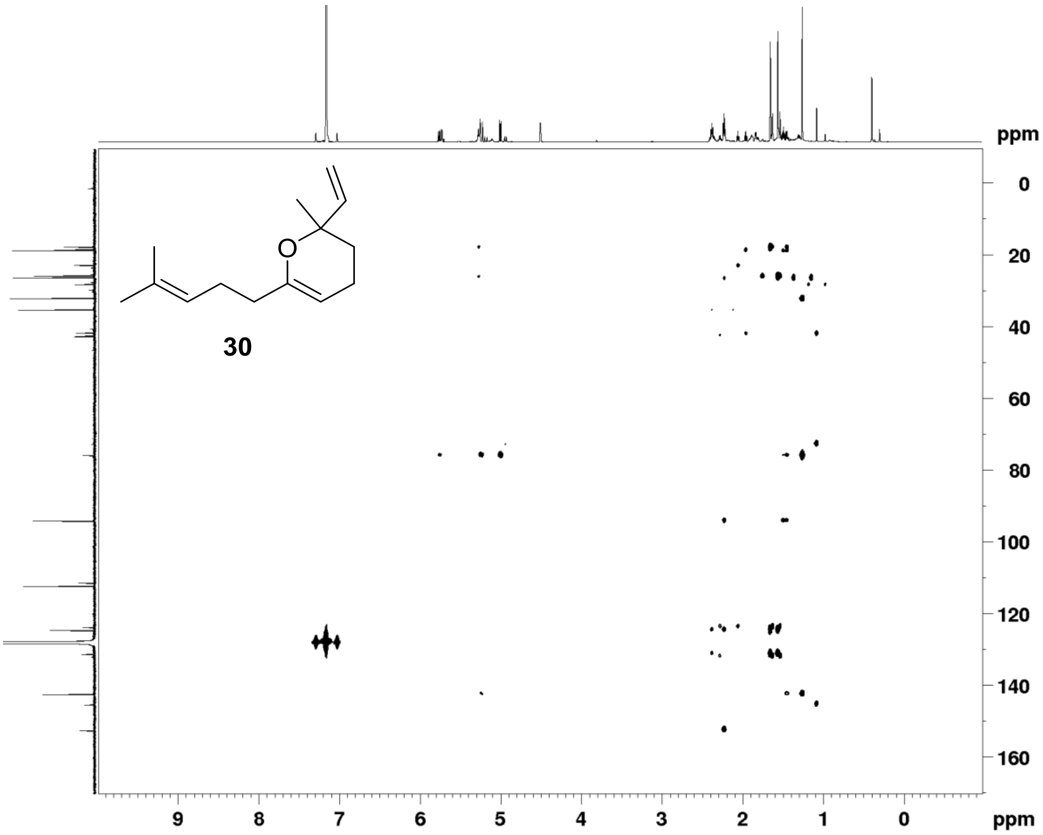

Figure S71. ^{1}H-^{13}C HMBC NMR spectrum of compound **30** in C_6D_6.

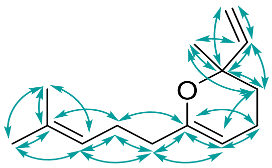

30

Figure S72. Key ^{1}H-^{13}C HMBC NMR correlations of **30** as indicated by arrows.

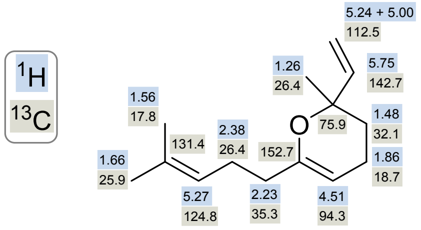

30

Figure S73. Full assignment of chemical shifts for compound **30**.

1.5.10 Structure elucidation of 33

To simplify structure elucidation only the centre of each HSQC signal will be used as the chemical shift, despite different multiplicities, since overlapping signals can be differentiated more easily.

Table S29. ^{1}H NMR signals and their corresponding ^{13}C NMR signals for compound **33** as analysed with the support of ^{1}H-^{13}C HSQC. The quaternary carbon atoms are listed at the bottom.

δ (^{1}H)/ppm	δ (^{13}C)/ppm	DEPT135/HSQC phase
4.75	111.2	CH_2
3.00 + 2.59	38.4	CH_2
2.98	30.4	CH/CH_3
2.29 + 2.10	37.2	CH_2
2.16 + 2.00	48.1	CH_2
2.15	52.7	CH_2
1.52	26.2	CH_2
1.43 + 1.27	37.2	CH_2
1.11	30.1	CH/CH_3
1.06	30.6	CH/CH_3
0.95	19.8	CH/CH_3
	150.4	C_q
	137.0	C_q
	132.3	C_q
	36.0	C_q

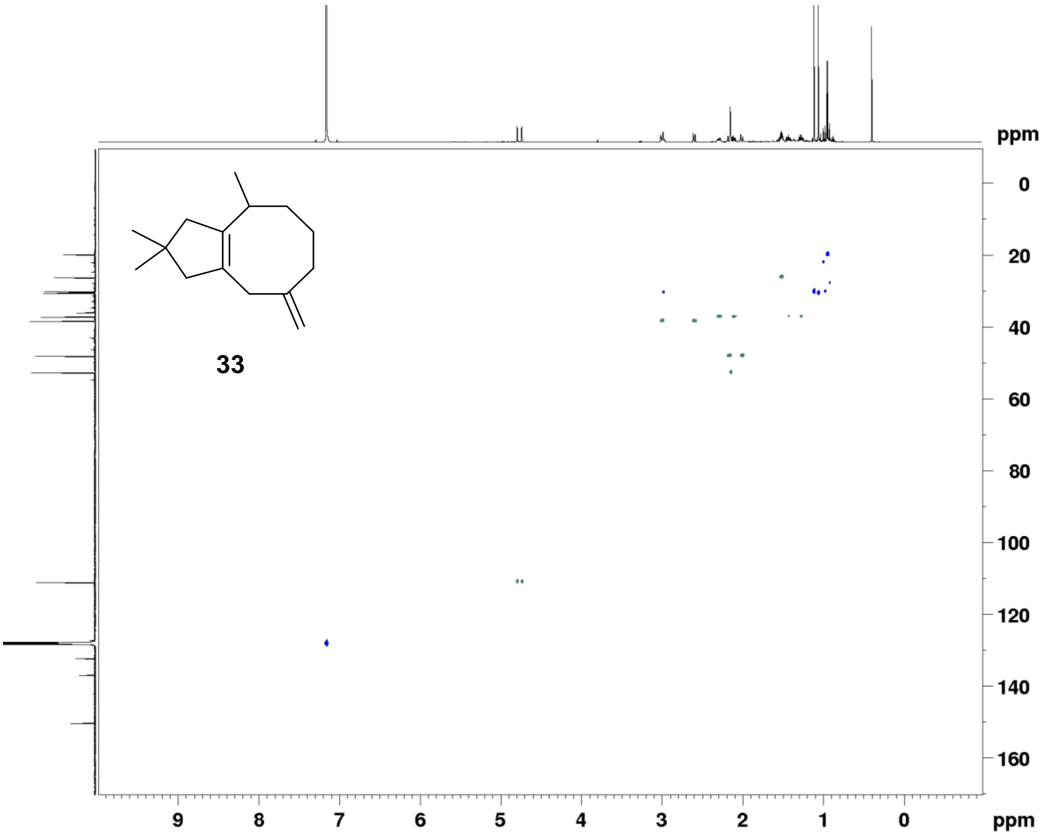

Figure S74. ^{1}H-^{13}C HSQC NMR spectrum of compound **33** in C_6D_6 (pos. phase = blue (CH/CH_3), neg. phase = green (CH_2)).

Table S30. ^{1}H NMR signals and the corresponding ^{1}H-^{1}H COSY correlations for compound **33**. Signals with weak intensities are given in parentheses.

δ (^{1}H)/ppm	COSY correlations
4.75	(3.00, 2.59, 2.29)
3.00 + 2.59	2.16 + 2.00
2.98	1.43 + 1.27, 0.95
2.29 + 2.10	1.52
2.16 + 2.00	3.00 + 2.59
2.15	
1.52	2.29 + 2.10, 1.43 + 1.27
1.43 + 1.27	2.98, 1.52
1.11	1.06
1.06	1.11
0.95	2.98

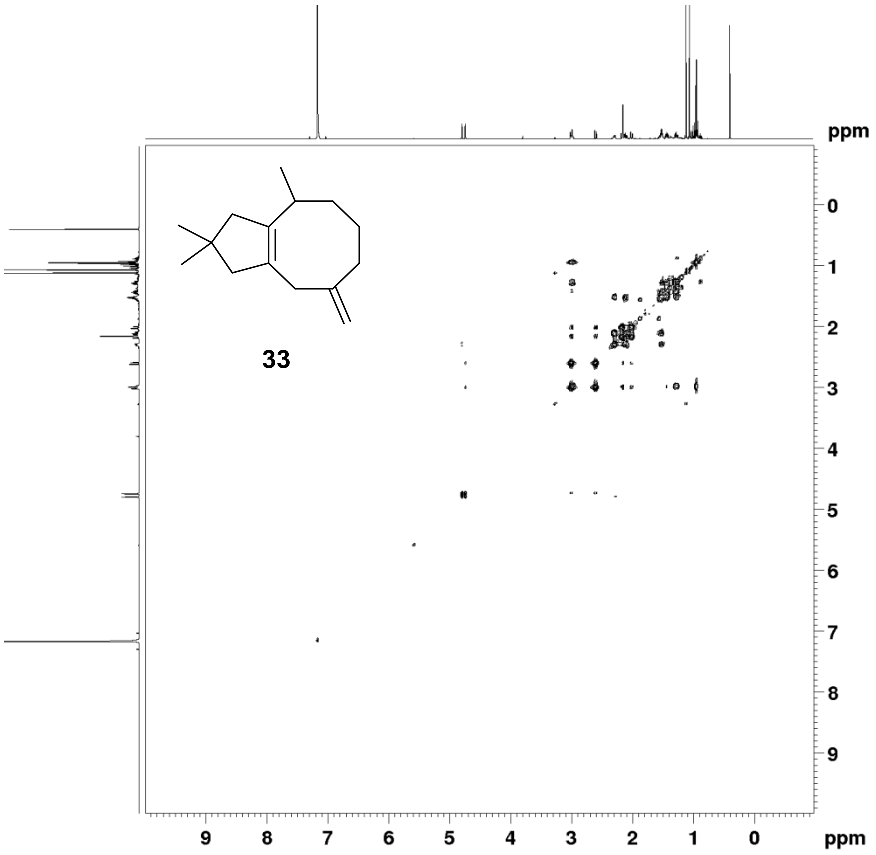

Figure S75. ^{1}H-^{1}H COSY NMR spectrum of compound **33** in C_6D_6.

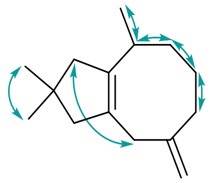

33

Figure S76. Key ^{1}H-^{1}H COSY NMR correlations of **33** as indicated by arrows.

Table S31: Selected correlations between ^{13}C NMR signals and neighbouring ^{1}H NMR signals as collected from the ^{1}H-^{13}C HMBC spectrum of compound **33**. Note: Signals with weak intensities are given in parentheses.

δ (^{1}H)/ppm	δ (^{13}C)/ppm
4.75	150.4, 38.4, 37.2
3.00 + 2.59	150.4, 137.0, 132.3, 111.2, 52.7, 37.2
2.98	48.1, 19.8
2.29 + 2.10	150.4, 111.2, 38.4, 37.2, 26.2
2.16 + 2.00	137.0, 132.3, 30.4
2.15	137.0, 132.3, 48.1, 36.0, 30.4
1.52	150.4, 37.2, 30.4
1.43 + 1.27	137.0, 37.2, 30.4, 26.2, 19.8

δ (^{1}H)/ppm	δ (^{13}C)/ppm
1.11	52.7, 48.1, 36.0, 30.6
1.06	52.7, 48.1, 36.0, 30.1
0.95	137.0, 37.2, 30.4

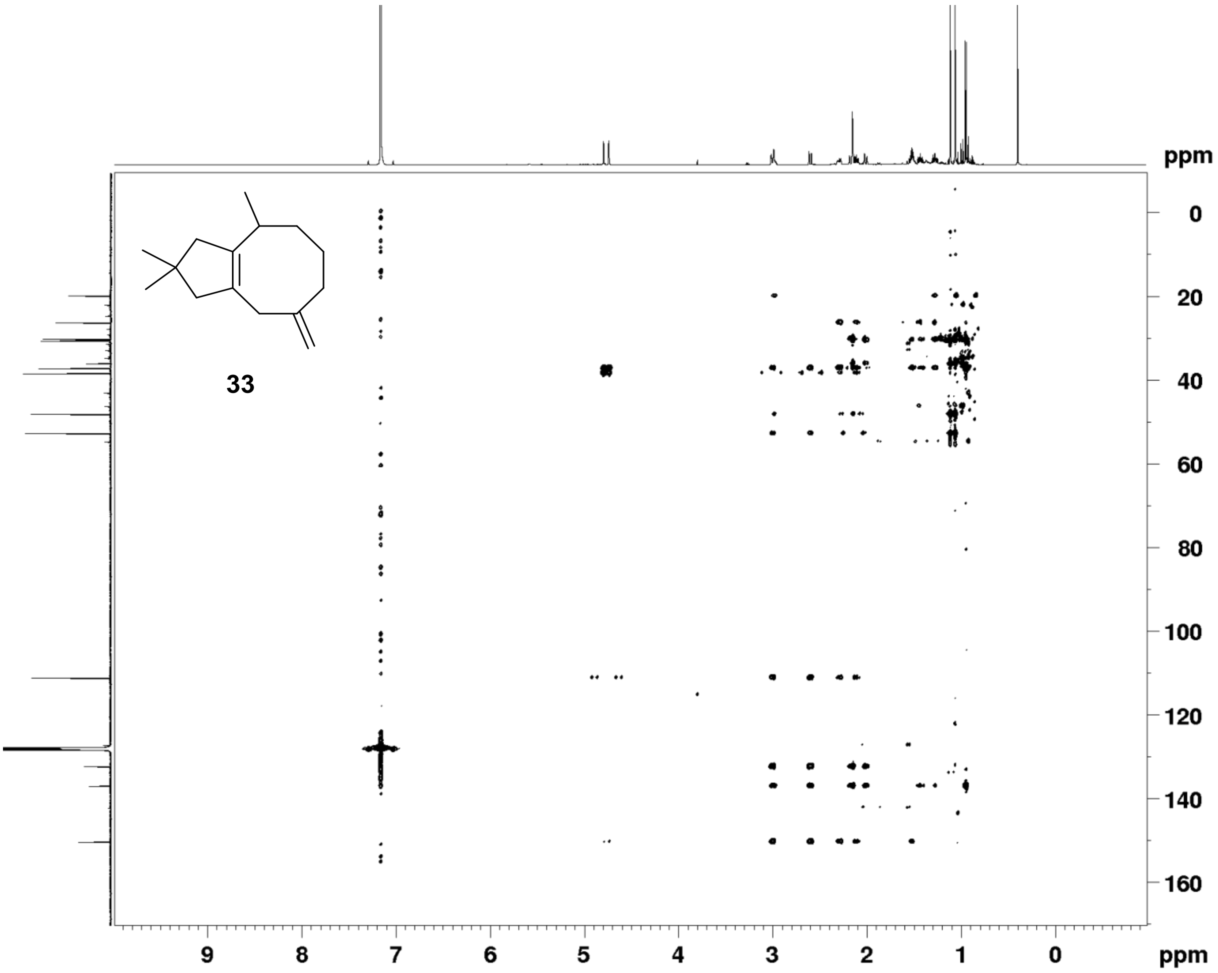

Figure S77. ^{1}H-^{13}C HMBC NMR spectrum of compound **33** in C_6D_6.

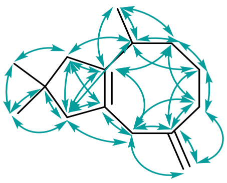

33

Figure S78. Key ^{1}H-^{13}C HMBC NMR correlations of **33** as indicated by arrows.

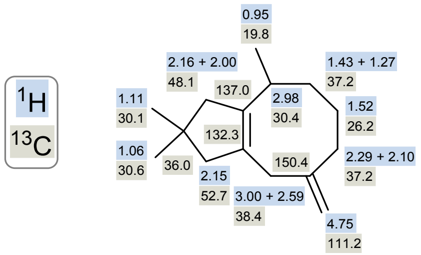

33

Figure S79. Full assignment of chemical shifts for compound **33**.

1.5.11 Structure elucidation of 34

To simplify structure elucidation only the centre of each HSQC signal will be used as the chemical shift, despite different multiplicities, since overlapping signals can be differentiated more easily.

Table S32. ^{1}H NMR signals and their corresponding ^{13}C NMR signals for compound **34** as analysed with the support of ^{1}H-^{13}C HSQC. The quaternary carbon atoms are listed at the bottom.

δ (^{1}H)/ppm	δ (^{13}C)/ppm	DEPT135/HSQC phase
1.93 + 1.63	42.4	CH_2
1.71 + 1.47	22.2	CH_2
1.70	48.9	CH/CH_3
1.64 + 1.22	41.8	CH_2
1.59 + 0.93	31.0	CH2
1.57 + 1.20	54.1	CH_2
1.51	50.4	CH/CH_3
1.43 + 1.26	35.6	CH_2
1.33 + 1.24	24.4	CH_2
0.99	30.5	CH/CH_3
0.95	20.3	CH/CH_3
0.73	30.3	CH/CH_3
	73.5	C_q
	35.9	C_q
	34.0	C_q

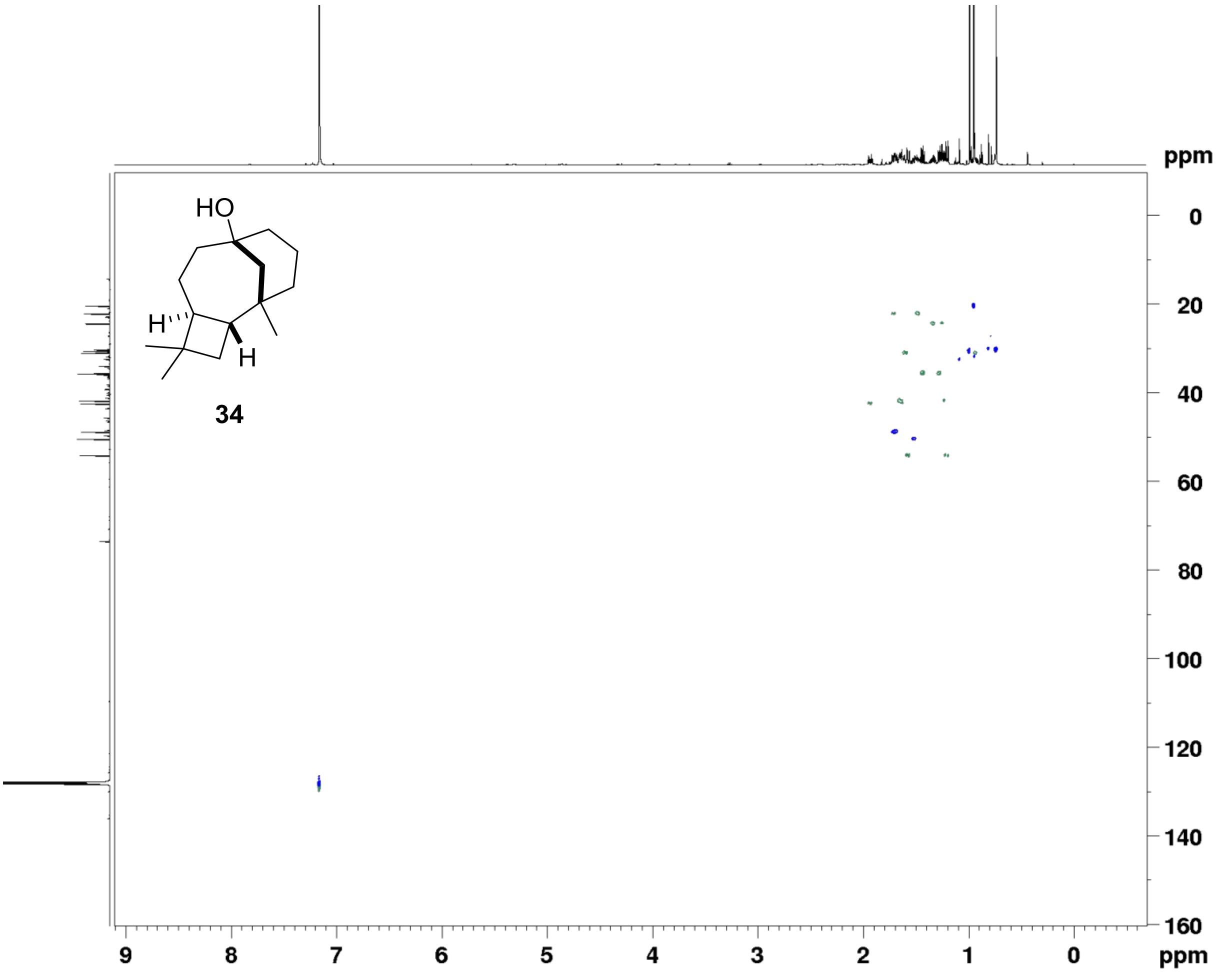

Figure S80. ^{1}H-^{13}C HSQC NMR spectrum of compound **34** in C_6D_6 (pos. phase = blue (CH/CH_3), neg. phase = green (CH_2)).

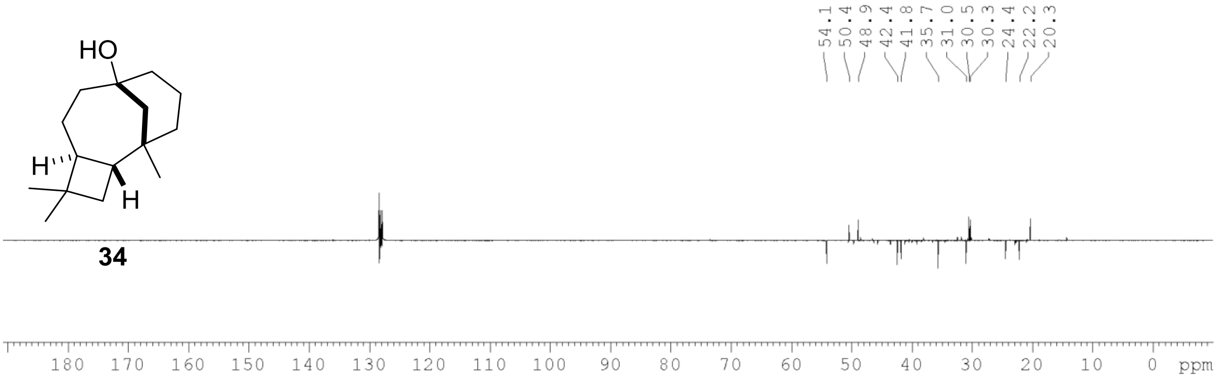

Figure S81. $^{13}C\{^{1}H\}$ DEPT135 NMR spectrum of compound **34** in C_6D_6.

Since all ^{1}H-NMR signals are between 1.93 ppm and 0.73 ppm, the ^{1}H-^{1}H COSY suffers from intense superposition. Therefore, ^{1}H-^{13}C H2BC data was collected additionally.

Table S33. ^{1}H NMR signals and the corresponding ^{1}H-^{1}H COSY and ^{1}H-^{13}C H2BC correlations for compound **34**. Signals with weak intensities are given in parentheses. Superposing signals which cannot be distinguished, are marked with "x".

δ (^{1}H)/ppm	COSY correlations	H2BC correlations
1.93 + 1.63	1.33 + 1.24	
1.71 + 1.47	1.2x, 0.93	
1.70	Hard to distinguish	50.4, 24.4
1.64 + 1.22		22.2
1.59 + 0.93		22.2
1.57 + 1.20	-	(73.5)
1.51	1.70, 1.26	48.9, 35.6
1.43 + 1.26	1.51	
1.33 + 1.24	1.6x	48.9, 42.4
0.99	-	35.6 or 35.9, 20.3,
0.95	-	35.6 or 35.9, 30.5
0.73	-	

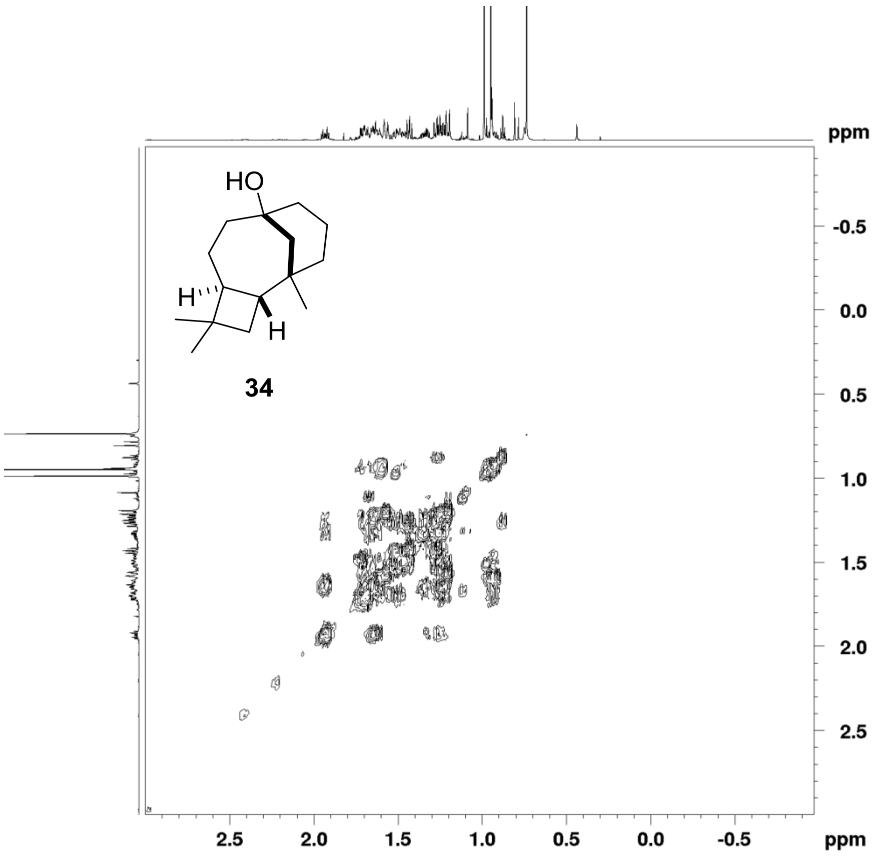

Figure S82. ^{1}H-^{1}H COSY NMR spectrum of compound **34** in C_6D_6.

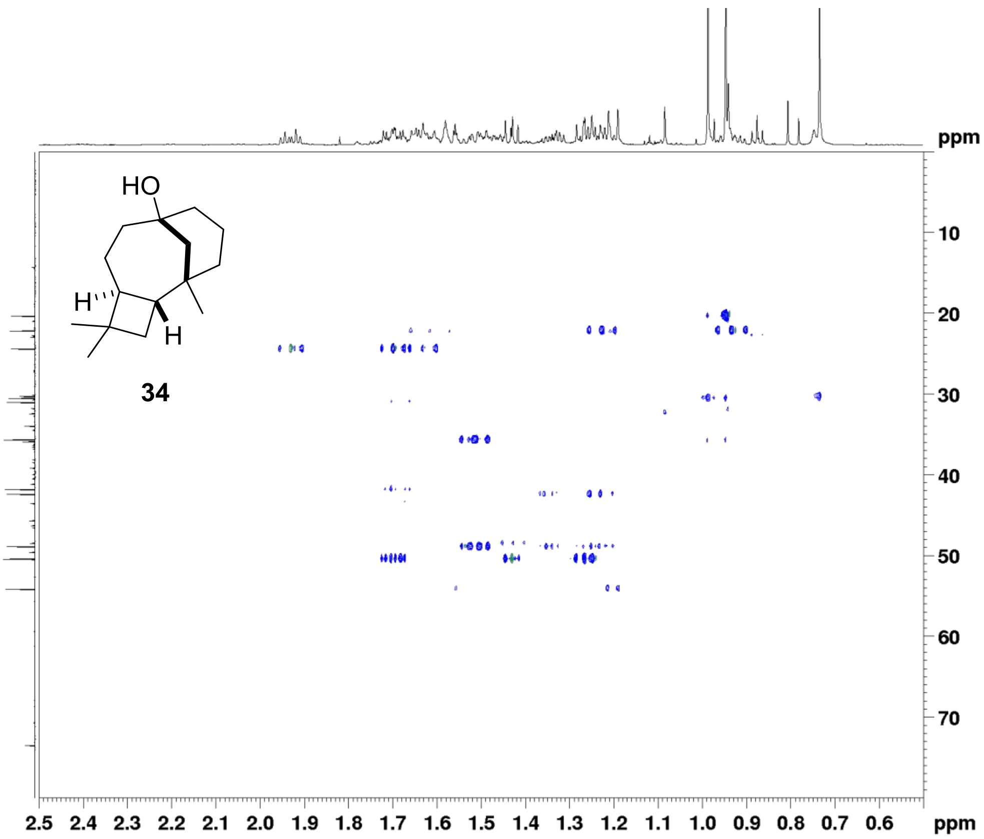

Figure S83. ^{1}H-^{13}C H2BC NMR spectrum of compound **34** in C_6D_6.

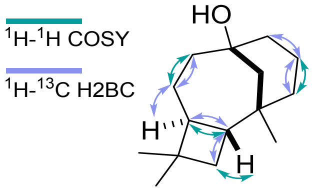

Figure S84. Key ^{1}H-^{1}H COSY (green) and ^{1}H-^{13}C H2BC (violet) NMR correlations of **34** as indicated by arrows.

Table S34. Selected correlations between ^{13}C NMR signals and neighbouring ^{1}H NMR signals as collected from the ^{1}H-^{13}C HMBC spectrum of compound **34**. Note: Signals with weak intensities are given in parentheses.

δ (^{1}H)/ppm	δ (^{13}C)/ppm
1.93 + 1.63	73.5, 48.9, 41.8, 24.4
1.71 + 1.47 or 1.70	50.4, 42.4, 35.9, 33.9, 30.6, 20.3
1.64 + 1.22	22.2
1.59 + 0.93	50.4
1.57 + 1.20	73.5, 50.4, 42.4, 41.8, 34.0, 31.0, 30.3
1.51	54.1, 48.9, 35.6, 31.0, 24.4
1.43 + 1.26	50.4, 48.9, 35.9, 34.0, 30.5, 24.4, 20.3

δ (^{1}H)/ppm	δ (^{13}C)/ppm
1.33 + 1.24	73.5, 50.4, 48.9, 42.4, 35.9
0.99	48.9, 35.6 or 35.9, 20.3
0.95	48.9, 35.6 or 35.9, 30.5
0.73	73.5, 54.1, 50.4, 34.0, 31.0

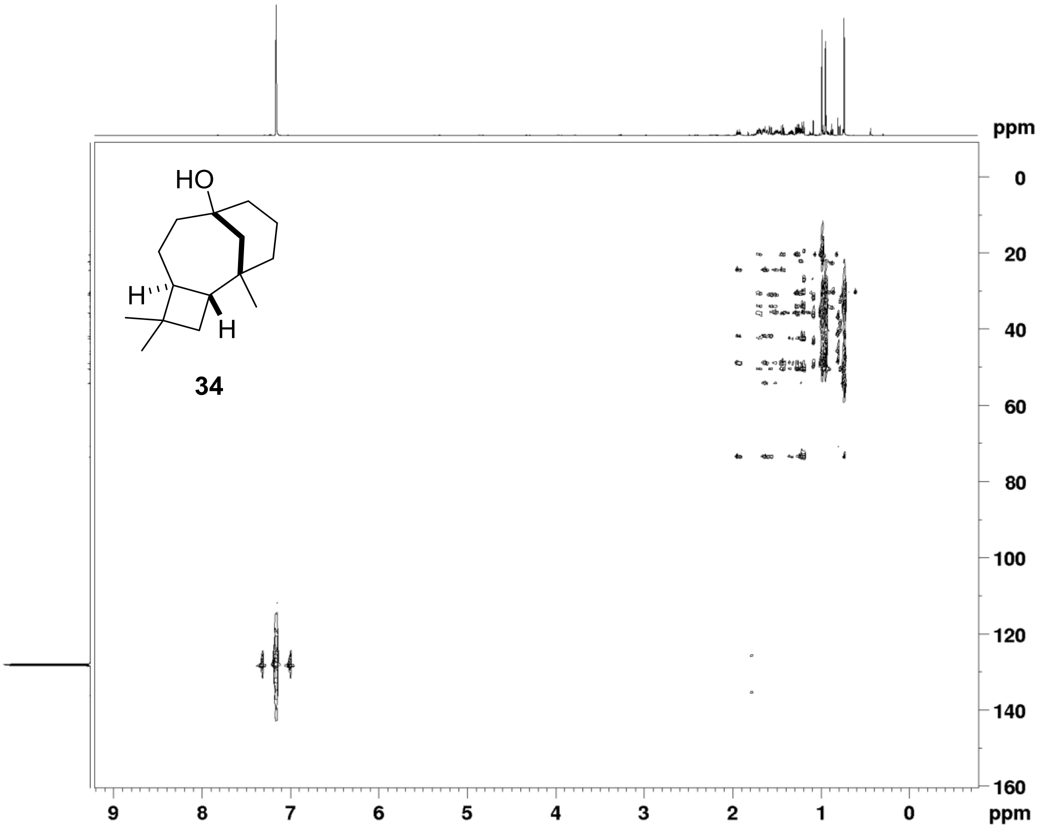

Figure S85. ^{1}H-^{13}C HMBC NMR spectrum of compound **34** in C_6D_6.

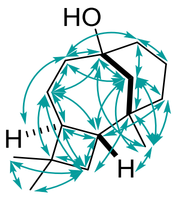

34

Figure S86. Key ^{1}H-^{13}C HMBC NMR correlations of **34** as indicated by green arrows.

In a previous publication we've published a diastereomer of **34** in which the 4-7 ring annelation is *anti*, too. Since we can't observe a ^{1}H-^{1}H NOESY correlation between 1.51 ppm and 1.70 ppm, instead correlations to opposing geminal Me groups (1.51 ppm to 0.95 ppm and 1.70 ppm to 0.99 ppm). This opens two possibilities referring to the relative stereochemistry: Either the CH_2 bridge is on the same side as the closer CH group (1.51 ppm), or on the opposing side. As the second would be the same (enantiomeric) molecule as already published before, **34** must have the proton at 1.51 ppm and the CH_2 bridge on the same side of the molecule. The superposition of multiple protons makes it hard to identify key correlations for the CH_2 bridge. Instead ^{1}H-^{1}H NOESY correlations between the methyl group (0.73 ppm) and the CH group (1.51 ppm) indicate the correct assignment if compared to the previously published structure. Comparing the correlations between the Me groups and CH groups in both

diastereomers, we observe a higher relative integral ratio for correlation signals in **34** than for **46** (analogous protons of the previously published diastereomer).

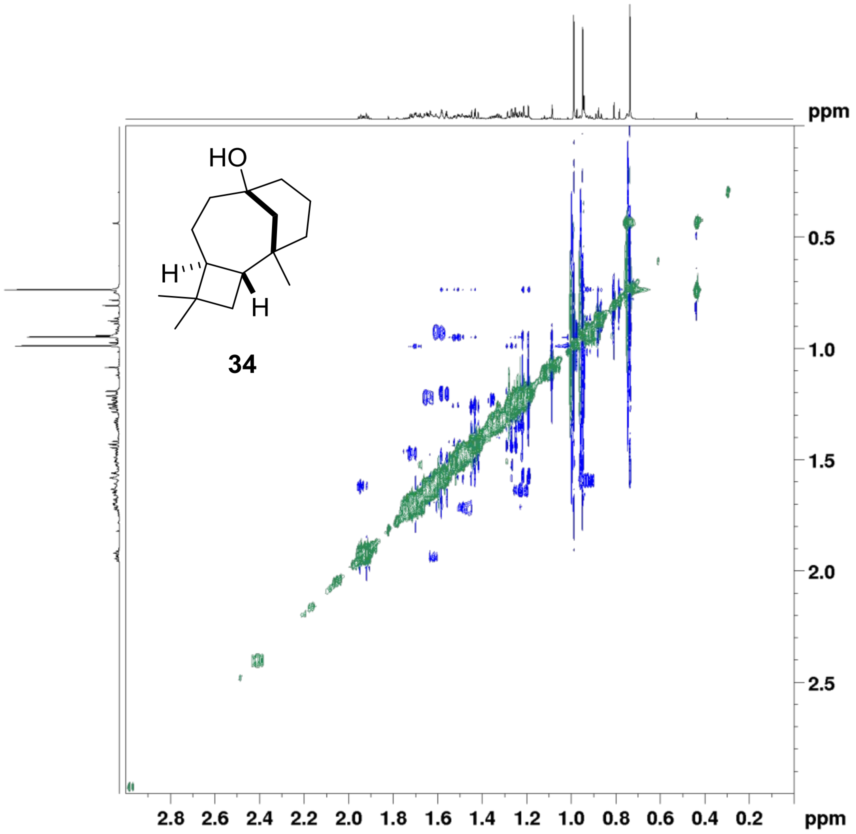

Figure S87. ^{1}H-^{1}H NOESY NMR spectrum of compound **34** in C_6D_6.

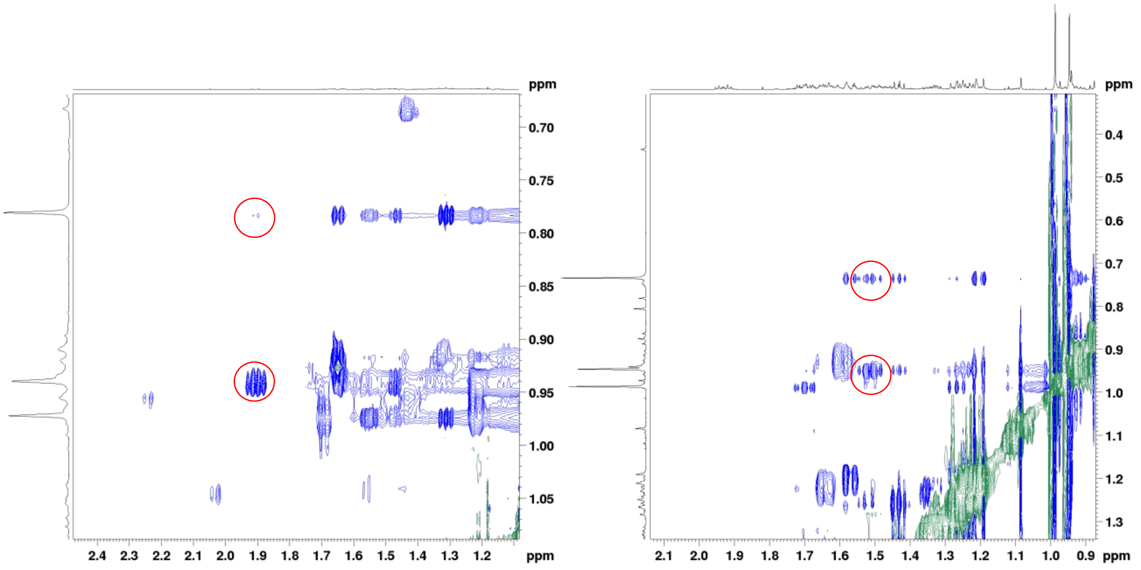

Figure S88. Zoom-In of ^{1}H-^{1}H NOESY NMR spectra of previously published compound **46** (left, *anti*) and this work's compound **34** (right, *syn*) in C_6D_6.

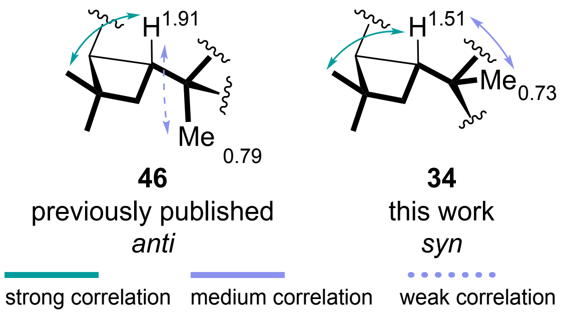

Figure S89. Key ^{1}H-^{1}H NOESY NMR correlations of previously published compound **46** (left, *anti*) and this work's compound **34** (right, *syn*) in C_6D_6.

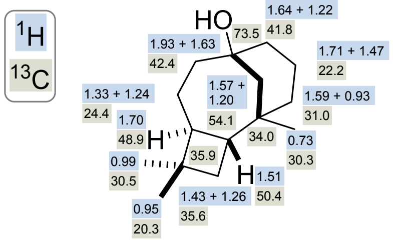

34

Figure S90. Full assignment of chemical shifts for compound **34**.

1.5.12 Structure elucidation of 37

To simplify structure elucidation only the centre of each HSQC signal will be used as the chemical shift, despite different multiplicities, since overlapping signals can be differentiated more easily.

Table S35. ^{1}H NMR signals and their corresponding ^{13}C NMR signals for compound **37** as analysed with the support of ^{1}H-^{13}C HSQC and $^{13}C\{^{1}H\}$ DEPT135 experiments. The quaternary carbon atoms are listed at the bottom.

δ (^{1}H)/ppm	δ (^{13}C)/ppm	DEPT135/HSQC phase
4.81 + 4.74	108.7	CH_2
1.89 + 0.71	44.0	CH_2
1.86	51.8	CH/CH_3
1.67	20.7	CH/CH_3
1.66 + 1.52	37.4	CH_2
1.65	28.2	CH_2
1.62 + 1.34	28.9	CH_2
1.50 + 1.18	41.1	CH_2
1.46 + 1.07	34.9	CH_2
1.42	20.9	CH_2
1.13 + 0.98	38.9	CH_2

δ (^{1}H)/ppm	δ (^{13}C)/ppm	DEPT135/HSQC phase
0.77	32.2	CH/CH_3
	152.4	C_q
	71.2	C_q
	34.2	C_q

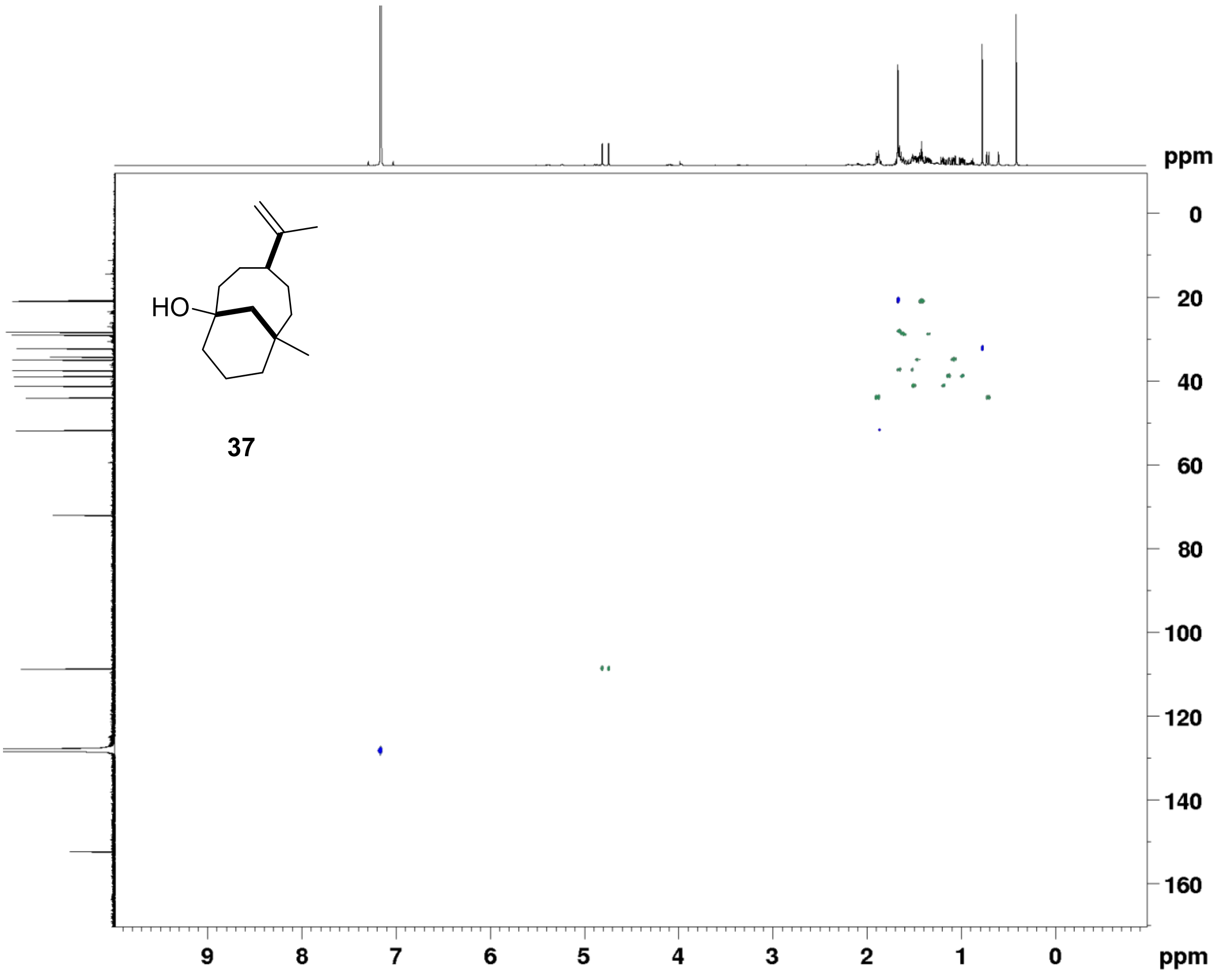

Figure S91. ^{1}H-^{13}C HSQC NMR spectrum of compound **37** in C_6D_6 (pos. phase = blue (CH/CH_3), neg. phase = green (CH_2)).

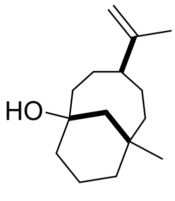

37

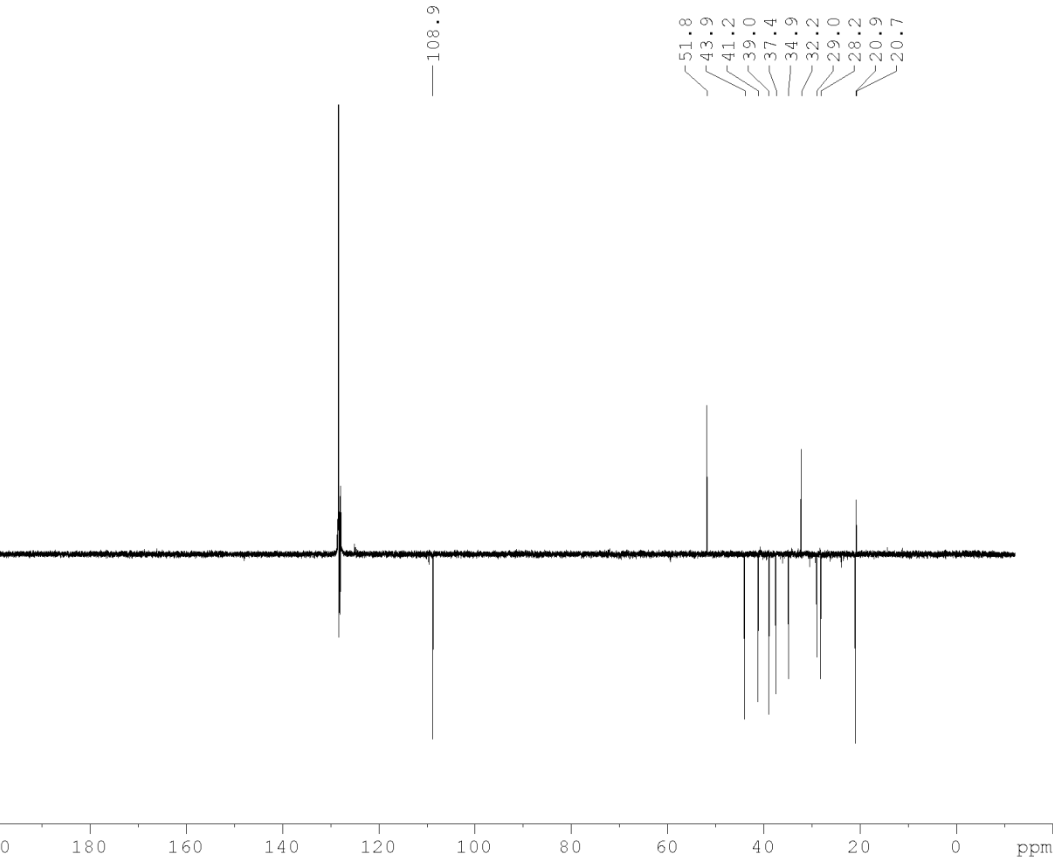

Figure S92. $^{13}C\{^{1}H\}$ DEPT135 NMR spectrum of compound **37** in C_6D_6.

Table S36. ^{1}H NMR signals and the corresponding ^{1}H-^{1}H COSY correlations for compound **37**. Signals with weak intensities are given in parentheses.

δ (^{1}H)/ppm	COSY correlations
4.81 + 4.74	1.67
1.89 + 0.71	(1.50, 1.13)
1.86	1.66, 1.34
1.67	
1.66 + 1.52	1.86
1.65	
1.62 + 1.34	1.07, (1.86, 1.46)
1.50 + 1.18	1.42
1.46 + 1.07	Hard to distinguish
1.42	1.18, 0.98
1.13 + 0.98	1.50, 1.42
0.77	1.46

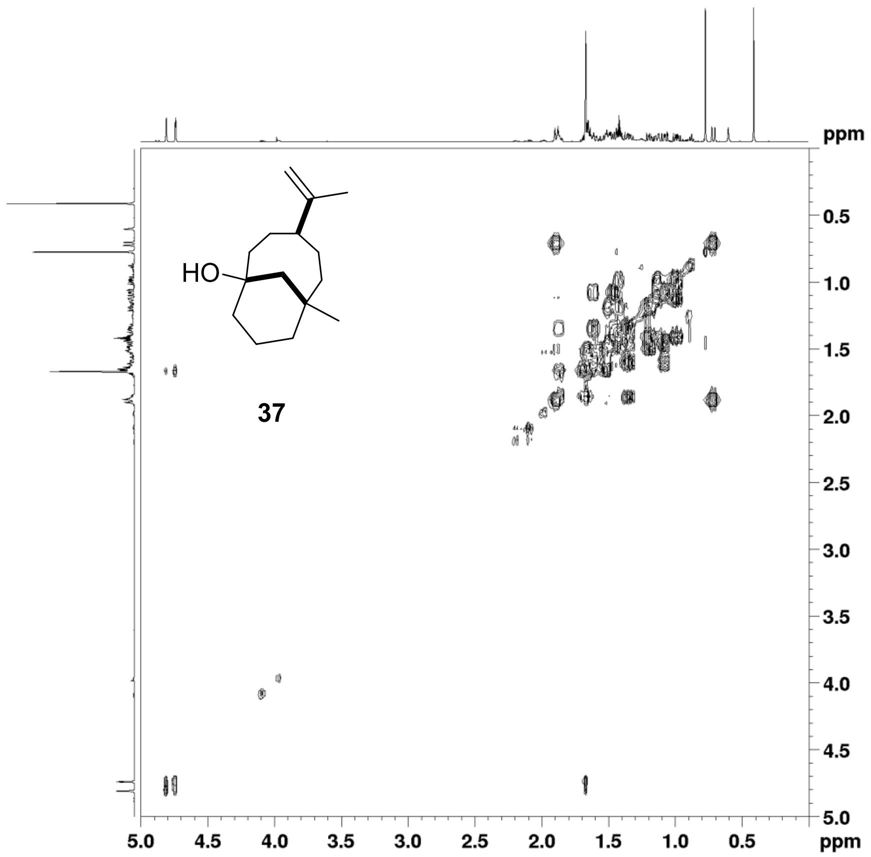

Figure S93. ^{1}H-^{1}H COSY NMR spectrum of compound **37** in C_6D_6.

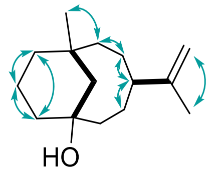

Figure S94. Key ^{1}H-^{1}H COSY NMR correlations of **37** as indicated by arrows.

Table S37. Selected correlations between ^{13}C NMR signals and neighbouring ^{1}H NMR signals as collected from the ^{1}H-^{13}C HMBC spectrum of compound **37**.

δ (^{1}H)/ppm	δ (^{13}C)/ppm
4.81 + 4.74	51.8, 20.7
1.89 + 0.71	72.0, 41.1, 38.9, 37.4, 34.1
1.86	
1.67	152.4, 108.7, 51.8
1.66 + 1.52	Hard to distinguish
1.65	Hard to distinguish
1.62 + 1.34	51.8, 34.1, 28.2
1.50 + 1.18	72.0, 37.4, 20.9

δ (^{1}H)/ppm	δ (^{13}C)/ppm
1.46 + 1.07	51.8, 44.0, 38.9, 34.1, 32.2
1.42	72.0, 41.1
1.13 + 0.98	44.0, 41.1, 34.8, 32.2, 20.9
0.77	44.0, 38.9, 34.9, 20.9, (72.0)

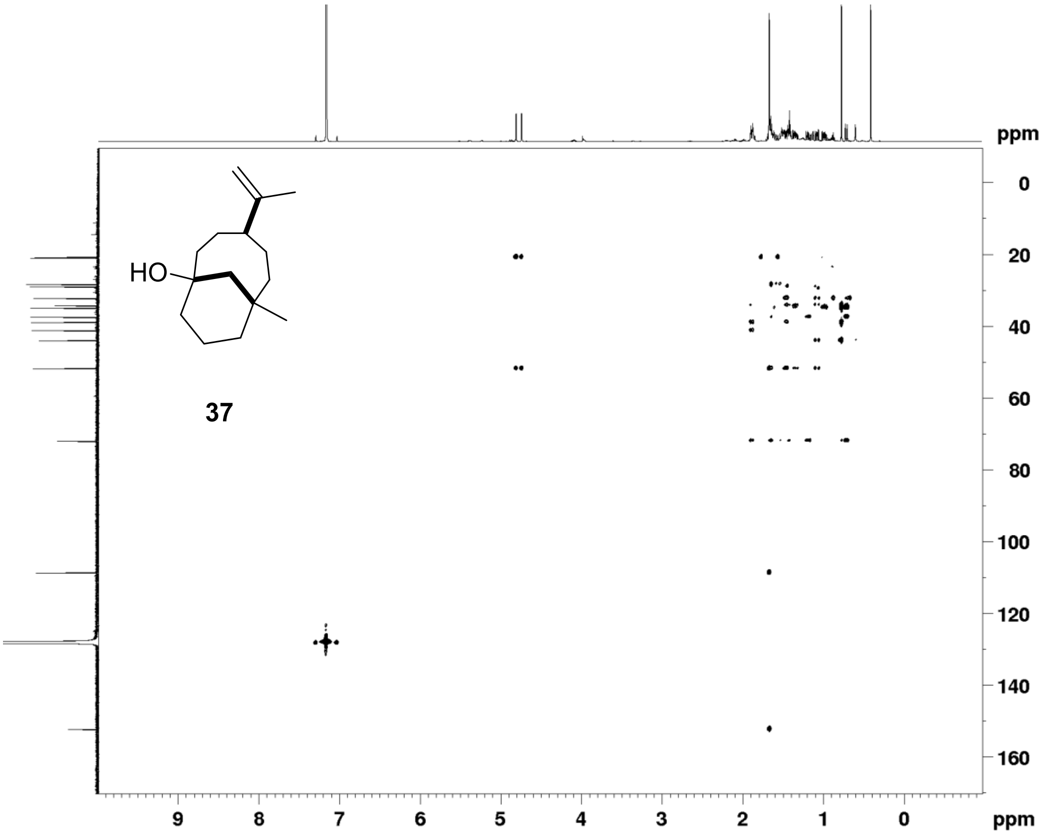

Figure S95. ^{1}H-^{13}C HMBC NMR spectrum of compound **37** in C_6D_6.

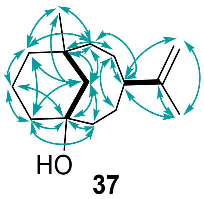

37

Figure S96. Key ^{1}H-^{13}C HMBC NMR correlations of **37** as indicated by green arrows.

The relative stereochemistry of **37** is analysed by ^{1}H-^{1}H NOESY NMR. In the two possible diastereomers the CH_2 bridge and the isopropylidene group are either *syn* or *anti* orientated. As **37** shows correlations between 1.89 ppm (CH_2 bridge) and 1.34 ppm (CH_2), 1.67 ppm (Me group) and 1.34 ppm (CH_2), as well as correlations between 1.86 ppm (CH group) and 4.81 ppm (olefinic CH_2), we believe that the *syn* diastereomer was formed.

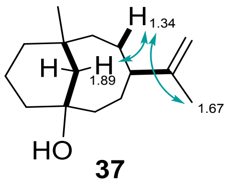

37

Figure S97. Key ^{1}H-^{1}H NOESY NMR correlations of **37** as indicated by arrows.

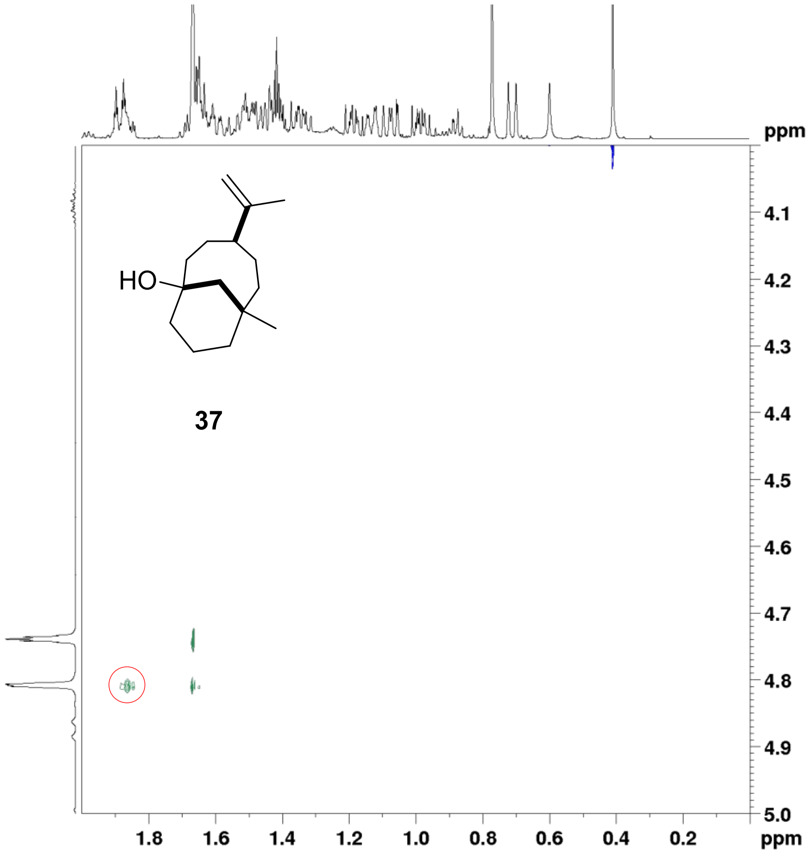

Figure S98. Zoom-In of ^{1}H-^{1}H NOESY NMR spectrum of compound **37** in C_6D_6. Key correlation marked in red circle.

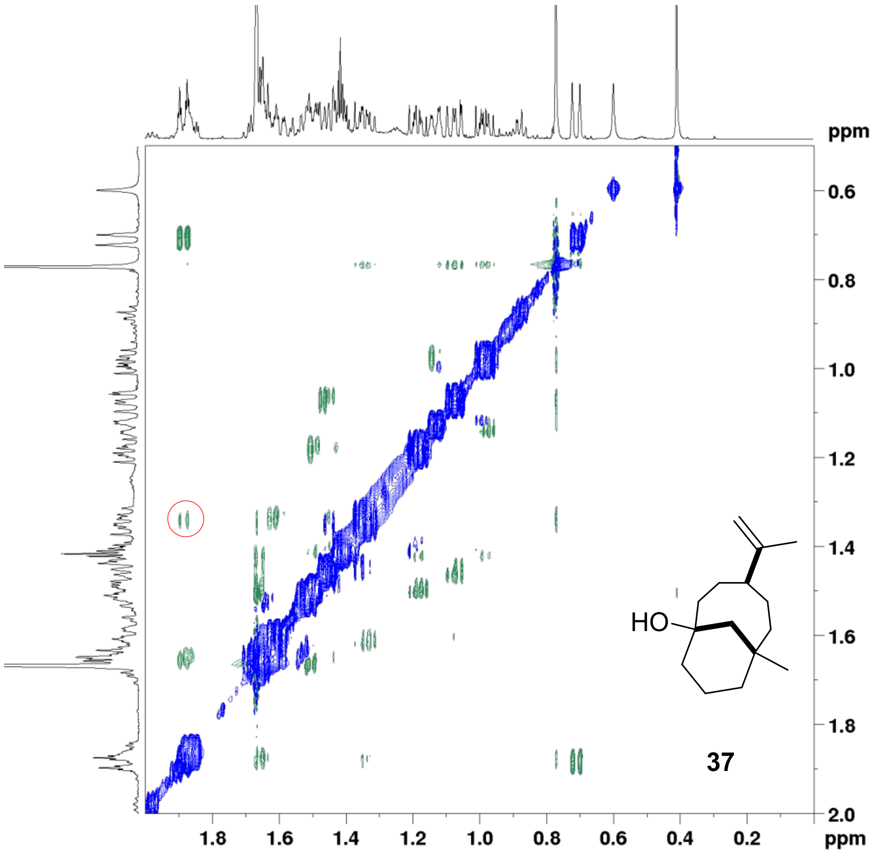

Figure S99. Zoom-In of ^{1}H-^{1}H NOESY NMR spectrum of compound **37** in C_6D_6. Key correlation marked in red circle.

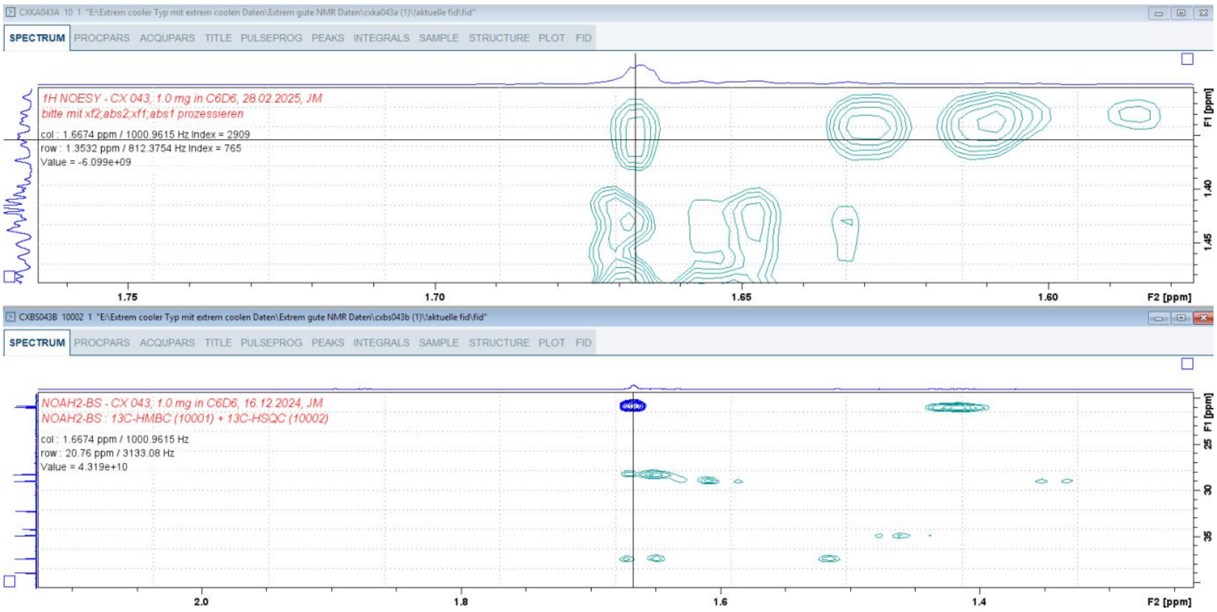

Figure S100. Screenshot of zoom-in of ^{1}H-^{1}H NOESY NMR (top) and ^{1}H-^{13}C HSQC (bottom) spectrum of compound **37** in C_6D_6 with respect to signal superposition of 1.6 ppm. Simultaneous view shows correlation of 1.34 ppm belongs to methyl group (1.67 ppm).

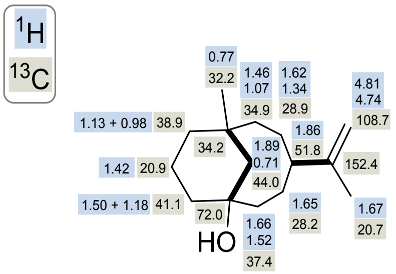

37

Figure S101. Full assignment of chemical shifts for compound **37**.

1.5.13 Structure elucidation of 38

To simplify structure elucidation only the centre of each HSQC signal will be used as the chemical shift, despite different multiplicities, since overlapping signals can be differentiated more easily.

Table S38. ^{1}H NMR signals and their corresponding ^{13}C NMR signals for compound **38** as analysed with the support of ^{1}H-^{13}C HSQC and $^{13}C\{^{1}H\}$ DEPT135 experiments. The quaternary carbon atoms are listed at the bottom.

δ (^{1}H)/ppm	δ (^{13}C)/ppm	DEPT135/HSQC phase
4.88	122.7	CH/CH_3
4.82 + 4.66	108.2	CH_2
2.12 + 1.83	42.0	CH_2
2.12 + 2.03	39.1	CH_2
1.98 + 1.55	31.0	CH_2
1.67 + 1.52	22.9	CH_2
1.60	17.1	CH/CH_3
1.55 + 1.29	22.1	CH_2
1.31	26.4	CH/CH_3
1.08	15.7	CH/CH_3
1.02	29.1	CH/CH_3
0.50	29.6	CH/CH_3
	147.9	C_q
	133.3	C_q
	18.8	C_q

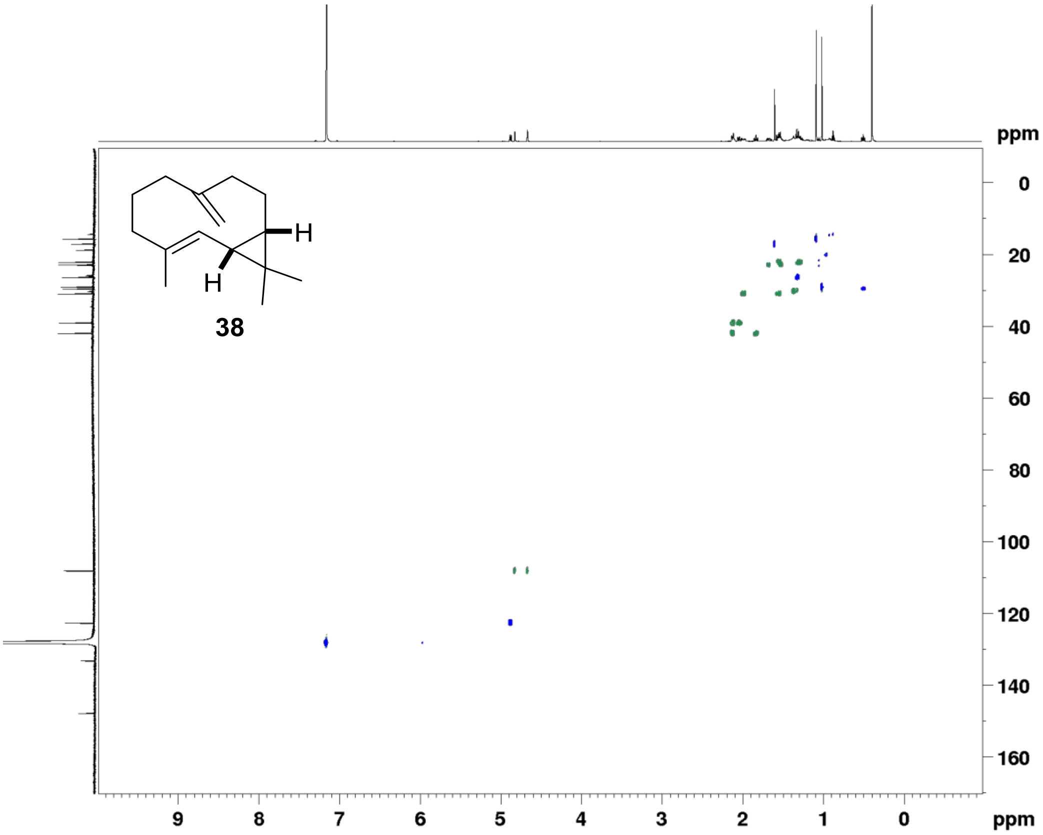

Figure S102. ^{1}H-^{13}C HSQC NMR spectrum of compound **38** in C_6D_6 (pos. phase = blue (CH/CH_3), neg. phase = green (CH_2)).

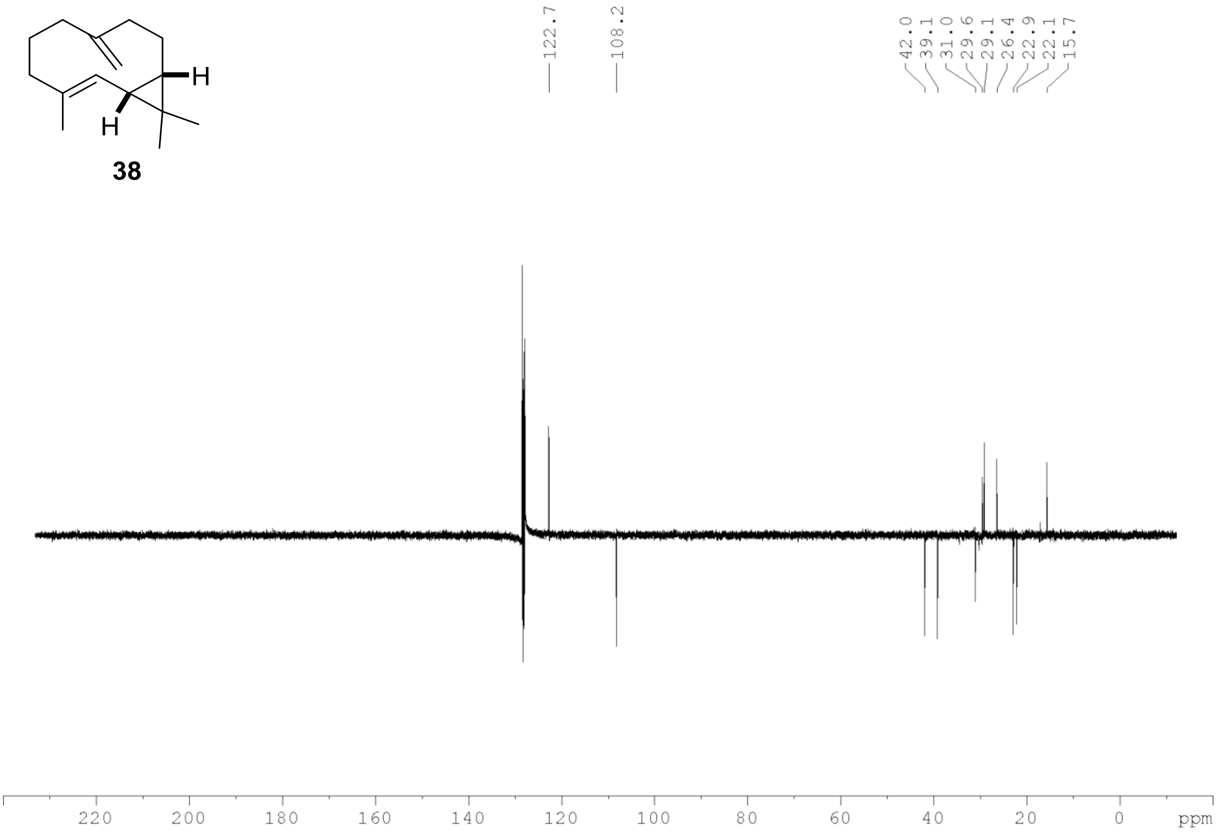

Figure S103. $^{13}C\{^{1}H\}$ DEPT135 NMR spectrum of compound **38** in C_6D_6.

Table S39. ^{1}H NMR signals and the corresponding ^{1}H-^{1}H COSY correlations for compound **38**. Signals with weak intensities are given in parentheses. Superposing signals which cannot be distinguished, are marked with "x"

δ (^{1}H)/ppm	COSY correlations
4.88	1.60, 1.31
4.82 + 4.66	2.12, 1.98, 1.55
2.12 + 1.83	1.67, 1.52
2.12 + 2.03	1.55, 1.31
1.98 + 1.55	4.82 + 4.66
1.67 + 1.52	2.12, 1.83, 1.5x, (1.98)
1.60	4.88, 2.12
1.55 + 1.29	2.12, 2.03, 0.50
1.31	4.88, 0.50
1.08	1.02
1.02	1.08
0.50	1.55, 1.31

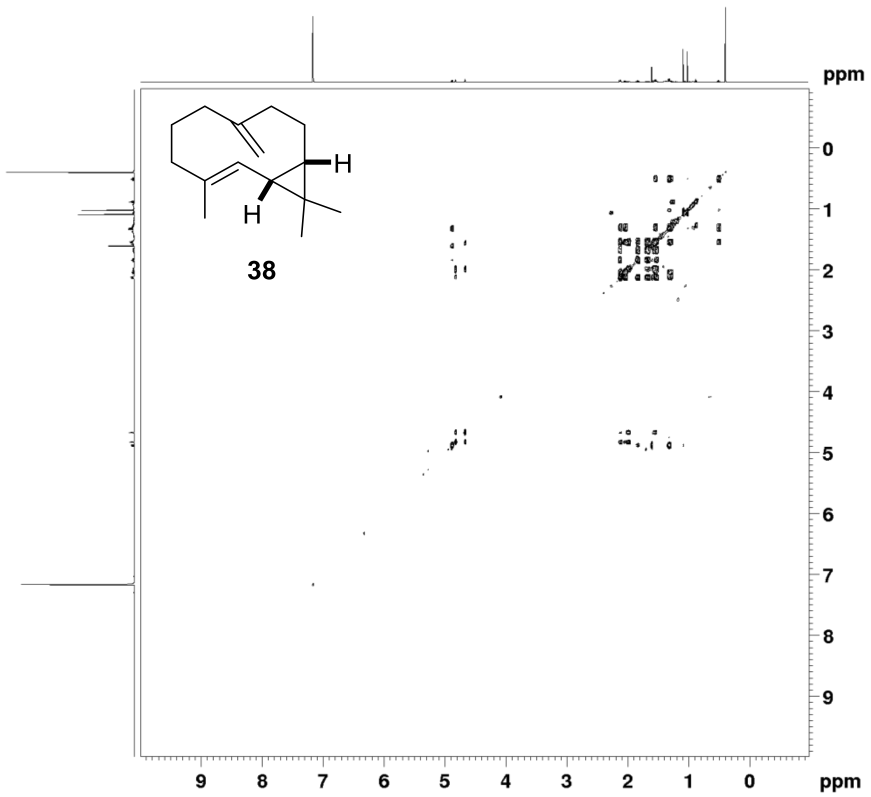

Figure S104. ^{1}H-^{1}H COSY NMR spectrum of compound **38** in C_6D_6.

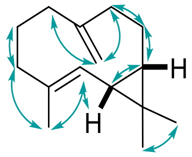

38

Figure S105. Key ¹H-¹H COSY NMR correlations of **38** as indicated by arrows.

Table S40. Selected correlations between ¹³C NMR signals and neighbouring ¹H NMR signals as collected from the ¹H-¹³C HMBC spectrum of compound **38**.

δ (¹H)/ppm	δ (¹³C)/ppm
4.88	42.0, 17.1
4.82 + 4.66	39.1, 31.0
2.12 + 1.83	133.3, 122.7, 31.0, 22.9, 17.1
2.12 + 2.03	147.9, 108.2, 31.0, 22.1
1.98 + 1.55	42.0
1.67 + 1.52	147.9
1.60	133.3, 122.7, 42.0
1.55 + 1.29	
1.31	133.3, 29.6 or 29.1
1.08	29.6 or 29.1, 26.4, 18.8
1.02	29.6, 26.4, 18.8, 15.7
0.50	(29.1)

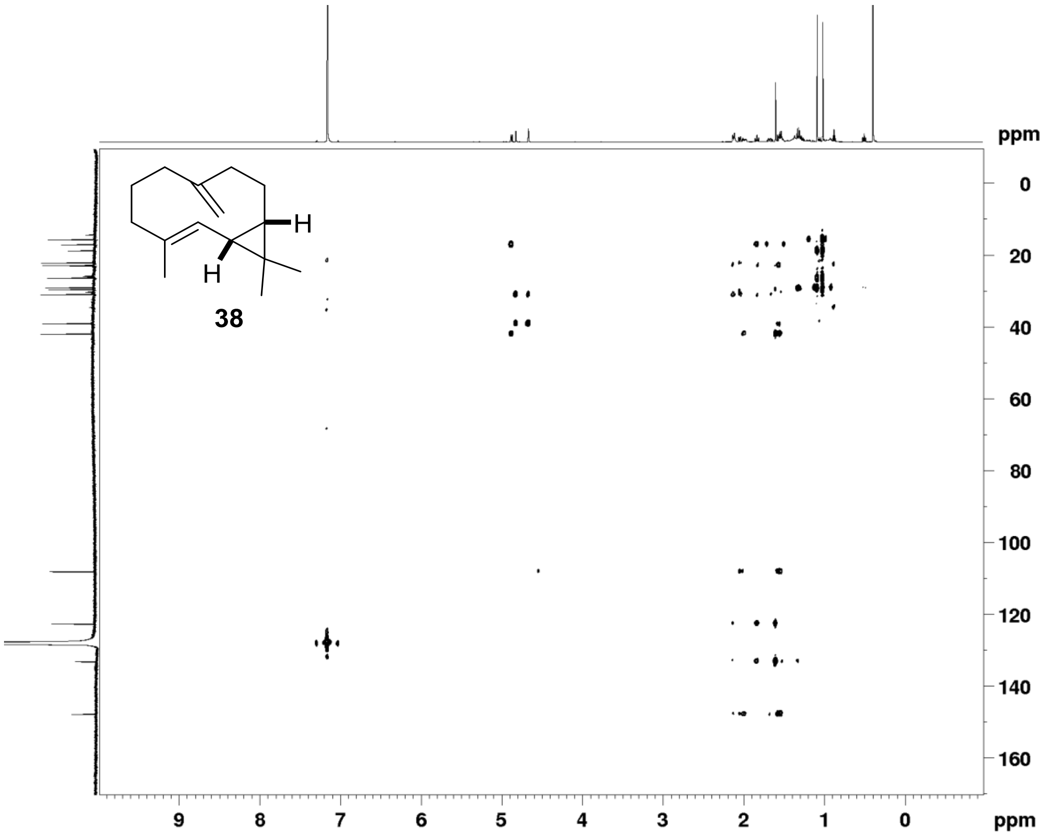

Figure S106. ^{1}H-^{13}C HMBC NMR spectrum of compound **38** in C_6D_6.

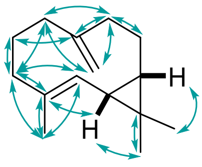

38

Figure S107. Key ^{1}H-^{13}C HMBC NMR correlations of **38** as indicated by arrows.

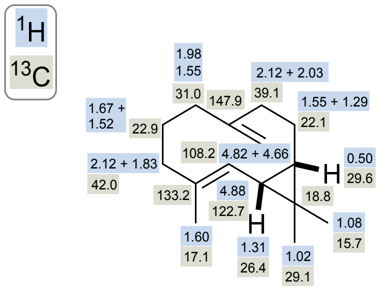

38

Figure S108. Full assignment of chemical shifts for compound **38**.

1.5.14 Structure elucidation of 32

Since **32** was elucidated by X-Ray the NMR spectra will be shown below without detailed analysis.

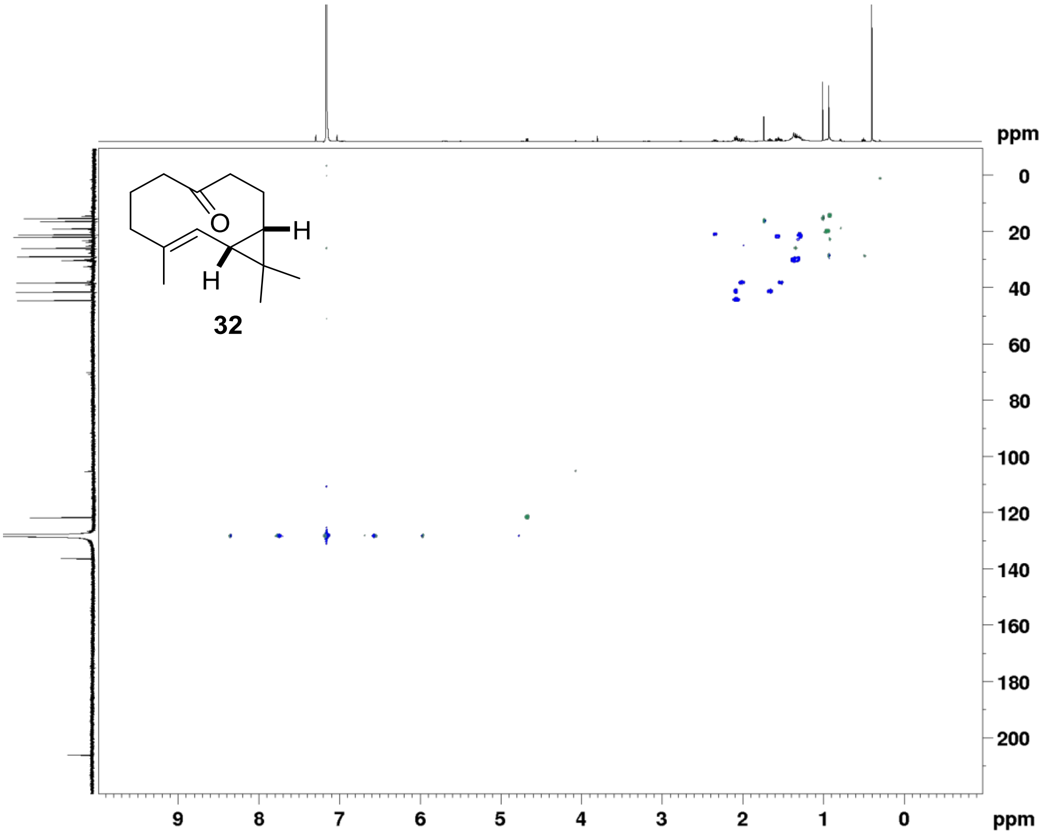

Figure S109. ${}^{1}H$-${}^{13}C$ HSQC NMR spectrum of compound **32** in C_6D_6 (pos. phase = blue (CH/CH_3), neg. phase = green (CH_2)).

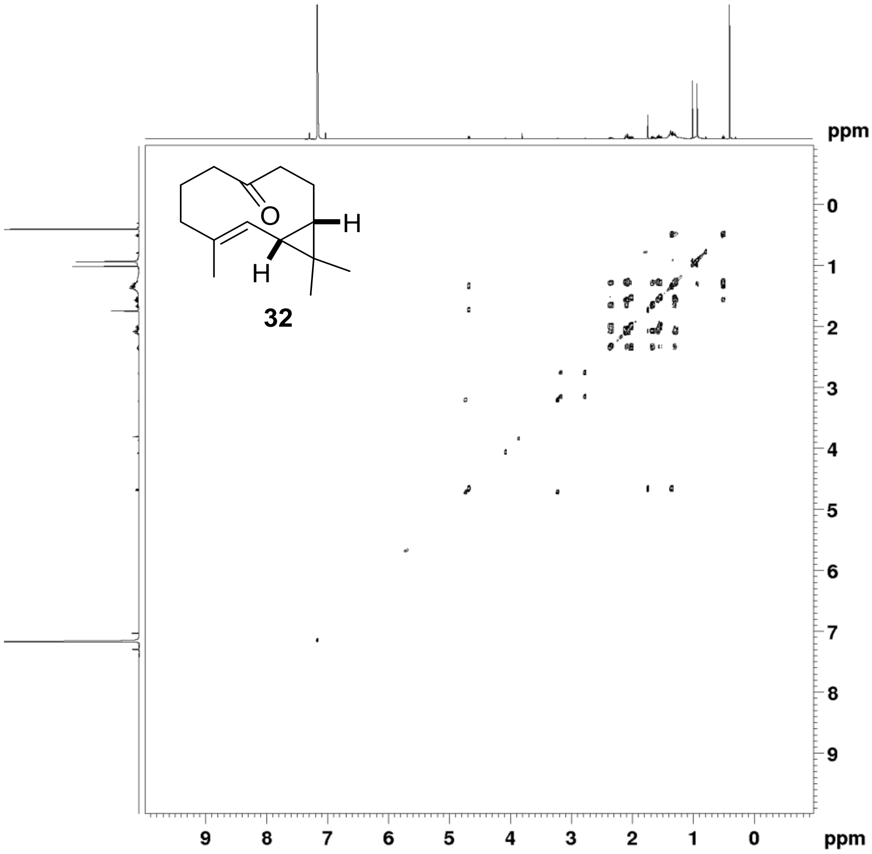

Figure S110. ${}^{1}H$-${}^{1}H$ COSY NMR spectrum of compound **32** in C_6D_6.

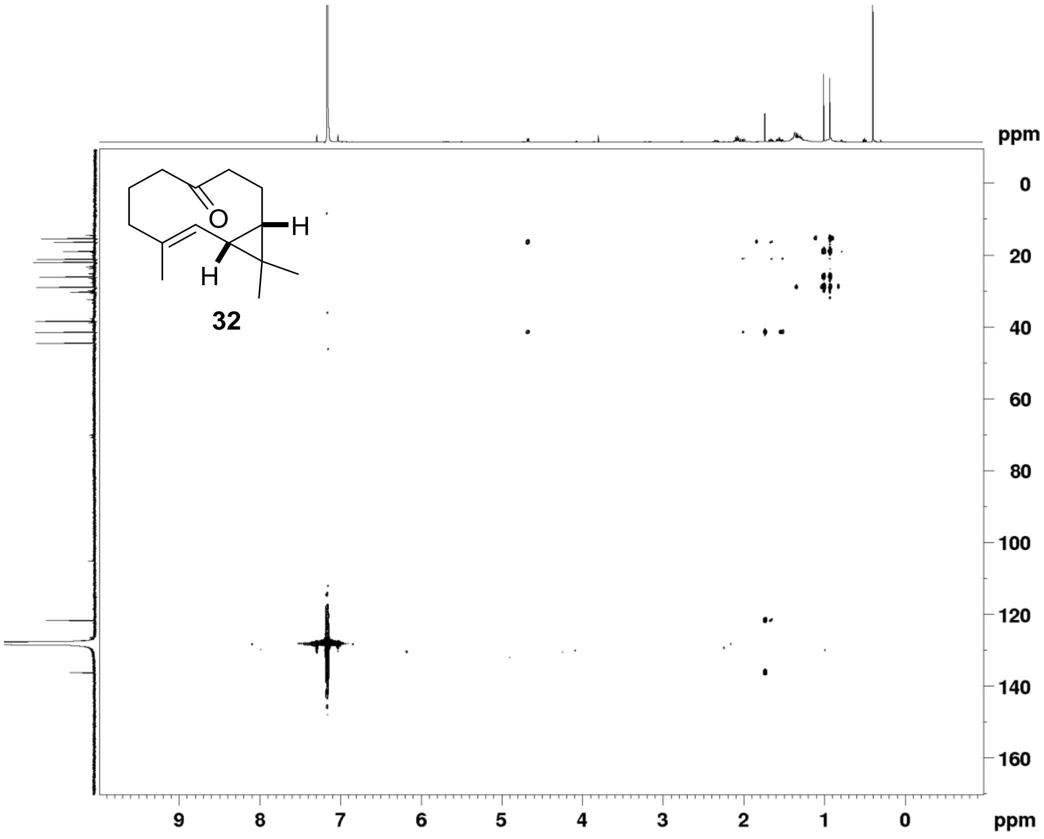

Figure S111. ^{1}H-^{13}C HMBC NMR spectrum of compound **32** in C_6D_6.

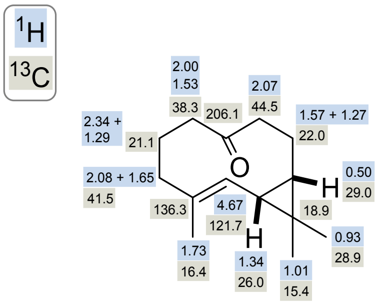

32

Figure S112. Full assignment of chemical shifts for compound **32**.

1.5.15 Structure elucidation of 44

To simplify structure elucidation only the centre of each HSQC signal will be used as the chemical shift, despite different multiplicities, since overlapping signals can be differentiated more easily.

Table S41. ^{1}H NMR signals and their corresponding ^{13}C NMR signals for compound **44** as analysed with the support of ^{1}H-^{13}C HSQC and $^{13}C\{^{1}H\}$ DEPT135 experiments. The quaternary carbon atoms are listed at the bottom.

δ (^{1}H)/ppm	δ (^{13}C)/ppm	DEPT135/HSQC phase
2.43	34.4	CH/CH_3

δ (^1H)/ppm	δ (^{13}C)/ppm	DEPT135/HSQC phase
2.18	41.6	CH/CH_3
1.84 + 1.53	27.9	CH_2
1.84 + 1.41	16.9	CH_2
1.82	50.9	CH/CH_3
1.71 + 1.37	40.6	CH_2
1.66 + 1.20	50.4	CH_2
1.54 + 1.42	40.3	CH_2
1.18	32.2	CH/CH_3
1.15	29.5	CH/CH_3
0.99	27.6	CH/CH_3
0.86	27.1	CH/CH_3
1.71	74.3	C_q
	38.2	C_q
	36.9	C_q

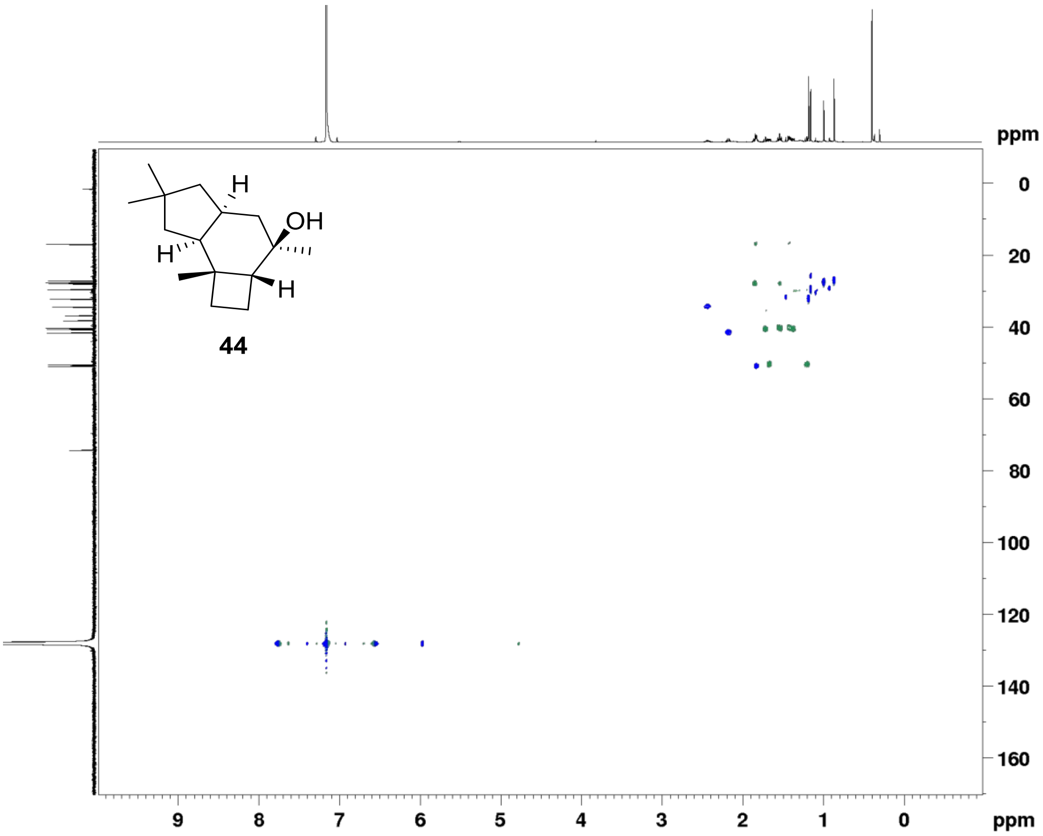

Figure S113. ^1H-^{13}C HSQC NMR spectrum of compound **44** in C_6D_6 (pos. phase = blue (CH/CH_3), neg. phase = green (CH_2)).

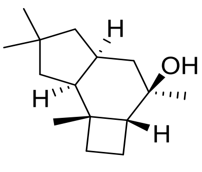

44

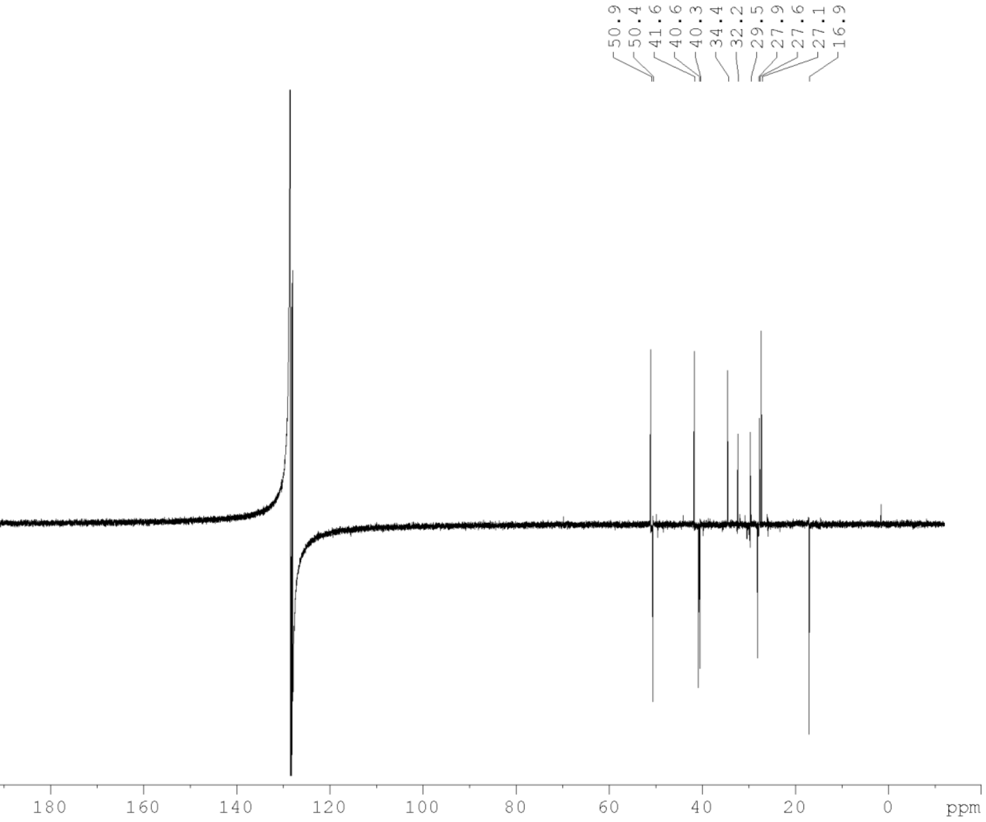

Figure S114. $^{13}C\{^1H\}$ DEPT135 NMR spectrum of compound **44** in C_6D_6.

Table S42. ^1H NMR signals and the corresponding ^1H-^1H COSY correlations for compound **44**. Signals with weak intensities are given in parentheses.

δ (^1H)/ppm	COSY correlations
2.43	2.18, 1.66 + 1.20, 1.54 + 1.42
2.18	2.43, 1.71 + 1.37
1.84 + 1.53	Hard to distinguish
1.84 + 1.41	Hard to distinguish
1.82	Hard to distinguish
1.71 + 1.37	2.18, 0.99
1.66 + 1.20	2.43, 0.99 (1.37)
1.54 + 1.42	Hard to distinguish
1.18	-
1.15	0.99
0.99	1.71, 1.20, 1.15
0.86	-

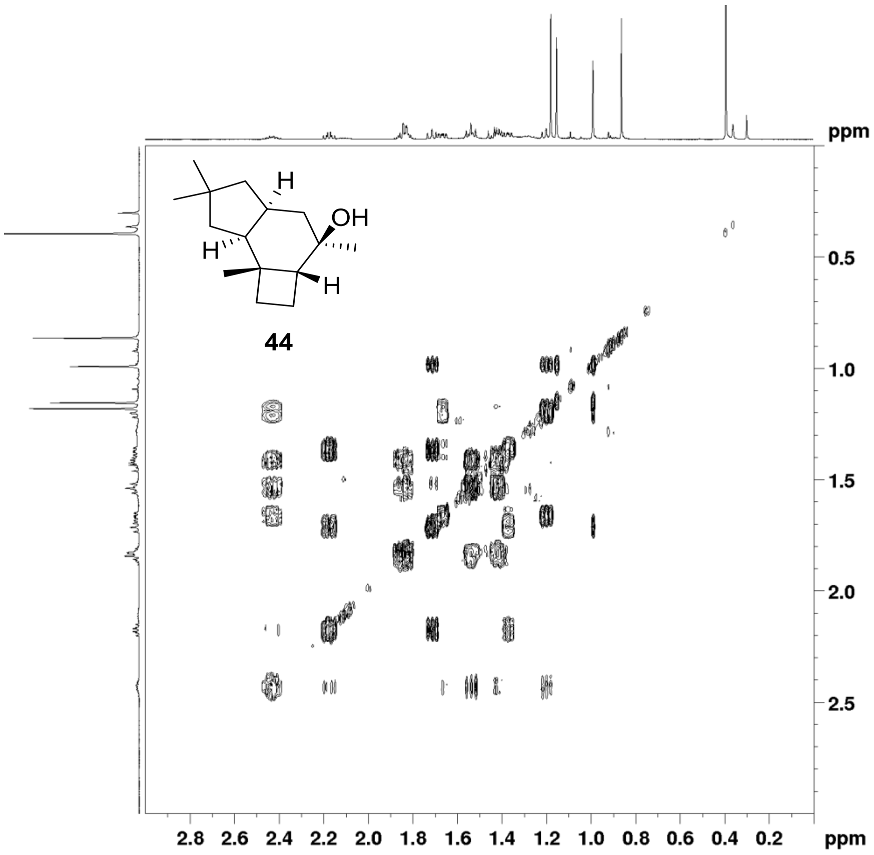

Figure S115. ^{1}H-^{1}H COSY NMR spectrum of compound **44** in C_6D_6.

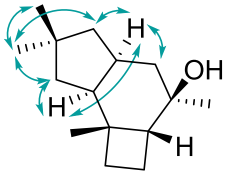

44

Figure S116. Key ^{1}H-^{1}H COSY NMR correlations of **44** as indicated by arrows.

Table S43. Selected correlations between ^{13}C NMR signals and neighbouring ^{1}H NMR signals as collected from the ^{1}H-^{13}C HMBC spectrum of compound **44**. Existence of hydroxy group based on ^{1}H-^{13}C HMBC correlation of 1.71 ppm and 74.3 ppm and ^{1}H-^{1}H NOESY correlation signals with water residues (see below).

δ (^{1}H)/ppm	δ (^{13}C)/ppm
2.43	-
2.18	40.6, 34.4
1.84 / 1.84 / 1.82	74.3, 40.3, 36.9, 32.2, 27.9, 16.9
1.71 + 1.37	50.4, 41.6, 38.2, 34.4, 29.5, 27.6
1.71 (OH group)	74.3
1.66 + 1.20	40.6, 40.3, 38.2, 34.4, 27.6, (29.6)
1.54 / 1.53	50.4, 36.9, 34.4, 32.2, 27.9, 16.9

δ (^{1}H)/ppm	δ (^{13}C)/ppm
1.42 / 1.41	50.9, 41.6, 36.9, 34.4, 27.9, 27.6
1.18	50.9, 40.3, 36.9, 27.9
1.15	50.4, 40.6, 38.2, 27.6
0.99	50.4, 40.6, 38.2, 29.5
0.86	74.3, 50.9, 41.6

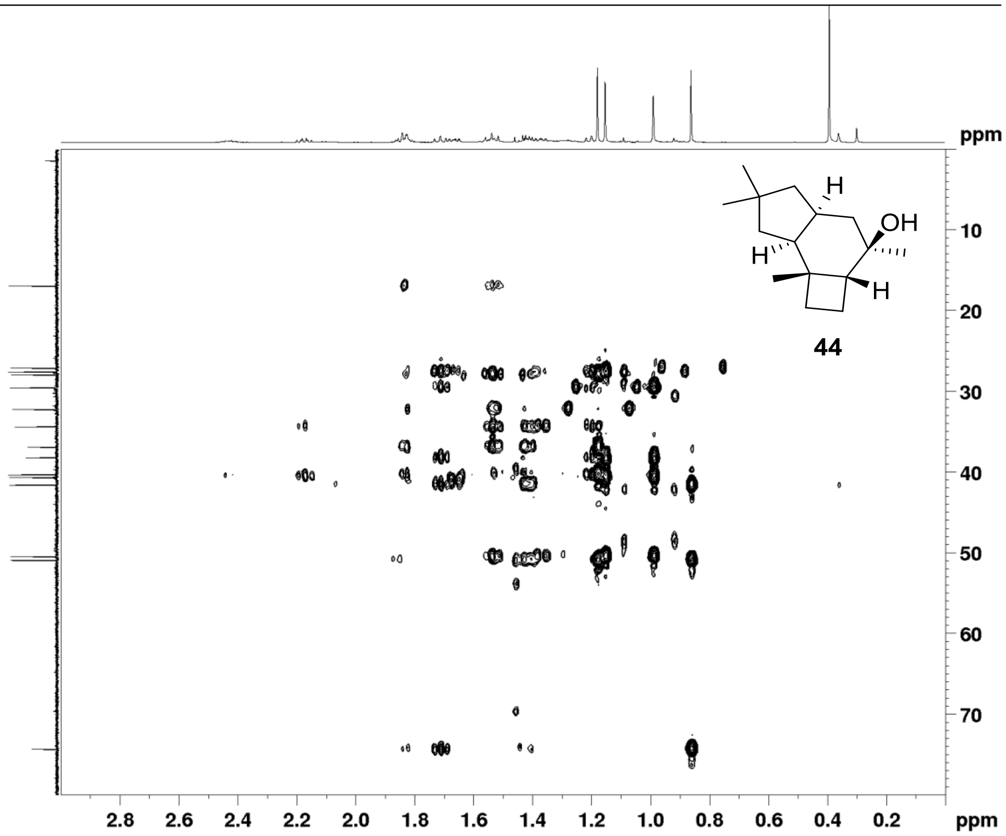

Figure S117. ^{1}H-^{13}C HMBC NMR spectrum of compound **44** in C_6D_6.

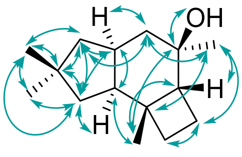

44

Figure S118. Key ^{1}H-^{13}C HMBC NMR correlations of **44** as indicated by arrows.

^{1}H-^{1}H NOESY analysis generally fits the proposed stereochemistry well. Some key signals will be shown here. Since the alcoholic proton exchanges with water, the water signal shows correlation to the molecule: 1.71 ppm (alcohol), 1.82 ppm (CH group), 1.54 ppm (CH_2 group). Additionally, there is no correlation between the two methyl groups (0.86 ppm and 1.18 ppm). Since the two CH groups (2.18 ppm and 2.43 ppm) show correlation to the same geminal methyl group (0.99 ppm) they can be considered *syn*. Since no correlation between the alcoholic proton and the CH groups (2.18 ppm and 2.43 ppm) can be observed nor a strong correlation between 2.18 ppm and 1.82 ppm, we believe that the relative stereochemistry of 1.82 ppm and

2.18 ppm is *anti*. A correlation between 1.82 ppm and 1.18 ppm is also plausible in a relative *anti* orientation. This relative stereochemistry is supported by the spacial proximity of 1.20 ppm and 1.54 ppm.

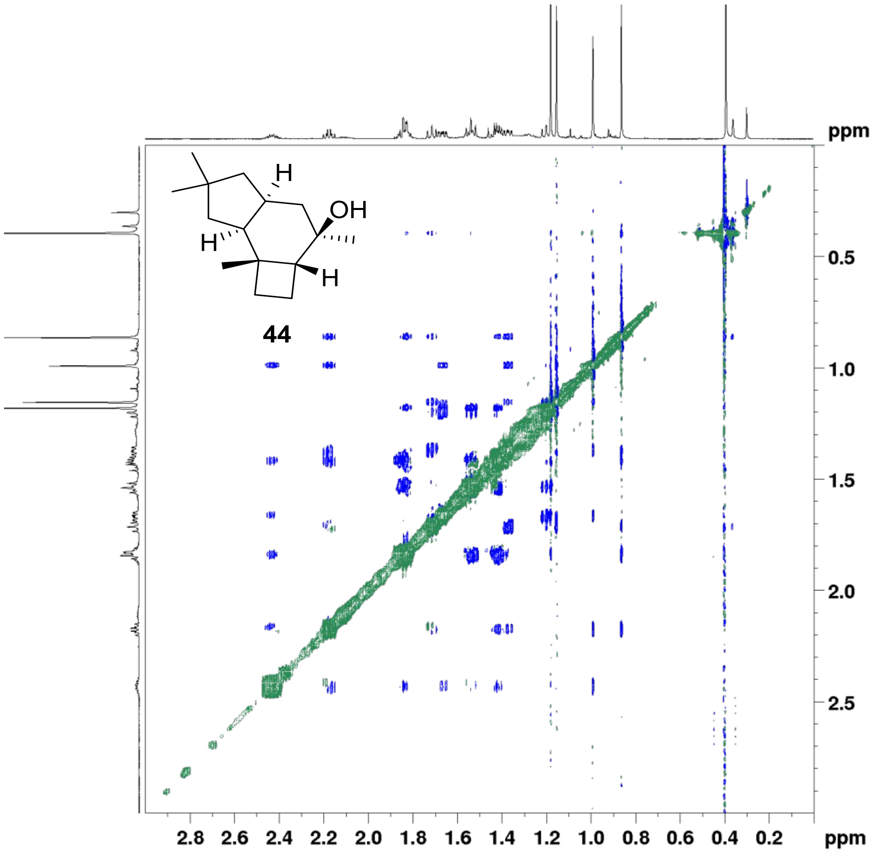

Figure S119. ^{1}H-^{1}H NOESY NMR spectrum of compound **44** in C_6D_6.

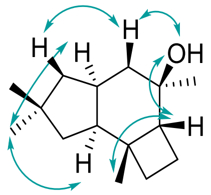

44

Figure S120. Key ^{1}H-^{1}H NOESY NMR correlations of **44** as indicated by arrows.

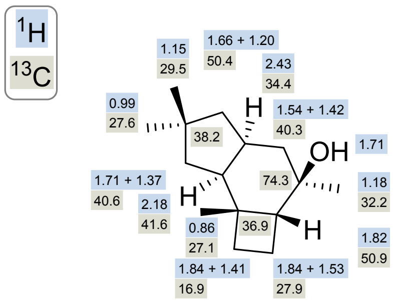

44

Figure S121. Full assignment of chemical shifts for compound **44**.

44 is known to literature, but only chemical shifts of methyl groups are given, while the other signals have been reported with shift reagents in a different solvent. [S16]

1.5.16 Structure elucidation of 42 in the presence of 43

To simplify structure elucidation only the centre of each HSQC signal will be used as the chemical shift, despite different multiplicities, since overlapping signals can be differentiated more easily.

Table S44. ^{1}H NMR signals and their corresponding ^{13}C NMR signals for compound **42** as analysed with the support of ^{1}H-^{13}C HSQC and $^{13}C\{^{1}H\}$ DEPT135 experiments. The quaternary carbon atoms are listed at the bottom.

δ (^{1}H)/ppm	δ (^{13}C)/ppm	DEPT135/HSQC phase
2.66	38.1	CH/CH_3
2.38 + 1.53	25.1	CH_2
2.13	44.7	CH_2
2.10	45.0	CH/CH_3
2.01 + 1.48	28.2	CH_2
1.63 + 1.10	48.9	CH_2
1.58	18.1	CH/CH_3
1.52 + 0.73	39.6	CH_2
1.21	29.7	CH/CH_3
1.08	30.4	CH/CH_3
1.04	29.4	CH/CH_3
	137.1	C_q
	127.5	C_q
	38.2	C_q
	37.1	C_q

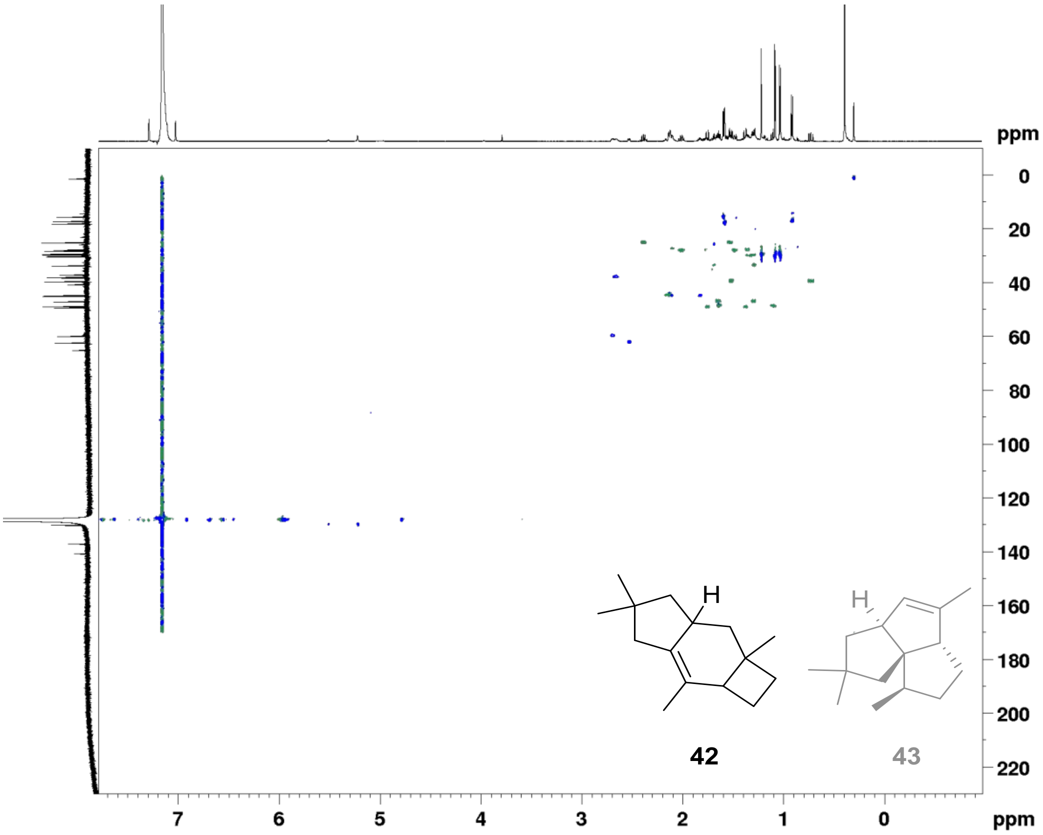

Figure S122. ^{1}H-^{13}C HSQC NMR spectrum of compound **42** in the presence of **43** in C_6D_6 (pos. phase = blue (CH/CH_3), neg. phase = green (CH_2)).

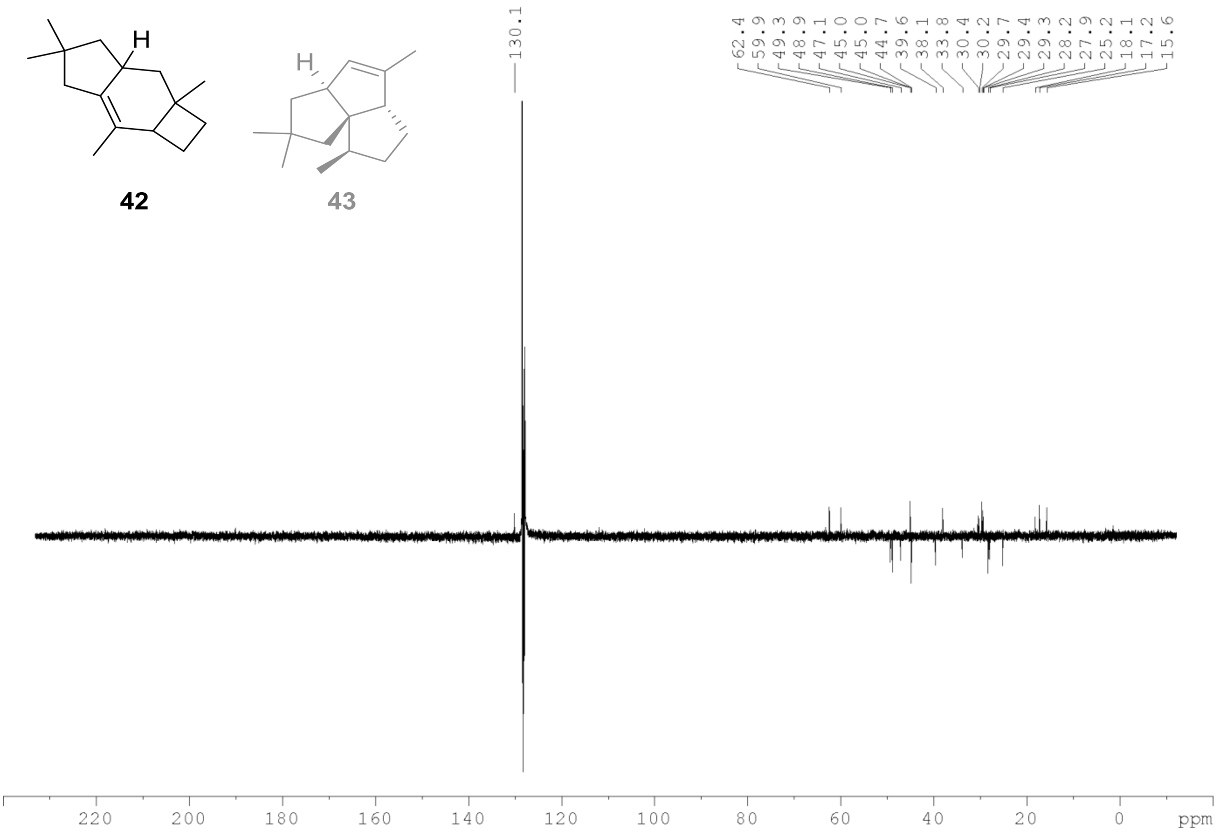

Figure S123. $^{13}C\{^{1}H\}$ DEPT135 NMR spectrum of compound **42** in the presence of **43** in C_6D_6.

Table S45. ^1H NMR signals and the corresponding ^1H-^1H COSY correlations for compound **42**. Signals with weak intensities are given in parentheses.

δ (^1H)/ppm	COSY correlations
2.66	1.52, (1.63)
2.38 + 1.53	2.01 + 1.48, 2.10
2.13	1.58
2.10	2.38 + 1.53, 2.01 + 1.48
2.01 + 1.48	2.38 + 1.53, 1.21, (2.10)
1.63 + 1.10	2.66, 2.13, 1.04
1.58	2.66, 2.13
1.52 + 0.73	2.66
1.21	2.01 + 1.48
1.08	-
1.04	-

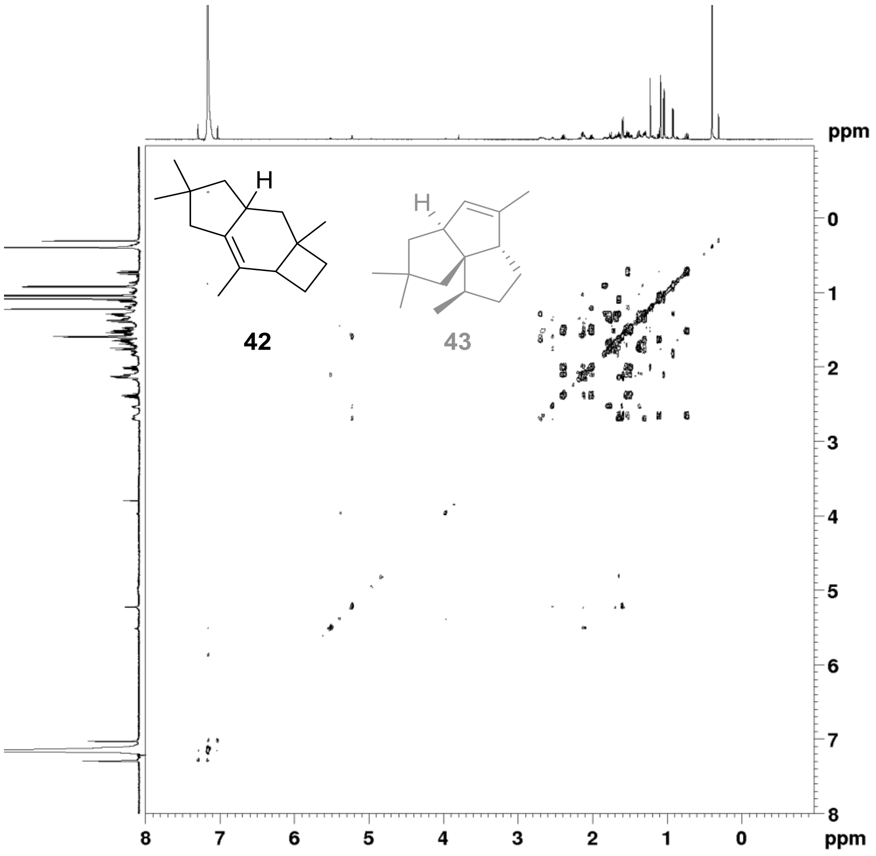

Figure S124. ^1H-^1H COSY NMR spectrum of compounds **42** in the presence of **43** in C_6D_6.

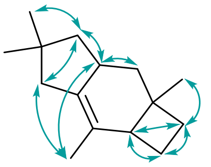

42

Figure S125. Key ^{1}H-^{1}H COSY NMR correlations of **42** as indicated by arrows.

Table S46. Selected correlations between ^{13}C NMR signals and neighbouring ^{1}H NMR signals as collected from the ^{1}H-^{13}C HMBC spectrum of compound **42**.

δ (^{1}H)/ppm	δ (^{13}C)/ppm
2.66	
2.38 + 1.53	127.5, 45.0
2.13	
2.10	
2.01 + 1.48	39.6, 38.2, 25.1
1.63 + 1.10	137.1, 44.7, 29.4
1.58	137.1, 127.5
1.52 + 0.73	137.1, 38.1
1.21	45.0, 39.6, 38.2, 28.2
1.08	48.9, 44.7, 37.1, 29.4
1.04	48.9, 44.7, 37.1, 30.4

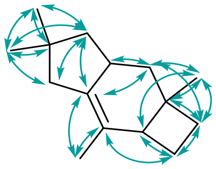

42

Figure S126. Key ^{1}H-^{13}C HMBC NMR correlations of **42** as indicated by arrows.

Key signals for this ^{1}H-^{1}H NOESY NMR analysis correlations between one of the geminal methyl groups (1.04 ppm) and the CH group (2.66 ppm) as well as the correlation between the same CH group (2.66 ppm) and one of the cyclobutene protons (2.01 ppm) and one of both vicinal CH_2 groups (1.52 ppm and 1.63 ppm). The other side of the molecule can be analysed based on the correlations between the single methyl group (1.21 ppm) and other protons. Strong correlations are observed for a CH group (2.10 ppm), likely 1.48 ppm and 1.52 ppm, and 0.73 ppm.

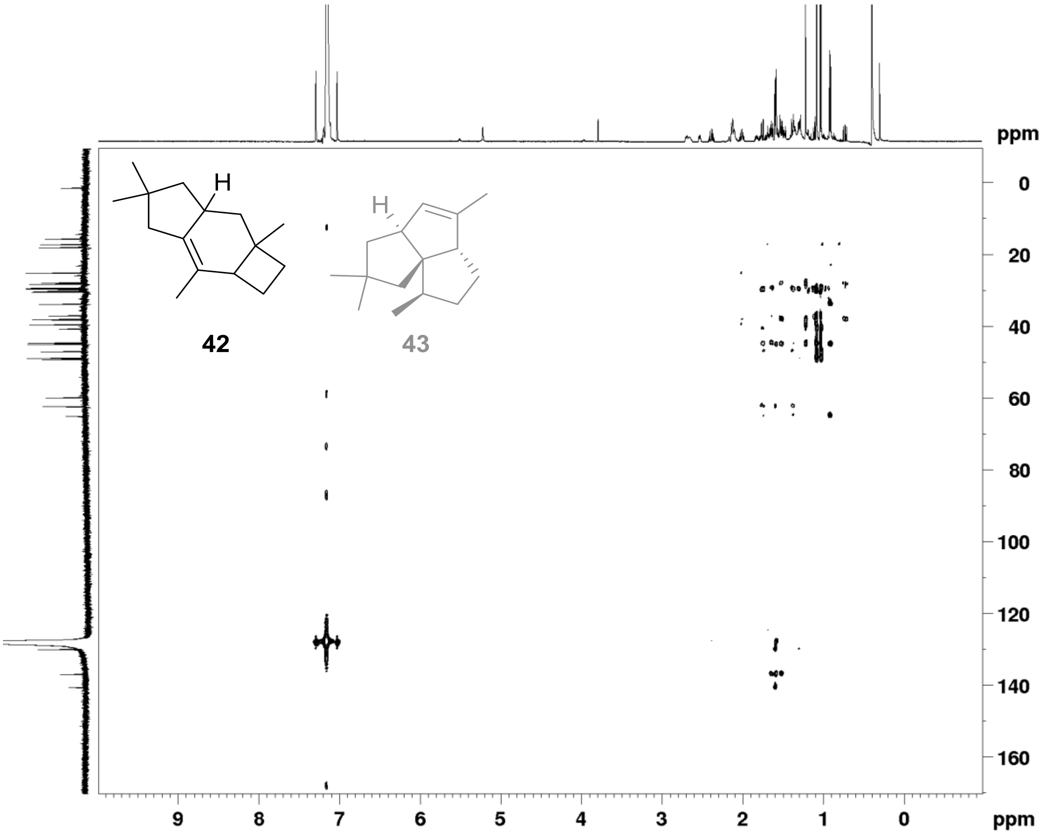

Figure S127. ^{1}H-^{13}C HMBC NMR spectrum of compounds **42** in the presence of **43** in C_6D_6.

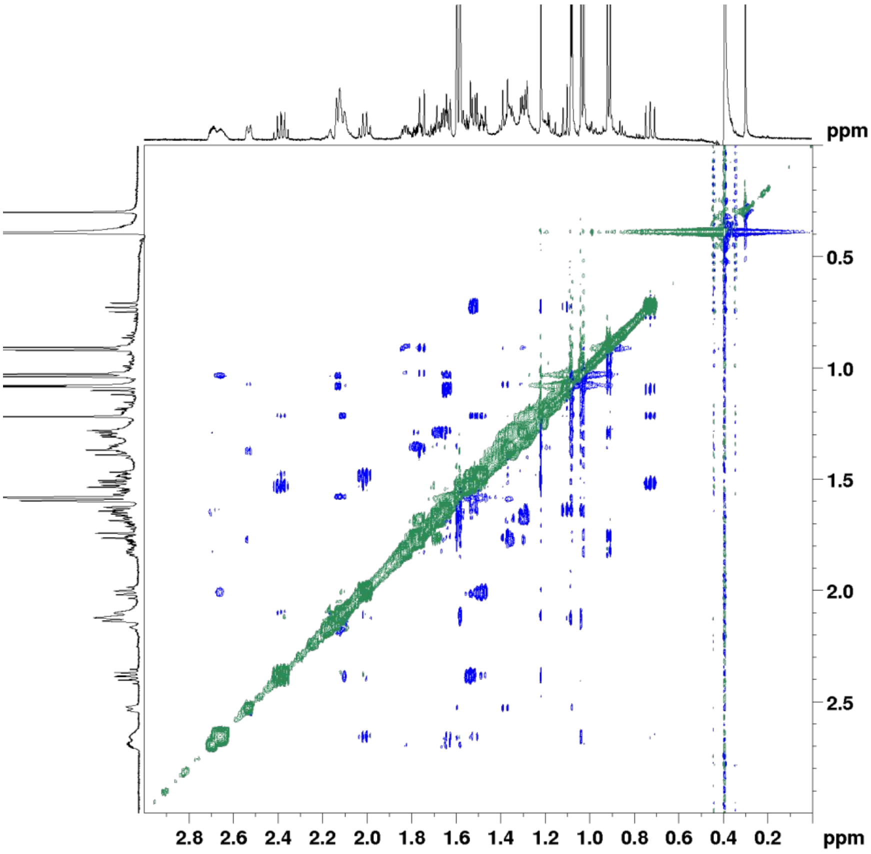

Figure S128. ^{1}H-^{1}H NOESY NMR spectrum of compound **42** in the presence of **43** in C_6D_6.

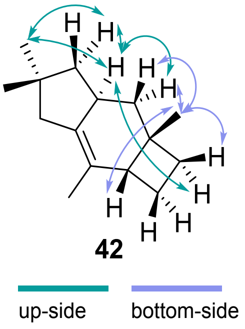

Figure S129. Key ^{1}H-^{1}H NOESY NMR correlations of **42** as indicated by arrows.

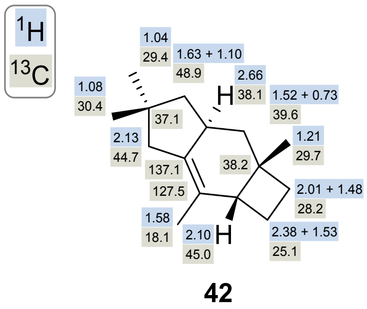

Figure S130. Full assignment of chemical shifts for compound **42**.

1.5.17 Structure elucidation of 35

To simplify structure elucidation only the centre of each HSQC signal will be used as the chemical shift, despite different multiplicities, since overlapping signals can be differentiated more easily. During structure elucidation solvents were present which doesn't interfere with the analysis. Clean ^{1}H and ^{13}C spectra are given below.

Table S47. ^{1}H NMR signals and their corresponding ^{13}C NMR signals for compound **35** as analysed with the support of ^{1}H-^{13}C HSQC experiments. The quaternary carbon atoms are listed at the bottom.

δ (^{1}H)/ppm	δ (^{13}C)/ppm	HSQC phase
3.02 + 1.60	44.3	CH_2
2.51 + 1.70	35.3	CH_2
2.21 + 1.61	43.8	CH_2
1.89	38.6	CH/CH_3
1.87 + 1.18	33.8	CH_2
1.70 + 1.30	31.2	CH_2
1.66 + 1.14	51.2	CH_2
1.62	19.3	CH/CH_3
0.92	35.1	CH/CH_3
0.89	18.1	CH/CH_3
0.85	28.2	CH/CH_3

δ (^{1}H)/ppm	δ (^{13}C)/ppm	HSQC phase
	129.7	C_q
	129.4	C_q
	77.2	C_{OH}
	35.2	C_q

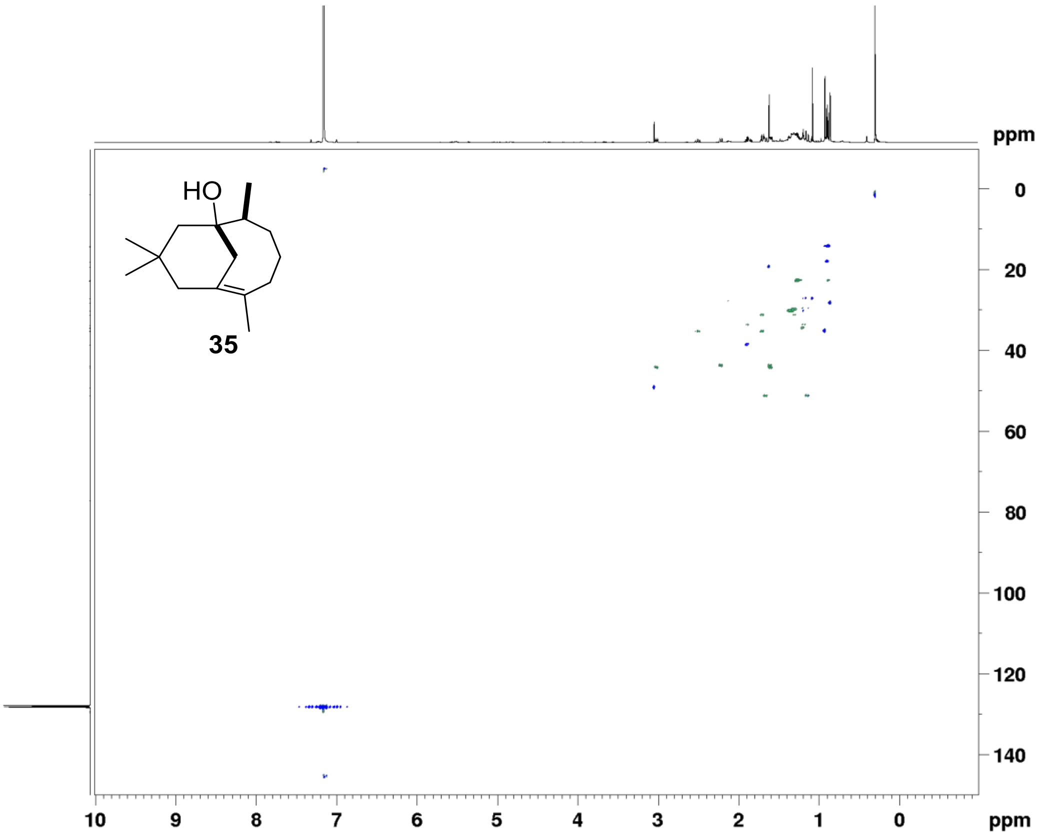

Figure S131. ^{1}H-^{13}C HSQC NMR spectrum of compound **35** in C_6D_6 (pos. phase = blue (CH/CH_3), neg. phase = green (CH_2)).

Table S48. ^{1}H NMR signals and the corresponding ^{1}H-^{1}H COSY correlations for compound **35**. Signals with weak intensities are given in parentheses.

δ (^{1}H)/ppm	COSY correlations
3.02 + 1.60	(2.21, 1.66)
2.51 + 1.70	1.30
2.21 + 1.61	(3.02)
1.89	0.89
1.87 + 1.18	1.70 + 1.30
1.70 + 1.30	2.51 + 1.70, 1.87 + 1.18
1.66 + 1.14	(3.02)
1.62	-
0.92	-
0.89	1.89
0.85	-

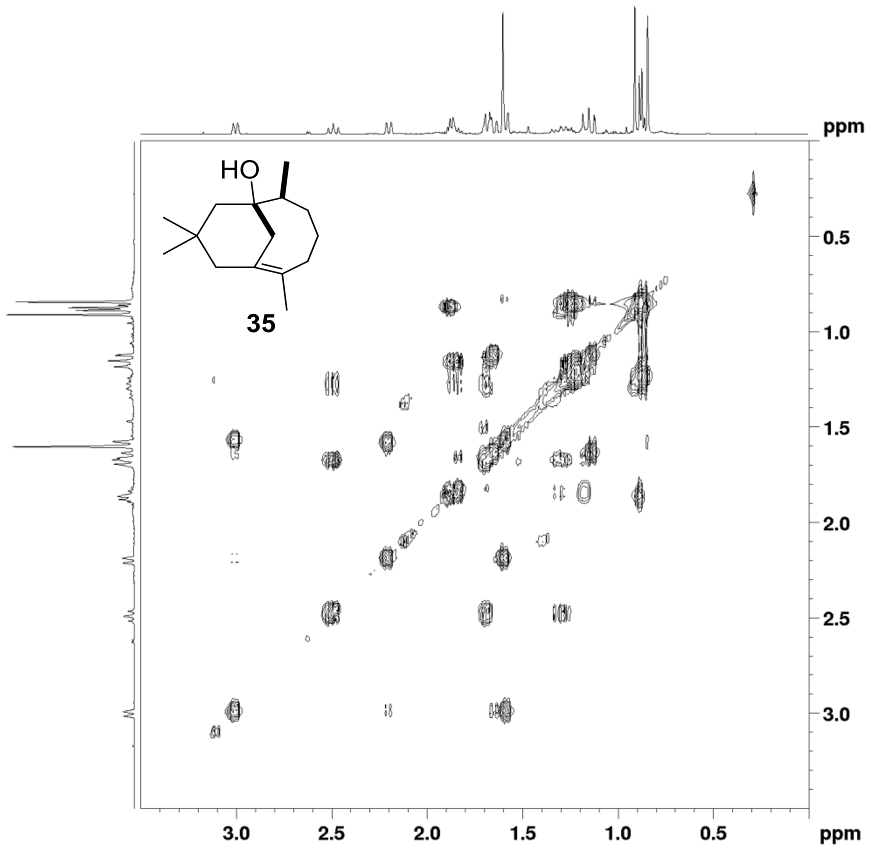

Figure S132. ^{1}H-^{1}H COSY NMR spectrum of **35** in C_6D_6.

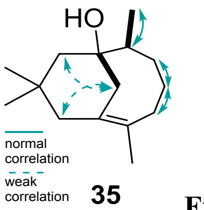

35

Figure S133. Key ^{1}H-^{1}H COSY NMR correlations of **35** as indicated by green arrows. Dashed arrows refer to observed correlations considered weaker than others.

Table S49. Selected correlations between ^{13}C NMR signals and neighbouring ^{1}H NMR signals as collected from the ^{1}H-^{13}C HMBC spectrum of compound **35**. Weak signals are given in parentheses.

δ (^{1}H)/ppm	δ (^{13}C)/ppm
3.02 + 1.60	129.7 and/or 129.4, 77.2, 51.2, 43.8
2.51 + 1.70	129.7 and/or 129.4, 33.8, 31.2, 19.3, (38.6)
2.21 + 1.61	129.7 and/or 129.4, 51.2, 44.3, 35.3 and/or 35.2, 28.1
1.89	77.2, 33.8, 31.2, 18.1
1.87 + 1.18	77.2, 38.6, 31.2
1.70 + 1.30	Hard to distinguish
1.66 + 1.14	77.2, 38.6, 35.3 and/or 35.2, 28.2, (44.3)
1.62	129.7 and/or 129.4, 35.3
0.92	51.2, 43.8, 35.3 and/or 35.2, 28.2
0.89	77.2, 38.6, 33.8
0.85	51.2, 43.8, 35.3 and/or 35.2

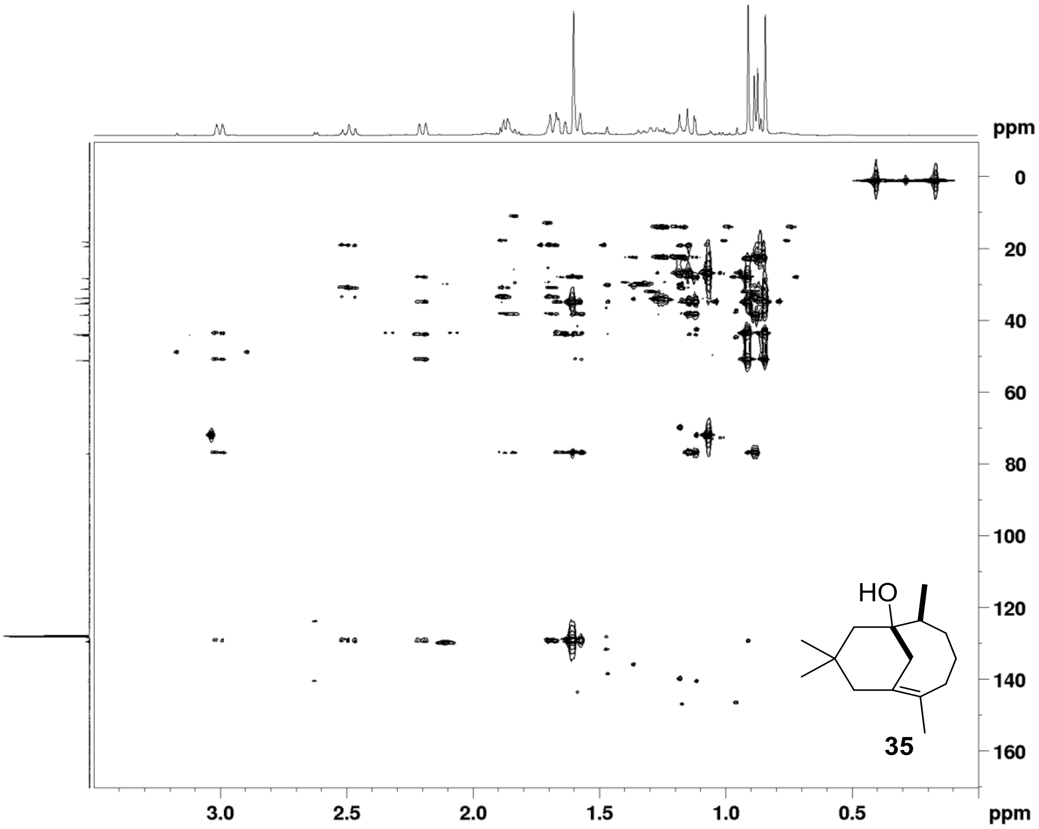

Figure S134. ^{1}H-^{13}C HMBC NMR spectrum of compound **35** in C_6D_6.

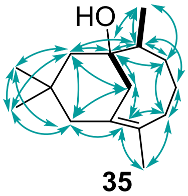

35

Figure S135. Key ^{1}H-^{13}C HMBC NMR correlations of **35** as indicated by arrows.

Elucidation of relative stereochemistry of **35** is based on ^{1}H-^{1}H-NOESY analysis. Important correlations are observed for 3.00 ppm. Correlations between 3.00 ppm and 1.87 ppm, as well as 2.51 ppm indicate that this proton is directed toward the larger ring. This also explains the low field shift of this proton, as its orientation is in the same level as the double bond, which leads to the observed low field shift. Another important correlation is observed between 1.89 ppm and 0.89 ppm which can be explained if both groups are on the opposide side of the CH_2 group. This also fits the observed correlation between 1.62 ppm (Me group) and 2.21 ppm.

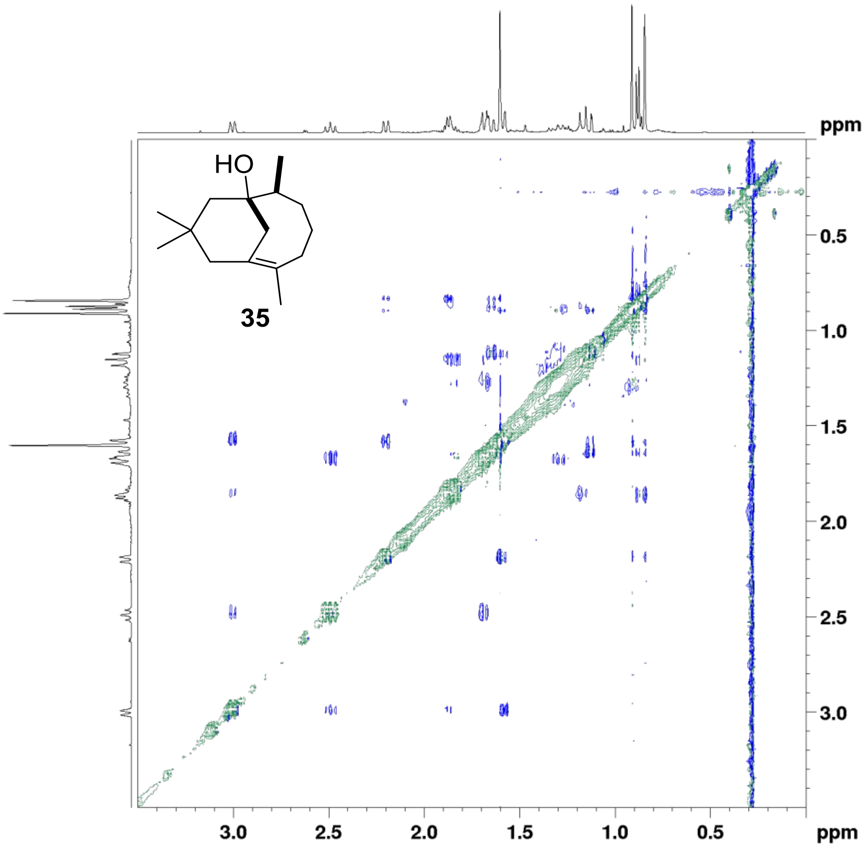

Figure S136. ^{1}H-^{1}H NOESY NMR spectrum of compound **35** in C_6D_6.

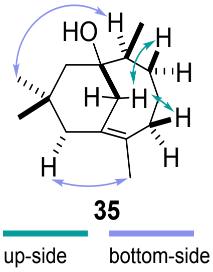

Figure S137. Key ^{1}H-^{1}H NOESY NMR correlations of **35** as indicated by arrows.

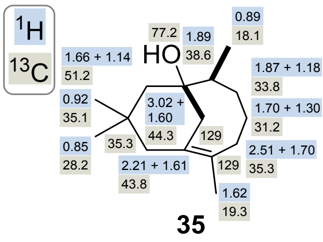

Figure S138. Full assignment of chemical shifts for compound **35**.

1.5.18 Structure elucidation of 36

To simplify structure elucidation only the centre of each HSQC signal will be used as the chemical shift, despite different multiplicities, since overlapping signals can be differentiated more easily. **36** and its structure elucidation was performed as an additional set of signals to elucidate the non-oxidized structure of **35**.

Table S50. ^{1}H NMR signals and their corresponding ^{13}C NMR signals for compound **36** as analysed with the support of ^{1}H-^{13}C HSQC experiments. The quaternary carbon atoms are listed at the bottom.

δ (^{1}H)/ppm	δ (^{13}C)/ppm	HSQC phase
2.07 + 1.54	43.9	CH_2
1.87 + 1.59	39.0	CH_2
1.67 + 1.00	49.8	CH_2
1.67	39.8	CH/CH_3
1.61 + 1.36	44.0	CH_2
1.55 + 1.10	32.6	CH_2
1.47 + 1.12	26.9	CH_2
1.18	18.6	CH/CH_3
0.92	17.9	CH/CH_3
0.84	28.8	CH/CH_3
0.83	36.1	CH/CH_3
	74.9	C_q
	64.9	C_q
	64.5	C_q
	31.5	C_q

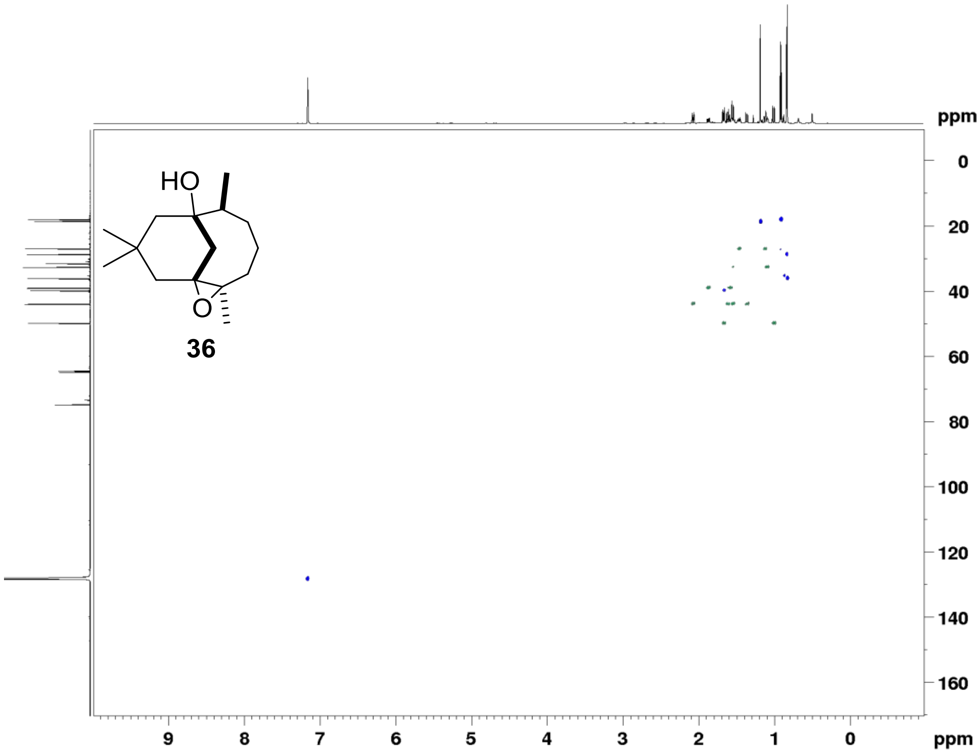

Figure S139. ^{1}H-^{13}C HSQC NMR spectrum of compound **36** in C_6D_6 (pos. phase = blue (CH/CH_3), neg. phase = green (CH_2)).

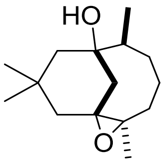

36

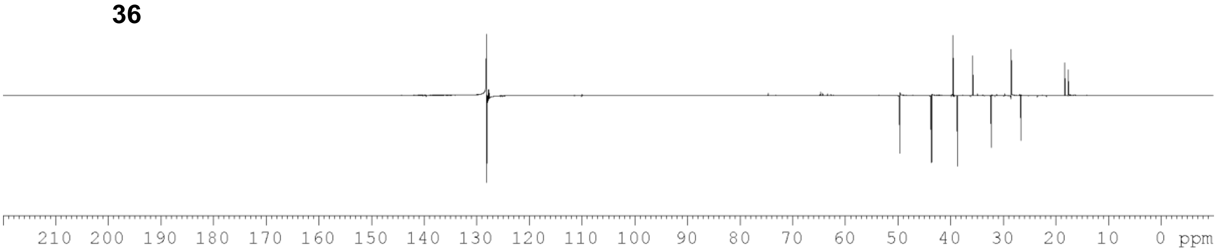

Figure S140. $^{13}C\{^{1}H\}$ DEPT135 NMR spectrum of compound **36** in C_6D_6.

Table S51. ^{1}H NMR signals and the corresponding ^{1}H-^{1}H COSY correlations for compound **36**. Signals with weak intensities are given in parentheses.

δ (^{1}H)/ppm	COSY correlations
2.07 + 1.54	(1.67, 1.36)
1.87 + 1.59	1.47
1.67 + 1.00	2.07, 0.84
1.67	0.92
1.61 + 1.36	2.07, 0.84
1.55 + 1.10	1.67, 1.47, (1.87)
1.47 + 1.12	1.87 + 1.59
1.18	1.59
0.92	1.67
0.84	1.61, 1.00
0.83	(1.36)

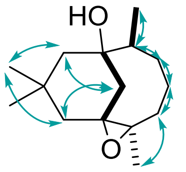

36

Figure S141. Key ^{1}H-^{1}H COSY NMR correlations of **36** as indicated by green arrows.

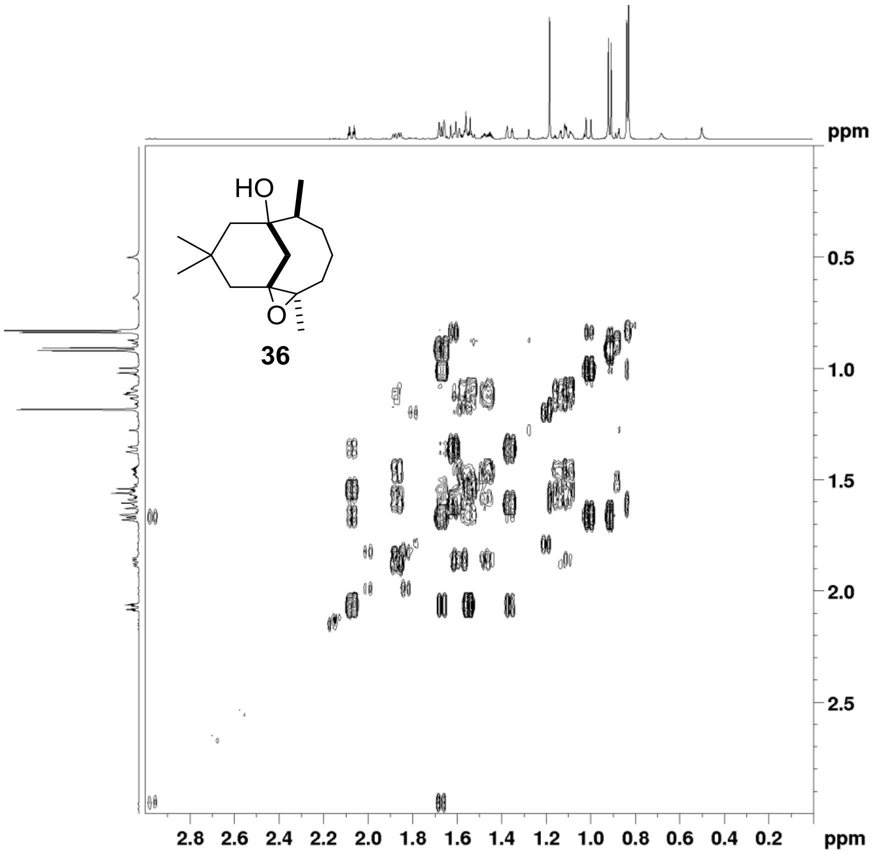

Figure S142. ^{1}H-^{1}H COSY NMR spectrum of **36** in C_6D_6.

Table S52. Selected correlations between ^{13}C NMR signals and neighbouring ^{1}H NMR signals as collected from the ^{1}H-^{13}C HMBC spectrum of compound **36**. Weak signals are given in parentheses.

δ (^{1}H)/ppm	δ (^{13}C)/ppm
2.07 + 1.54	74.9, 64.5 and/or 64.9, 49.8, 44.0
1.87 + 1.59	64.5 and/or 64.9, 32.6, 26.9, 18.6
1.67 + 1.00	74.9, 44.0 and/or 43.9, 39.8, 36.1, 31.5, 28.8
1.67	26.9, 17.9, (32.6)
1.61 + 1.36	64.5 and/or 64.9, 49.8, 43.9, 36.1, 31.6, 28.8,
1.55 + 1.10	Hard to distinguish
1.47 + 1.12	39.8, 39.0, 32.6 (64.5 and/or 64.9)
1.18	64.5 and/or 64.9, 39.0
0.92	74.9, 39.8, 32.6
0.84	49.8, 44.0, 36.1, 31.5
0.83	49.8, 44.0, 31.5, 28.8

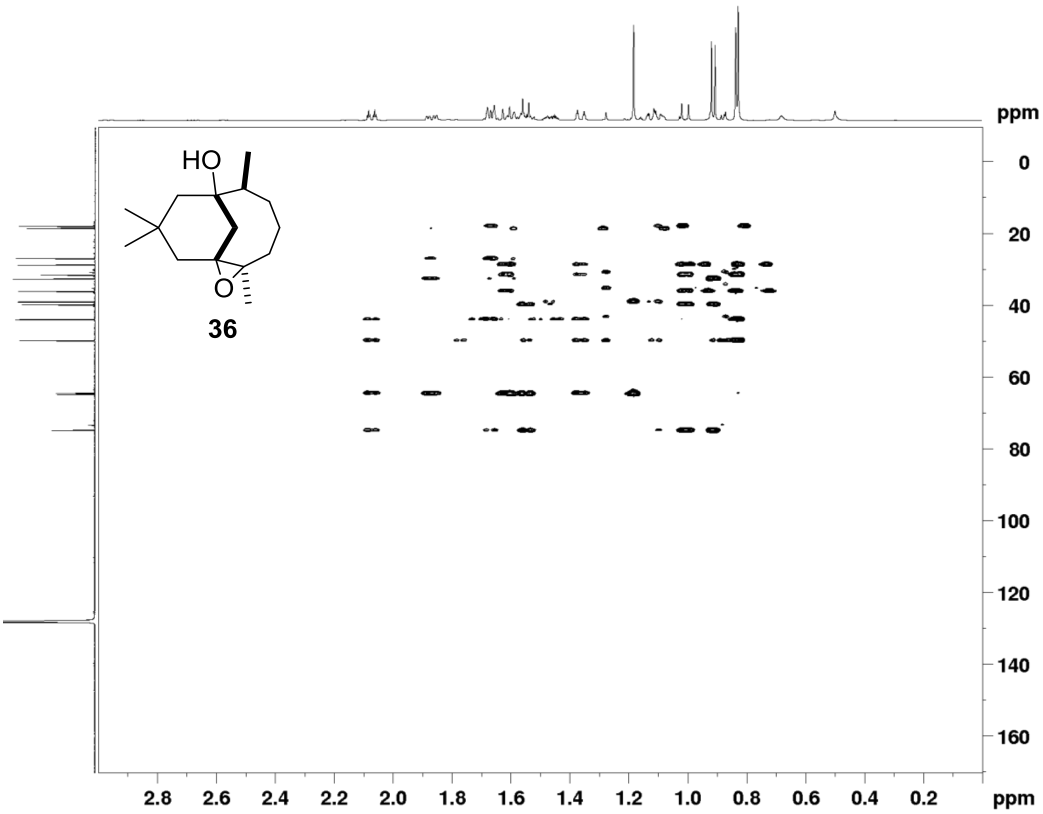

Figure S143. ^{1}H-^{13}C HMBC NMR spectrum of compound **36** in C_6D_6.

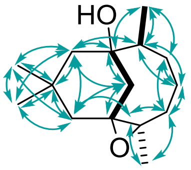

Figure S144. Key ^{1}H-^{13}C HMBC NMR correlations of **36** as indicated by green arrows.

Elucidation of relative stereochemistry is elucidated by ^{1}H-^{1}H NOESY analysis. Some key correlations will be discussed here. The correlation between 2.07 ppm and 1.5 ppm area fits to the previously observed correlations in **35**. As no other correlation for 2.07 ppm shows similar intensity, likely 1.57 ppm and 1.59 ppm are both part of this broad correlation signal. The methyl group at 1.18 ppm has a correlation towards 0.84 ppm, 1.36 ppm and 1.87 ppm. The methyl group at 0.84 ppm has correlations towards 1.67 ppm, 1.36 ppm and 1.18 ppm. These indicate the bottom-side of the molecule similar to the relative orientation observed in **35**. With respect to **35** in which the bottom-side is sterically hindered, the epoxide sitting on the up-side, with the methyl group (1.18 ppm) on the bottom-side could be explained, as we observe one favoured diastereomer using the non-chiral *m*-CPBA for epoxidation.

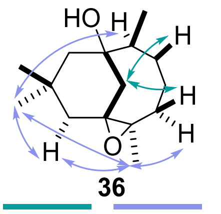

Figure S145. Key ^{1}H-^{1}H NOESY NMR correlations of **36** as indicated by arrows.

Additionally, 1,1-ADEQUATE NMR was performed to strengthen the CH_2 bridge as analysed. Also a differentiation of the ^{13}C signals of the epoxide was made possible, with 65.9 being next to the methyl group.

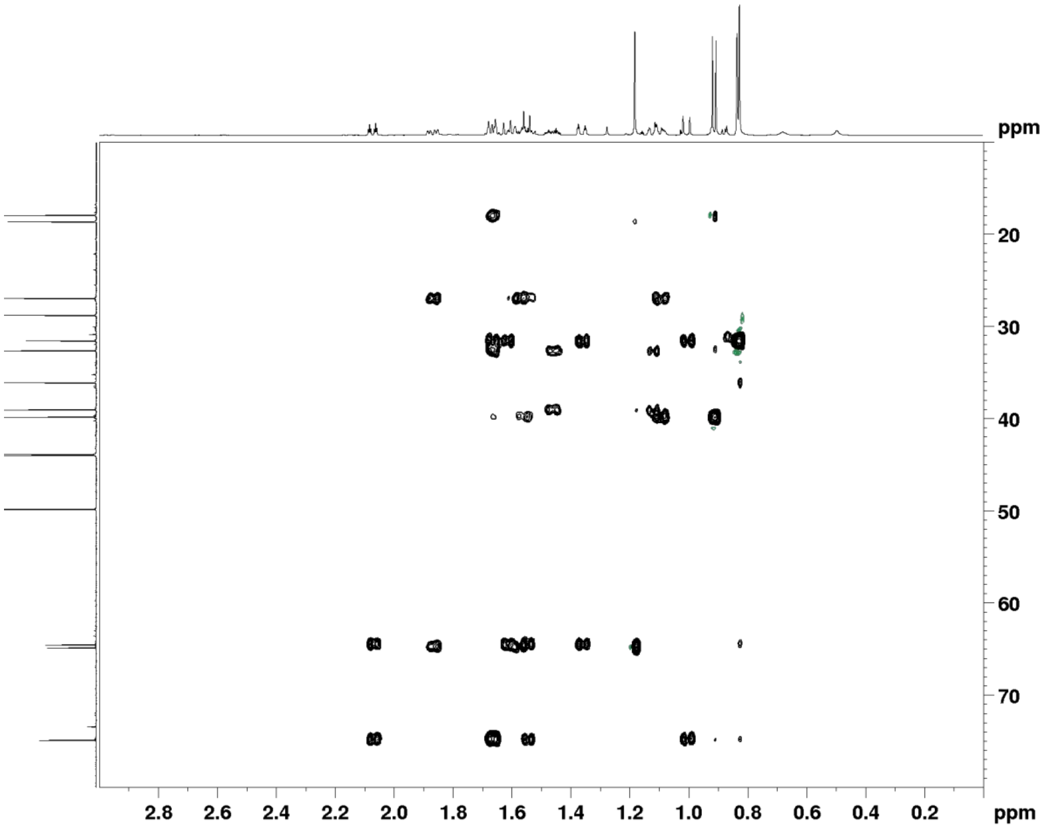

Figure S146. ^1H-^{13}C ADEQUATE NMR spectrum of compound **36** in C_6D_6.

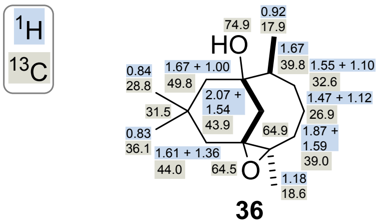

36

Figure S147. Full assignment of chemical shifts for compound **36**.

1.5.19 Structure elucidation of 40

To simplify structure elucidation only the centre of each HSQC signal will be used as the chemical shift, despite different multiplicities, since overlapping signals can be differentiated more easily.

Table S53. ^1H NMR signals and their corresponding ^{13}C NMR signals for compound **40** as analysed with the support of ^1H-^{13}C HSQC and $^{13}C\{^1H\}$ DEPT135 experiments. The quaternary carbon atoms are listed at the bottom.

δ (^1H)/ppm	δ (^{13}C)/ppm	DEPT135/HSQC phase
5.25	129.0	CH/CH_3
5.24	133.1	CH/CH_3
5.13	129.8	CH/CH_3

δ (^{1}H)/ppm	δ (^{13}C)/ppm	DEPT135/HSQC phase
4.79 + 4.75	109.2	CH_2
2.40 + 2.20	35.0	CH_2
2.35 + 2.12	40.0	CH_2
2.29 + 2.18	35.5	CH_2
2.28 + 2.18	42.1	CH_2
2.08	45.5	CH/CH_3
1.64	20.9	CH/CH_3
1.51	17.1	CH/CH_3
1.33 + 1.17	20.8	CH/CH3
	151.1	C_q
	132.2	C_q

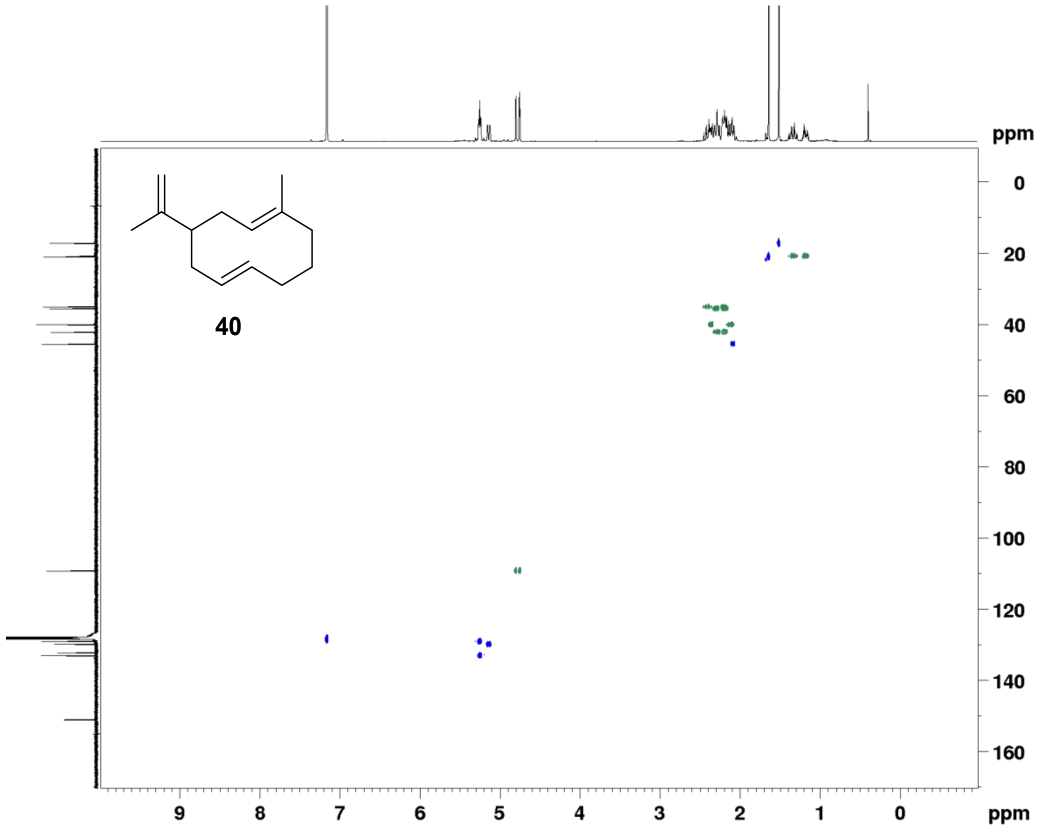

Figure S148. ^{1}H-^{13}C HSQC NMR spectrum of compound **40** in C_6D_6 (pos. phase = blue (CH/CH_3), neg. phase = green (CH_2)).

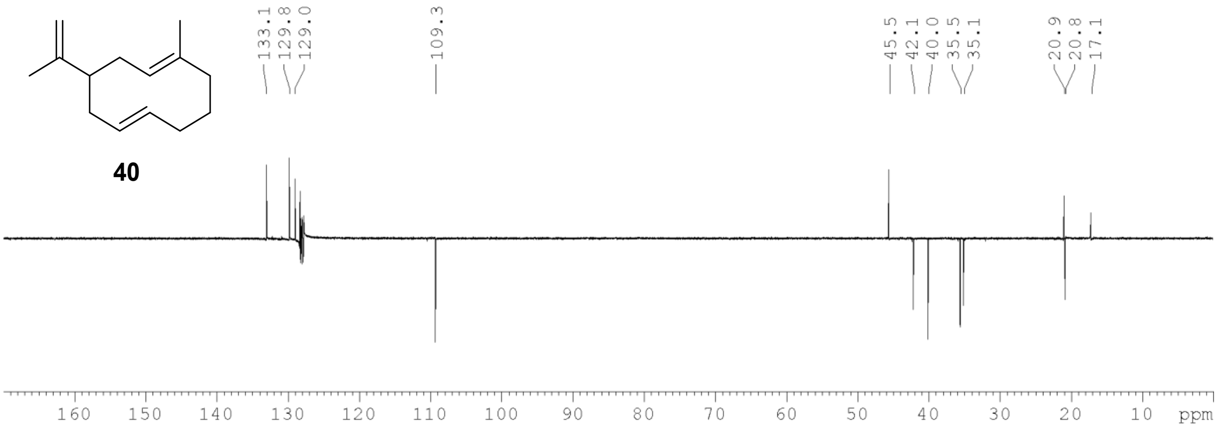

Figure S149. $^{13}C\{^1H\}$ DEPT135 NMR spectrum of compound **40** in C_6D_6.

Table S54. ^1H NMR signals and the corresponding ^1H-^1H COSY correlations for compound **40**. Signals with weak intensities are given in parentheses.

δ (^1H)/ppm	COSY correlations
5.25	2.29/2.28 + 2.18
5.24	2.35 + 2.12
5.13	2.40 + 2.20, 1.51
4.79 + 4.75	1.64
2.40 + 2.20	5.13, 2.08
2.35 + 2.12	5.24
2.29 + 2.18	Hard to distinguish
2.28 + 2.18	Hard to distinguish
2.08	2.40
1.64	4.75
1.51	5.13, 2.20
1.33 + 1.17	2.29/2.28 + 2.18

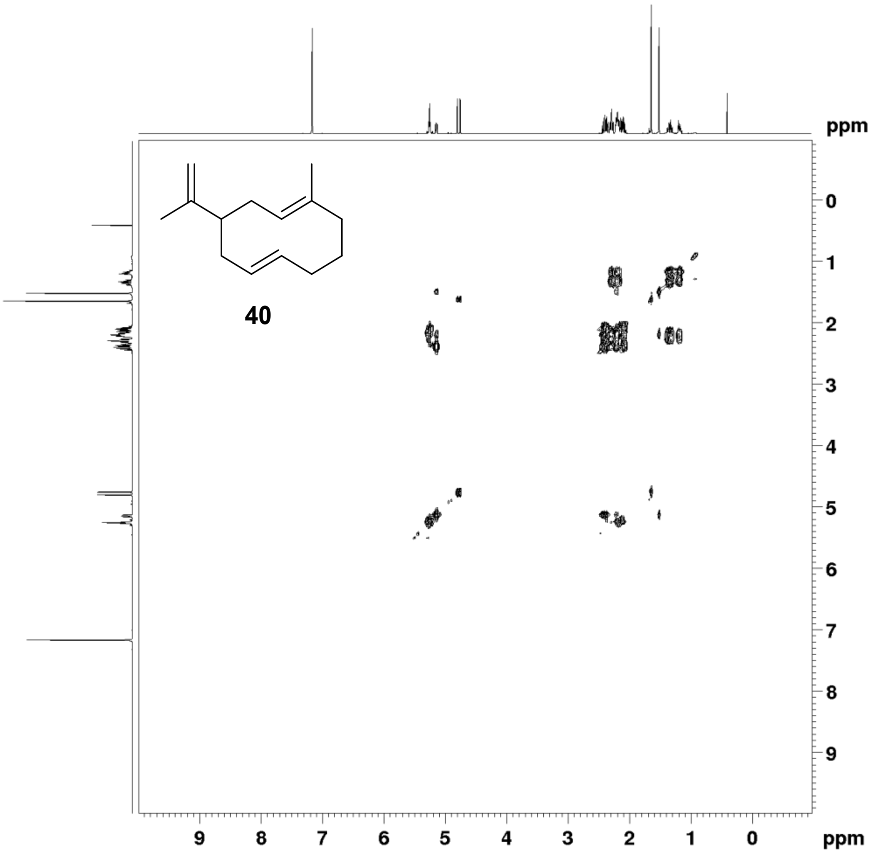

Figure S150. ^{1}H-^{1}H COSY NMR spectrum of **40** in C_6D_6.

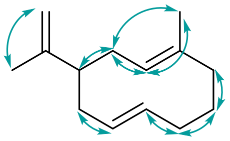

40

Figure S151. Key ^{1}H-^{1}H COSY NMR correlations of **40** as indicated by arrows.

Table S55. Selected correlations between ^{13}C NMR signals and neighbouring ^{1}H NMR signals as collected from the ^{1}H-^{13}C HMBC spectrum of compound **40**. Weak signals are given in parentheses.

δ (^{1}H)/ppm	δ (^{13}C)/ppm
5.25	40.0
5.24	35.5
5.13	45.5, 42.1, 35.0, 17.1
4.79 + 4.75	151.1, 45.5, 20.9
2.40 + 2.20	132.3, 129.8, 45.5, 40.0
2.35 + 2.12	133.1, 129.0, 45.5, 35.0
2.29 + 2.18	Hard to distinguish
2.28 + 2.18	Hard to distinguish
2.08	151.1, 109.2, 40.0, 20.9

δ (^{1}H)/ppm	δ (^{13}C)/ppm
1.64	151.1, 109.2, 45.5
1.51	132.3, 129.8, 42.1
1.33 + 1.17	132.3, 129.0, 42.1, 35.5

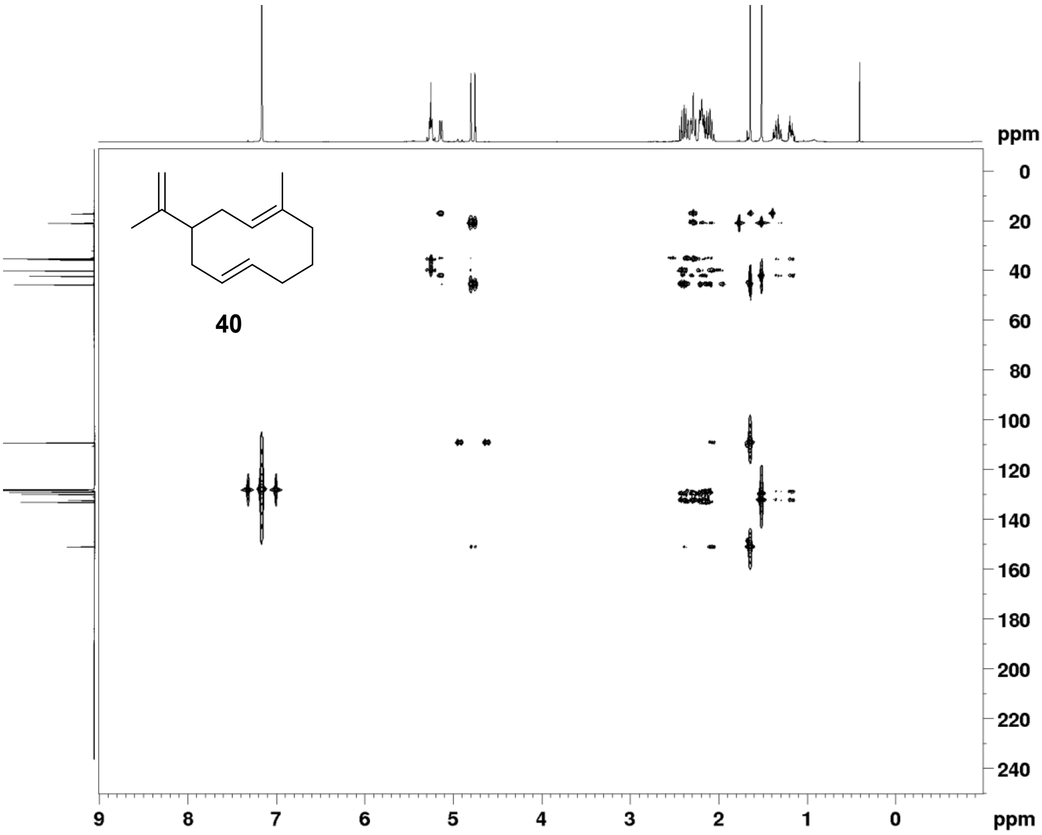

Figure S152. ^{1}H-^{13}C HMBC NMR spectrum of compound **40** in C_6D_6.

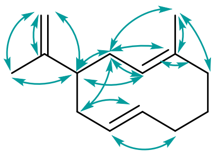

40

Figure S153. Key ^{1}H-^{13}C HMBC NMR correlations of **40** as indicated by arrows.

^{1}H-^{1}H-NOESY analysis revealed that the olefinic protons at 5.24 ppm, 5.13 ppm are on the same side as the CH group at 2.08 ppm, while the Methyl group (1.51 ppm) and 5.25 ppm are orientated on the side of the macrocycle. The (*E*)-configuration of the trisubstituted double bond is determined by a strong correlation between 1.51 ppm and 2.40 ppm.

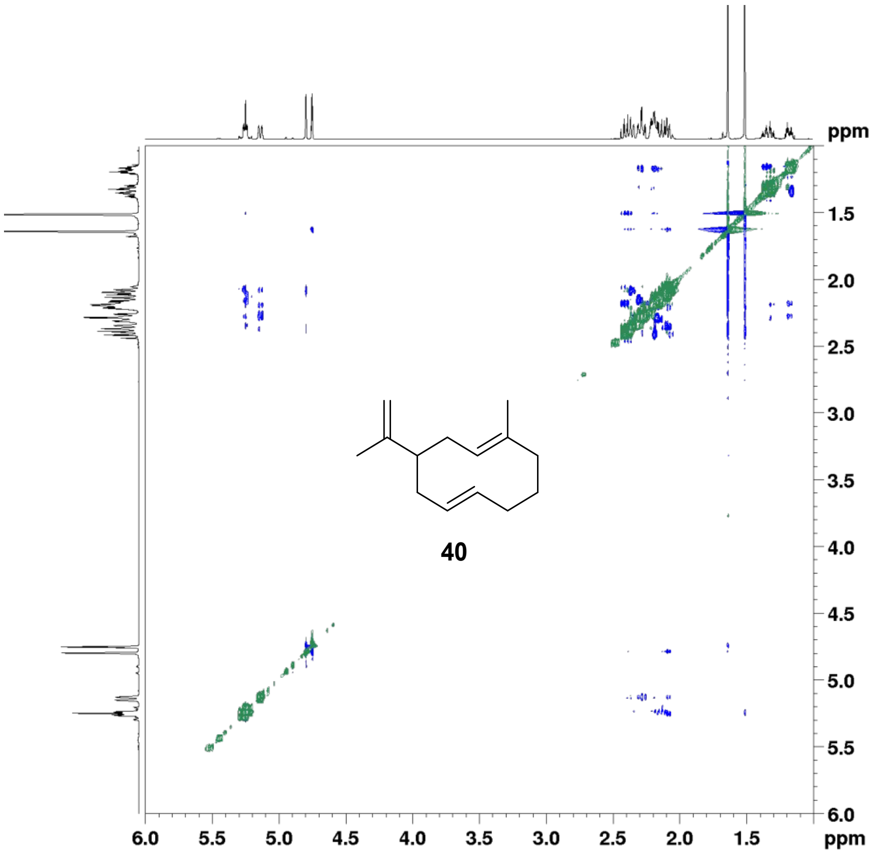

Figure S154. ^{1}H-^{1}H NOESY NMR spectrum of compound **40** in C_6D_6.

Figure S155. Key ^{1}H-^{1}H NOESY NMR correlations of **40** as indicated by arrows.

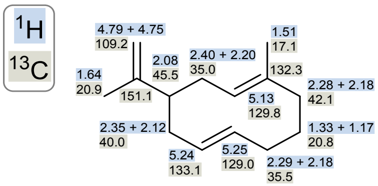

40

Figure S156. Full assignment of chemical shifts for compound **40**.

1.5.20 Structure elucidation of 41

To simplify structure elucidation only the centre of each HSQC signal will be used as the chemical shift, despite different multiplicities, since overlapping signals can be differentiated more easily.

Table S56. ^{1}H NMR signals and their corresponding ^{13}C NMR signals for compound **41** as analysed with the support of ^{1}H-^{13}C HSQC and $^{13}C\{^{1}H\}$ DEPT135 experiments. The quaternary carbon atoms are listed at the bottom.

δ (^{1}H)/ppm	δ (^{13}C)/ppm	DEPT135/HSQC phase
6.10	142.6	CH/CH_3
5.59	128.5	CH/CH_3
5.50	139.6	CH/CH_3
5.10 + 5.00	113.4	CH_2

δ (^{1}H)/ppm	δ (^{13}C)/ppm	DEPT135/HSQC phase
1.73	50.7	CH/CH_3
1.66 + 1.21	26.5	CH_2
1.66 + 1.18	39.6	CH_2
1.51 + 1.42	22.5	CH_2
1.47 + 1.38	29.4	CH_2
1.19	30.3	CH/CH_3
1.19	30.3	CH/CH_3
1.01	28.2	CH/CH_3
	70.3	C_q
	39.5	C_q

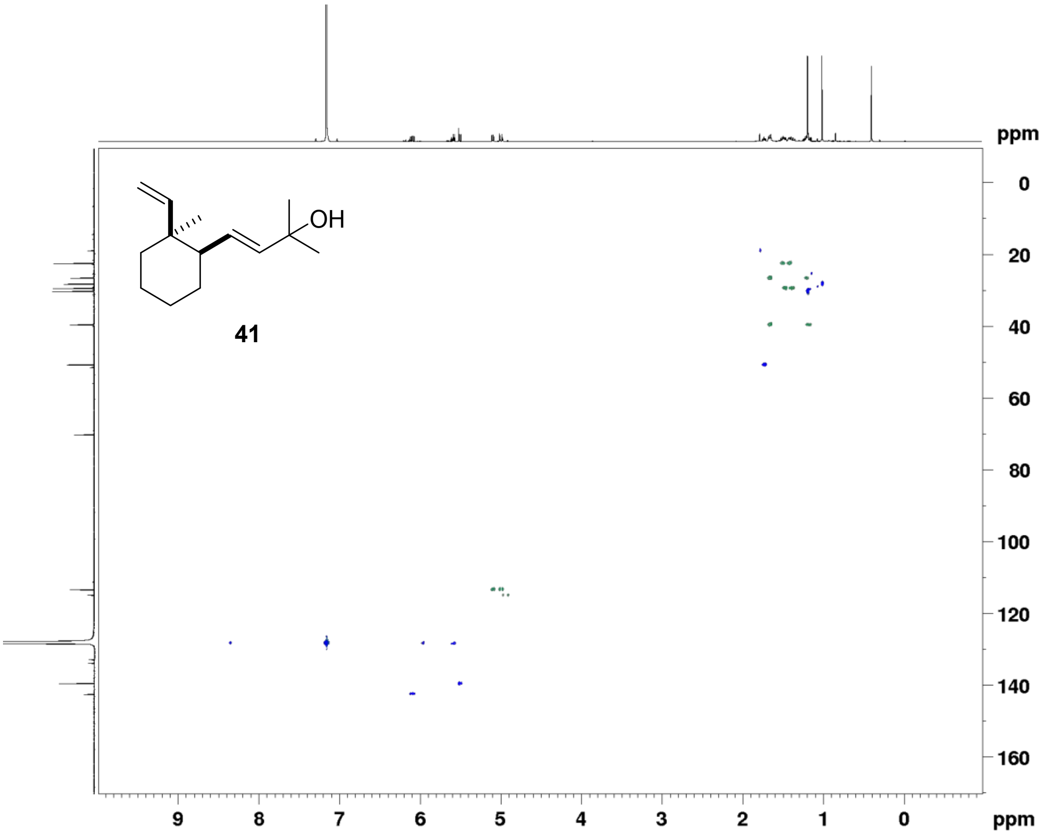

Figure S157. ^{1}H-^{13}C HSQC NMR spectrum of compound **41** in C_6D_6 (pos. phase = blue (CH/CH_3), neg. phase = green (CH_2)).

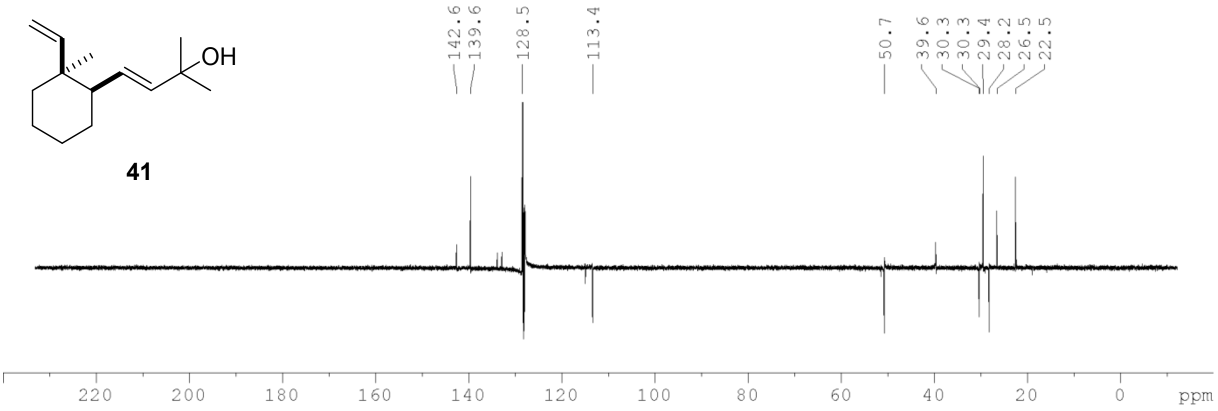

Figure S158. $^{13}C\{^{1}H\}$ DEPT135 NMR spectrum of compound **41** in C_6D_6.

^{1}H-^{1}H COSY NMR was performed two times. The first measurement was done for the entire spectra, while a second measurement was done selectively for the aliphatic region to obtain better resolution. However, COSY analysis in this region stays challenging.

Table S57. ^{1}H NMR signals and the corresponding ^{1}H-^{1}H COSY correlations for compound **41**. Signals with weak intensities are given in parentheses.

δ (^{1}H)/ppm	COSY correlations
6.10	5.10 + 5.00
5.59	5.50, 1.73
5.50	5.59
5.10 + 5.00	6.10
1.73	5.59, 1.47 + 1.38
1.66 + 1.21	1.51 + 1.42, 1.47 + 1.38
1.66 + 1.18	1.51 + 1.42
1.51 + 1.42	1.66 + 1.21, 1.66 + 1.18
1.47 + 1.38	1.73, 1.66 + 1.21
1.19	-
1.19	-
1.01	(5.00, 1.66, 1.47)

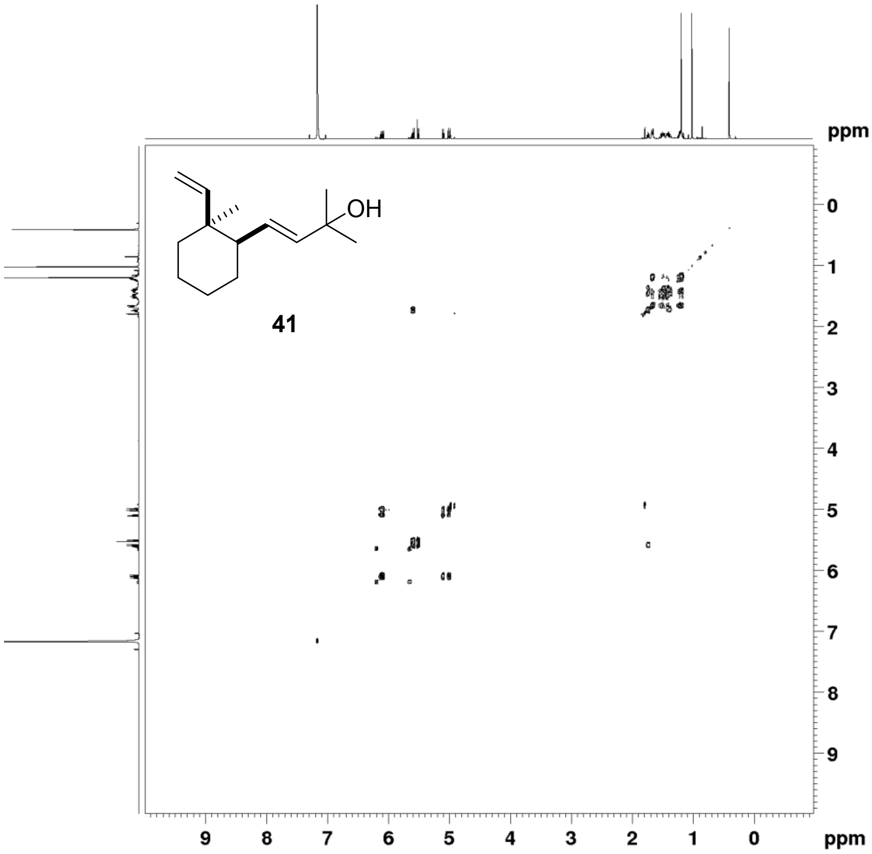

Figure S159. ^1H-^1H COSY NMR spectrum of **41** in C_6D_6.

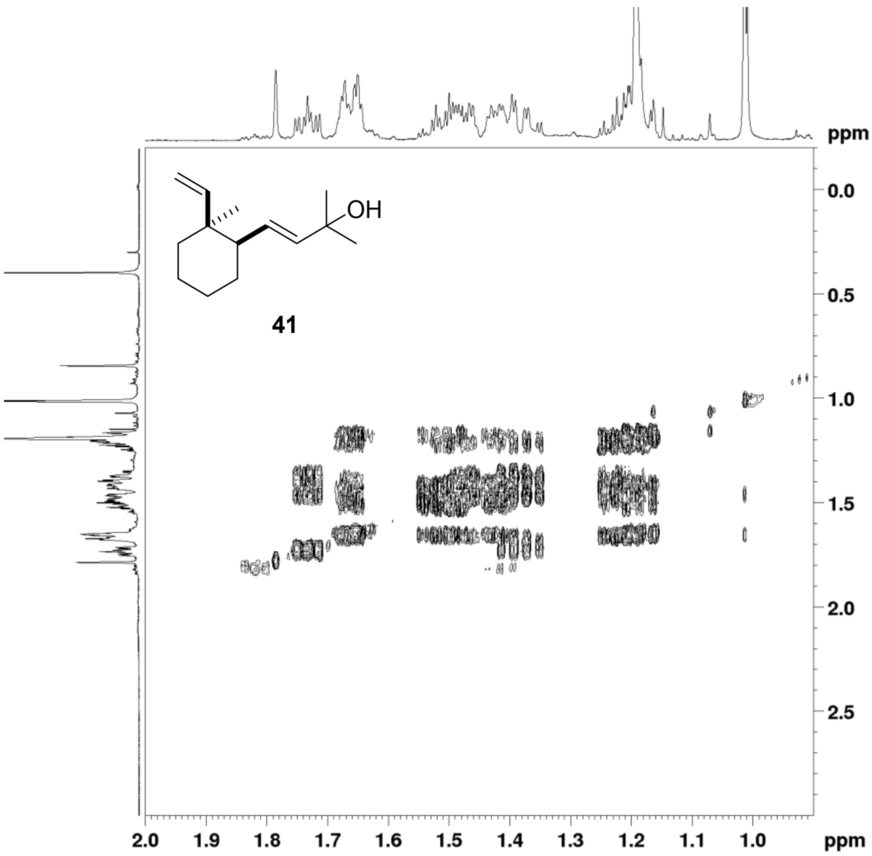

Figure S160. Zoom-In of second measurement of ^1H-^1H COSY NMR spectrum of **41** in C_6D_6 for aliphatic region.

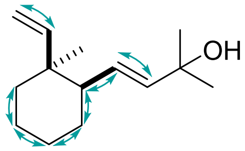

41

Figure S161. Key ^{1}H-^{1}H COSY NMR correlations of **41** as indicated by arrows.

^{1}H-^{13}C HMBC NMR was performed two times. The first measurement was done for the entire spectra, while a second measurement was done selectively for the aliphatic region to obtain better resolution.

Table S58. Selected correlations between ^{13}C NMR signals and neighbouring ^{1}H NMR signals as collected from the ^{1}H-^{13}C HMBC spectrum of compound **41**. Weak signals are given in parentheses. Signals that can't be distinguished are marked with an x.

δ (^{1}H)/ppm	δ (^{13}C)/ppm
6.10	39.X, 28.2
5.59	139.6, 70.3, 50.7, 29.4
5.50	128.5, 70.3, 50.7, 30.3
5.10 + 5.00	142.6, 39.X
1.73	142.6, 139.6, 128.5, 39.X, 29.4, 28.2, 26.5
1.66 + 1.21	Hard to distinguish
1.66 + 1.18	Hard to distinguish
1.51 + 1.42	39.x, 29.4
1.47 + 1.38	128.5, 50.7, 39.X, 26.5, 22.5
1.19	139.6, 70.3, 30.3
1.01	142.6, 50.7, 39.X

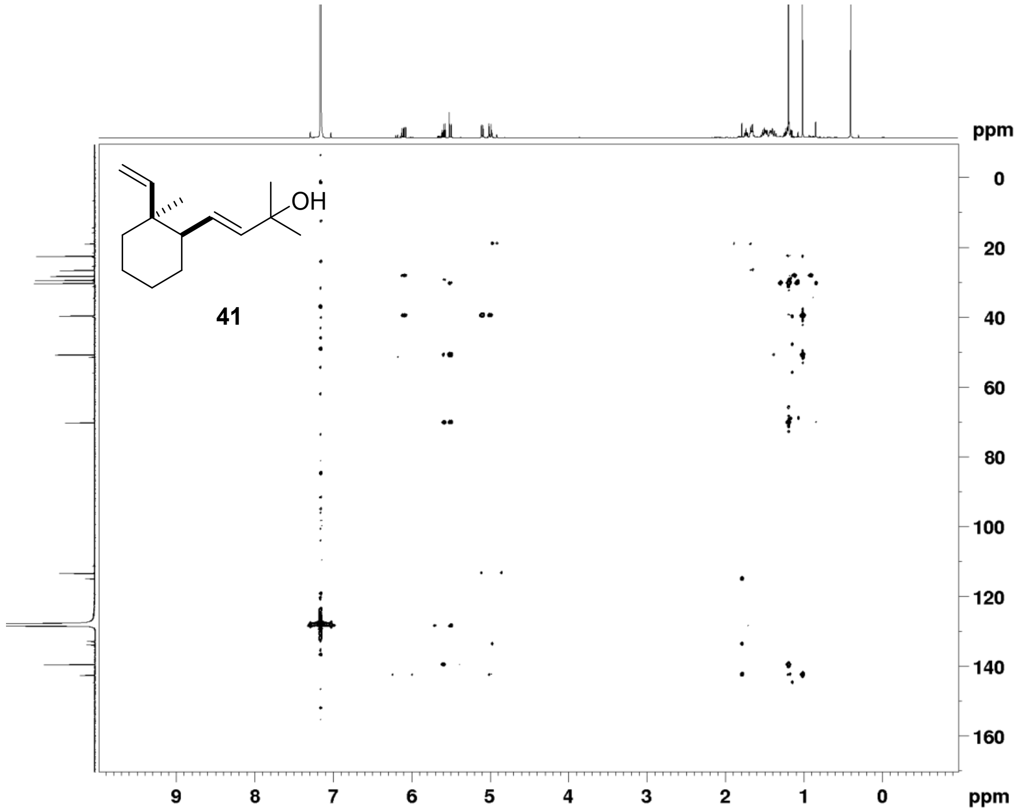

Figure S162. ^{1}H-^{13}C HMBC NMR spectrum of compound **41** in C_6D_6.

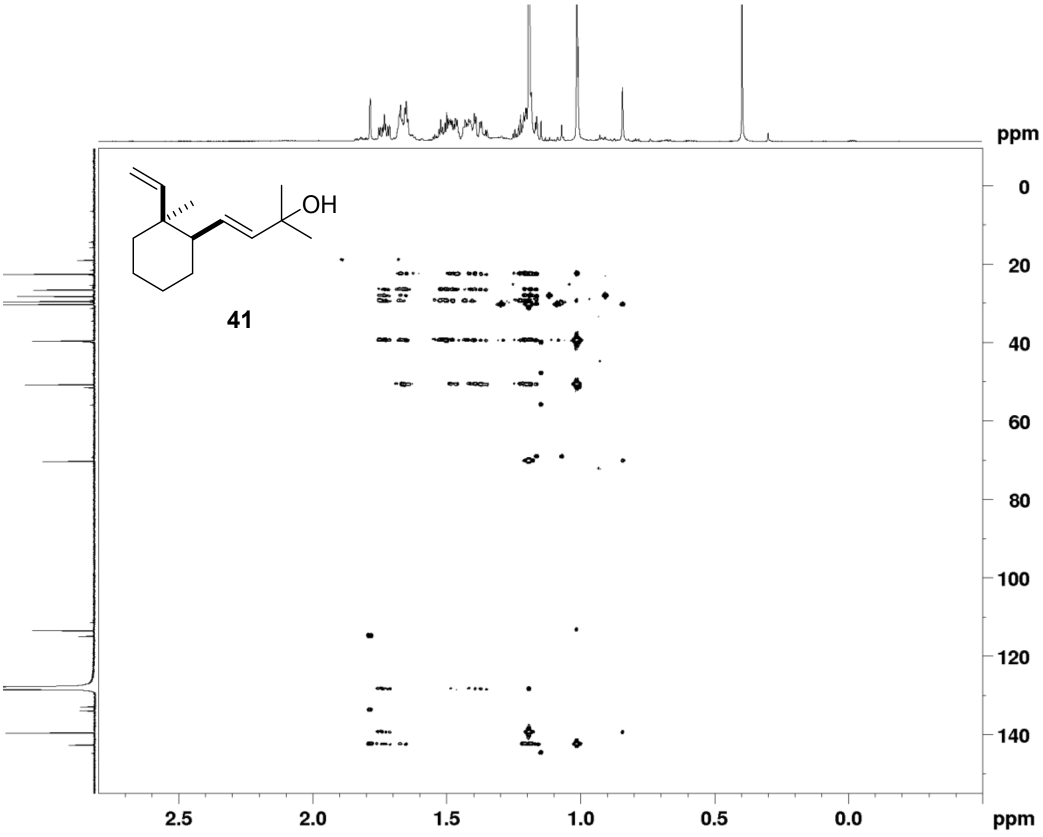

Figure S163. Zoom-In of second ^{1}H-^{13}C HMBC NMR measurement of compound **41** in C_6D_6 for aliphatic region.

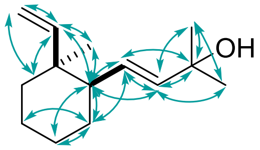

41

Figure S164. Key ^{1}H-^{13}C HMBC NMR correlations of **41** as indicated by arrows.

^{1}H-^{1}H-NOESY analysis shows only a dispersive signal (due to the strong ^{3}J coupling of 15.5 Hz, characteristic for *trans* configuration) between the olefinic protons at 5.59 ppm and 5.50 ppm confirming an (*E*)-configured double bond. ^{3}J-coupling of 1.73 indicates an axial position with ^{3}J = 11.8 Hz, 8.4 Hz and 3.5 Hz. ^{1}H-^{1}H NOESY correlation signal of 1.73 ppm and 1.01 ppm can be explained by an equatorial position of 1.01 ppm. Also 6.10 ppm shows correlation signals to 1.39 ppm and 1.50 ppm which can be explained by 1,3-diaxial relationships.

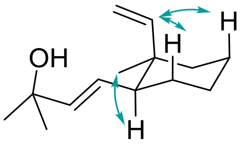

41

Figure S165. Key ^{1}H-^{1}H NOESY NMR correlations of **41** as indicated by arrows.

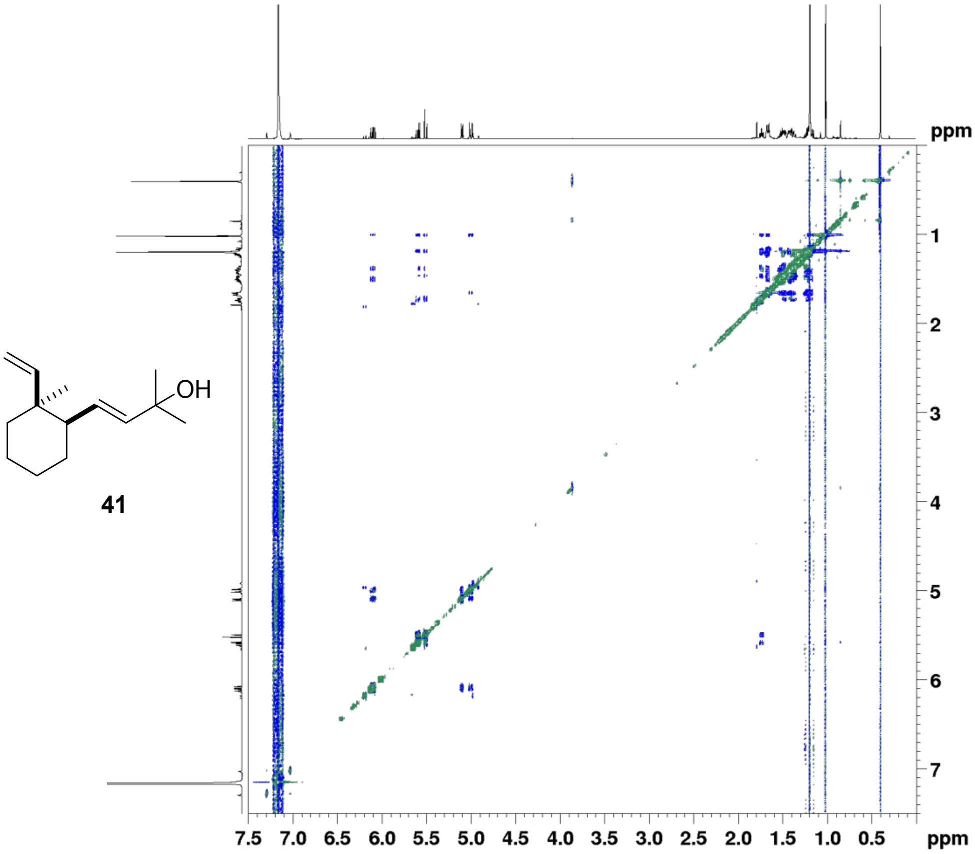

Figure S166. ^{1}H-^{1}H NOESY NMR spectrum of compound **41** in C_6D_6.

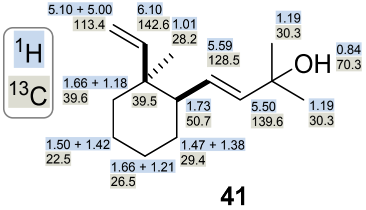

41

Figure S167. Full assignment of chemical shifts for compound **41**.

1.6 Presentation of AlphaFold model of Omp7

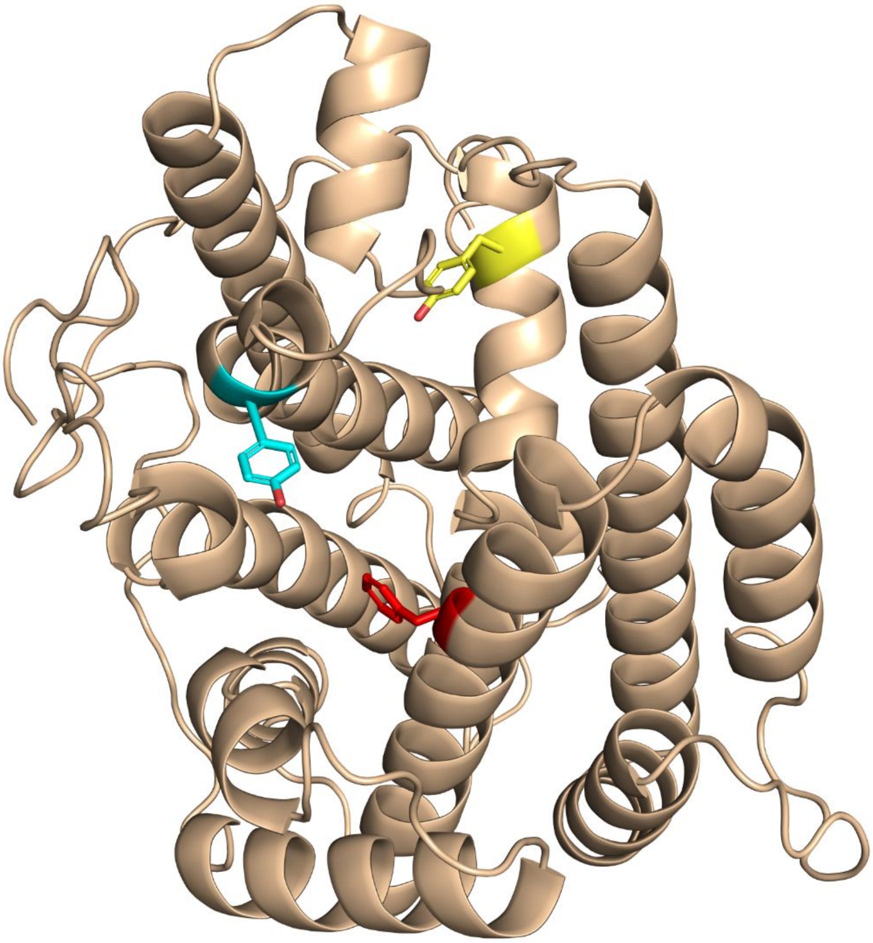

Figure S168. AlphaFold model of Omp7 with respective mutation sites marked: F81W (red), Y172F (yellow), Y227F (cyan).[36b]

1.7 X-Ray Data

Experimental: A colourless needle-shaped crystal with dimensions 0.69×0.09×0.08 mm^3 was mounted on a 18 mm mounted CryoLoop (20 micron, 0.2 - 0.3 mm, Hampton Research). Data were collected using an XtaLAB AFC12 (RINC): Kappa single diffractometer. The crystal was kept at a steady T = 100.00(10) K during data collection.

Data were measured using ω scans of 0.5° per frame for 2.9/11.5 s using Cu K_α radiation. The diffraction pattern was indexed and the total number of runs and images was based on the strategy calculation from the program CrysAlisPro (Rigaku, V1.171.43.95a, 2023) The maximum resolution that was achieved was = 79.066° (0.79 Å).

The diffraction pattern was indexed and the total number of runs and images was based on the strategy calculation from the program CrysAlisPro (Rigaku, V1.171.43.95a, 2023) and the unit cell was refined using CrysAlisPro (Rigaku, V1.171.43.95a, 2023) on 8870 reflections, 69% of the observed reflections.

Crystal Data. $C_{14}H_{22}O$, Mr = 206.31, monoclinic, $P2_1$ (No. 4), a = 6.84480(10) Å, b = 10.6082(2) Å, c = 8.81470(10) Å, β = 102.302(2)°, $\alpha = \gamma$ = 90°, V = 625.348(17) Å^3, T = 100(10) K, Z = 2, Z' = 1, m(Cu K_a) = 0.504, 12862 reflections measured, 2641 unique (R_{int} = 0.0673) which were used in all calculations. The final wR_2 was 0.1157 (all data) and R_1 was 0.0425 (I > 2(I)).

Data reduction, scaling and absorption corrections were performed using CrysAlisPro (Rigaku, V1.171.43.95a, 2023). The final completeness is 99.90 % out to 79.066° in Θ. A multi-scan absorption correction was performed using CrysAlisPro 1.171.43.95a (Rigaku Oxford Diffraction, 2023) using spherical harmonics,implemented in SCALE3 ABSPACK scaling algorithm. The absorption coefficient μ of this material is 0.504 mm^{-1} at this wavelength (λ = 1.542 Å) and the minimum and maximum transmissions are 0.316 and 1.000.

The structure was solved and the space group $P2_1$ (# 4) determined by the ShelXT(Sheldrick, 2015) structure solution program using Intrinsic Phasing and refined by Least Squares using version 2019/3 of ShelXL 2019/3 (Sheldrick, 2015). All non-hydrogen atoms were refined anisotropically. Hydrogen atom positions were calculated geometrically and refined using the riding model.

Absolute structure determination: Flack parameter: 0.1 (2), Hooft parameter: 0.17 (13).

CSD 2450081

Summary of Data - Deposition Number 2450081

Compound Name:
Data Block Name: data_cs031
Unit Cell Parameters: a 6.84480(10) b 10.6082(2) c 8.81470(10)P21)

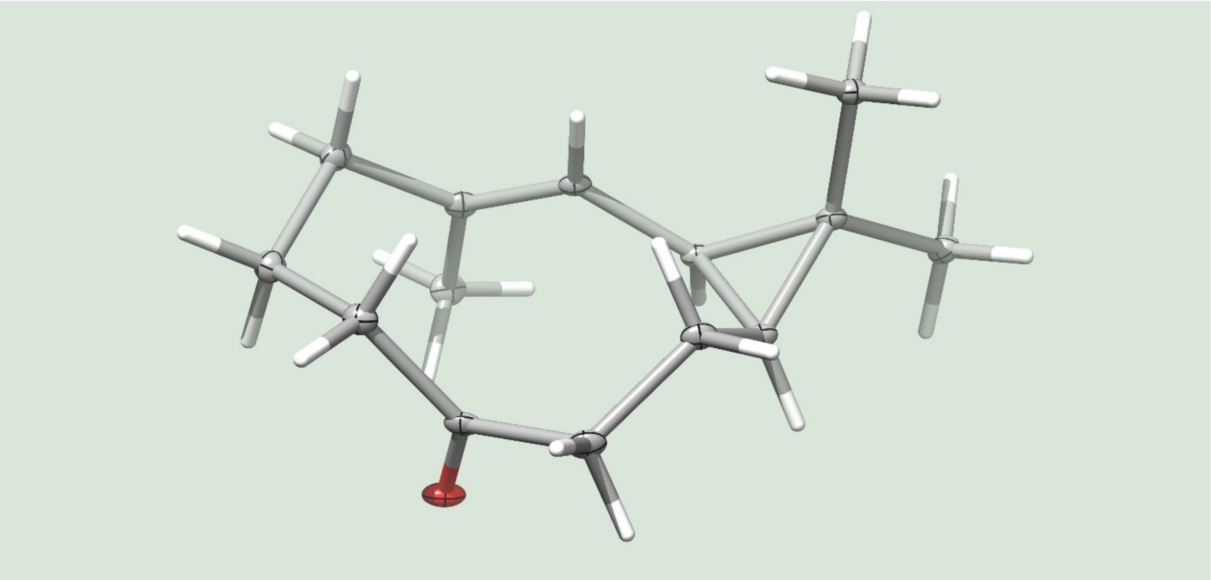

2. Copies of NMR Spectra

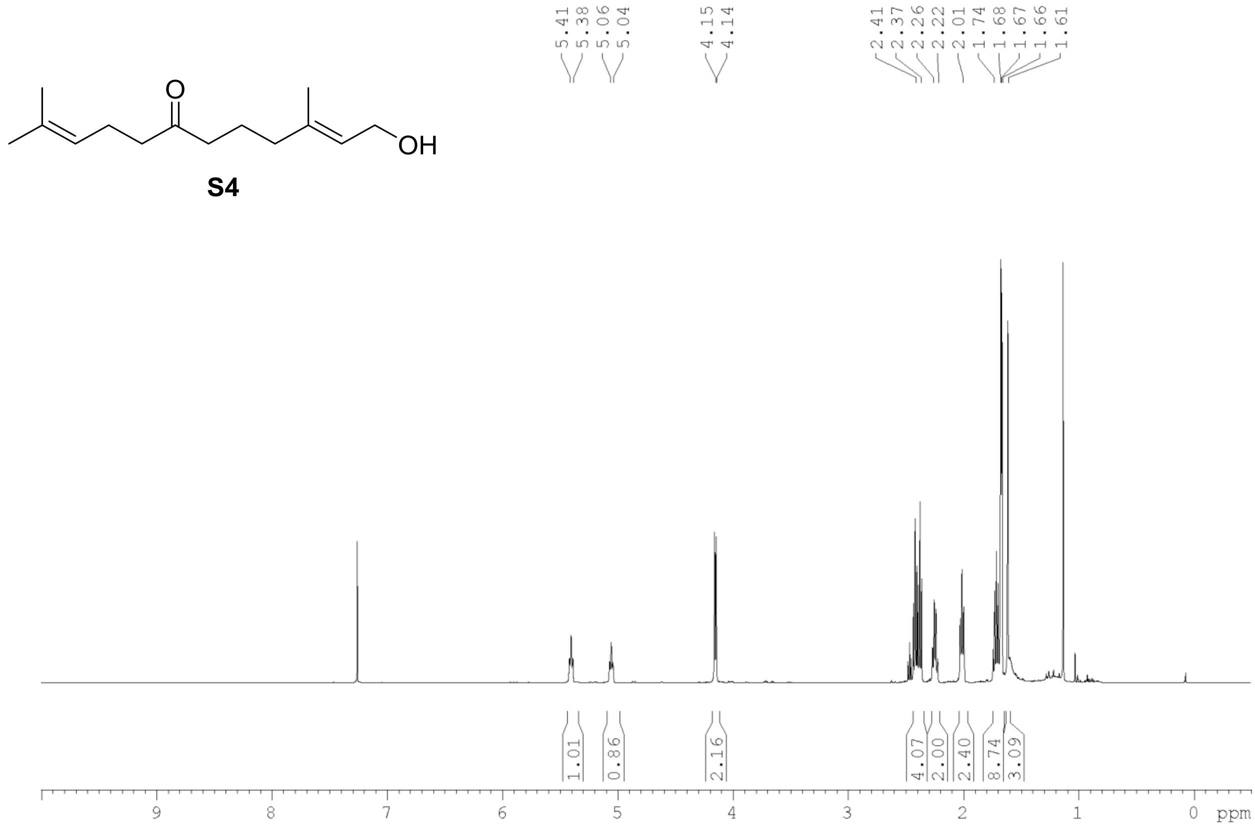

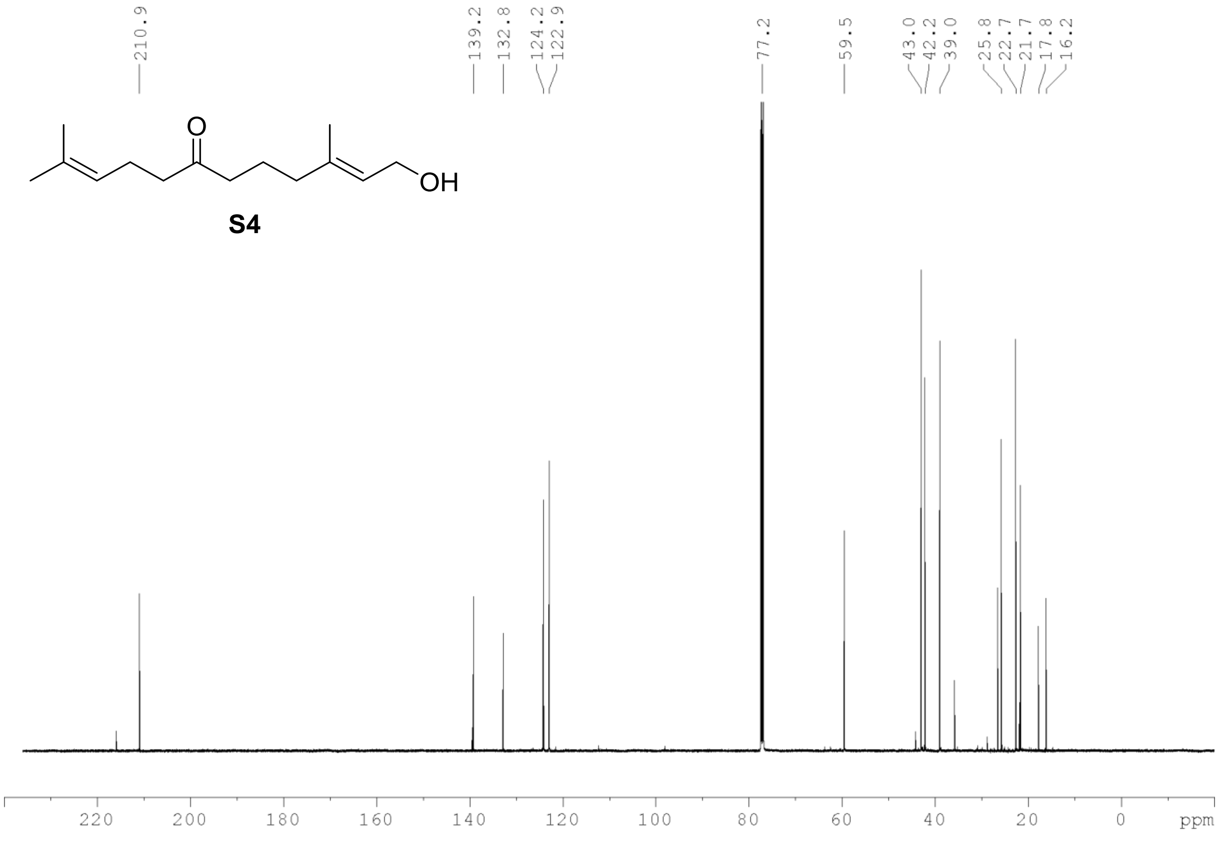

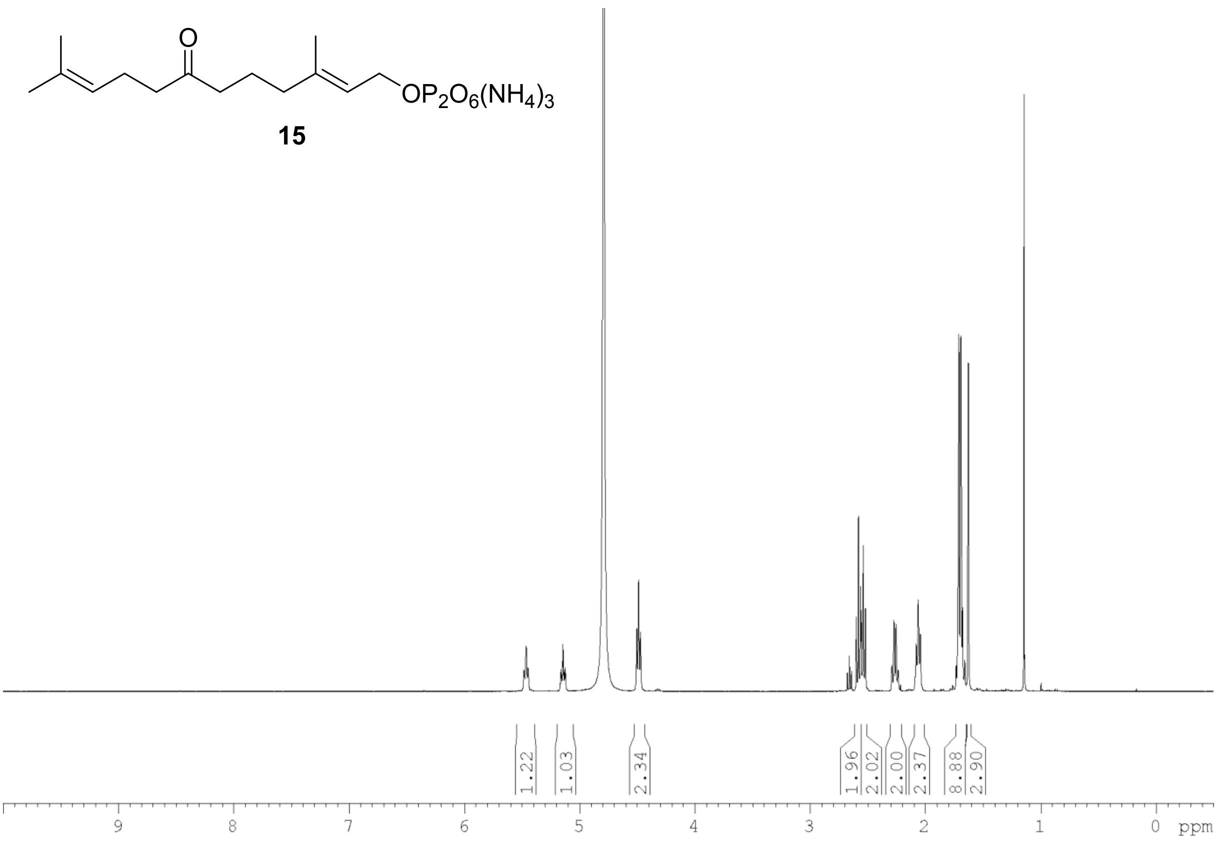
O
OP2O6(NH4)3
15
1.22
1.03
2.34
1.96
2.02
2.00
2.37
8.88
2.90
9
8
7
6
5
4
3
2
1
0 ppm

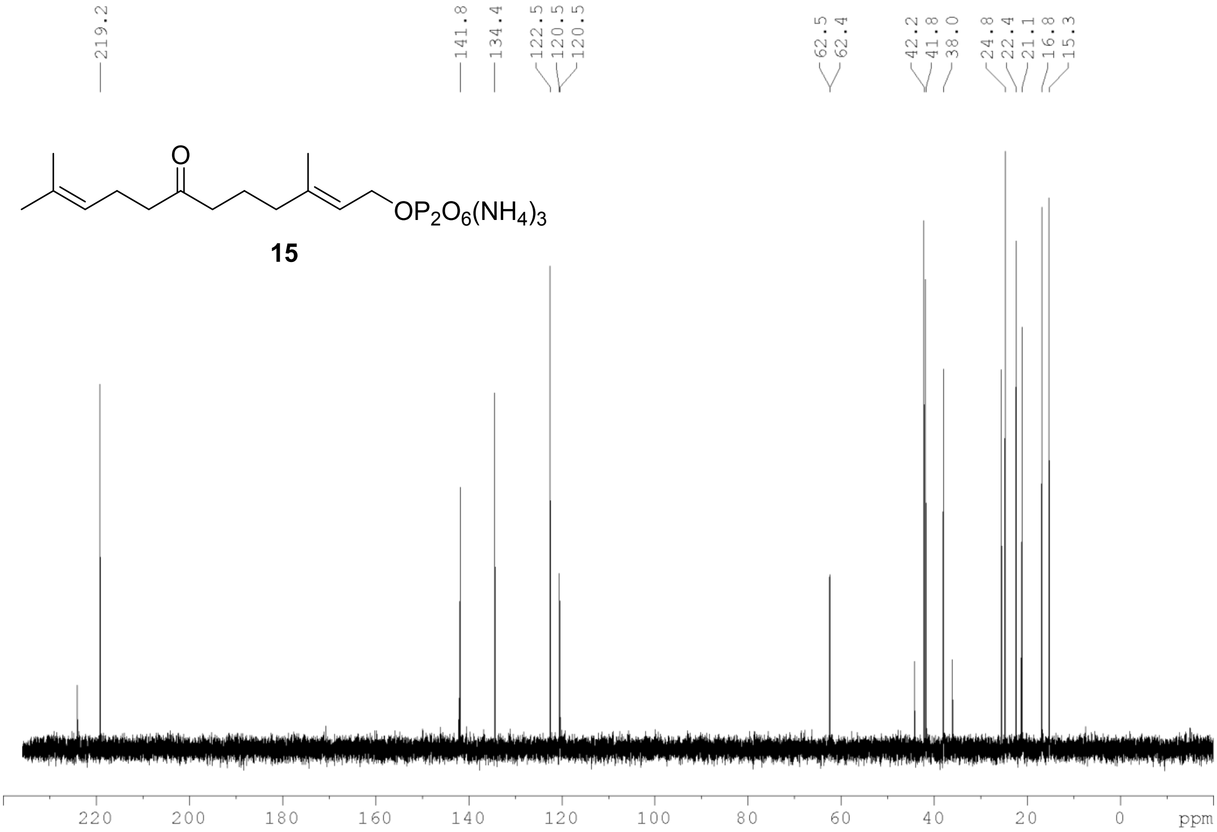
219.2
141.8
134.4
122.5
120.5
120.5
62.5
62.4
42.2
41.8
38.0
24.8
22.4
21.1
16.8
15.3
O
OP2O6(NH4)3
15
220
200
180
160
140
120
100
80
60
40
20
0
ppm

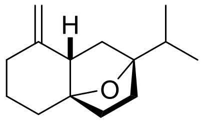

28b

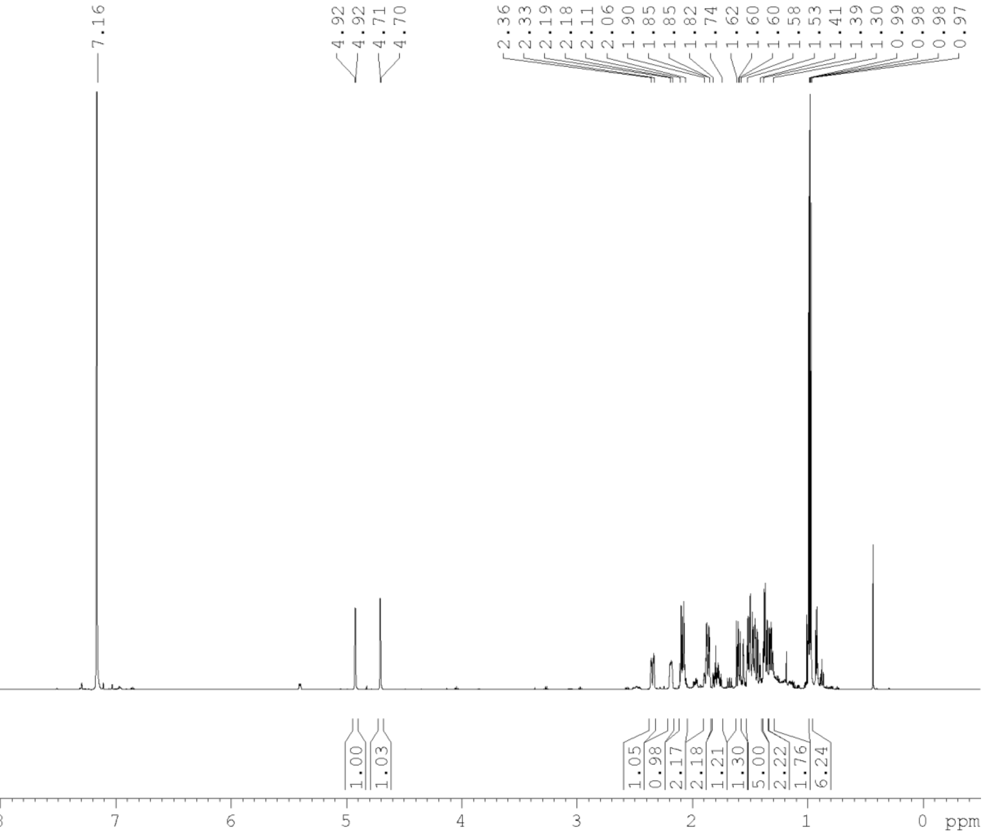
7.16
4.92
4.92
4.71
4.70
2.36
2.33
2.19
2.18
2.11
2.06
1.90
1.85
1.85
1.82
1.74
1.62
1.60
1.60
1.58
1.53
1.41
1.39
1.30
0.99
0.98
0.98
0.97
1.00
1.03
1.05
0.98
2.17
2.18
1.21
1.30
5.00
2.22
1.76
6.24
9 8 7 6 5 4 3 2 1 0 ppm

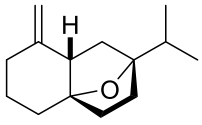

28b

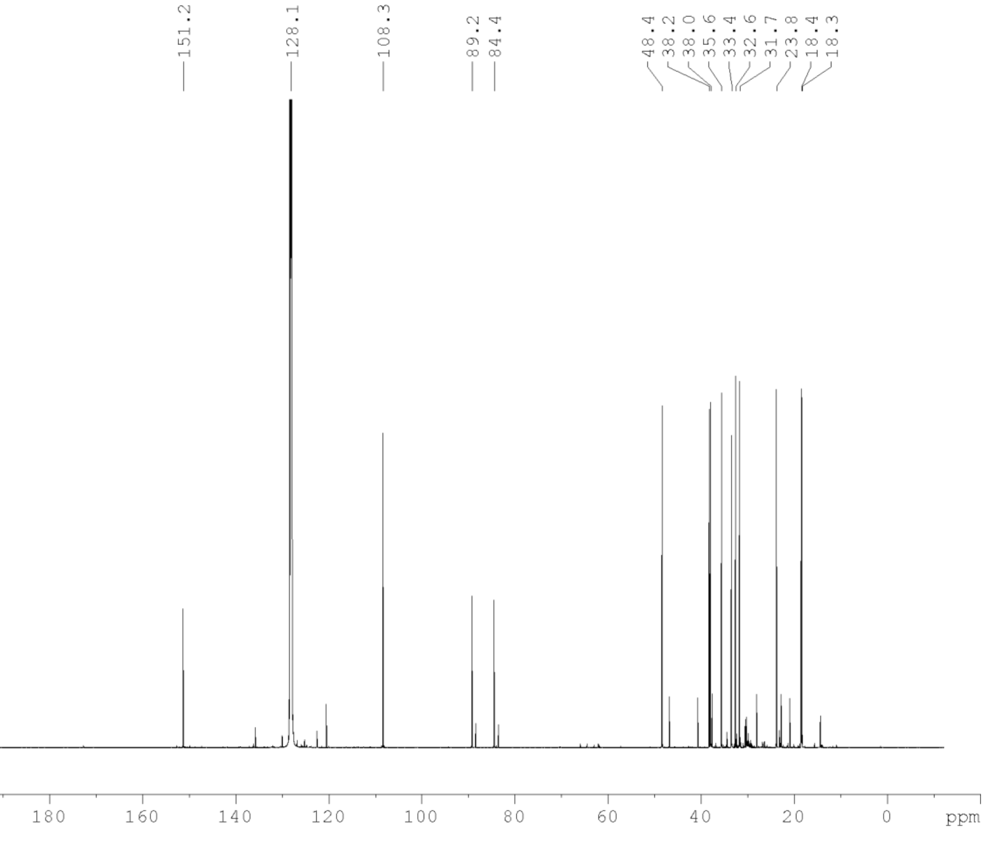
151.2
128.1
108.3
89.2
84.4
48.4
38.2
38.0
35.6
33.4
32.6
31.7
23.8
18.4
18.3
220 200 180 160 140 120 100 80 60 40 20 0 ppm

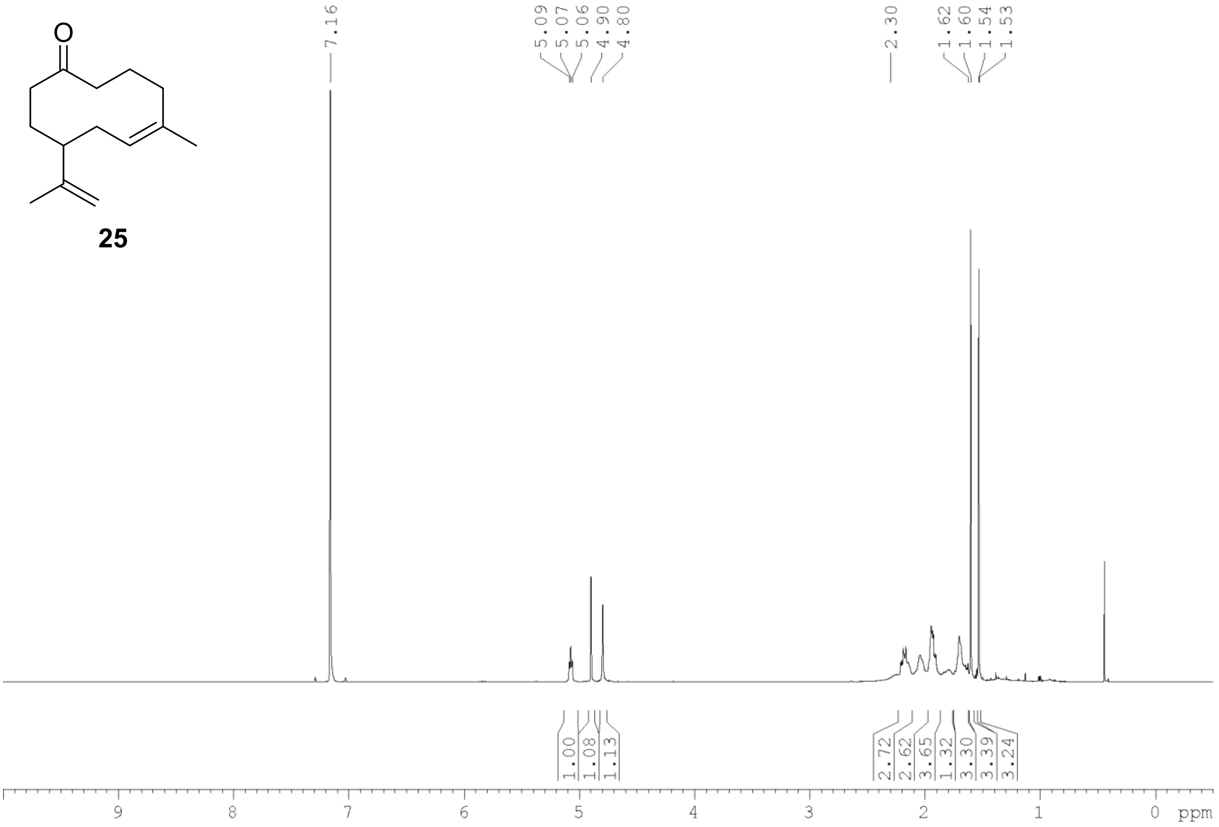
25
7.16
5.09
5.07
5.06
4.90
4.80
2.30
1.62
1.60
1.54
1.53
1.00
1.08
1.13
2.72
2.62
3.65
1.32
3.30
3.39
3.24
9 8 7 6 5 4 3 2 1 0 ppm

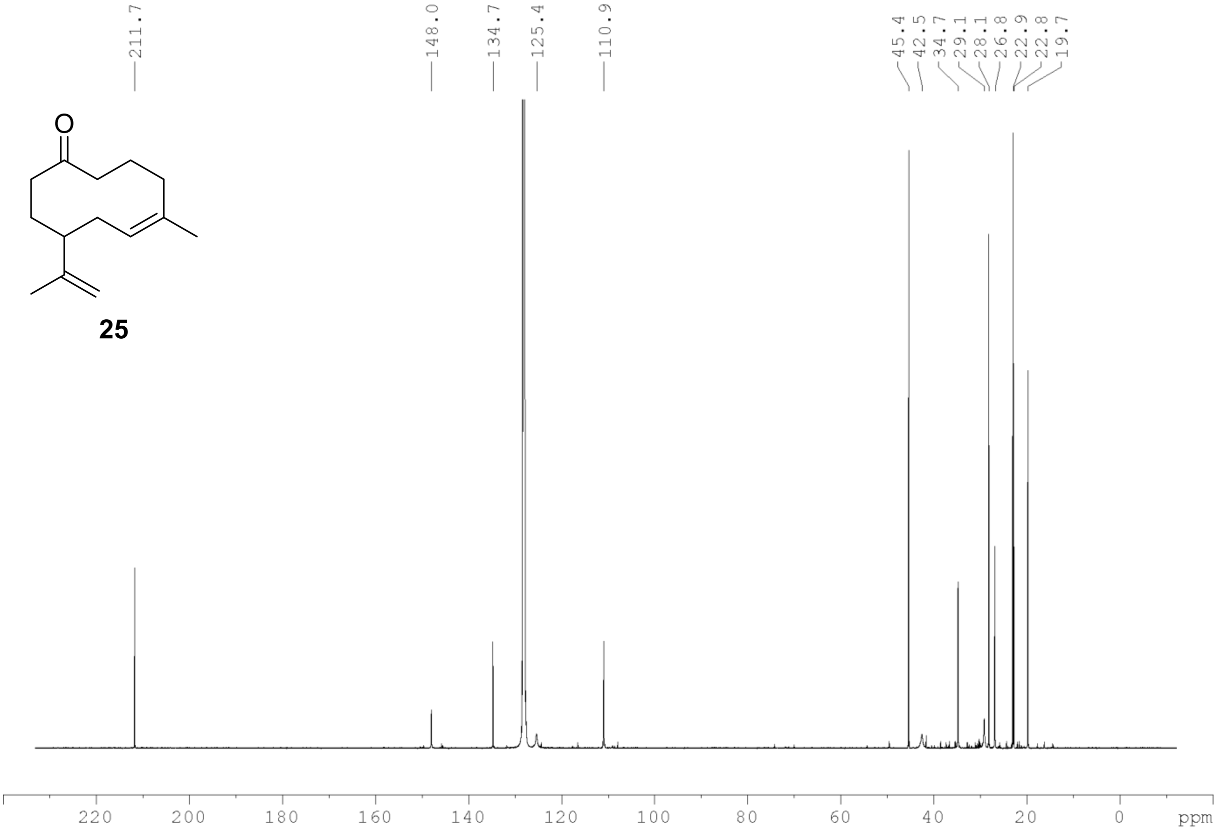
211.7
148.0
134.7
125.4
110.9
45.4
42.5
34.7
29.1
28.1
26.8
22.9
22.8
19.7
25
220 200 180 160 140 120 100 80 60 40 20 0 ppm

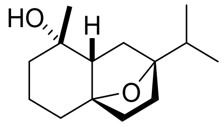

28a

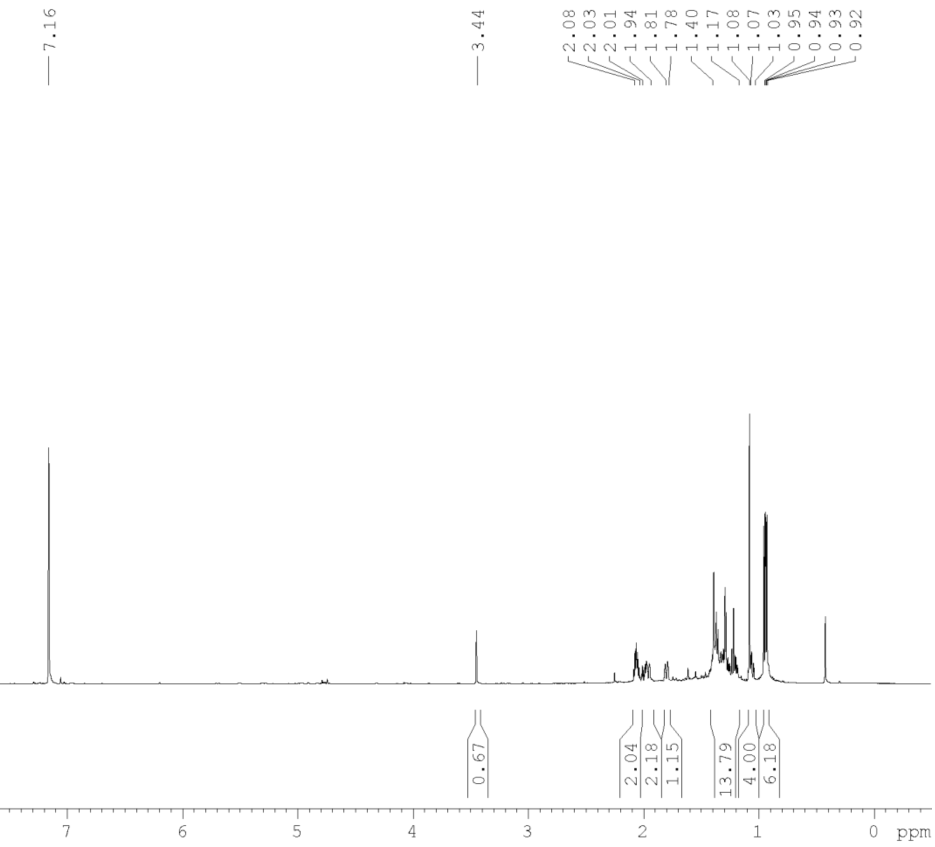
7.16
3.44
2.08
2.03
2.01
1.94
1.81
1.78
1.40
1.17
1.08
1.07
1.03
0.95
0.94
0.93
0.92
0.67
2.04
2.18
1.15
13.79
4.00
6.18
9 8 7 6 5 4 3 2 1 0 ppm

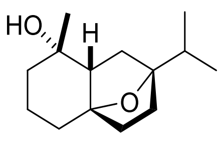

28a

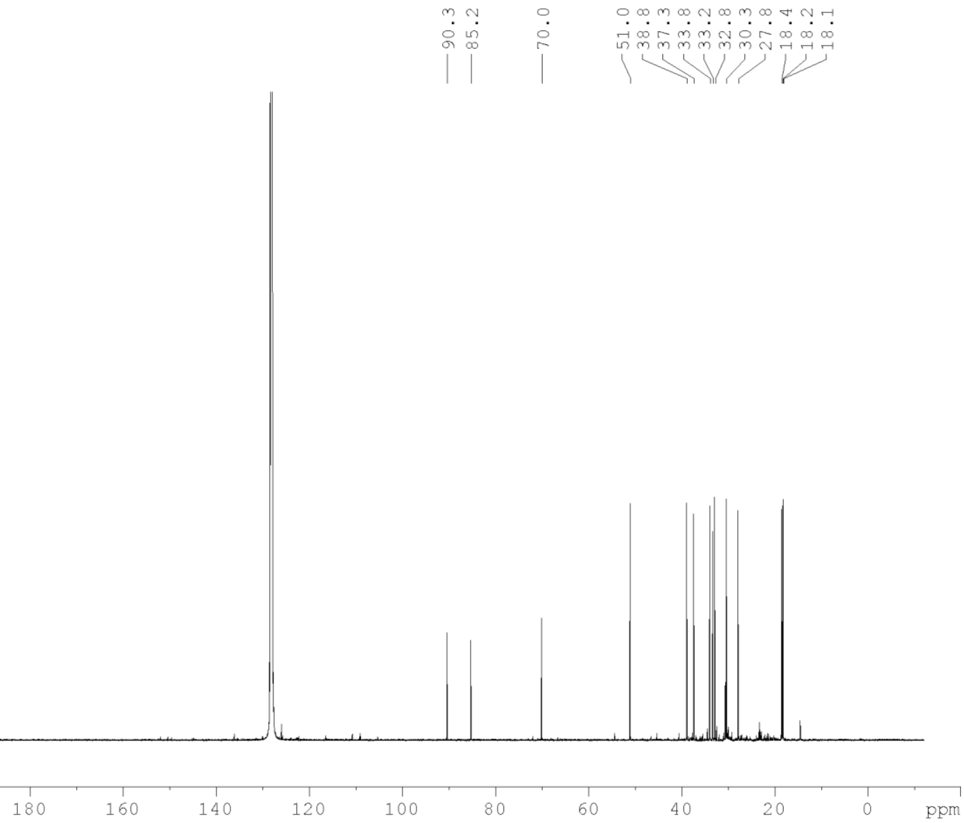
90.3
85.2
70.0
51.0
38.8
37.3
33.8
33.2
32.8
30.3
27.8
18.4
18.2
18.1
220 200 180 160 140 120 100 80 60 40 20 0 ppm

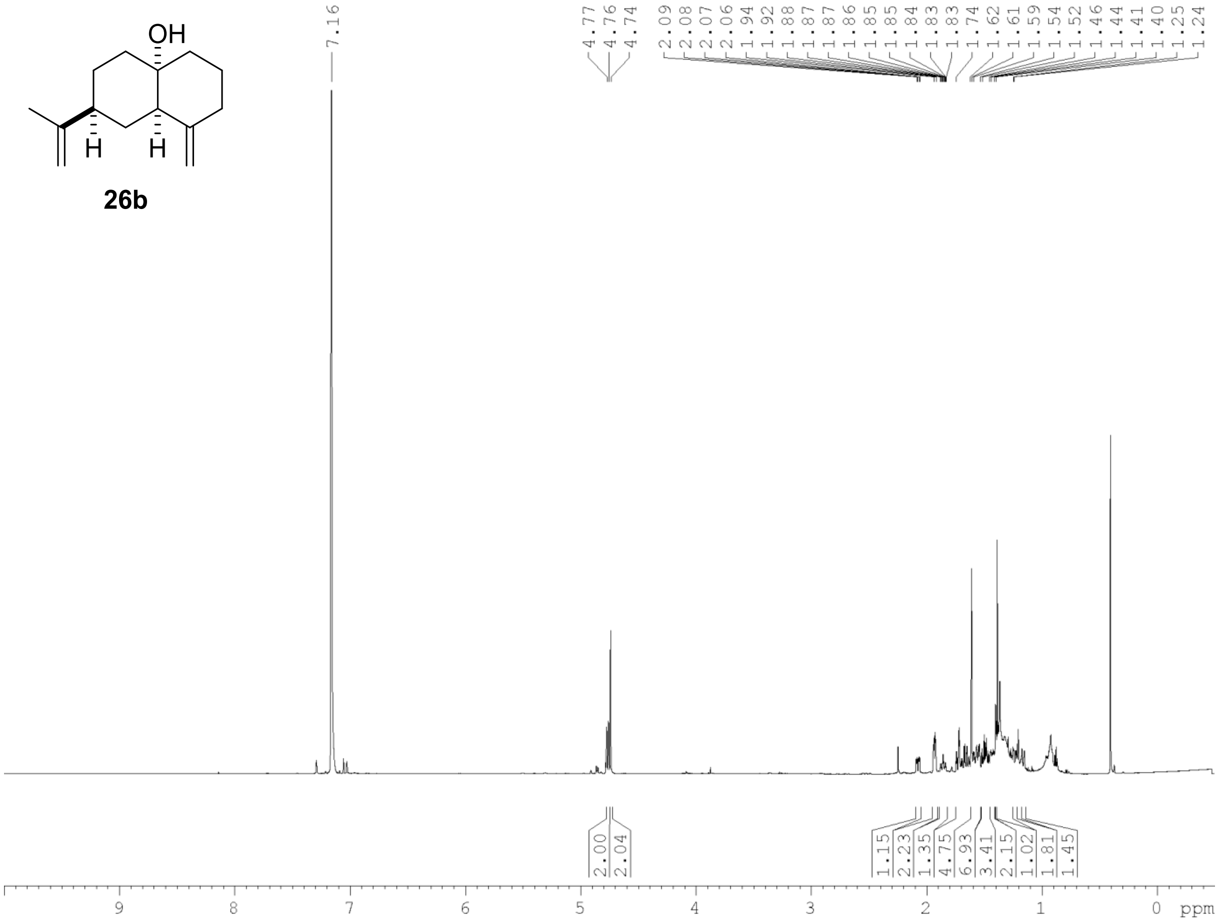
26b
7.16
4.77
4.76
4.74
2.09
2.08
2.07
2.06
1.94
1.92
1.88
1.87
1.87
1.86
1.85
1.85
1.84
1.83
1.83
1.74
1.62
1.61
1.59
1.54
1.52
1.46
1.44
1.41
1.40
1.25
1.24
2.00
2.04
1.15
2.23
1.35
4.75
6.93
3.41
2.15
1.02
1.81
1.45
9
8
7
6
5
4
3
2
1
0 ppm

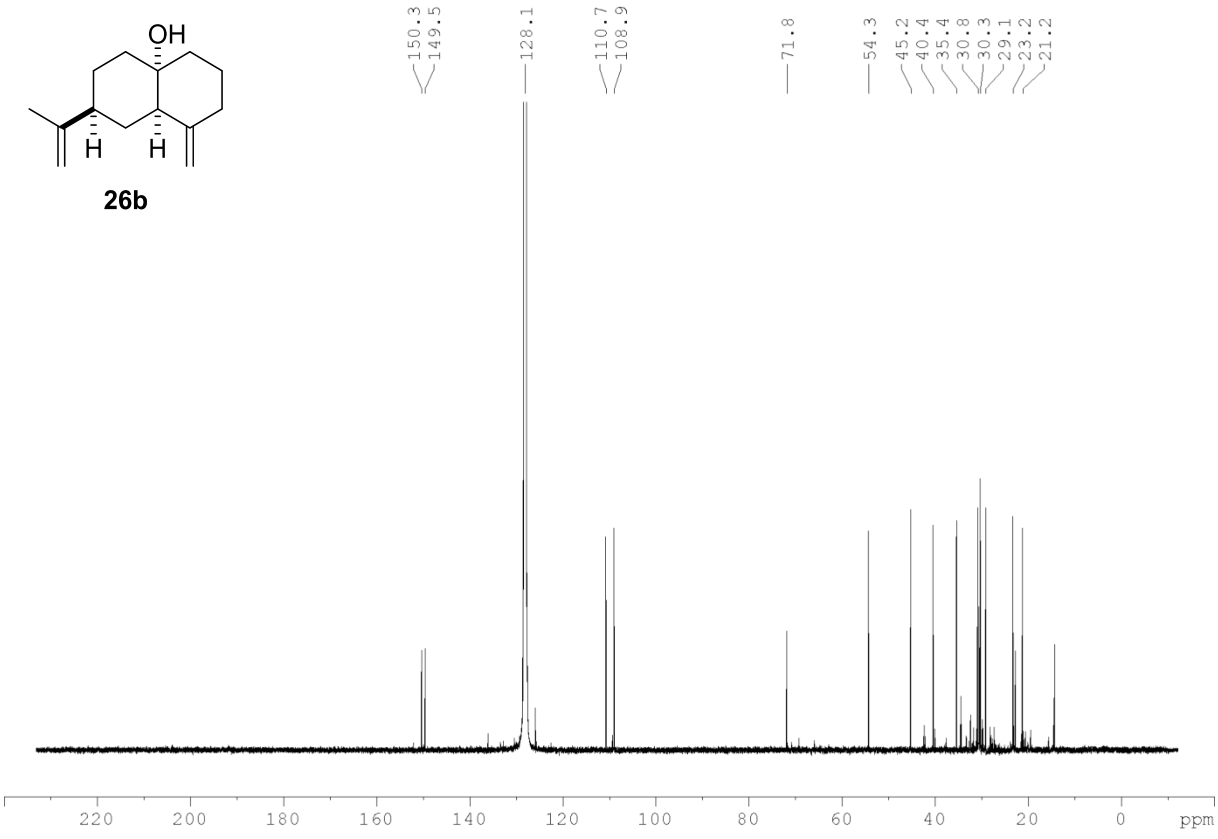
26b
150.3
149.5
128.1
110.7
108.9
71.8
54.3
45.2
40.4
35.4
30.8
30.3
29.1
23.2
21.2
220
200
180
160
140
120
100
80
60
40
20
0
ppm

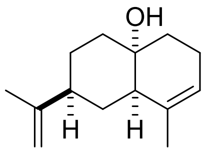

26a

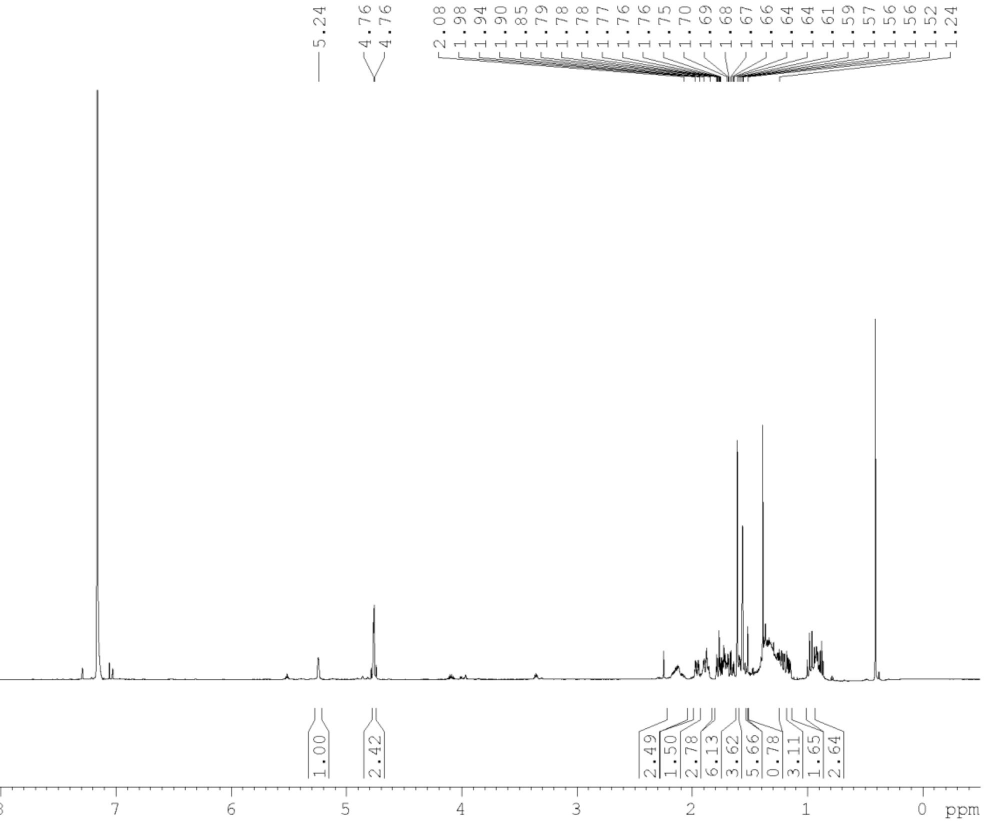
5.24
4.76
4.76
2.08
1.98
1.94
1.90
1.85
1.79
1.78
1.78
1.77
1.76
1.76
1.75
1.70
1.69
1.68
1.67
1.66
1.64
1.64
1.61
1.59
1.57
1.56
1.56
1.52
1.24
1.00
2.42
2.49
1.50
2.78
6.13
3.62
5.66
0.78
3.11
1.65
2.64
9
8
7
6
5
4
3
2
1
0 ppm

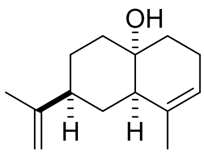

26a

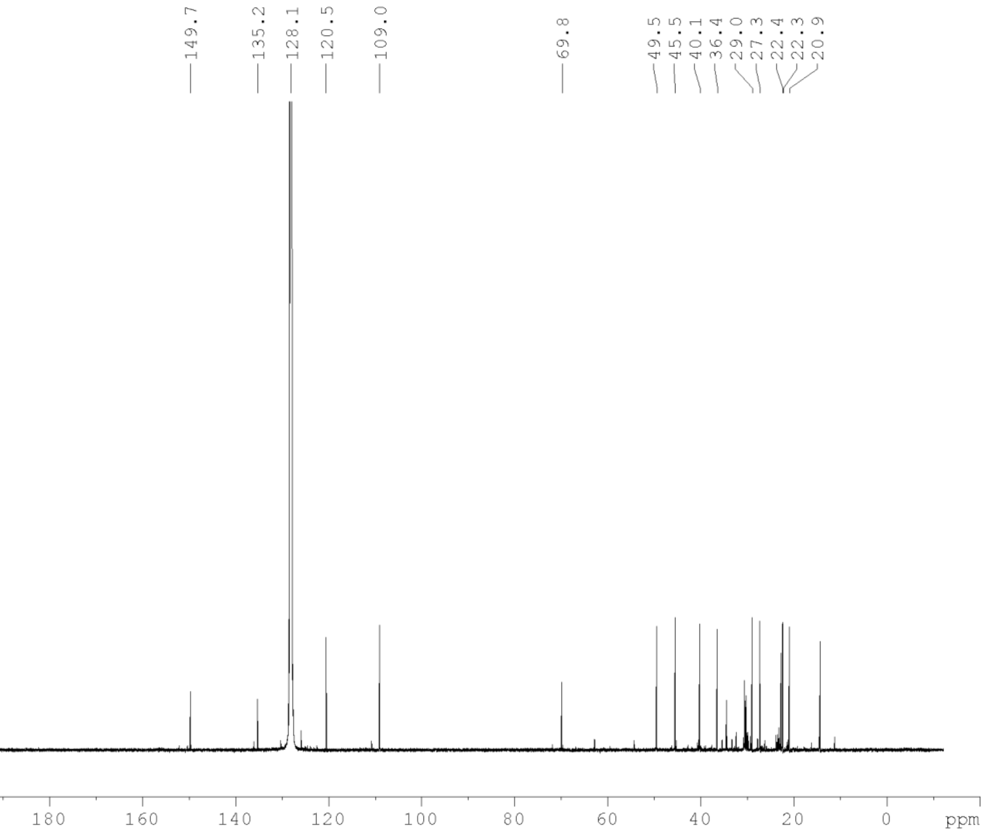
149.7
135.2
128.1
120.5
109.0
69.8
49.5
45.5
40.1
36.4
29.0
27.3
22.4
22.3
20.9
220
200
180
160
140
120
100
80
60
40
20
0
ppm

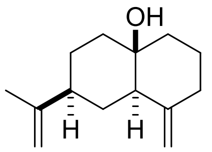
OH
H
H

27b

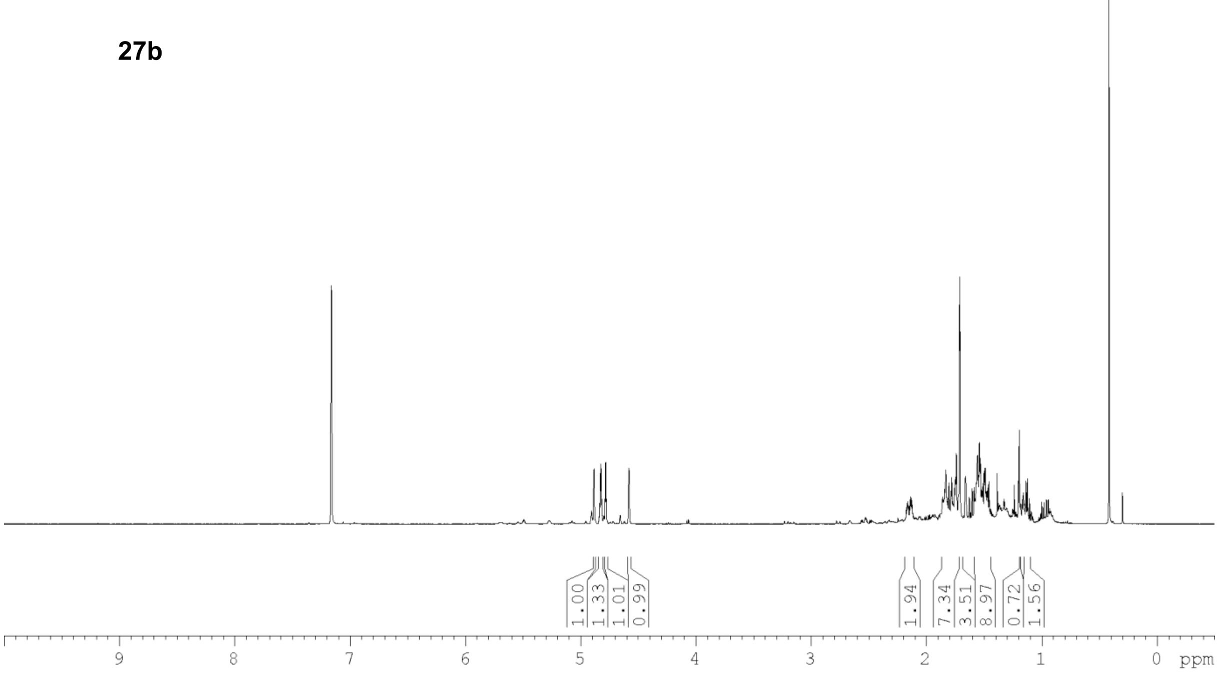
1.00
1.33
1.01
0.99
1.94
7.34
3.51
8.97
0.72
1.56
9
8
7
6
5
4
3
2
1
0
ppm

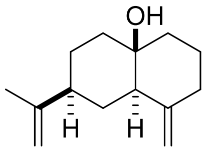
OH
H
H

27b

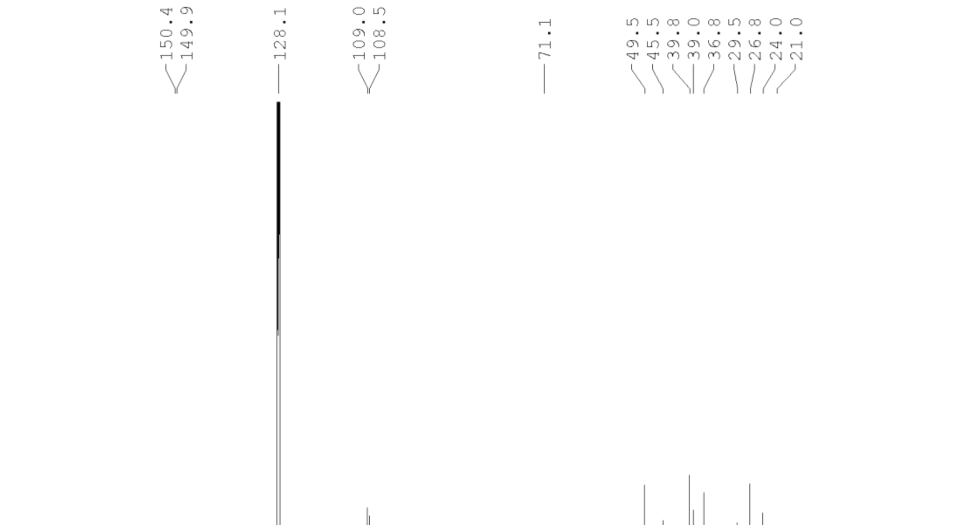
150.4
149.9
128.1
109.0
108.5
71.1
49.5
45.5
39.8
39.0
36.8
29.5
26.8
24.0
21.0
220
200
180
160
140
120
100
80
60
40
20
0
ppm

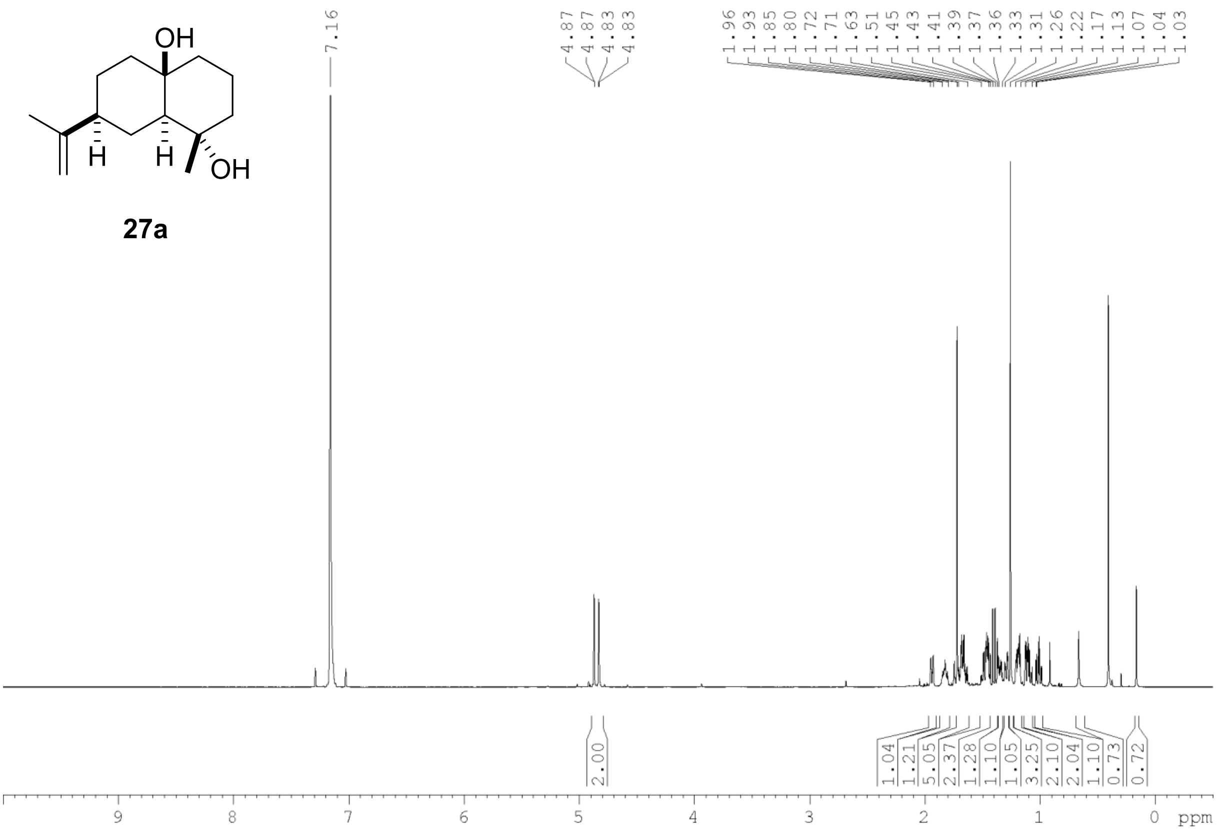
OH
H
H
OH
27a
7.16
4.87
4.87
4.83
4.83
1.96 1.93 1.85 1.80 1.72 1.71 1.63 1.51 1.45 1.43 1.41 1.39 1.37 1.36 1.33 1.31 1.26 1.22 1.17 1.13 1.07 1.04 1.03
2.00
1.04 1.21 5.05 2.37 1.28 1.10 1.05 3.25 2.10 2.04 1.10 0.73 0.72
9 8 7 6 5 4 3 2 1 0 ppm

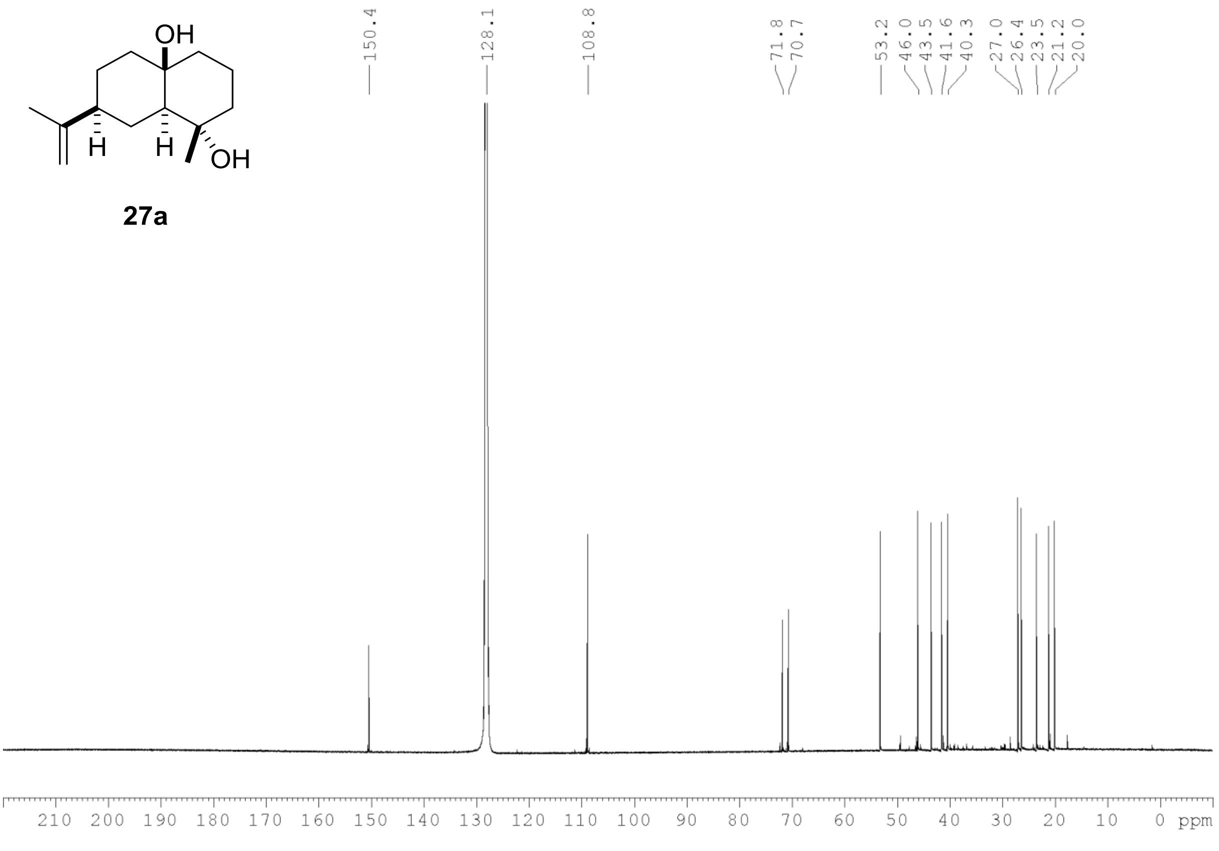
OH
H
H
OH
27a
150.4
128.1
108.8
71.8
70.7
53.2 46.0 43.5 41.6 40.3
27.0 26.4 23.5 21.2 20.0
210 200 190 180 170 160 150 140 130 120 110 100 90 80 70 60 50 40 30 20 10 0 ppm

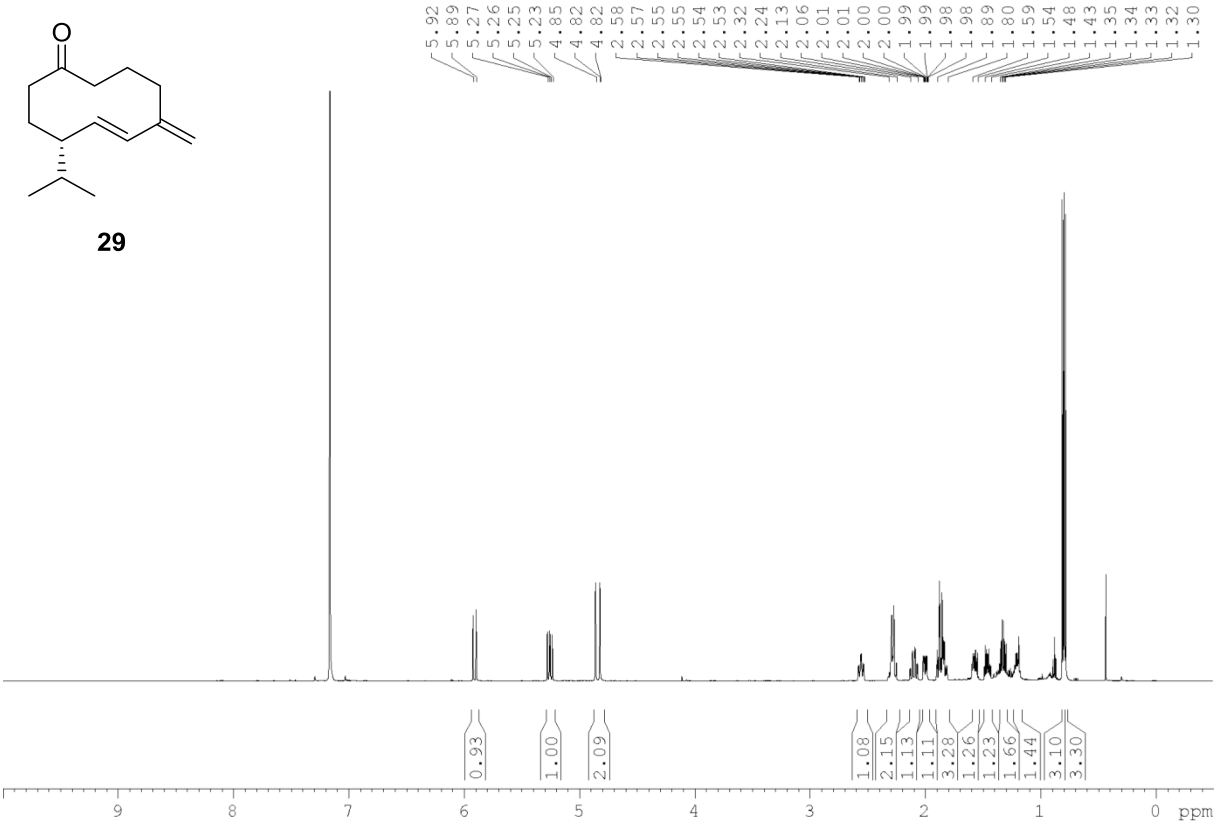
29
5.92
5.89
5.27
5.26
5.25
5.23
4.85
4.82
4.82
2.58
2.57
2.55
2.55
2.54
2.53
2.32
2.24
2.13
2.06
2.01
2.01
2.00
2.00
1.99
1.99
1.98
1.98
1.89
1.80
1.59
1.54
1.48
1.43
1.35
1.34
1.33
1.32
1.30
0.93
1.00
2.09
1.08
2.15
1.13
1.11
3.28
1.26
1.23
1.66
1.44
3.10
3.30
9 8 7 6 5 4 3 2 1 0 ppm

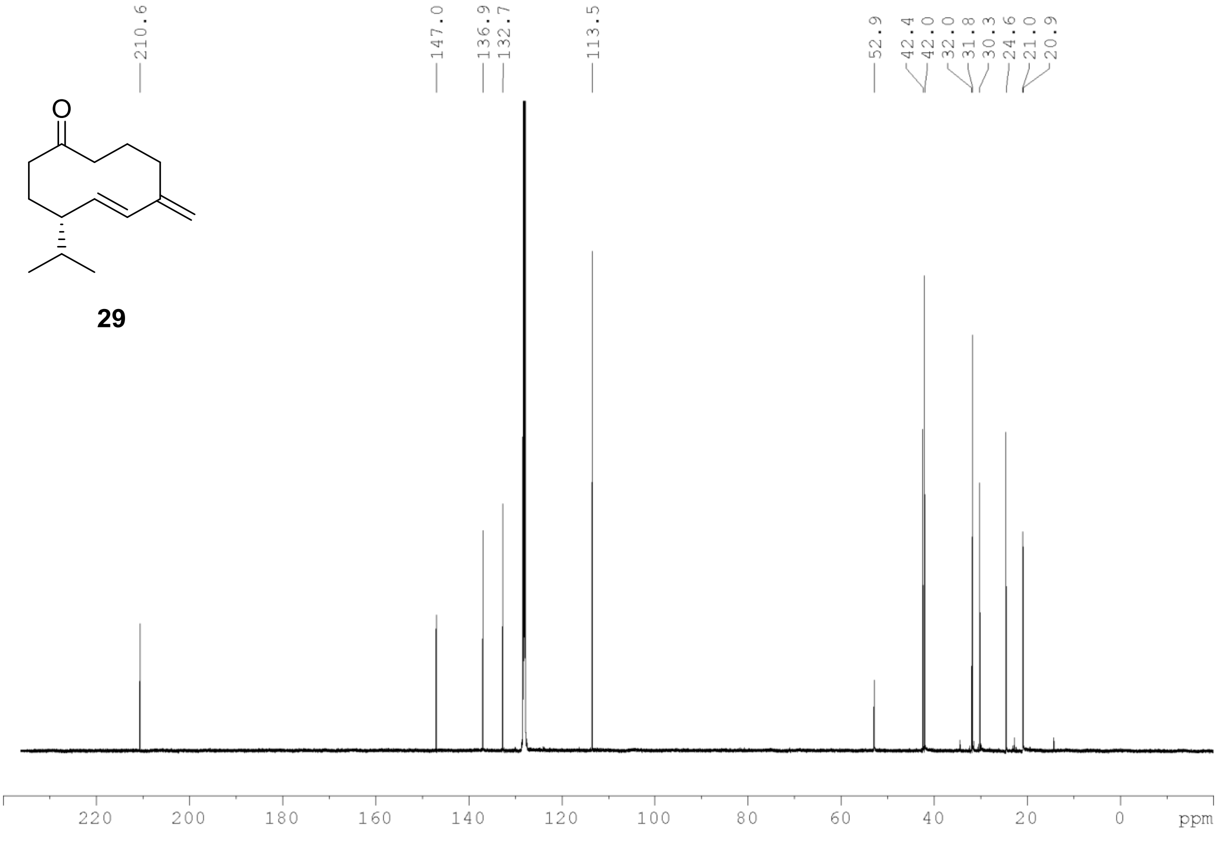
210.6
147.0
136.9
132.7
113.5
52.9
42.4
42.0
32.0
31.8
30.3
24.6
21.0
20.9
29
220 200 180 160 140 120 100 80 60 40 20 0 ppm

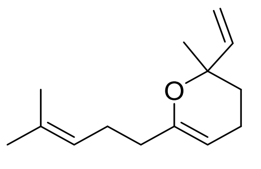

30

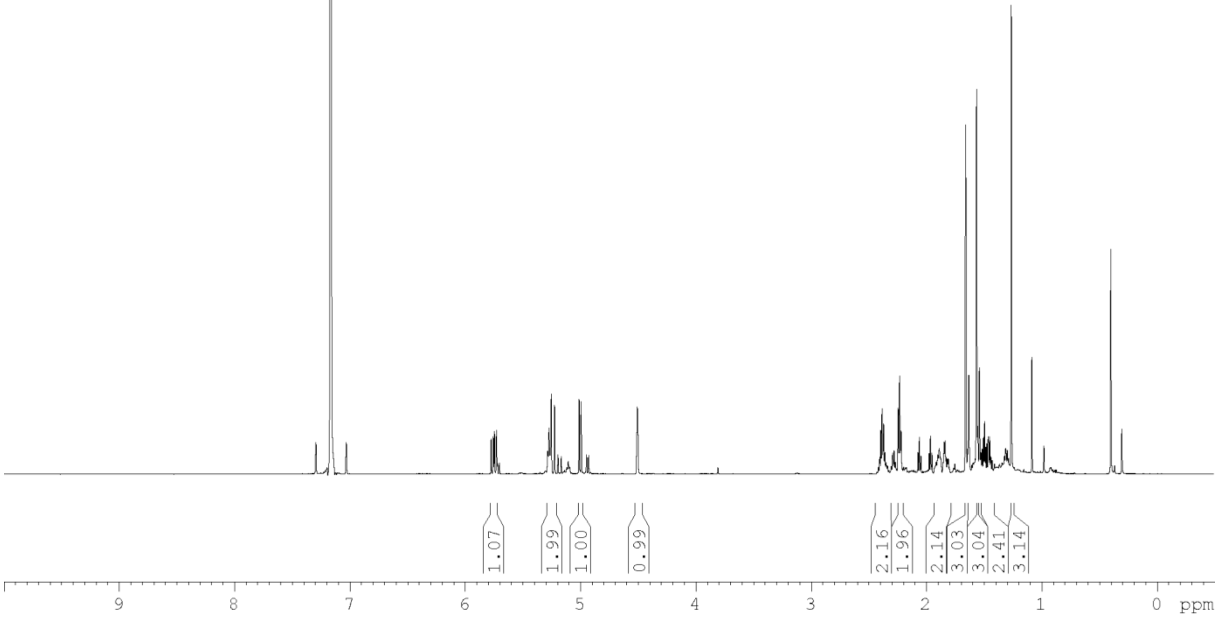
5.77
5.76
5.75
5.73
5.28
5.22
5.01
4.99
4.51
2.43
2.32
2.24
2.23
2.21
1.92
1.80
1.65
1.56
1.52
1.42
1.26
1.07
1.99
1.00
0.99
2.16
1.96
2.14
3.03
3.04
2.41
3.14
9
8
7
6
5
4
3
2
1
0 ppm

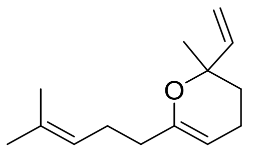

30

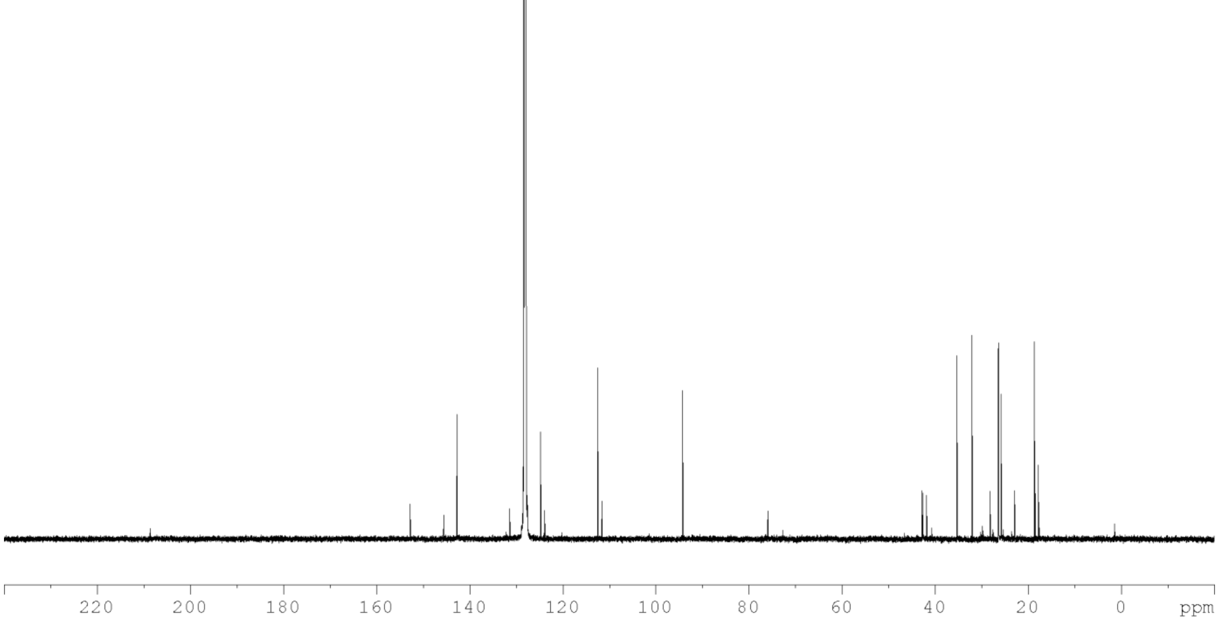
152.7
142.7
131.4
124.8
112.5
94.3
75.9
35.3
32.1
26.4
26.4
25.9
18.7
17.8
220
200
180
160
140
120
100
80
60
40
20
0
ppm

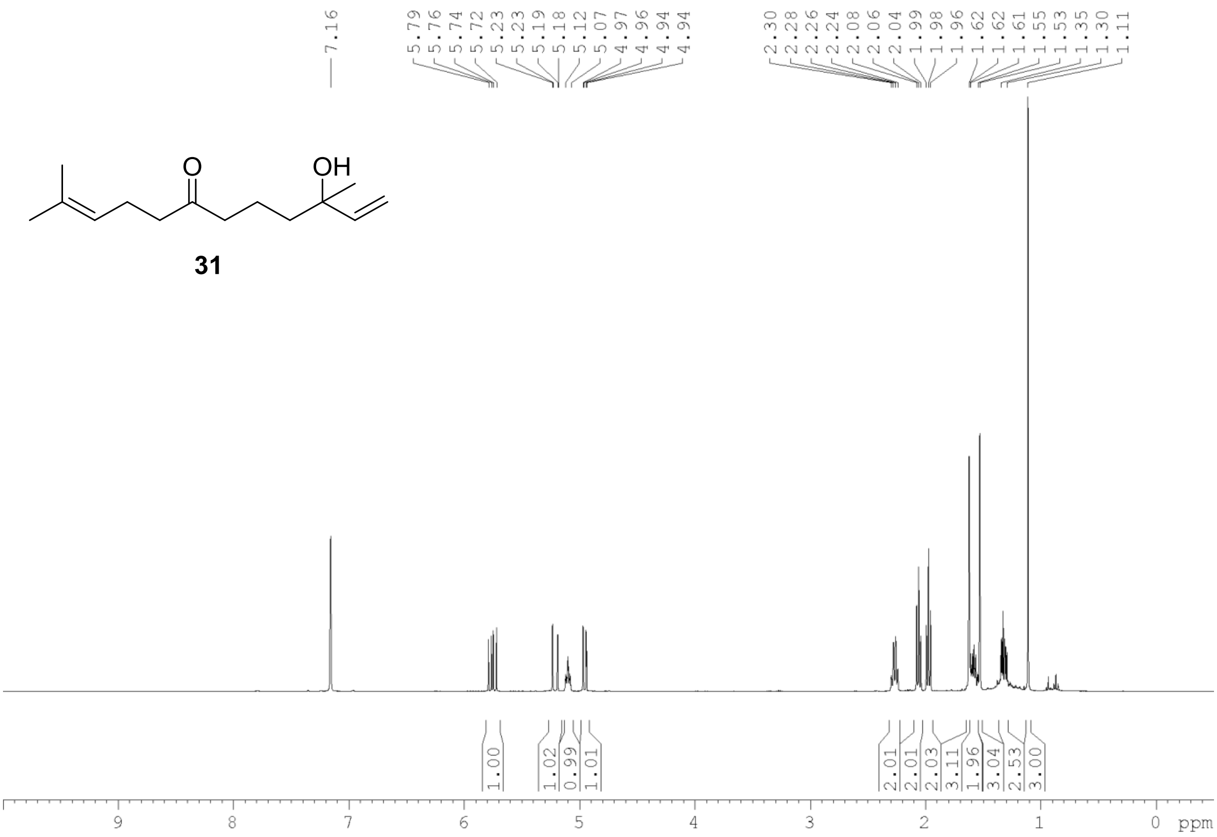
7.16
5.79
5.76
5.74
5.72
5.23
5.23
5.19
5.18
5.12
5.07
4.97
4.96
4.94
4.94
2.30
2.28
2.26
2.24
2.08
2.06
2.04
1.99
1.98
1.96
1.62
1.62
1.61
1.55
1.53
1.35
1.30
1.11
O
OH
31
1.00
1.02
0.99
1.01
2.01
2.01
2.03
3.11
1.96
3.04
2.53
3.00
9
8
7
6
5
4
3
2
1
0 ppm

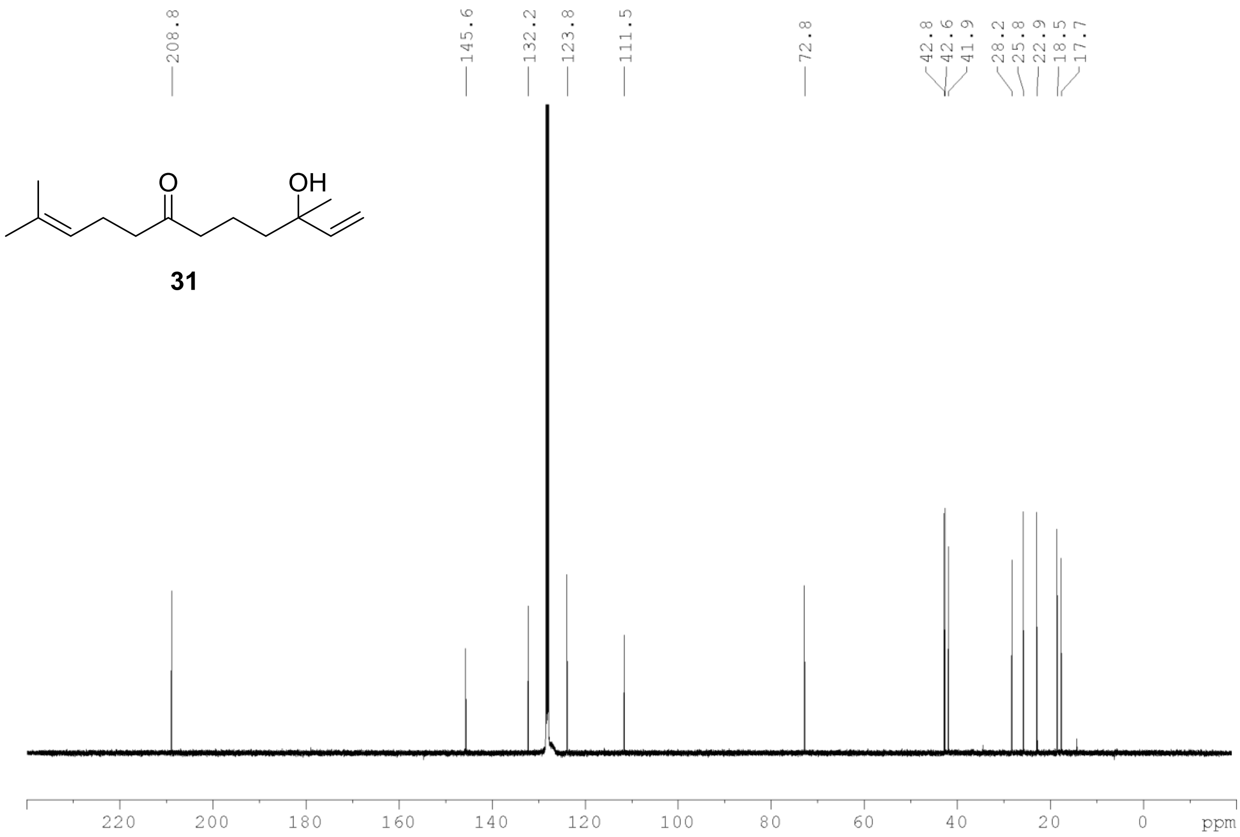
208.8
145.6
132.2
123.8
111.5
72.8
42.8
42.6
41.9
28.2
25.8
22.9
18.5
17.7
O
OH
31
220
200
180
160
140
120
100
80
60
40
20
0
ppm

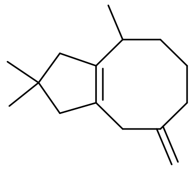

33

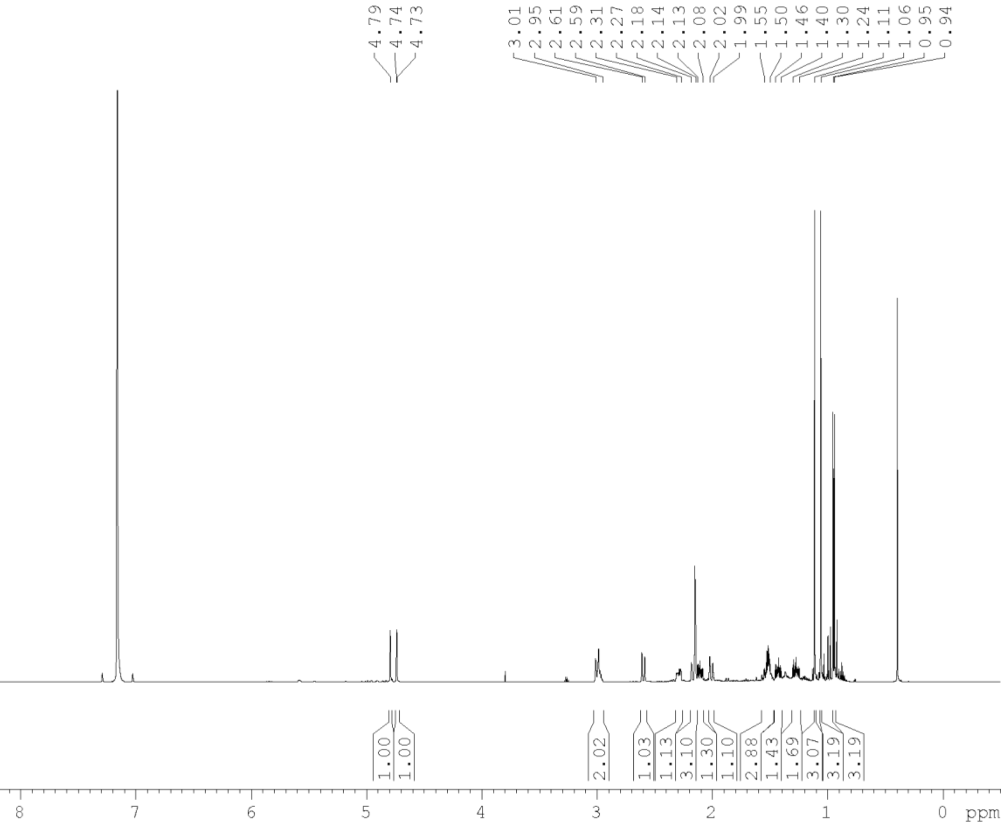
4.79
4.74
4.73
3.01
2.95
2.61
2.59
2.31
2.27
2.18
2.14
2.13
2.08
2.02
1.99
1.55
1.50
1.46
1.40
1.30
1.24
1.11
1.06
0.95
0.94
1.00
1.00
2.02
1.03
1.13
3.10
1.30
1.10
2.88
1.43
1.69
3.07
3.19
3.19
9
8
7
6
5
4
3
2
1
0 ppm

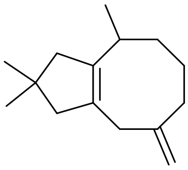

33

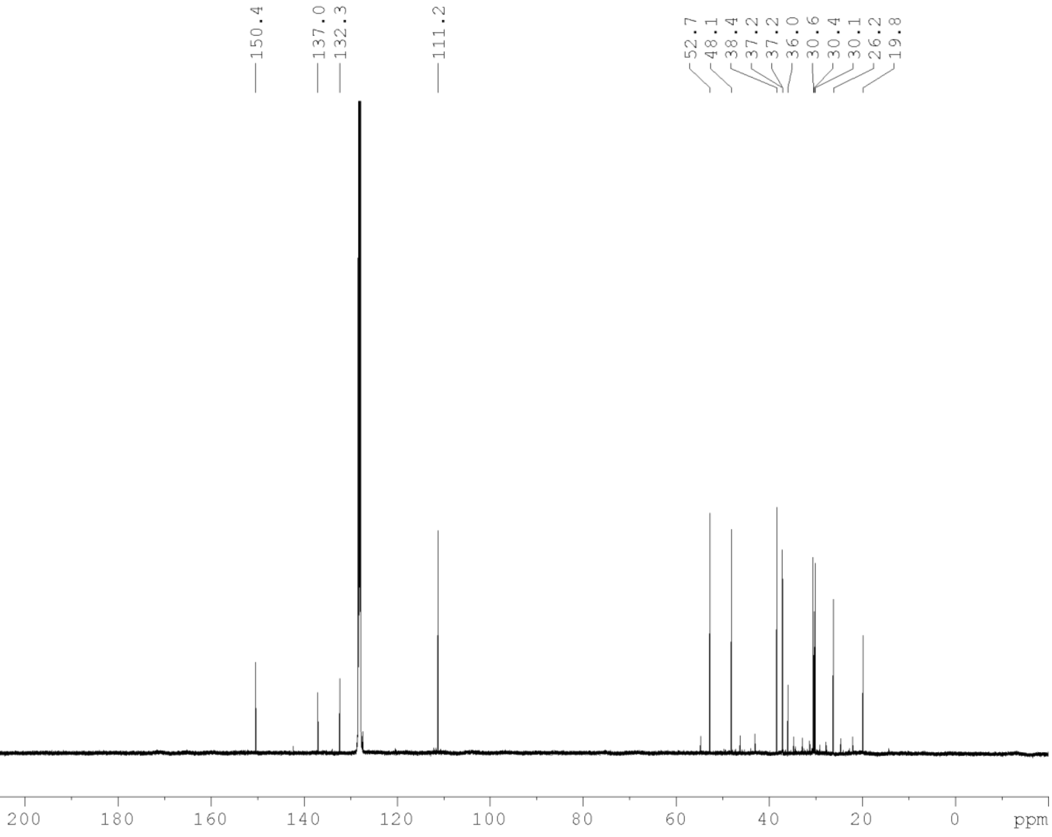
150.4
137.0
132.3
111.2
52.7
48.1
38.4
37.2
37.2
36.0
30.6
30.4
30.1
26.2
19.8
220
200
180
160
140
120
100
80
60
40
20
0
ppm

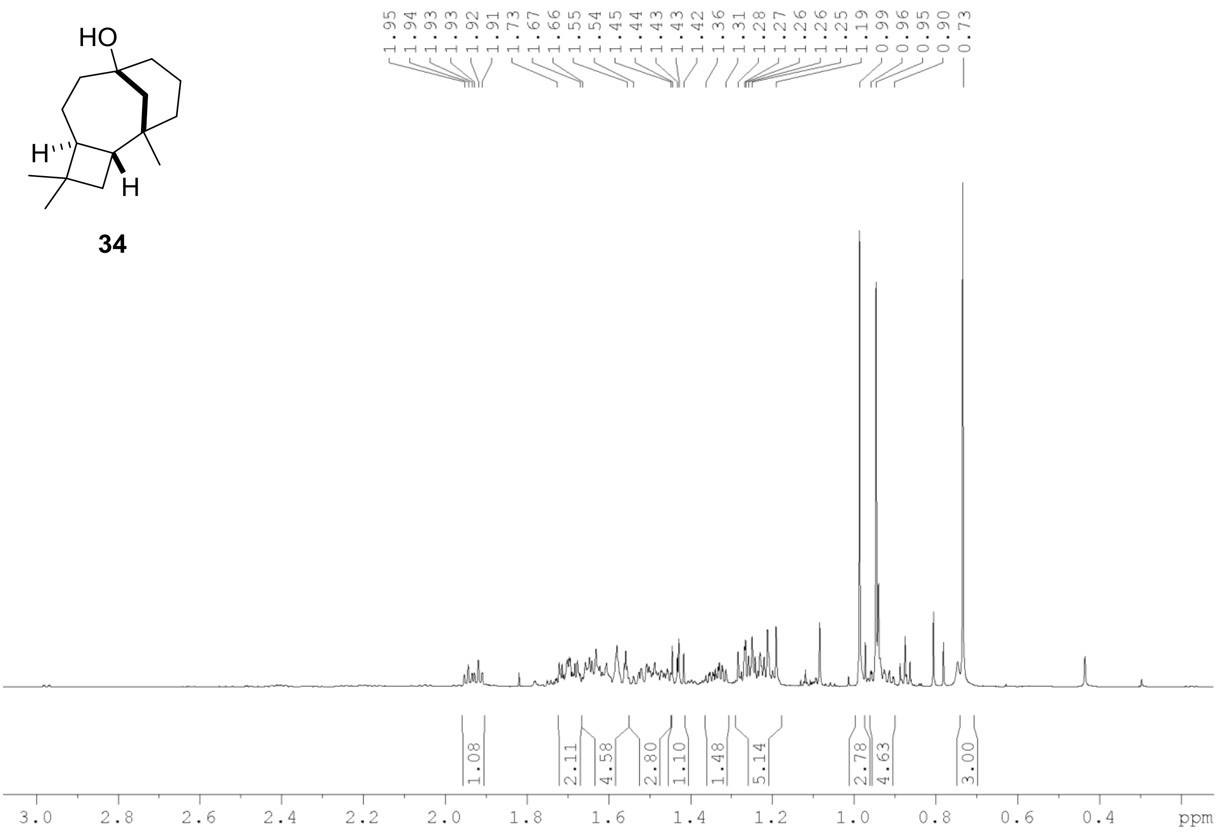
HO
H
H
34
1.95 1.94 1.93 1.93 1.92 1.91 1.73 1.67 1.66 1.55 1.54 1.45 1.44 1.43 1.43 1.42 1.36 1.31 1.28 1.27 1.26 1.26 1.25 1.19 0.99 0.96 0.95 0.90 0.73
1.08 2.11 4.58 2.80 1.10 1.48 5.14 2.78 4.63 3.00
3.0 2.8 2.6 2.4 2.2 2.0 1.8 1.6 1.4 1.2 1.0 0.8 0.6 0.4 ppm

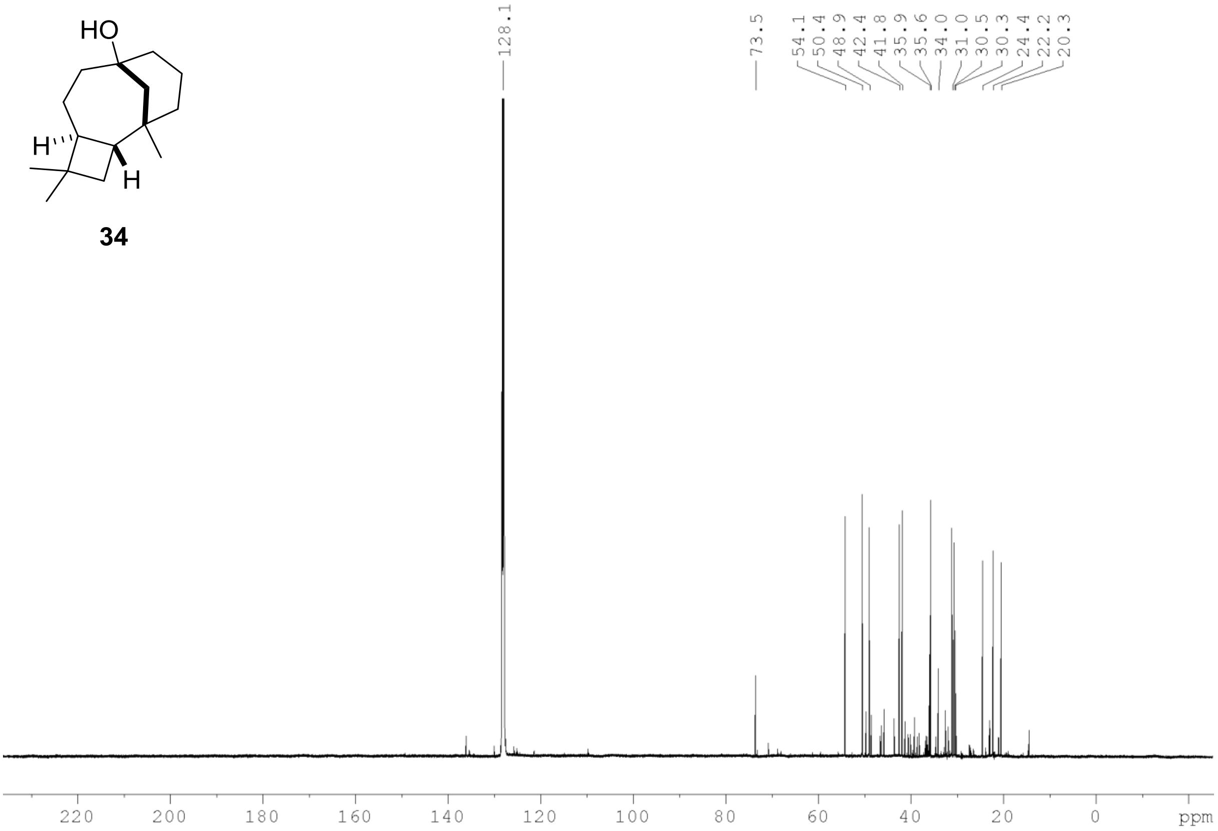
HO
H
H
34
128.1
73.5
54.1 50.4 48.9 42.4 41.8 35.9 35.6 34.0 31.0 30.5 30.3 24.4 22.2 20.3
220 200 180 160 140 120 100 80 60 40 20 0 ppm

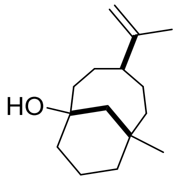

37

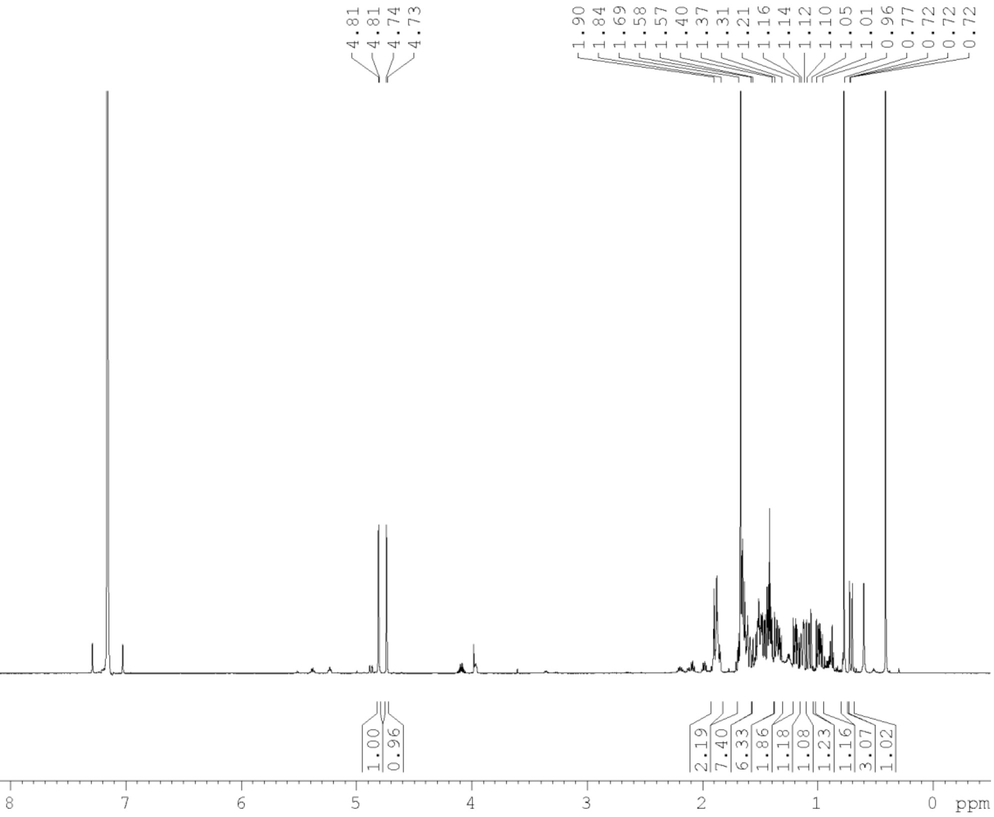
4.81
4.81
4.74
4.73
1.90
1.84
1.69
1.58
1.57
1.40
1.37
1.31
1.21
1.16
1.14
1.12
1.10
1.05
1.01
0.96
0.77
0.72
0.72
0.72
1.00
0.96
2.19
7.40
6.33
1.86
1.18
1.08
1.23
1.16
3.07
1.02
9 8 7 6 5 4 3 2 1 0 ppm

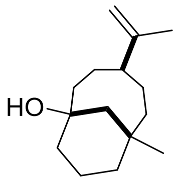

37

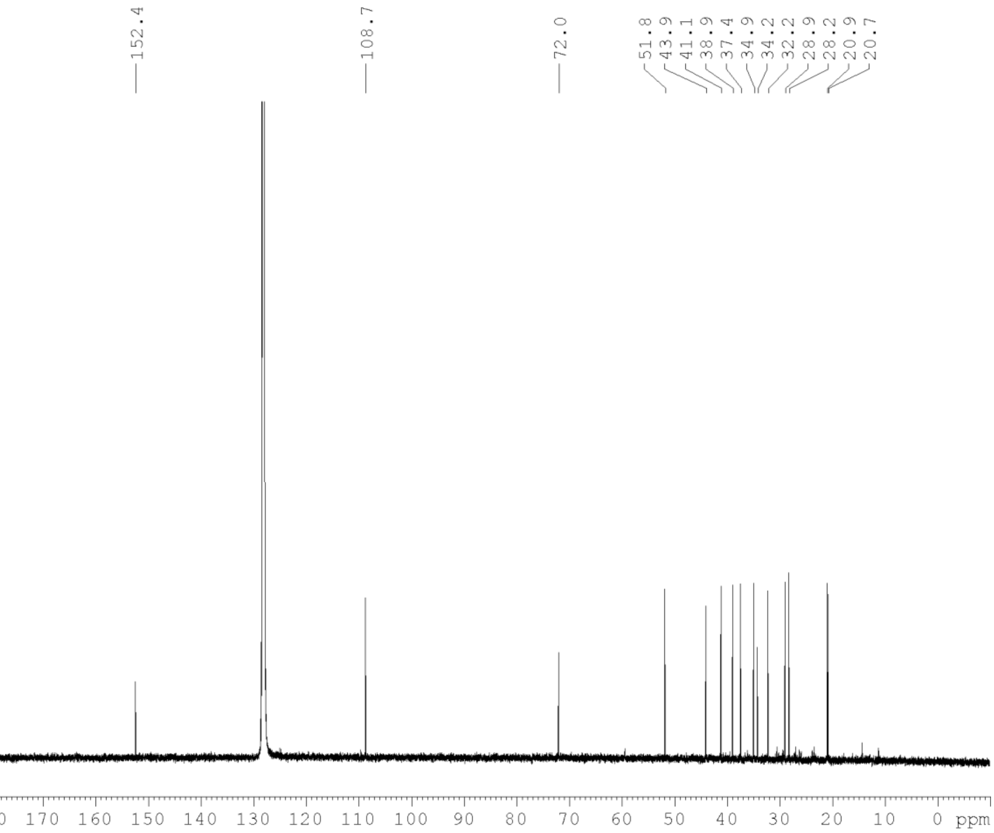
152.4
108.7
72.0
51.8
43.9
41.1
38.9
37.4
34.9
34.2
32.2
28.9
28.2
20.9
20.7
210 200 190 180 170 160 150 140 130 120 110 100 90 80 70 60 50 40 30 20 10 0 ppm

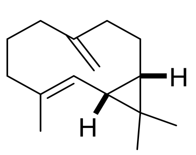
H
H

38

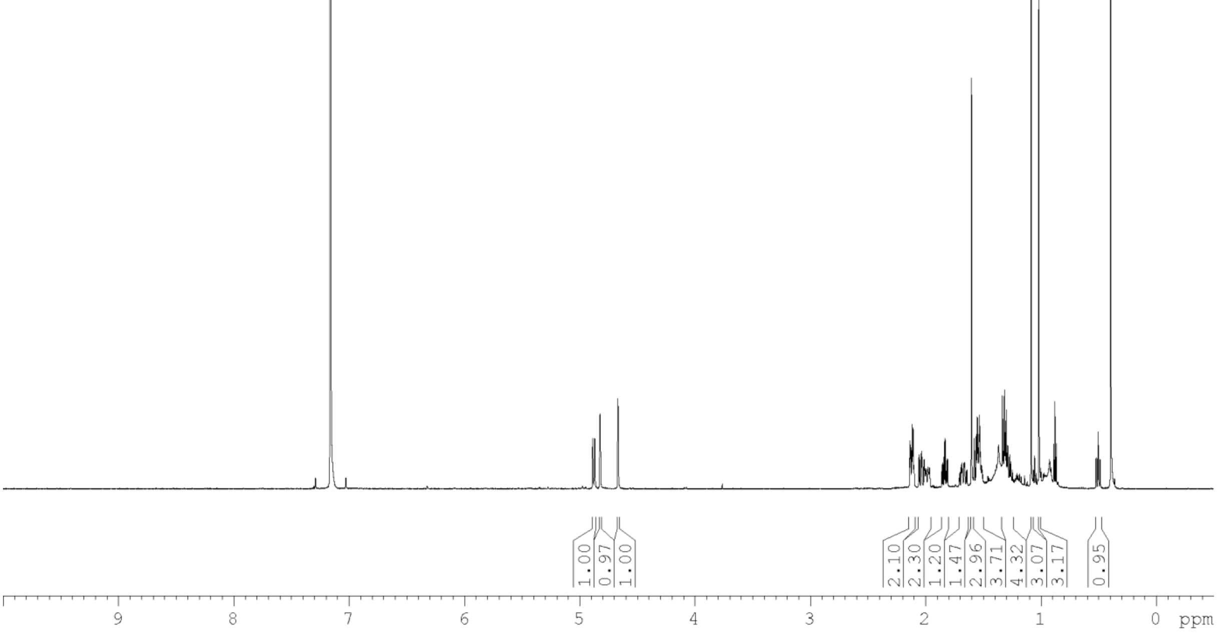
4.88
4.87
4.82
4.82
4.67
2.14
2.10
2.06
1.96
1.86
1.85
1.83
1.83
1.81
1.81
1.71
1.64
1.60
1.60
1.58
1.50
1.33
1.26
1.08
1.01
0.52
0.52
0.51
1.00
0.97
1.00
2.10
2.30
1.20
1.47
2.96
3.71
4.32
3.07
3.17
0.95
9
8
7
6
5
4
3
2
1
0 ppm

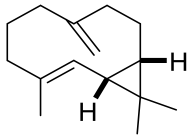
H
H

38

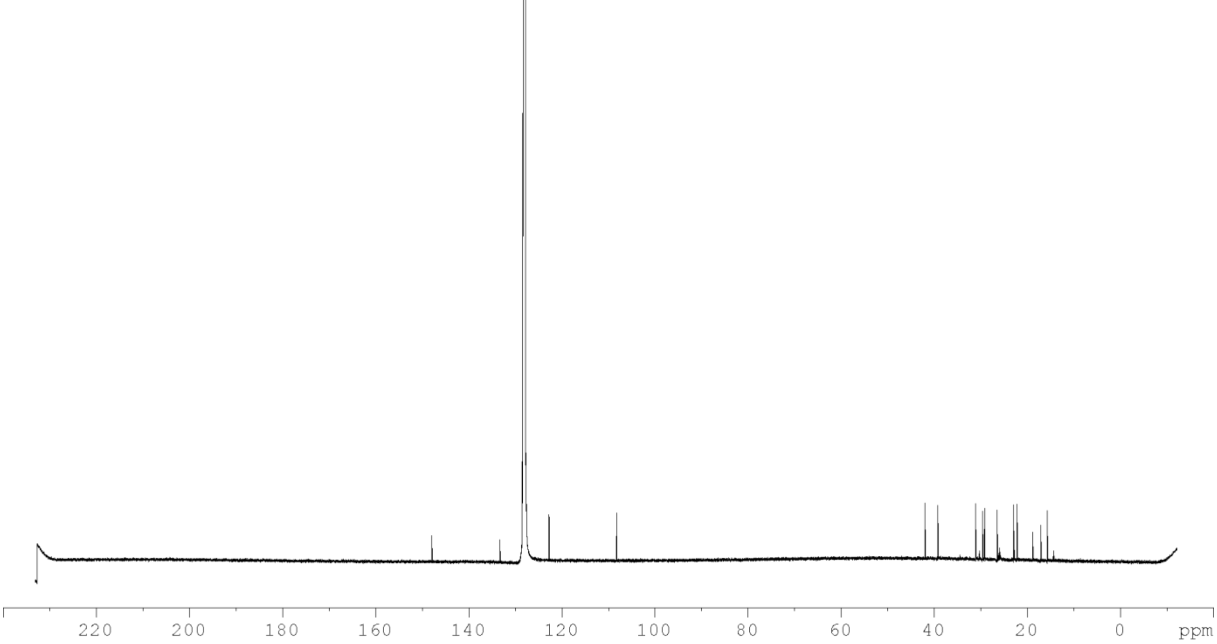
147.9
133.3
122.7
108.2
42.0
39.1
31.0
29.6
29.1
26.4
22.9
22.1
18.8
17.1
15.7
220
200
180
160
140
120
100
80
60
40
20
0
ppm

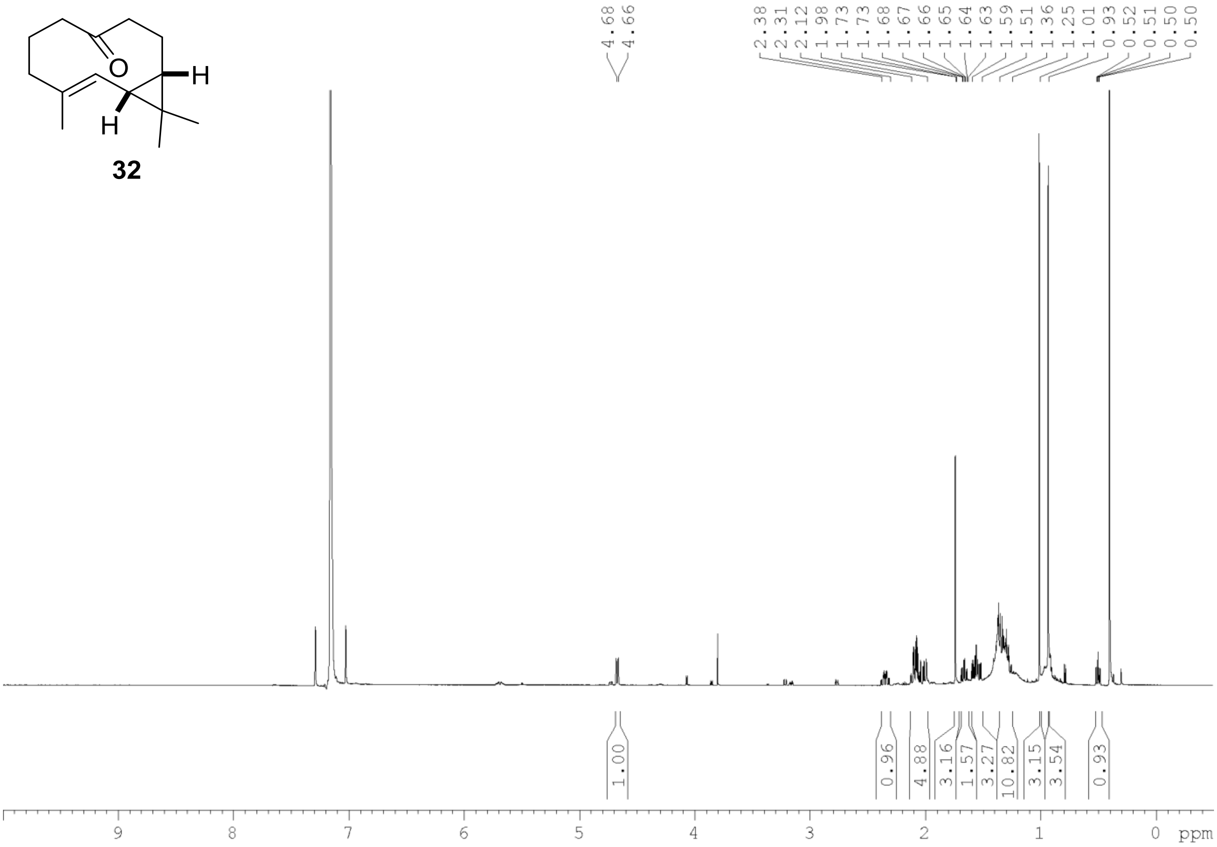
O
H
H
32
4.68
4.66
2.38
2.31
2.12
1.98
1.73
1.73
1.68
1.67
1.66
1.65
1.64
1.63
1.59
1.51
1.36
1.25
1.01
0.93
0.52
0.51
0.50
0.50
1.00
0.96
4.88
3.16
1.57
3.27
10.82
3.15
3.54
0.93
9 8 7 6 5 4 3 2 1 0 ppm

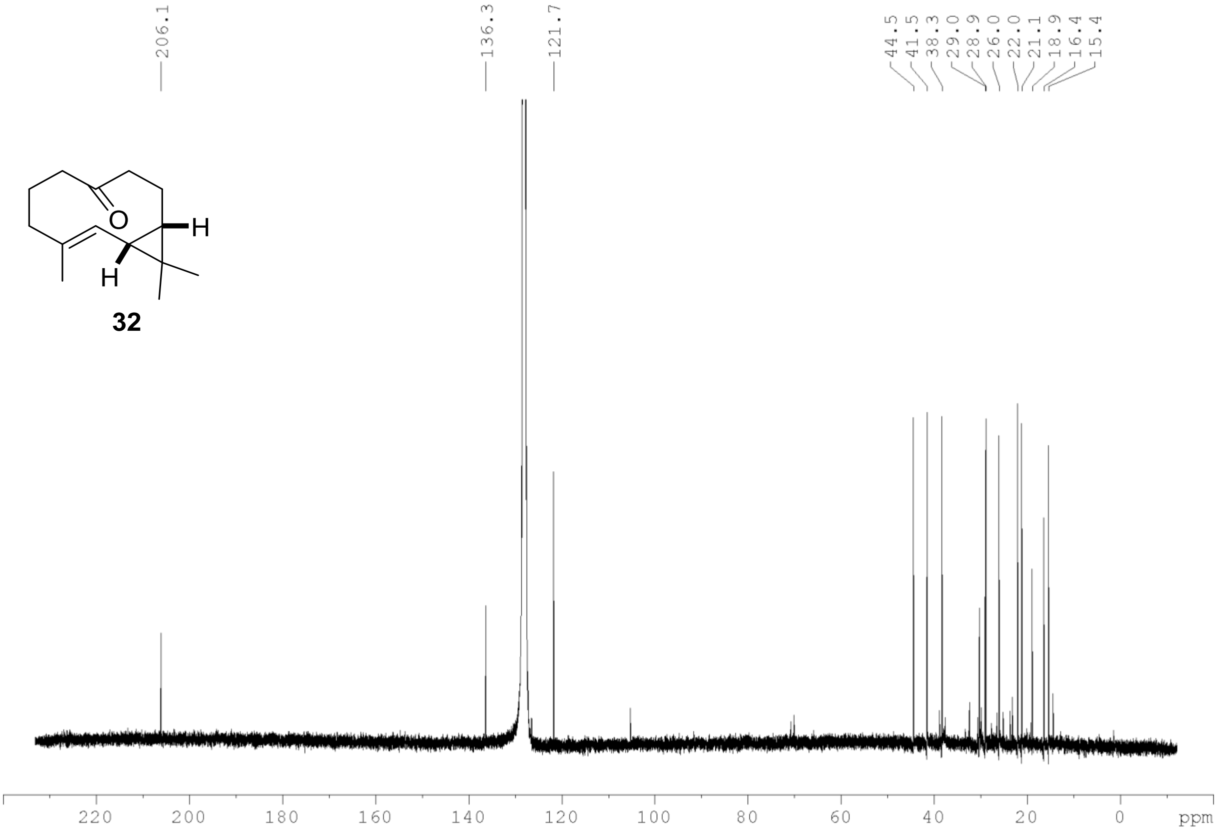
206.1
136.3
121.7
44.5
41.5
38.3
29.0
28.9
26.0
22.0
21.1
18.9
16.4
15.4
O
H
H
32
220 200 180 160 140 120 100 80 60 40 20 0 ppm

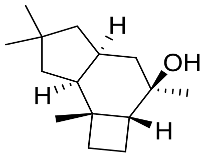
H
OH
H
H

44

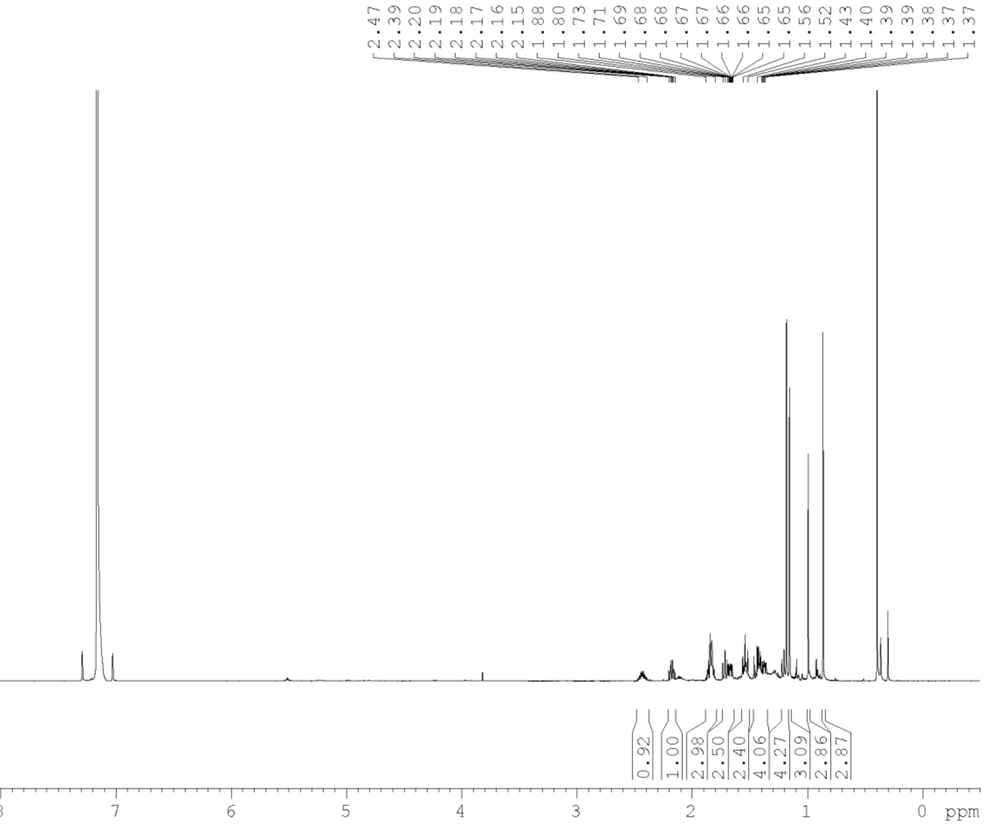
2.47 2.39 2.20 2.19 2.18 2.17 2.16 2.15 1.88 1.80 1.73 1.71 1.69 1.68 1.68 1.67 1.67 1.66 1.66 1.65 1.65 1.56 1.52 1.43 1.40 1.39 1.39 1.38 1.37 1.37
0.92 1.00 2.98 2.50 2.40 4.06 4.27 3.09 2.86 2.87
9 8 7 6 5 4 3 2 1 0 ppm

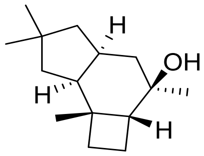
H
OH
H
H

44

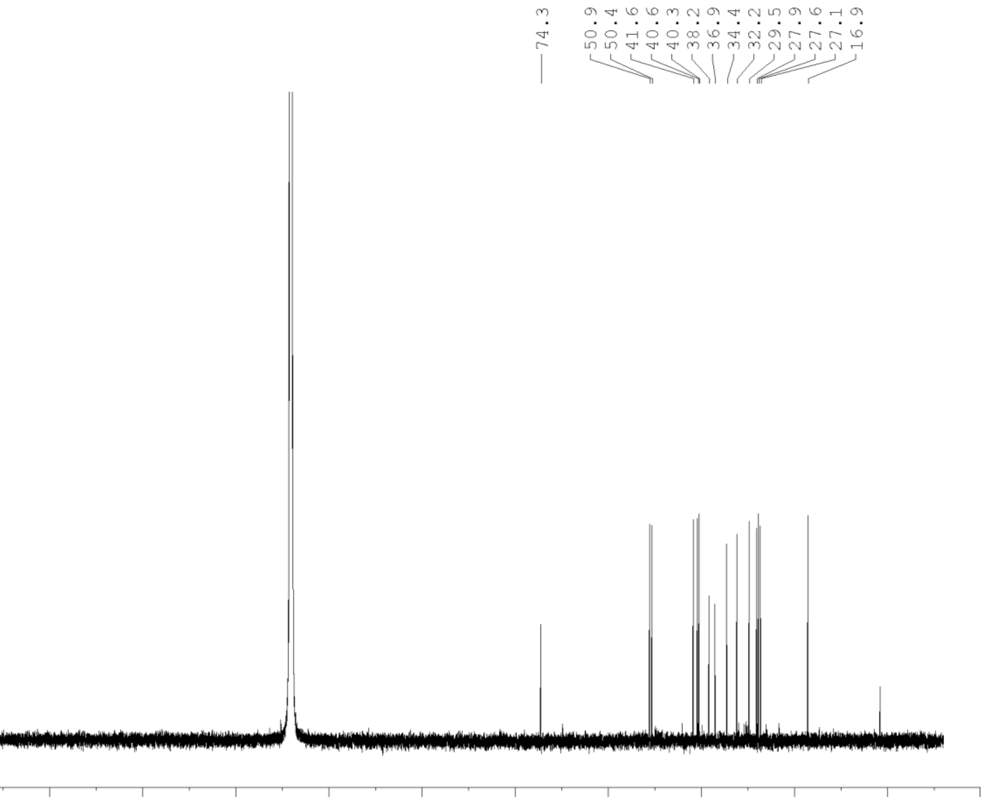
74.3
50.9 50.4 41.6 40.6 40.3 38.2 36.9 34.4 32.2 29.5 27.9 27.6 27.1 16.9
220 200 180 160 140 120 100 80 60 40 20 0 ppm

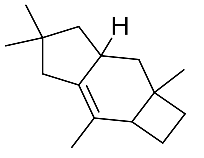

42

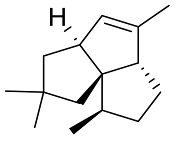

43

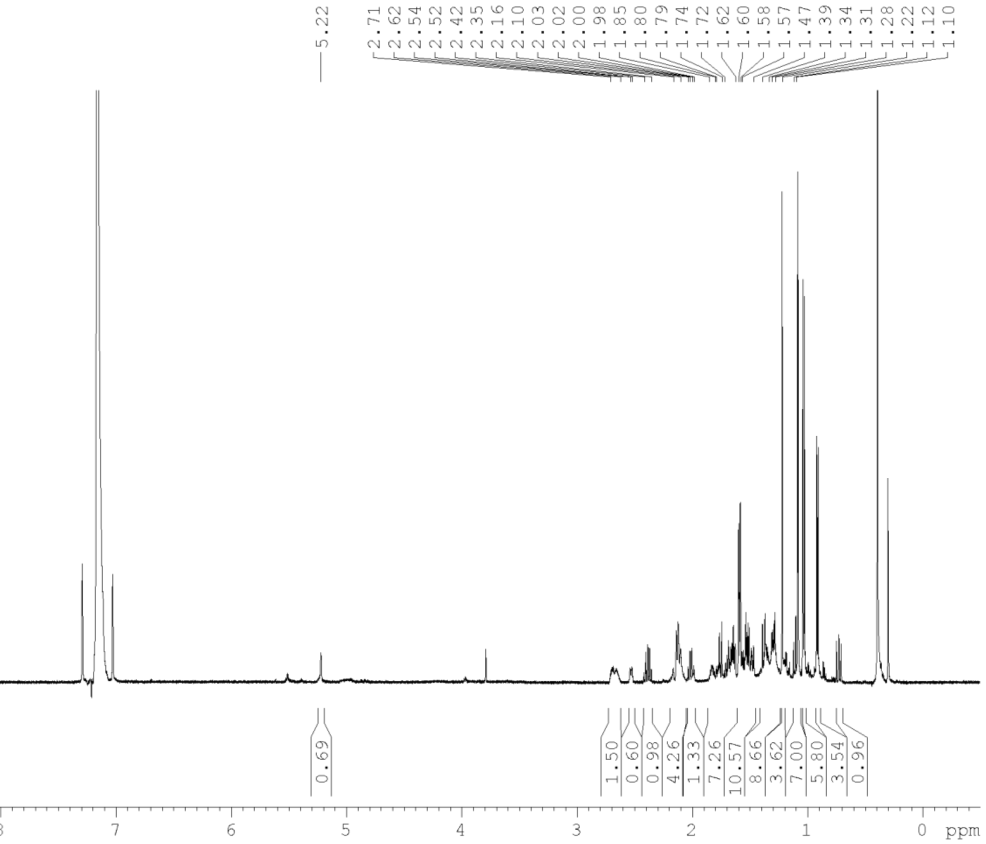
5.22
2.71
2.62
2.54
2.52
2.42
2.35
2.16
2.10
2.03
2.02
2.00
1.98
1.85
1.80
1.79
1.74
1.72
1.62
1.60
1.58
1.57
1.47
1.39
1.34
1.31
1.28
1.22
1.12
1.10
0.69
1.50
0.60
0.98
4.26
1.33
7.26
10.57
8.66
3.62
7.00
5.80
3.54
0.96
9 8 7 6 5 4 3 2 1 0 ppm

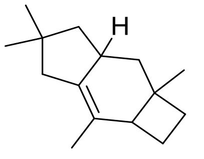

42

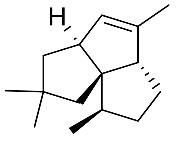

43

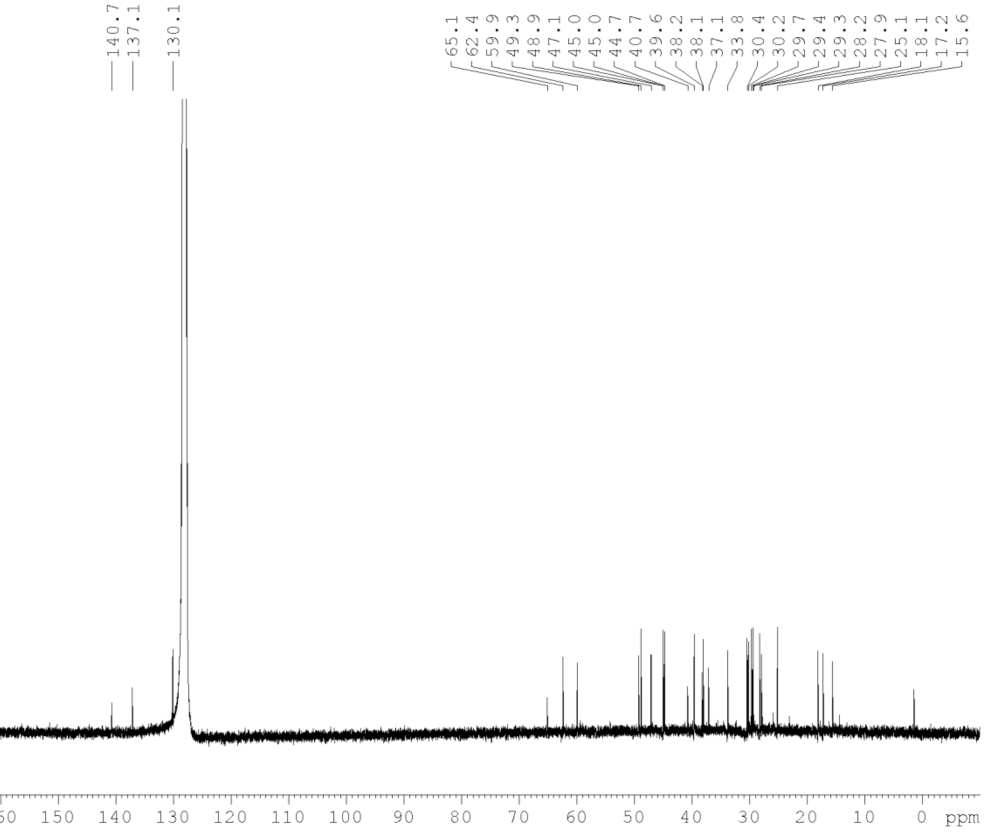
140.7
137.1
130.1
65.1
62.4
59.9
49.3
48.9
47.1
45.0
44.7
40.7
39.6
38.2
38.1
37.1
33.8
30.4
30.2
29.7
29.4
29.3
28.2
27.9
25.1
18.1
17.2
15.6
190 180 170 160 150 140 130 120 110 100 90 80 70 60 50 40 30 20 10 0 ppm

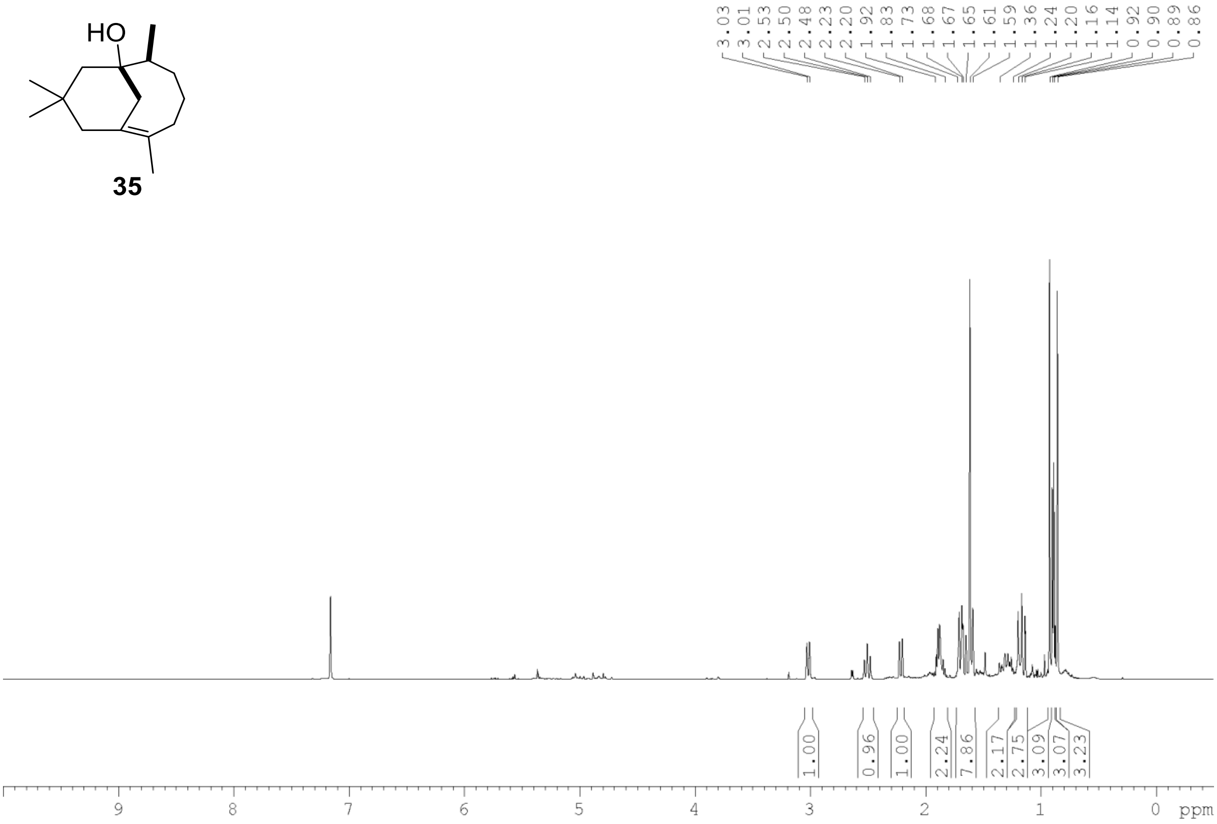
HO
35
3.03
3.01
2.53
2.50
2.48
2.23
2.20
1.92
1.83
1.73
1.68
1.67
1.65
1.61
1.59
1.36
1.24
1.20
1.16
1.14
0.92
0.90
0.89
0.86
1.00
0.96
1.00
2.24
7.86
2.17
2.75
3.09
3.07
3.23
9
8
7
6
5
4
3
2
1
0 ppm

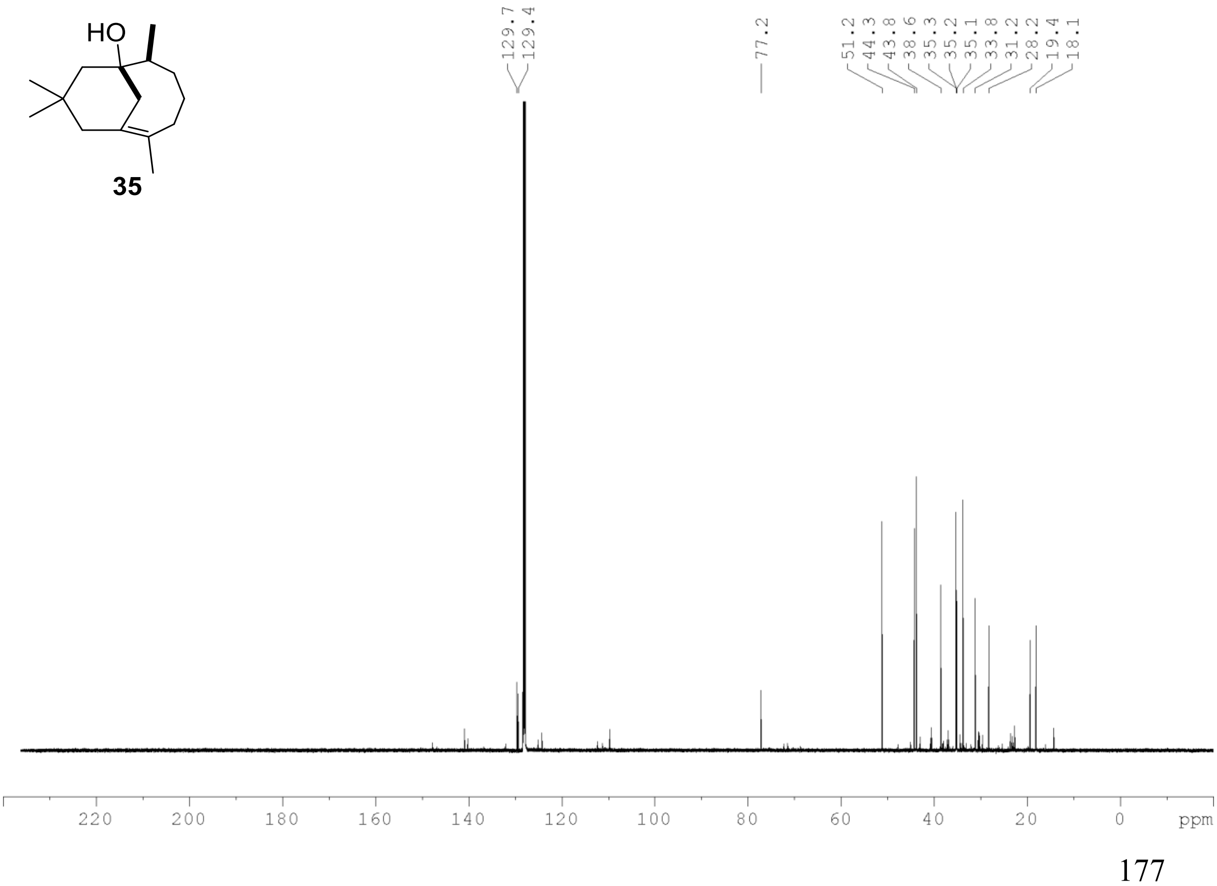
HO
35
129.7
129.4
77.2
51.2
44.3
43.8
38.6
35.3
35.2
35.1
33.8
31.2
28.2
19.4
18.1
220
200
180
160
140
120
100
80
60
40
20
0
ppm

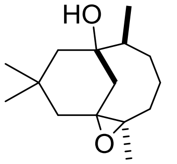
HO
O

36

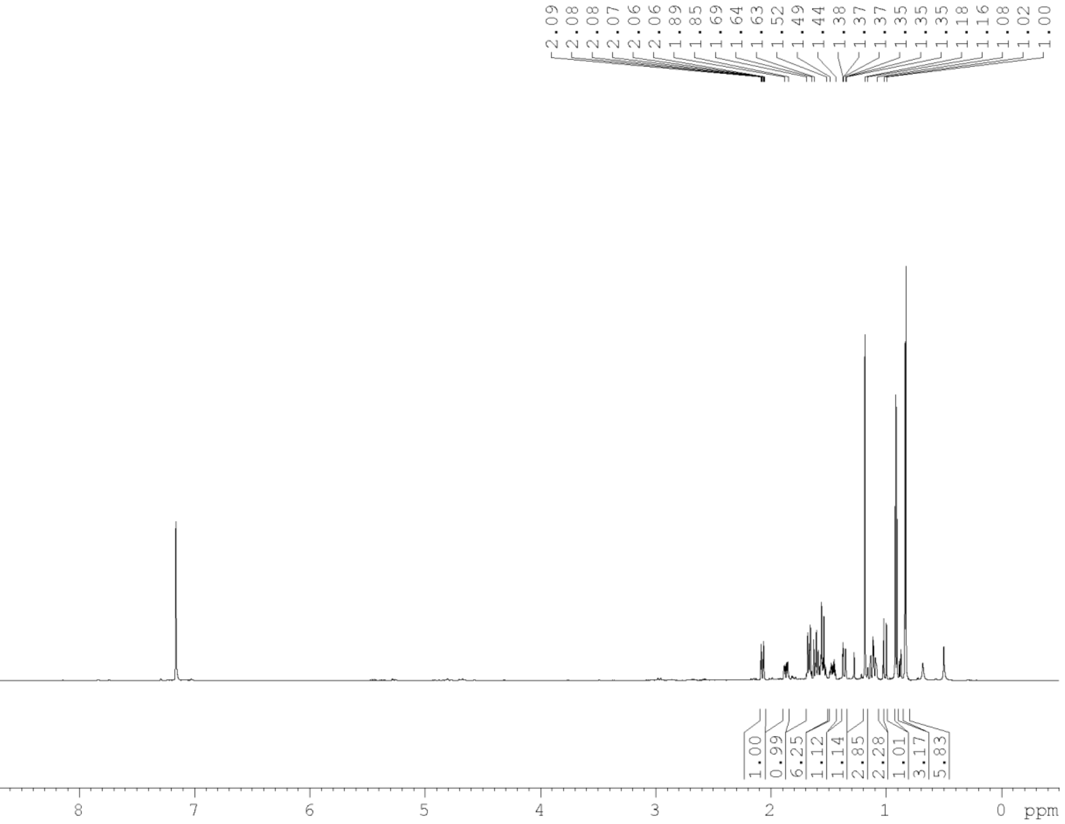
2.09
2.08
2.07
2.06
2.06
1.89
1.85
1.69
1.64
1.63
1.52
1.49
1.44
1.38
1.37
1.37
1.35
1.35
1.18
1.16
1.08
1.02
1.00
1.00
0.99
6.25
1.12
1.14
2.85
2.28
1.01
3.17
5.83
9
8
7
6
5
4
3
2
1
0 ppm

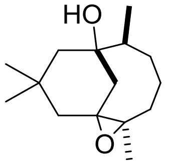
HO
O

36

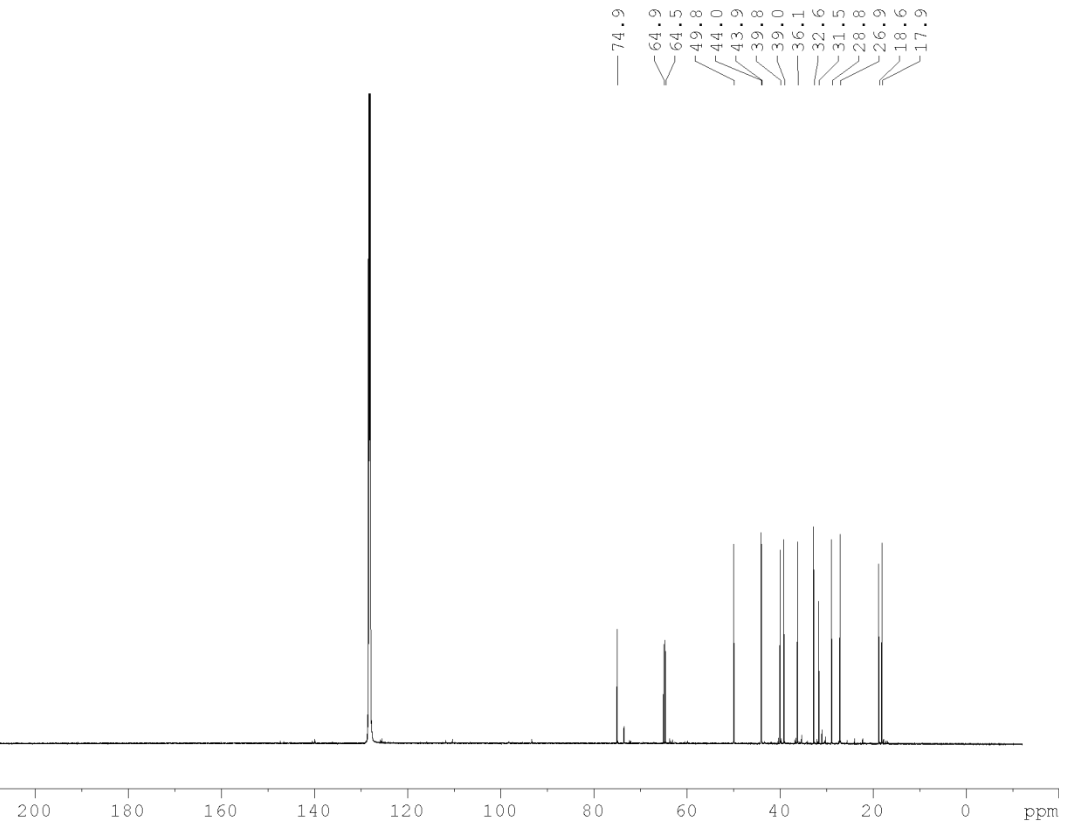
74.9
64.9
64.5
49.8
44.0
43.9
39.8
39.0
36.1
32.6
31.5
28.8
26.9
18.6
17.9
220
200
180
160
140
120
100
80
60
40
20
0
ppm

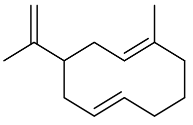

40

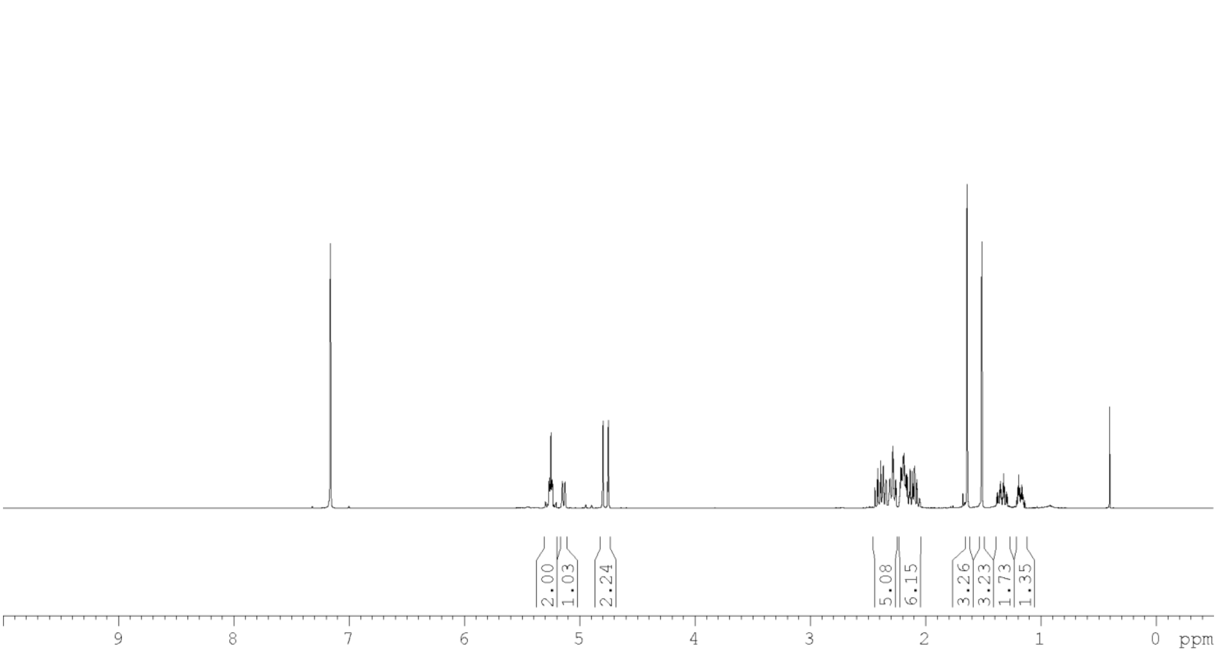
7.16
5.30
5.20
5.15
5.12
4.80
4.79
4.76
4.74
2.44
2.25
2.23
2.15
2.14
2.05
1.64
1.63
1.51
1.51
1.38
1.29
1.21
1.15
2.00
1.03
2.24
5.08
6.15
3.26
3.23
1.73
1.35
9 8 7 6 5 4 3 2 1 0 ppm

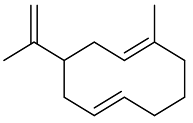

40

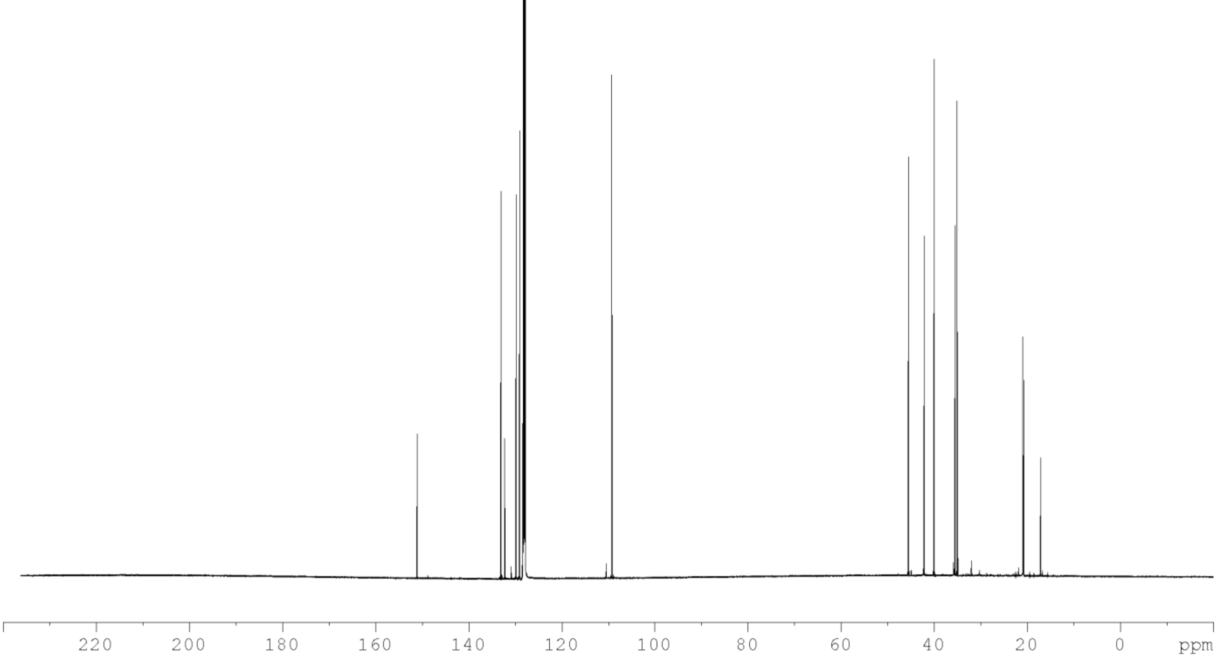
151.1
133.1
132.3
129.8
129.0
109.2
45.5
42.1
40.0
35.5
35.0
20.9
20.8
17.1
220 200 180 160 140 120 100 80 60 40 20 0 ppm

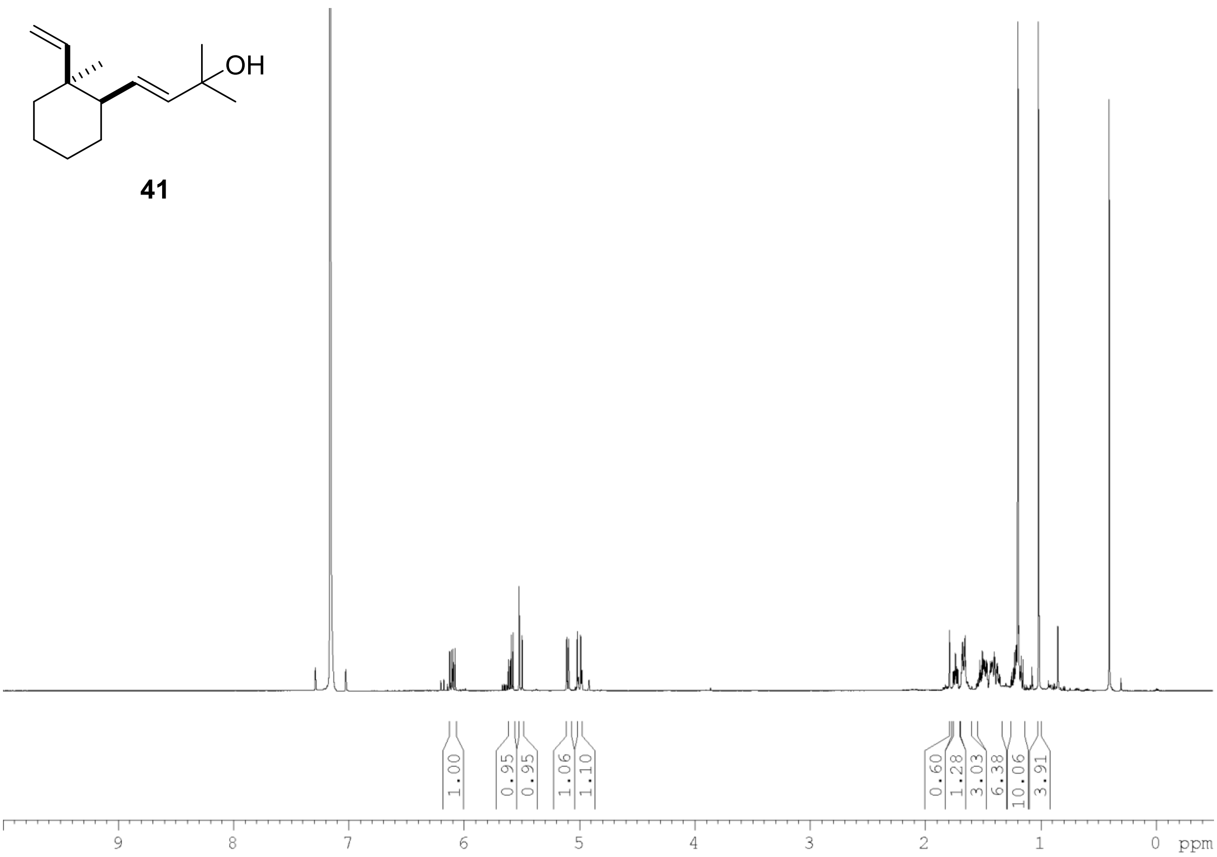
OH
1.00
0.95
0.95
1.06
1.10
0.60
1.28
3.03
6.38
10.06
3.91
9 8 7 6 5 4 3 2 1 0 ppm

41

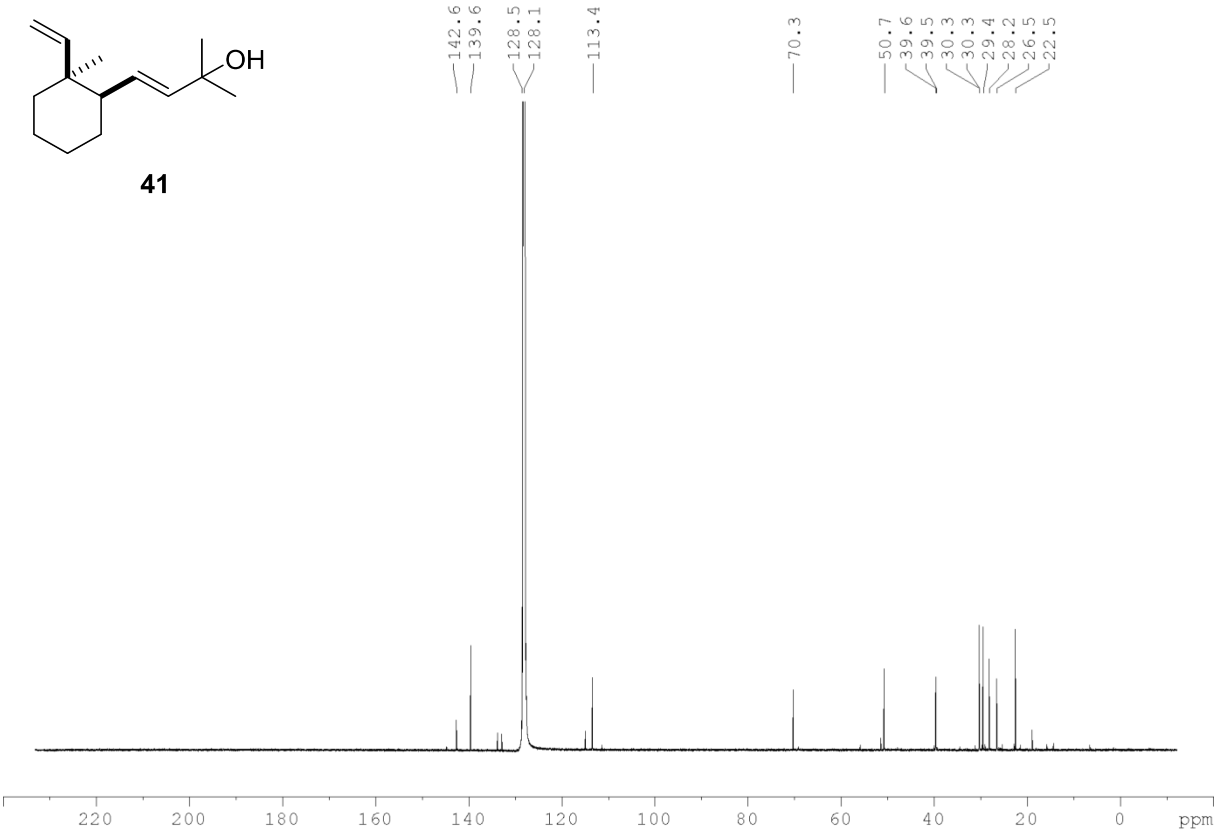
OH
142.6
139.6
128.5
128.1
113.4
70.3
50.7
39.6
39.5
30.3
30.3
29.4
28.2
26.5
22.5
220 200 180 160 140 120 100 80 60 40 20 0 ppm

41

3. References (supporting information)

[S1] Oberhauser, C.; Harms, V.; Seidel, K.; Schröder, B.; Ekramzadeh, K.;Beutel, S.; Winkler, S.; Lauterbach, L.; Dickschat, J. S.; Kirschning, A., Exploiting the Synthetic Potential of Sesquiterpene Cyclases for Generating Unnatural Terpenoids, *Angew. Chem. Int. Ed.* **2018**, *57*, 11802 – 11806.

[S2] Harms, Y.; Ravkina, V.; Kirschning, A., Mechanistic similarities of sesquiterpene cyclases PenA, Omp6/7, and BcBOT2 are unraveled by an unnatural "FPP-ether" derivative, O*rg. Lett.* **2021**, *23*, 3162 – 3166.

[S3] Lopez-Gallego, F.; Agger, S. A.; Abate-Pella, D.; Distefano, M. D.; Schmidt-Dannert, C., Sesquiterpene Synthases Cop4 and Cop6 from *Coprinus cinereus*: Catalytic Promiscuity and Cyclization of Farnesyl Pyrophosphate Geometric Isomers, *ChemBioChem* **2010**, *11*, 1093 – 1106.

[S4] Nakano, C.; Horinouchi, S.; Ohnishi, Y., Characterization of a Novel Sesquiterpene Cyclase Involved in (+)-Caryolan-1-ol Biosynthesis in *Streptomyces griseus.*

[S5] Wawrzyn, G. T.; Maureen, B. Q.; Choudhary, S.; López-Gallego, F.; Schmidt-Dannert, C., Draft Genome of *Omphalotus loearius* Provides a Predictive Framework for Sesquiterpenoid Natural Product Biosynthesis in Basidiomycota, *Chem. Biol.* **2012**, *19*, 772 – 783.

[S6] Yan, X.; Zhou, J.; Ge, J.; Li, W.; Liang, D.; Singh, W.; Black, G.; Nie, S.; Liu, J.; Sun, M.; Qiao, J.; Huang, M., Computer-Informed Engineering: A New Class I Sesquiterpene Synthase JeSTS4 for the Synthesis of an Unusual C10-(*S*)-Bicyclogermacrene, *ACS Catal.* **2022**, *12*, 4307 – 4045.

[S7] Cao, Y.; Zhang, R.; Liu, W.; Zhao, G.; Niu, W.; Guo, J.; Xian, M.; Liu, H.; Manipulation of the precursor supply for high-level production of longifolene by metabolically engineered *Escherichia coli*, *Sci. Rep.* **2019**, *9*, 95.

[S8] Kertsen, R. D.; Shoukou, L.; Fujita, D.; Pluskal, T.; Kram, S.; Smith, J. E., Iwai, T.; Noel, J. P., Fujita, M.; Weng, J.-K., A Red Algal Bourbonane Sesquiterpene Synthase Defined by Microgram-Scale NMR-Coupled Crystalline Sponge X-ray Diffraction Analysis, *J. Am. Chem. Soc.* **2017**, *139*, 16838 – 16844.

[S9] Hartwig, S.; Frister, T.; Alemdar, S.; Li, Z.; Krings, U.; Berger, R. G.; Scheper, T.; Beutel, S., Expression, purification and activity assay of a patchoulol synthase cDNA variant fused to thioredoxin in *Escherichia coli*, *Protein Expr. Purif.* **2014**, *97*, 61 – 71.

[S10] Falara, V.; Akhtar, T. A.; Nguyen, T. T. H.; Spyropoulou, E. A.; Bleeker, P. M., Schauvinhold, I.; Matsuba, Y.; Bonini, M. E.; Schilmiller, A. L.; Last, R. L., Schuurink, R. C.; Pichersky E., The Tomato Terpene Synthase Gene Family, *Plant Physiol.* **2011**, *157*, 770 – 789.

[S11] Struwe, H.; Schrödter, F.; Spinck, H.; Kirschning, A., Sesquiterpene Backbones Generated by Sesquiterpene Cyclases: Formation of *iso*-Caryolan-1-ol and an Isoclovane, *Org. Lett.* **2023**, *25*, 8575 – 8579.

[S12] Struwe, H.; Li, H.; Schrödter, F.; Höft, L., Fohrer, J.; Dickschat, J. S.; Kirschning, A., Telescoping a Prenyltransferase and a Diterpene Synthase to Transform Unnatural FPP Derivatives to Diterpenoids, *Org. Lett.* **2024**, *26*, 5888 – 5892.

[S13] Hou, A.; Dickschat, J. S., Using Terpene Synthase Plasticity in Catalysis: On the Enzymatic Conversion of Synthetic Farnesyl Diphosphate Analogues, *Chem. Eur. J.* **2021**, *27*, 15644 – 15649.

[S14] Kitahara, T.; Mori, M.; Mori, K., Total Synthesis of (−)-Periplanone-B, Natural Major Sex-Excitant Pheromone of the American Cockroach, *Periplaneta Americana*, *Tetrahedron* **1987**, *43*, 2689 – 2699.

[S15] Harmata, M.; Bohnert, G. J., A 4+3 Cycloaddition Approach to the Synthesis of (±)-Sterpurene, *Org. Lett.* **2003**, *5*, 59 – 61.

[S16] Ohfune, Y.; Shirahama, H.; Matsumoto, T., Syntheses of 7α- and 7β-protoilludanol, and 7(13)-protoilludene, possible biogenetic intermediates for illudoid sesquiterpenes, *Tetrahedron Lett.* **1975**, *16*, 4377 – 4380.
